# Investigations into linker effects of DNA–VHL ligand conjugates by multiplexed affinity measurements using focal molography

**DOI:** 10.1039/d6cb00011h

**Published:** 2026-03-20

**Authors:** Pascal Raschke, Simona Notova, Volker Gatterdam, Andreas Frutiger, Andreas Brunschweiger

**Affiliations:** a Institute of Pharmacy and Food Chemistry, Julius-Maximilians-Universität Würzburg, Am Hubland 97074 Würzburg Germany andreas.brunschweiger@uni-wuerzburg.de; b lino Biotech AG Soodstrasse 52 8134 Adliswil Switzerland

## Abstract

The determination of kinetic binding properties of DNA-tagged compounds binding to a target protein is an important step in the development of nucleic acid-based PROTACs. Focal molography (FM) is a recent addition to the toolbox of instruments that allow for measuring kinetic binding data of drugs binding to a target protein with high experimental throughput. Here, we applied focal molography to characterize the binding of DNA–VHL ligand conjugates—these are “DNA–PROTAC” compounds—to VHL. We synthesized two libraries of 20 such compounds with diverse amino acid linkers by solid-phase amide coupling and used FM to measure their affinity to VHL in both singleplex and 20-plex multiplexed measurement formats. Systematic comparison of equilibrium and kinetic fitting approaches reveals strong within-format correlations that preserve compound rankings despite systematic offsets in absolute *K*_D_ values. The multiplexed format achieves 20-fold higher throughput while yielding stronger structure–activity correlations with lipophilicity compared to singleplex measurements. Our analysis identifies hydrophobicity as the primary driver of the contribution of the linker part to overall linker-VHL ligand affinity for VHL in the case of DNA–PROTACs. These findings validate multiplexed FM as a robust platform for PROTAC structure–activity relationship studies and provide design principles for optimizing linker–E3 ligase interactions.

## Abstract 2

We measured the affinity of linker-diversified DNA–PROTACs to VHL by focal molography. Our analysis validates multiplexed affinity measurements and identifies hydrophobicity of the linker as the primary driver to linker-VHL ligand affinity for VHL.

## Graphical abstract

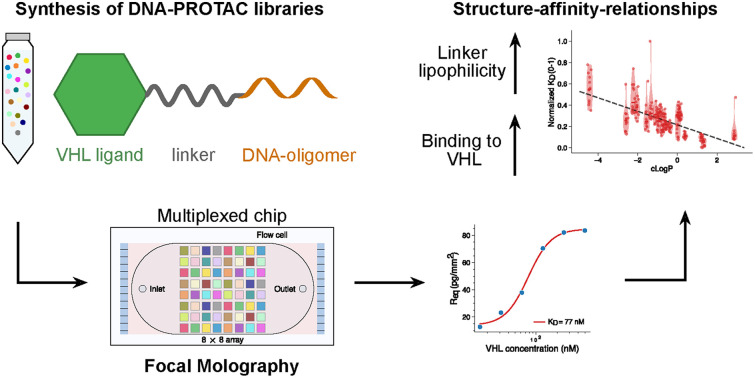

## 1. Introduction

Oligonucleotide conjugates have gained in importance both as part of technologies for drug discovery and as drug modalities themselves. For instance, oligonucleotide small molecule conjugates are the workhorse structures of DNA-encoded libraries,^1^ they are widely used for targeting the oligonucleotide to a tissue,^2^ and lately, oligonucleotide-PROTACs have been described that allow a DNA or RNA to degrade its cellular protein binding partner(s).^3–7^ Often, determination of the affinity of the conjugated compound for its biological target is a key question in a drug discovery project that uses nucleic acids as technology or modality component. Technologies that allow for rapid and ideally parallelized measurement of kinetic binding data for oligonucleotide conjugates would streamline compound optimization in such settings. Previously, surface plasmon resonance (SPR) has been used to determine the affinity of biotinylated RNA to streptavidin.^8^ For the validation and kinetic binding data measurements of DNA-coupled conjugates that were initially identified as hits from DNA-encoded library screens, a few methods have been demonstrated, including chip-based sensor technology,^9^ microscale thermophoresis (MST), fluorescence anisotropy (FA), alphascreen,^10^ enzyme-linked immunosorbent assay (ELISA), and SPR.^11,12^ Notably, the DNA tag attached to these compounds could itself serve as a handle for programmable self-assembly onto sensor surfaces, enabling rapid multiplexed array formation through Watson–Crick base pairing without complex spotting procedures.

**Fig. 1:**
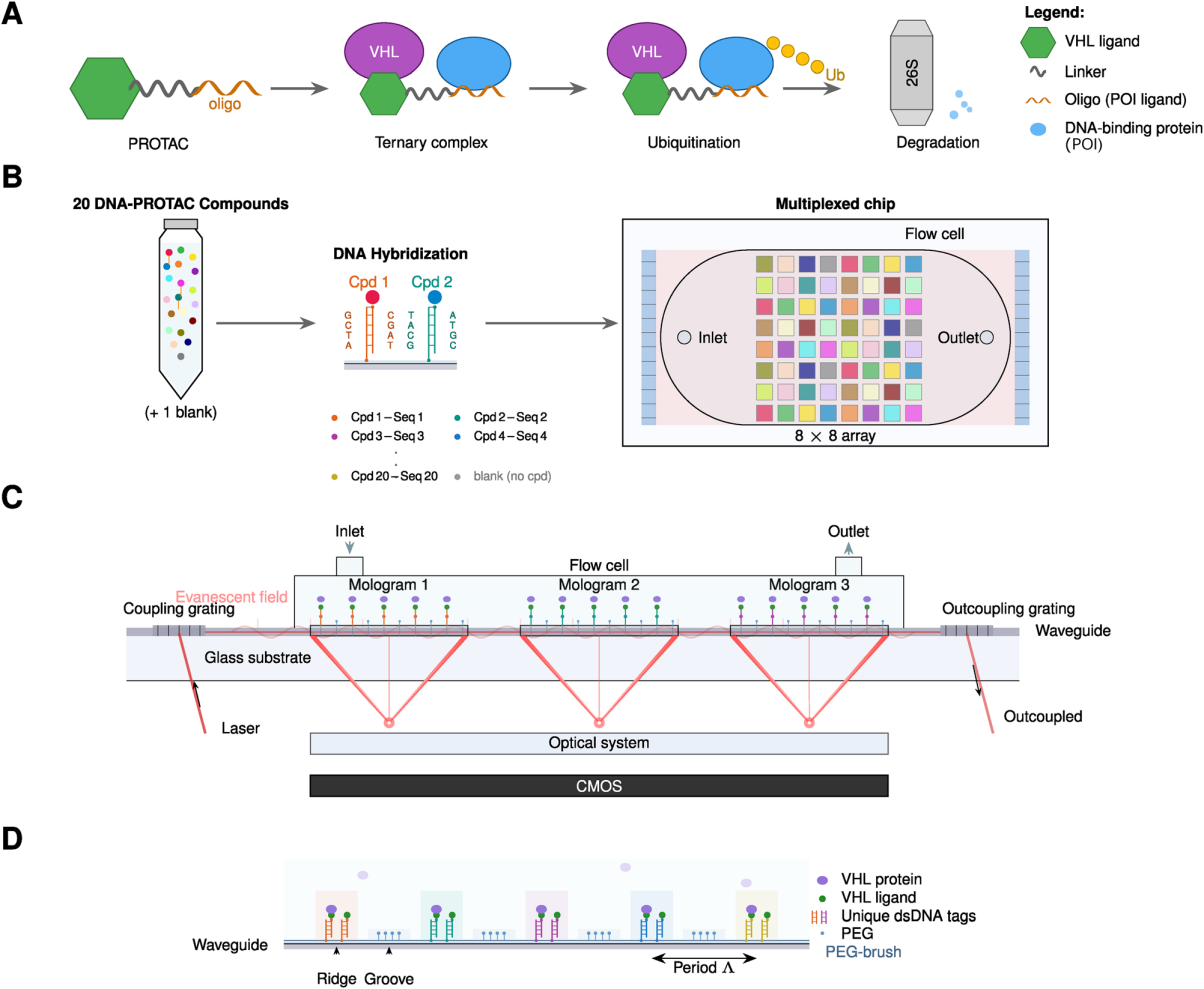
PROTAC mechanism and multiplexed focal molography workflow for affinity measurements. (A) PROTAC mechanism: a PROTAC molecule consists of an E3 ligase ligand (green hexagon) connected *via* a linker (grey) to a protein-of-interest (POI) ligand (in case of “DNA–PROTACs” here: orange singlestrand oligo). The PROTAC recruits both the E3 ligase (here VHL) and POI into a ternary complex, leading to ubiquitination of the POI and its subsequent degradation by the 26S proteasome. (B) Multiplexed self-assembly affinity measurement workflow: a pool of 20 DNA-tagged compounds is injected onto a chip pre-functionalized with complementary capture sequences. Watson–Crick hybridization directs each compound to its designated mologram position on an 8 × 8 array, enabling simultaneous measurement of all compounds in a single experiment. (C) Side view of the waveguide-based optical setup: laser light is coupled into a thin-film waveguide *via* a coupling grating and propagates with an evanescent field extending into the sample medium. Molograms—diffractive sensing elements functionalized with DNA-ligand–protein complexes—scatter light coherently to focal points detected by a CMOS camera. (D) Binding modulation principle: the mologram surface is chemically patterned with ridges containing double-stranded DNA (dsDNA) tags linked to VHL ligands that bind target protein, alternating with grooves passivated by PEG. This spatial modulation creates the coherent diffraction signal.

Focal molography (FM) is a label-free biosensing technology for real-time measurement of molecular interactions.^13–15^ The technique detects binding events through coherent light diffraction from a “mologram”—a sub-micrometer pattern of molecular recognition sites on a sensor chip (Fig. 1). Molograms are fabricated by structured light deprotection of surface-bound amines, enabling site-selective functionalization with capture molecules.^14,16^ When target molecules bind to these patterned sites, they create a periodic refractive index modulation that diffracts laser light into a focused spot; the intensity of this spot quantifies the amount of bound material.^17^

A key advantage of FM over established refractometric methods such as surface plasmon resonance (SPR) is its inherent rejection of non-specific binding.^15,18–20^ In SPR, all molecules adsorbed to the sensor surface contribute to the measured signal, necessitating careful blocking and reference subtraction. In contrast, non-specifically adsorbed molecules distribute randomly across the mologram surface and do not contribute to the coherent diffraction signal—only molecules bound at the patterned recognition sites generate signal. This selectivity enables reliable measurements directly in crude biological samples such as serum,^21,22^ and even in living cells.^23,24^ Recent studies have validated that FM provides kinetic constants highly comparable to SPR and bio-layer interferometry (BLI) in standard buffers, while demonstrating superior robustness in complex matrices.^21,25^

For multiplexed applications, FM offers a particularly streamlined workflow. Pools of up to 64 DNA-tagged ligands can be injected onto a chip where programmed Watson–Crick base pairing localizes each conjugate at a specific mologram position (Fig. B1, SI). The binding affinity of an unlabelled protein to all immobilized compounds is then measured simultaneously. Using 20-mer oligonucleotides provides duplex melting temperatures well above assay conditions, resulting in quasi-covalent immobilization that remains stable throughout the measurement.

Oligonucleotide–von Hippel–Lindau (VHL) ligand conjugates are being developed as an emerging modality for the targeted degradation of nucleic acid-binding proteins.^4,5^ In our research program for nucleic acid-E3 ligase ligands we investigate synthesis strategies and linkers for the design of these molecules. The design of linkers can have a large impact on the biological activity of PROTAC molecules.^26,27^ Previously, nucleic acid-PROTACs were synthesized using click chemistry (CuAAC) that requires azides and alkynes,^6,28–31^ by thiol–ene coupling reactions that require an electron-poor olefin (“Michael acceptor”) and a thiol,^5^ or by coupling dedicated phosphoramidites to the 5′-terminus of the oligonucleotide.^32^ Amide coupling strategies have been reported, but with a lack of linker variety as the VHL warhead was directly coupled to the oligonucleotide.^5^ We aimed at a synthesis strategy that allows for rapidly generating libraries of oligonucleotide-linker-VHL ligand conjugates which makes use of the availability of amino acid linkers to assess the impact of different linker structures on the affinity of DNA–PROTAC compounds for VHL.^26,33^ Systematic investigation of linker effects on binding affinity would benefit from higher experimental throughput than traditional methods such as isothermal titration calorimetry (ITC) or SPR can provide.

In this study, we synthesized two DNA–PROTAC libraries by solid-phase amide coupling using diverse amino acid linkers and systematically compared singleplex and multiplexed FM for characterizing their binding to VHL. By measuring 20 compounds using both formats and analyzing data with equilibrium and kinetic fitting approaches, we establish the validity of multiplexed measurements for structure–activity relationship (SAR) studies and identify physicochemical properties that govern linker-VHL binding affinity.

## 2. Results and discussion

### 2.1. Chemistry

**Fig. 2:**
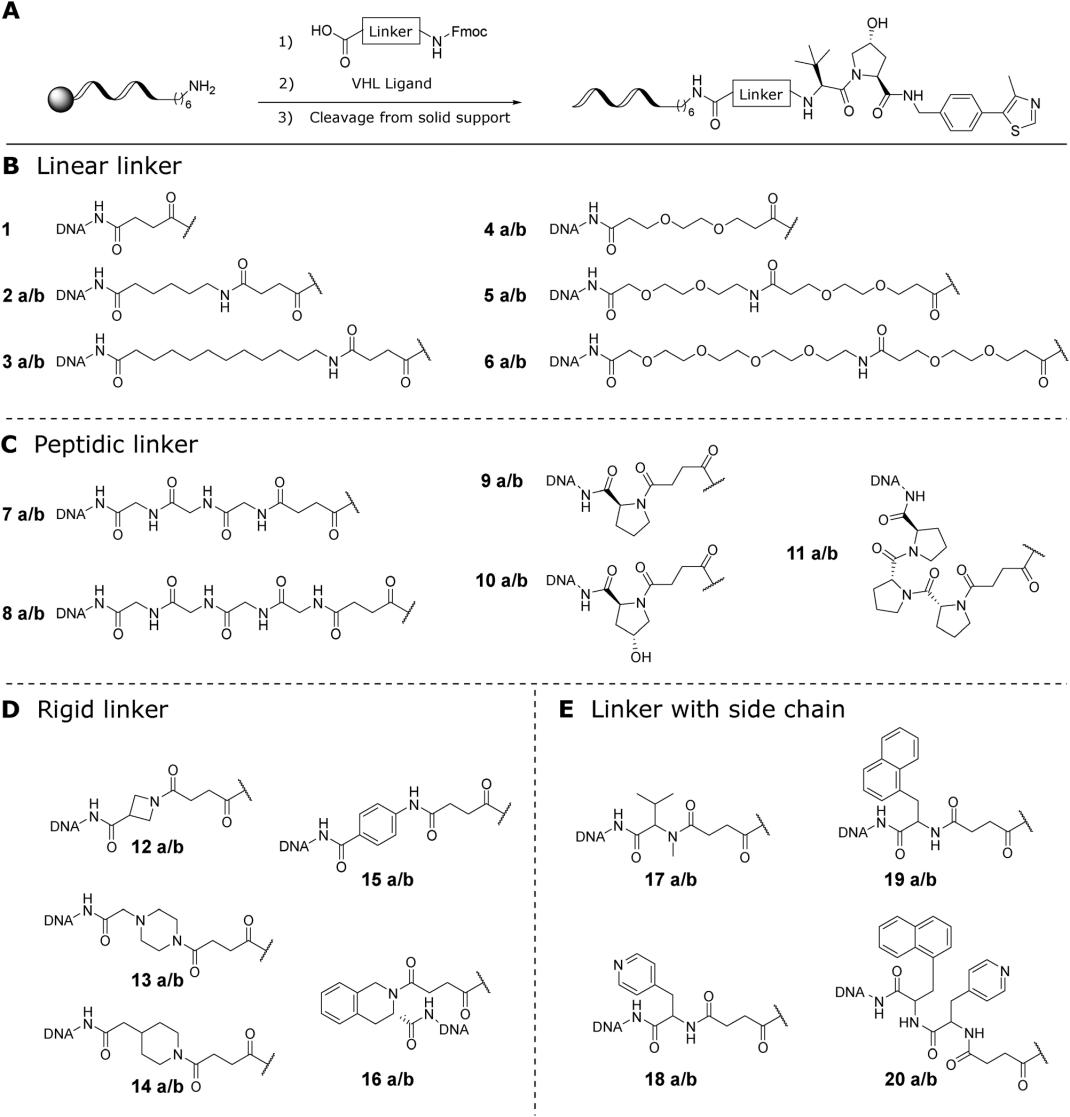
Synthesis of the DNA–PROTAC libraries (A) and chemical structures of linkers grouped by chemical structure (B–E). DNA = hexyl-20mer DNA. Library 1 contained compounds 1, 2a–20a with the same oligonucleotide sequence attached, while library 2 compounds 1, 2b–20b were tagged with unique DNA sequences for multiplexing. The oligonucleotide-linker conjugates were prepared using *ca.* 10 nmol aminohexyl-20mer DNAs, 100 equivalents of Fmoc-protected amino acid, 100 equivalents of coupling reagent HATU and 250 equivalents of DIPEA as base (1 h, 37 °C). No linker was attached for compounds 1 and 4a/b. Fmoc deprotection was achieved with piperidine (20% v/v in DMF). Finally, the VHL ligand carboxylic acid (VH032 amide-alkylC2-acid for compounds 1, 2a/b–3a/b and 7a/b–20a/b; VH032 PEG-2 carboxylic acid for compounds 4a/b–6a/b) was attached by a second amide coupling. The conjugates were cleaved from solid support using AMA solution and purified by semi-preparative RP-HPLC. Identity and purity were confirmed by LC-MS (see SI).

Two DNA–PROTAC libraries were synthesized by a solid-phase amide coupling strategy which taps into the rich pool of readily available amino acid linkers for PROTAC design. We selected a library of diverse Fmoc-protected amino acid linkers that are depicted in Fig. 2. They contained hydrophobic and hydrophilic (ethylene glycol) linear chains, oligo-glycines, oligo-prolines, rigid heterocyclic and aromatic linkers as well as linkers with mostly non-polar sidechains. These linkers were coupled by Fmoc peptide chemistry using HATU as coupling reagent to a single 20mer DNA oligomer (library 1: compounds 1, 2a–20a) and to 20 different DNA sequences for multiplexing (library 2: compounds 1, 2b–20b) on CPG solid support as previously published.^34^ The linker conjugates were Fmoc-deprotected with piperidine in DMF on the solid phase and a VHL ligand was coupled in the final synthesis step. After cleavage of the products from the solid phase, all DNA conjugates were purified by RP-HPLC. This scalable two-step coupling procedure allowed for library synthesis within a week. The identity and purity of the library was confirmed by LC-MS (see Fig. A8–A46, SI). Most Fmoc amino acid linkers were coupled to the amino-linker DNA with quantitative conversions to the amide, while product formation after the second coupling varied depending on the linker attached beforehand. While DNA conjugates with more bulky and longer linkers tended to show lower reaction efficiency, with *in situ* yields of 30–40% for compounds 3, 16, 18, 19, and 20, and as low as 10% for compound 17, shorter and linear linkers gave higher product yields (*in situ* yields > 60%). Importantly, the VHL E3 ligase ligand was not harmed by the cleavage conditions, which contrasted with a report on the hydrolysis of IMiD E3 ligase ligands,^32^ making our synthesis strategy a viable option for rapid compound synthesis. All compounds were obtained in purities suitable for affinity measurements.

### 2.2. Binding affinity determination across experimental formats

To comprehensively characterize the binding affinities of DNA–PROTAC compounds to VHL, we employed two experimental formats—singleplex and 20-plex multiplexed FM—and analyzed the data using both equilibrium and kinetic fitting approaches. This systematic comparison enabled assessment of *K*_D_ value consistency across different measurement strategies and identification of potential format-specific effects. We discuss the experimental protocol using the example of the multiplexed experiments; the singleplex experiments are described in the SI Part B.

**Fig. 3:**
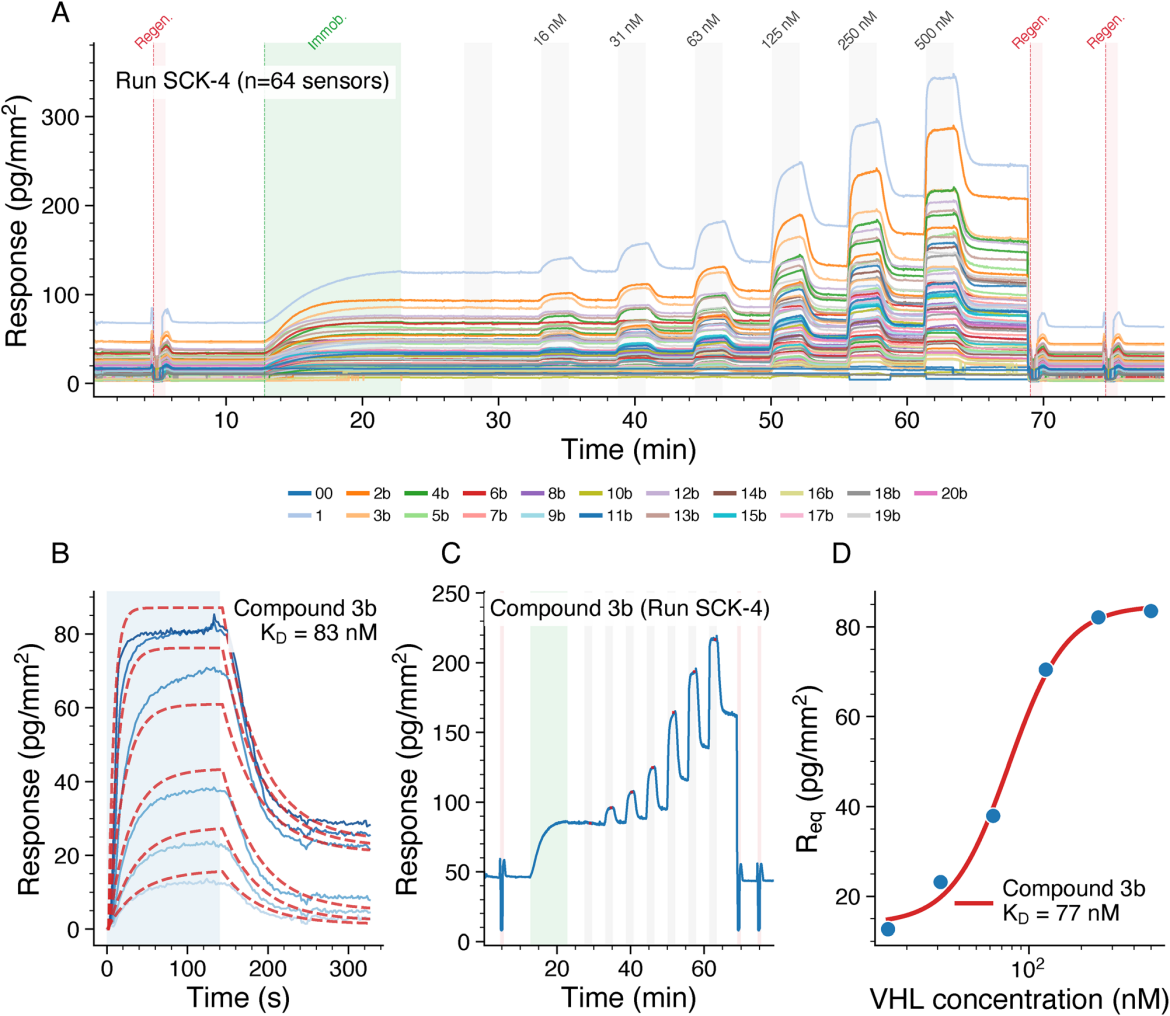
Multiplexed binding analysis (Run SCK-4). (A) Multiplexed sensorgram overview showing all 64 sensors (21 compounds in triplicate plus negative control), colored by compound identity. Green shading indicates DNA-ligand immobilization; gray shading indicates VHL injection phases; pink shading indicates regeneration. (B) Representative raw sensorgram for compound 3b showing binding to VHL at increasing concentrations (15.6–500 nM). Green shading indicates immobilization; pink shading indicates regeneration phases; gray shading indicates VHL injection phases; red dots mark steady-state regions. (C) Multi-cycle kinetic analysis for compound 3b: individual concentration cycles (solid blue lines, darker shades = higher concentrations) with global 1 : 1 kinetic fit (red dashed lines), yielding *K*_D_ = 83 nM. Blue shading indicates association phase. (D) Equilibrium binding isotherm for compound 3b showing steady-state response *versus* VHL concentration fitted to a 4-parameter Hill equation (red line), yielding *K*_D_ = 77 nM.

Fig. 3 presents the multiplexed binding analysis workflow. For the multiplexed measurements, all 20 DNA–PROTAC compounds were mixed and injected as one complex mixture onto the chip, where Watson–Crick hybridization directed each compound to its designated mologram position. Additionally, one reference oligonucleotide (00) without attached compound was included as a negative control; this blank mologram showed no signal during compound immobilization and no response upon VHL injection (Fig. B8, SI), confirming that FM selectively detects ordered molecules on the diffractive pattern while rejecting non-specific surface binding. Target specificity was further validated by injecting bovine serum albumin (BSA) at concentrations up to 100 μM across all compound-loaded molograms; no concentration-dependent binding was observed (Fig. B24, SI). This negative control, combined with FM's inherent rejection of non-specific binding through coherent detection,^18^ provides confidence that the observed signals arise from specific compound–VHL interactions. The coherent structure–activity relationships observed between binding affinity and lipophilicity (Fig. 6) further support assay specificity, as non-specific binding would not produce such systematic trends. The multiplexed format enabled simultaneous measurement of all 20 compounds in triplicate on a single sensor chip (Fig. 3A). The raw sensorgram for compound 3b shows binding responses at increasing VHL concentrations, with clear plateaus indicating that equilibrium was reached at each concentration step (Fig. 3B). Kinetic fitting using a global 1 : 1 binding model yielded *K*_D_ values directly from the association and dissociation phases (Fig. 3C), while the steady-state response values were fitted to a binding isotherm to obtain *K*_D_ by equilibrium analysis (Fig. 3D).

**Fig. 4:**
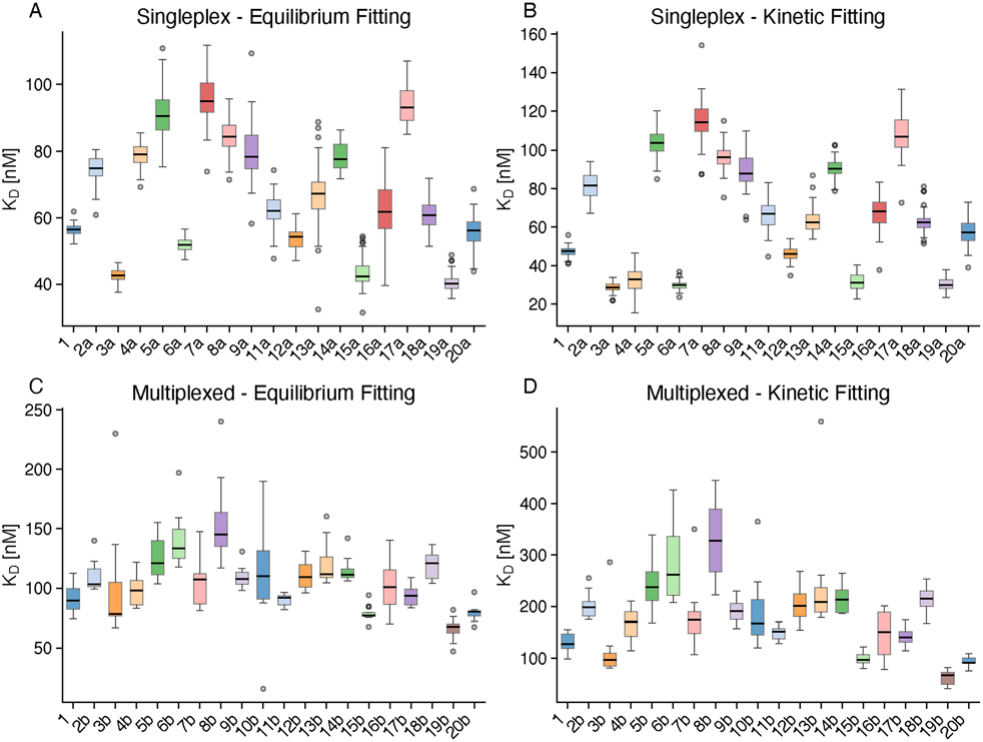
*K*_D_ distributions across analysis methods. Boxplots showing *K*_D_ values (nM) for each compound measured by (A) singleplex equilibrium fitting, (B) singleplex kinetic fitting, (C) multiplexed equilibrium fitting, and (D) multiplexed kinetic fitting. Boxes indicate interquartile range with median line; whiskers extend to 1.5× IQR; outliers shown as individual points. Sample sizes indicated above each box. Singleplex experiments used “a” suffix compounds (*n* = 54 measurements per compound); multiplexed used “b” suffix compounds (*n* = 12 per compound).

Fig. 4 shows the distribution of *K*_D_ values for each compound across the four analysis methods. The singleplex format, measuring one compound per chip with 54 independent measurements, yielded *K*_D_ values ranging from 20–160 nM. In contrast, the 20-plex multiplexed format, simultaneously measuring 20 compounds per chip with approximately 12 measurements per compound (triplicates in 4 independent runs), produced systematically higher *K*_D_ values ranging from 50–550 nM.

Notably, the singleplex experiments utilized compounds with “a” suffix linker variants (2a, 3a, 4a, *etc.*), while the multiplexed experiments employed the structurally similar (different DNA codes for immobilization) “b” suffix variants (2b, 3b, 4b, *etc.*). Compound 1, which served as a reference with the same DNA tag, was measured in both formats. Despite differences in absolute *K*_D_ values between formats, the relative ranking of compounds by binding affinity remained consistent, with compounds 3, 15, and 19 consistently identified as the strongest binders across all four methods (Table 1). The binding data showed that all linkers were tolerated by VHL, with *K*_D_ values ranging from 20–550 nM depending on the measurement format, in good agreement with published *K*_D_ values for VH032 of 185 nM^27^ and 180–300 nM by ITC and SPR.^35,36^ The systematic differences between singleplex and multiplexed *K*_D_ ranges and their implications for absolute accuracy *versus* relative ranking are discussed in detail in the cross-format comparison below. Importantly, these results demonstrated that the design of future nucleic acid-based PROTACs is not limited by linker constraints imposed by VHL binding.

**Table 1:** Median *K*_D_ values (nM) for representative compounds across analysis methods. Compounds are ranked by multiplexed equilibrium *K*_D_ (best binders first) due to the highest correlation with lipophilicity of this evaluation method (Fig. 6)

Compound	Singleplex		Multiplexed	
	Equilibrium	Kinetic	Equilibrium	Kinetic
19a/19b	40.1	29.8	67.8	67.4
15a/15b	42.4	31.2	76.9	97.3
3a/3b	42.7	28.7	78.4	97.0
1	56.5	47.5	89.5	127.5
6a/6b	51.8	29.9	133.5	261.4
8a/8b	84.5	96.2	145.0	327.4

### 2.3. Comparison of evaluation methods and formats

**Fig. 5:**
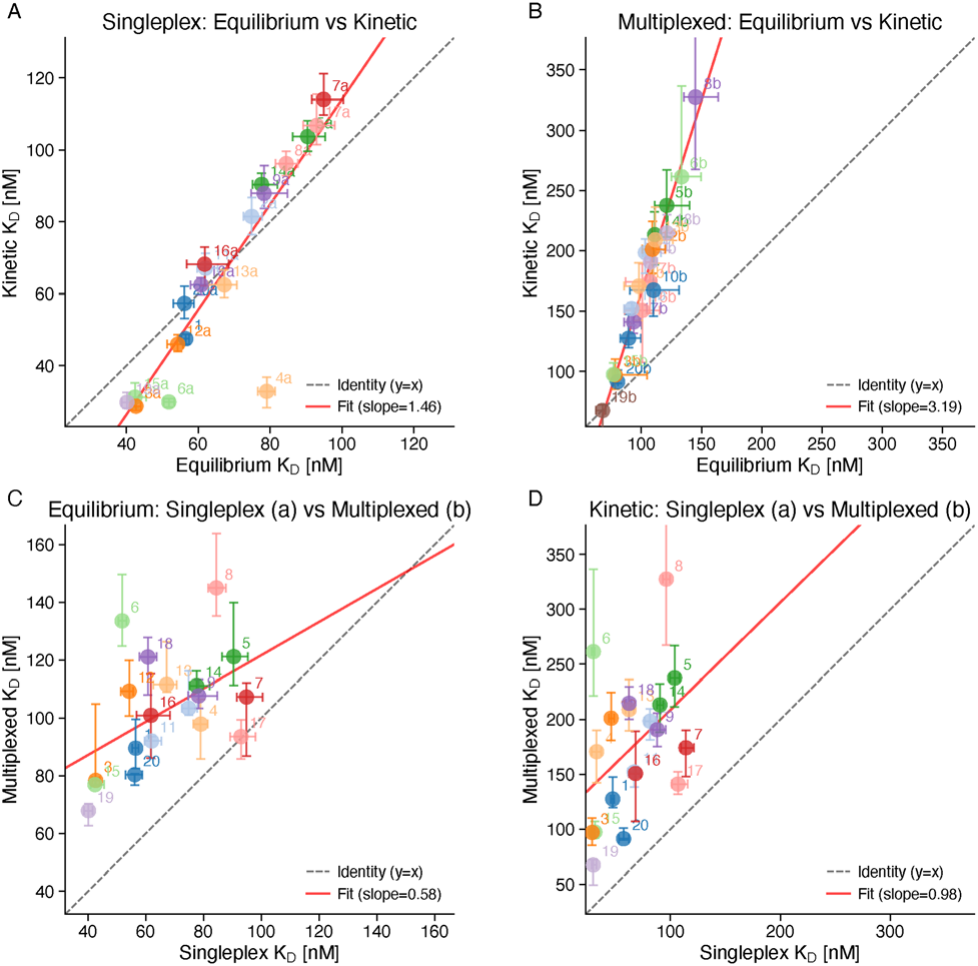
Method and format comparisons. Correlation plots comparing *K*_D_ values between methods and formats. (A) Singleplex: equilibrium *vs.* kinetic fitting (*r* = 0.88, *p* < 0.001). (B) Multiplexed: equilibrium *vs.* kinetic fitting (*r* = 0.98, *p* < 0.001). (C) Equilibrium fitting: singleplex “a” variants *vs.* multiplexed “b” variants (*r* = 0.50, *p* = 0.028). (D) Kinetic fitting: singleplex *vs.* multiplexed (*r* = 0.43, *p* = 0.065). Solid lines indicate linear regression; dashed lines show identity (*y* = *x*). Each point represents median *K*_D_ for one compound, colored by compound identity.

To evaluate whether the choice of evaluation method influences the determined *K*_D_ values, we directly compared equilibrium and kinetic fitting results within each experimental format (Fig. 5A and B). Within the singleplex format, equilibrium and kinetic fitting showed strong correlation (Pearson *r* = 0.88, *p* = 5.9 × 10^−7^), with a mean kinetic/equilibrium ratio of 0.95, indicating near-equivalence of the two approaches.

The multiplexed format exhibited even stronger correlation between evaluation methods (*r* = 0.98, *p* = 1.8 × 10^−13^), demonstrating excellent internal consistency. However, kinetic fitting in the multiplexed format yielded systematically higher *K*_D_ values, with a mean kinetic/equilibrium ratio of 1.64.

These results demonstrated that while absolute *K*_D_ values may differ between evaluation approaches, compound rankings are preserved. This finding has practical implications: equilibrium fitting may be preferred when equilibrium can be reached and kinetics are not of primary interest, or when the protein shows strong sticking to the sensor surface, while either method is suitable for SAR studies where relative rankings are of primary interest.

#### 2.3.1. Cross-format comparison

To assess whether singleplex and multiplexed formats produce comparable results, we matched “a” and “b” linker variants by their base compound number and compared *K*_D_ values across formats (Fig. 5C and D). The cross-format correlations were notably weaker than within-format method comparisons: equilibrium fitting showed *r* = 0.50 (*p* = 0.028), while kinetic fitting showed *r* = 0.43 (*p* = 0.065, not significant at *α* = 0.05). Spearman rank correlations yielded *ρ* = 0.41 (*p* = 0.090) and *ρ* = 0.37 (*p* = 0.135), respectively, indicating that while the overall rank ordering is only moderately preserved, this is expected given the compressed dynamic range in the multiplexed format.

The multiplexed format consistently yielded higher *K*_D_ values than singleplex, with ratios of approximately 1.6× for equilibrium fitting and 3× for kinetic fitting. Several factors may contribute to this systematic difference: (1) different surface densities of immobilized ligands and chip fabrication effects—the multiplexed chips were spotted whereas the singleplex chips were drop-casted to immobilize the single-stranded DNA capture probes—leading to different mass transfer limitations; (2) competitive rebinding effects when multiple ligands are co-immobilized on a densely functionalized chip. Importantly, the “a” and “b” variants are structurally identical in their pharmacophore and linker regions and differ only in the DNA tag sequence, which is separated from the VHL ligand by an amino acid linker; the negative control (oligonucleotide without compound, “00”) showed no VHL binding (Fig. B8, SI), directly demonstrating that the DNA tag does not contribute to the interaction.

Beyond these chip-level factors, the two formats differ in their measurement variability. In the singleplex format, compounds were measured sequentially through independent cycles of regeneration, re-immobilization, and protein injection, introducing run-to-run variability that broadens the observed *K*_D_ distributions. The multiplexed format measures all compounds simultaneously within the same injection cycle, eliminating this inter-run variability and producing a compressed but internally more consistent *K*_D_ range. Regarding absolute accuracy, the singleplex format—with its lower surface density—likely provides values closer to solution-phase affinities, as its *K*_D_ range (20–160 nM) brackets published ITC and SPR values for VH032 (180–300 nM).^35,36^

Despite these systematic offsets in absolute *K*_D_ values, the strongest binders (compounds 3, 15, and 19)—all sharing high lipophilicity (*c *log *P* > +1)—were reliably identified in both formats, and both datasets independently reveal the same structure–activity relationships with lipophilicity (see below), supporting the validity of multiplexed measurements for comparative SAR studies.

### 2.4. Structure–activity relationships with physicochemical properties

To identify molecular descriptors predictive of binding affinity, we analyzed correlations between normalized *K*_D_ values and various physicochemical properties of the compounds (Table A47).

#### 2.4.1. Lipophilicity as a driver of binding affinity

**Fig. 6:**
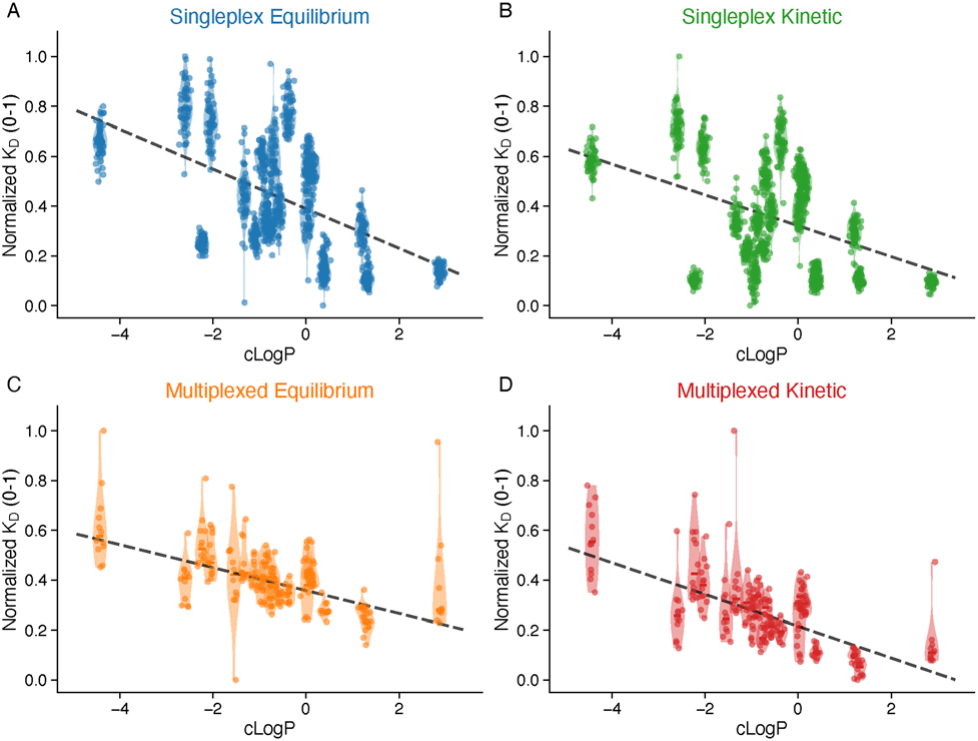
*K*_D_ correlation with lipophilicity. Violin plots showing normalized *K*_D_ (0–1 scale) *versus c *log *P* for (A) singleplex equilibrium, (B) singleplex kinetic, (C) multiplexed equilibrium, and (D) multiplexed kinetic methods. Each violin represents the distribution of *K*_D_ values for one compound positioned at its *c* log *P* value; individual measurements shown as points within violins. Dashed lines indicate linear regression of median values. All methods show significant negative correlation (*r* = −0.48 to −0.84, *p* < 0.05), indicating that higher lipophilicity is associated with stronger binding (lower *K*_D_).

The most striking correlation was observed between lipophilicity (*c* log *P*) and binding affinity (Fig. 6). All four analysis methods showed significant negative correlations, indicating that more lipophilic compounds exhibit stronger binding (lower *K*_D_) (Table 2):

**Table 2:** Correlation between *K*_D_ and lipophilicity (*c *log *P*). Spearman rank correlations (*ρ*) are reported alongside Pearson correlations (*r*) to provide a range-independent measure of association

Method	*r*	*ρ*	*p*-value	Sig.
Singleplex Eq.	−0.59	−0.52	0.008	**
Singleplex Kin.	−0.48	−0.39	0.039	*
Multiplex Eq.	−0.84	−0.78	4.0 × 10^−6^	****
Multiplex Kin.	−0.82	−0.73	1.1 × 10^−5^	****

The multiplexed format revealed substantially stronger correlations (*r* ≈ −0.83) compared to singleplex (*r* ≈ −0.53). To assess whether this difference could be an artifact of the compressed *K*_D_ range in the multiplexed format, we computed range-independent Spearman rank correlations: the multiplexed format retained stronger correlations (*ρ* = −0.78 and *ρ* = −0.73 for equilibrium and kinetic, respectively) compared to singleplex (*ρ* = −0.52 and *ρ* = −0.39). This confirms that the enhanced structure–activity signal in the multiplexed format is not solely attributable to range compression. We note that the key finding—that both formats independently identify the same trend direction (higher lipophilicity → lower *K*_D_ → stronger binding)—is the biologically meaningful conclusion for SAR purposes. This enhanced correlation likely reflects the more uniform experimental conditions achieved when all compounds are measured simultaneously on the same chip, as discussed in the cross-format comparison above.

#### 2.4.2. Additional physicochemical descriptors

Analysis of additional molecular descriptors (SI, Table B1 and Fig. B2–B7) revealed that aqueous solubility (*c *log *S*) showed strong positive correlation with *K*_D_ (*r* = 0.59 − 0.79), consistent with the inverse relationship between *c *log *S* and *c *log *P*. Relative polar surface area (PSA) showed moderate positive correlation (*r* = 0.49 − 0.61), indicating that compounds with higher polarity bind less favorably.

In contrast, the number of hydrogen bond donors, hydrogen bond acceptors, total surface area, and absolute polar surface area showed weak or no significant correlation with binding affinity. These findings suggest that overall hydrophobicity, rather than specific hydrogen bonding capacity or molecular size, is the primary driver of linker-VHL binding affinity differences in this compound series.

### 2.5. Implications for PROTAC design

Importantly, all tested linker structures were well-tolerated by VHL, providing flexibility to optimize the linker for overall drug-like properties (solubility, permeability, metabolic stability) rather than VHL binding affinity. While the consistent negative correlation between *c *log *P* and *K*_D_ indicates that hydrophobic interactions contribute to linker-VHL binding, this effect is modest and must be balanced against drug-like property considerations: highly lipophilic compounds often suffer from poor aqueous solubility, increased plasma protein binding, and unfavorable metabolic profiles.^37^ Based on our data, compounds in the *c *log *P* range of 0 to +2 offer an optimal balance between binding affinity and physicochemical tractability.

The observation that relative PSA (but not absolute PSA or total surface area) correlates with binding affinity suggests that the *fraction* of hydrophobic surface, rather than overall molecular size, determines binding strength. This insight may guide future linker design toward maintaining a low polar surface fraction while varying other structural features to optimize target engagement and cellular permeability.

### 2.6. Workflow advantages of DNA-directed immobilization for multiplexed one to many measurements

While conventional SPR imaging (SPRi) platforms offer high multiplexing capacity, the key differentiator of FM lies in the workflow efficiency enabled by DNA-directed immobilization (DDI). In conventional SPRi systems, each sensor spot must be individually functionalized with a different ligand—typically requiring complex microfluidic printheads, multiple spotting cycles, or contact printing procedures that can take hours to complete and may introduce spot-to-spot variability.^38^

In contrast, FM chips are pre-functionalized with an array of orthogonal oligonucleotide capture sequences. This enables a fundamentally different experimental workflow: pooled DNA-tagged compounds are simply injected onto the chip, and Watson–Crick base pairing automatically directs each compound to its designated sensing location within 5–10 minutes. This “one-pot” immobilization eliminates the need for sequential ligand spotting and dramatically reduces assay setup time. The approach is particularly advantageous for iterative compound screening, where new compound libraries can be rapidly loaded onto fresh chips without specialized printing equipment.

Furthermore, the DDI approach offers straightforward chip regeneration. The DNA duplex can be dissociated using mild denaturing conditions (3 M guanidinium chloride and 50 mM NaOH), allowing the oligonucleotide-functionalized chip to be stripped of bound compounds and reloaded with a different library. This regeneration capability, combined with the rapid compound loading, positions FM as an efficient platform for high-throughput affinity screening campaigns where both speed and flexibility are essential.

## 3. Conclusions

We have demonstrated the rapid synthesis of DNA–VHL ligand conjugates with diverse amino acid linkers by solid-phase Fmoc-peptide chemistry and their characterization by multiplexed FM. Key findings include:

1. Solid-phase amide coupling enables efficient synthesis of DNA–PROTAC libraries with diverse linker structures, with the VHL ligand remaining stable under cleavage conditions.

2. Equilibrium and kinetic fitting methods show strong correlation within each measurement format (*r* = 0.88 − 0.98), preserving compound rankings despite systematic offsets in absolute *K*_D_ values.

3. The multiplexed format achieves 20-fold higher throughput while yielding stronger SAR correlations, owing to the elimination of run-to-run variability inherent to sequential singleplex processing. The singleplex format provides absolute *K*_D_ values closer to solution-phase conditions, while the multiplexed format offers superior relative compound ranking.

4. Lipophilicity (*c *log *P*) is the primary predictor of linker-VHL binding affinity, with more hydrophobic compounds exhibiting stronger binding across all measurement conditions.

These findings establish multiplexed FM as a robust platform for PROTAC SAR studies and provide both a synthesis strategy and nucleic acid-based design principles for optimizing linker–E3 ligase interactions in targeted protein degradation programs.

Beyond our study on the effect of linker structures on the DNA–PROTAC affinity for VHL, the multiplexed affinity measurement method may be applied for the “on-DNA” validation of hits from DNA-encoded library screens. Furthermore, oligonucleotide-based affinity measurements using FM can address two additional therapeutic modalities: (i) targeting modules such as GalNAc conjugates used in approved antisense oligonucleotide drugs (*e.g.*, givosiran, inclisiran), where binding characterization is critical for optimizing tissue-specific delivery; and (ii) nucleic acid-based PROTACs, an emerging modality where oligonucleotide tags direct protein degradation but for which limited binding chemistry data are currently available.

## 4. Experimental

### 4.1. General

Chemicals with high purity (>95%) were purchased from Avantor VWR (Langenfeld, Germany), BLD Pharmatech (Shanghai, China), Fisher Scientific (Schwerte, Germany) and Sigma-Aldrich (Taufkirchen, Germany). All solvents used were at least analytical grade (>99%). Ultrapure lab water was obtained using a Merck Millipore Milli-Q Reference A+ system. Detailed methods for RP-HPLC purification, LC-MS analysis, and oligonucleotide concentration determination are provided in the SI.

### 4.2. Protein expression and purification

Human VHL (residues 54–213) was expressed together with EloB (residues 1–104) and EloC (residues 17–112) in *Escherichia coli* BL21(DE3) in LB medium at 18 °C overnight following induction with 0.5 M IPTG.^35^ Cell lysis was achieved by sonication in lysis buffer (20 mM Tris-HCl pH 8.0, 500 mM NaCl, 10 mM imidazole, 10% (v/v) glycerol). The VHL-EloB-EloC complex was purified by immobilized metal affinity chromatography (IMAC), followed by anion-exchange and size-exclusion chromatography (see SI for details).

### 4.3. Synthesis of DNA–PROTAC conjugates

DNA–PROTAC conjugates were synthesized by solid-phase amide coupling on CPG support.^34^ Briefly, the DMT-protective group was removed from the CPG-bound oligonucleotide (10 nmol) using 3% (v/v) dichloroacetic acid. Fmoc-protected amino acid linkers were coupled using HATU (100 equiv.) and DIPEA (250 equiv.) in dry DMF at 37 °C for 1 h. After Fmoc deprotection with 20% piperidine in DMF, the VHL ligand carboxylic acid was coupled under the same conditions. The conjugates were cleaved from the solid support using AMA solution (aqueous ammonia (30%)/aqueous methyl amine (40%), 1 : 1, v/v) for 4 h at room temperature and purified by semi-preparative RP-HPLC. Identity and purity were confirmed by LC-MS (see SI for detailed protocols and compound characterization).

### 4.4. Focal molography measurements

Binding affinities were determined using FM on a MACS Matchmaker instrument (Miltenyi Biotec, Germany). DNA–PROTAC compounds were immobilized on molographic sensor chips (lino Biotech AG, Switzerland) *via* their DNA tags, and VHL protein was flowed over the surface at concentrations ranging from 15.6 to 500 nM. Real-time binding responses were recorded at room temperature. Detailed experimental parameters including flow rates and injection volumes are provided in the SI Part B.

#### 4.4.1. Singleplex format

All singleplex measurements were performed on a single chip containing a 6 × 9 array of 54 sensing spots. Each mologram featured identical [cs01|PEG] architecture, where the cs01 oligonucleotide capture strand was immobilized on the diffractive ridges and the grooves were capped with a small PEG_2_ molecule (polyethylene glycol) to minimize non-specific binding. Compounds were measured sequentially, with each DNA–PROTAC compound hybridized to all 54 spots to provide 54 independent binding curves per compound. The “a” suffix linker variants (compounds 2a–20a) were used in this format.

#### 4.4.2. Multiplexed format

The 20-plex format utilized a single chip containing an 8 × 8 array of 64 sensing spots. Each mologram was functionalized with a distinct oligonucleotide capture strand ([cs01|PEG] through [cs20|PEG]), enabling simultaneous immobilization of 20 different DNA–PROTAC compounds. Each compound was represented by 3 replicate spots. Compound 1 and the “b” suffix linker variants (compounds 2b–20b) were used in this format.

### 4.5. Data analysis

#### 4.5.1. Equilibrium fitting

Steady-state response values were plotted against VHL concentration and fitted to a four-parameter logistic (4PL) model:1
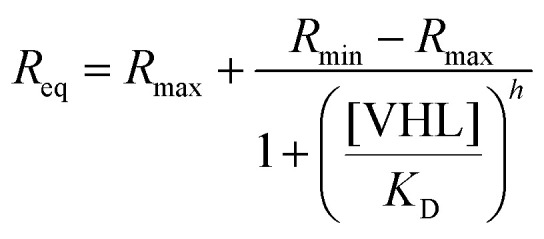
where *R*_min_ and *R*_max_ are the lower and upper asymptotic response values, *K*_D_ is the dissociation constant (concentration at the inflection point), and *h* is the Hill slope describing the steepness of the binding curve. For simple 1 : 1 binding, *h* = 1 and *R*_min_ = 0, which reduces to the standard hyperbolic binding isotherm.

#### 4.5.2. Kinetic fitting

Prior to fitting, the raw sensorgram was segmented into individual injection cycles. A blank cycle (buffer only) was subtracted from each cycle to correct for injection spikes due to flow rate changes. Additionally, the baseline offset accumulated from previous cycles was removed, such that each cycle started at zero response. Association and dissociation phases were then fitted to a 1 : 1 Langmuir binding model adapted to include a non-dissociating fraction (*f*_stable_). While *k*_a_ and *k*_d_ were fitted globally across all cycles, *f*_stable_ was treated as a local parameter, fitted independently for each injection cycle. The association phase follows standard Langmuir kinetics:

2 *R*(*t*) = *R*_eq_(1 − e^−(*k*_a_*C*+*k*_d_)*t*^)

where 
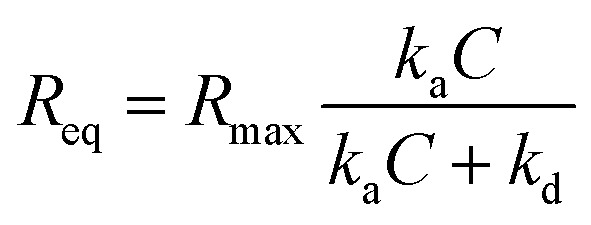
 is the equilibrium response at concentration *C*. The dissociation phase is modified to include a non-dissociating fraction:

3 *R*(*t*) = *R*_0_[(1 − *f*_stable_)e^−*k*_d_*t*^ + *f*_stable_]

where *R*_0_ is the response at the start of dissociation. This modification accounts for irreversible binding or very slow dissociation. The dissociation constant was calculated from the fitted rate constants:4
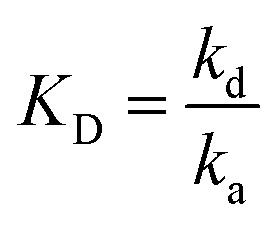


### 4.6. Physicochemical property calculation

Molecular descriptors including *c *log *P*, *c *log *S*, polar surface area (PSA), relative PSA, total surface area, and hydrogen bond donor/acceptor counts were calculated using ChemAxon software based on the SMILES representations of each compound.

### 4.7. Statistical analysis

Pearson correlation coefficients were calculated to assess relationships between *K*_D_ values and physicochemical properties. For cross-method comparisons, *K*_D_ values were normalized to a 0–1 scale within each method:5
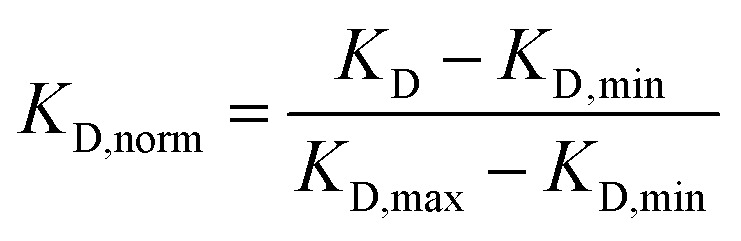


Linear regression was performed using SciPy (v1.11), and significance was assessed at *α* = 0.05. Statistical analyses and figure generation were performed using Python 3.11 with matplotlib.

## Author contributions

AB conceptualized the research project and wrote the manuscript. PR synthesized the “DNA–PROTAC”- compounds, made the figures and wrote the manuscript. AF performed the data analysis, made the figures and wrote the manuscript. SN performed the kinetic experiments. VG initiated the research project.

## Conflicts of interest

Andreas Frutiger, Volker Gatterdam and Simona Notova are employees of Lino Biotech AG and involved in the commercialization of focal molography.

## Supplementary Material

CB-007-D6CB00011H-s001

## Data Availability

The data supporting this article have been included as part of the supplementary information (SI). Supplementary information is available. See DOI: https://doi.org/10.1039/d6cb00011h.
