# Supplementary material for: Investigations into linker effects of DNA–VHL ligand conjugates by multiplexed affinity measurements using focal molography
Source: RSC Chem Biol. 2026 Mar 20;7(5):870–9. doi: 10.1039/d6cb00011h (PMC13014410; doi:10.1039/d6cb00011h)
Supplement: CB-007-D6CB00011H-s001 [file CB-007-D6CB00011H-s001.pdf]

Supplementary Information (SI) for RSC Chemical Biology.
This journal is © The Royal Society of Chemistry 2026

Supporting Information:

Investigations into Linker Effects of DNA-VHL Ligand Conjugates by Multiplexed Affinity Measurements Using Focal Molography

Pascal Raschke,[a] Simona Notova,[b] Volker Gatterdam,[b] Andreas Frutiger,[b] and Andreas Brunschweiger[a,*]

[a] *Institute of Pharmacy and Food Chemistry, Julius-Maximilians-Universität Würzburg, Am Hubland, 97074 Würzburg, Germany*
[b] *lino Biotech AG, Soodstrasse 52, 8134 Adliswil, Switzerland*
[*] *Corresponding author. E-mail: andreas.brunschweiger@uni-wuerzburg.de*

Contents

Part A: General Methods and Synthesis

- General Methods (RP-HPLC, LC-MS, UV spectroscopy)
- **Tables SI-A1–SI-A2**: HPLC and LC-MS gradient parameters
- Protein Expression and Purification
- **Figures SI-A1–SI-A3**: SDS-PAGE and SEC chromatograms
- Solid-Phase Synthesis Protocols
- **Figures SI-A4–SI-A7**: Reaction schemes
- Compound Overview and Characterization
- **Tables SI-A3–SI-A4**: Library 1 and Library 2 compound overview
- **Figures SI-A8–SI-A46**: LC-MS characterization of all compounds

Part B: Binding Data Analysis

- Focal Molography Measurements (methodology)
- **Figure SI-B1**: Sensor chip layout for multiplexed measurements
- **Table SI-B1**: Summary of correlations between K_D and physicochemical properties
- **Figures SI-B2–SI-B7**: K_D correlations with physicochemical properties
- **Figure SI-B8**: Negative control sensorgrams
- **Figures SI-B9–SI-B13**: Raw sensorgrams
- **Figures SI-B14–SI-B23**: Equilibrium and kinetic binding fits (compounds 2–20)
- **Figure SI-B24**: BSA negative control screen
- **Tables SI-B2–SI-B20**: Detailed kinetic parameters by compound

Part A: General Methods and Synthesis

General Methods

Reagents. Chemicals with a high purity (>95%) were purchased from Avantor VWR (Langenfeld, Germany), BLD Pharmatech (Shanghai, China), Fisher Scientific (Schwerte, Germany) and Sigma-Aldrich (Taufkirchen, Germany). All solvents used were at least analytical grade (>99%). Ultrapure lab water quality was achieved by usage of Merck Millipore Milli-Q® Reference A+ ultrapure lab water system (Darmstadt, Germany). Plasmids were purchased from Addgene (Watertown, United States). DNA oligonucleotides with 5'-aminolinker modification bound to controlled pore glass solid support (CPG, 1000 Å porosity) were synthesized by EllaBiotech (Fürstenfeldbruck, Germany). Reactors for solid-phase synthesis were purchased from Carl-Roth (Karlsruhe, Germany).

Semi-preparative RP-HPLC method. Oligonucleotide-small molecule conjugates were purified by RP-HPLC Agilent 1260 Infinity II (Santa Clara, United States) with fraction collector using a C18 stationary phase (Phenomenex (Torrance, United States), Gemini; 5 µm, C18, 110 Å, 100×10 mm). A gradient from 100 mM aqueous triethylammonium acetate buffer (pH = 8.0, eluent A) to MeOH (eluent B) was used at a flow rate of 3 mL/min (**Table SI-A1**). UV absorption chromatogram was recorded at 260 and 280 nm wavelengths.

Table SI-A1. Linear gradient parameters of semi-preparative RP-HPLC method.

t (min)	Eluent A (%)	Eluent B (%)
0–1	80	20
1→9	20	80
9→9.30	0	100
9.30–11	0	100
11→14	80	20
14–15	80	20

LC-MS method. Oligonucleotide-small molecule conjugate identity and purity was analyzed by LC-MS Agilent 1260 Infinity II (Santa Clara, United States) using a C18 stationary phase (Phenomenex (Torrance, United States), Gemini; 3 µm, C18, 110 Å, 100×2 mm). A gradient from an aqueous mixture of 100 mM hexafluoroisopropanol (HFIP) and 15 mM triethylammonium (eluent A) and MeOH (eluent B) was used at a flow rate of 0.5 mL/min at 55 °C (**Table SI-A2**). UV absorption chromatogram was recorded at 260 nm wavelength. MS spectra (ESI negative mode) were simultaneously obtained. OpenLab CDS MS Spectral Deconvolution software was used to automatically calculate compound mass.

Table SI-A2. Linear gradient parameters of LC-MS method.

t (min)	Eluent A (%)	Eluent B (%)
0	90	10
0→3	50	50
3→9	5	95
9–10	5	95
10→15	90	10

Determination of oligonucleotide concentrations. Oligonucleotide concentrations were determined by UV spectroscopy using a spectrophotometer Implen NanoPhotometer N120 (München, Germany).

Protein Expression and Purification

Human VM02-VHL encoding amino acids 54–213 and human VM26-EloB (amino acids 1–104) / EloC (amino acids 17–112) were expressed in *Escherichia coli* BL21 (DE3) in LB medium at 18 °C overnight following induction with 0.5 M IPTG. Cell lysis was achieved by sonification of a cell pellet in 180 mL of lysis buffer (20 mM Tris-HCl (pH 8.0), 500 mM NaCl, 10 mM imidazole, 10 mM $MgCl_2$). Expressed proteins were purified by Ni-NTA column chromatography in the same buffer and eluted with IMAC elution buffer (20 mM Tris-HCl pH 8.0, 500 mM NaCl, 300 mM imidazole). Elution fractions were analysed by SDS-PAGE analysis.

With proof of desired protein expression IMAC eluates were pooled and dialyzed overnight at 6–8 °C against 2 L dialysis buffer (20 mM Bis-Tris pH 7.0, 150 mM NaCl, 1 mM DTT) and the by centrifugation cleared dialysate was loaded to MonoQ 10/100 anion-exchange chromatography column, pre-equilibrated with IEX-A buffer (20 mM Bis-Tris pH 7.0, 150 mM NaCl, 1 mM DTT). The column was washed with the same buffer and elution was performed by a gradient to 100% of IEX-B buffer (20 mM Bis-Tris pH 7.0, 1 M NaCl, 1 mM DTT). IEX chromatogram and SDS-PAGE indicated the protein complex did not bind properly to the column, therefore size exclusion chromatography (SEC) with a Superdex 75 16/600 column was performed with the washing fraction from the anion-exchange chromatography. A clean peak eluted in SEC, verifying successful complex formation.

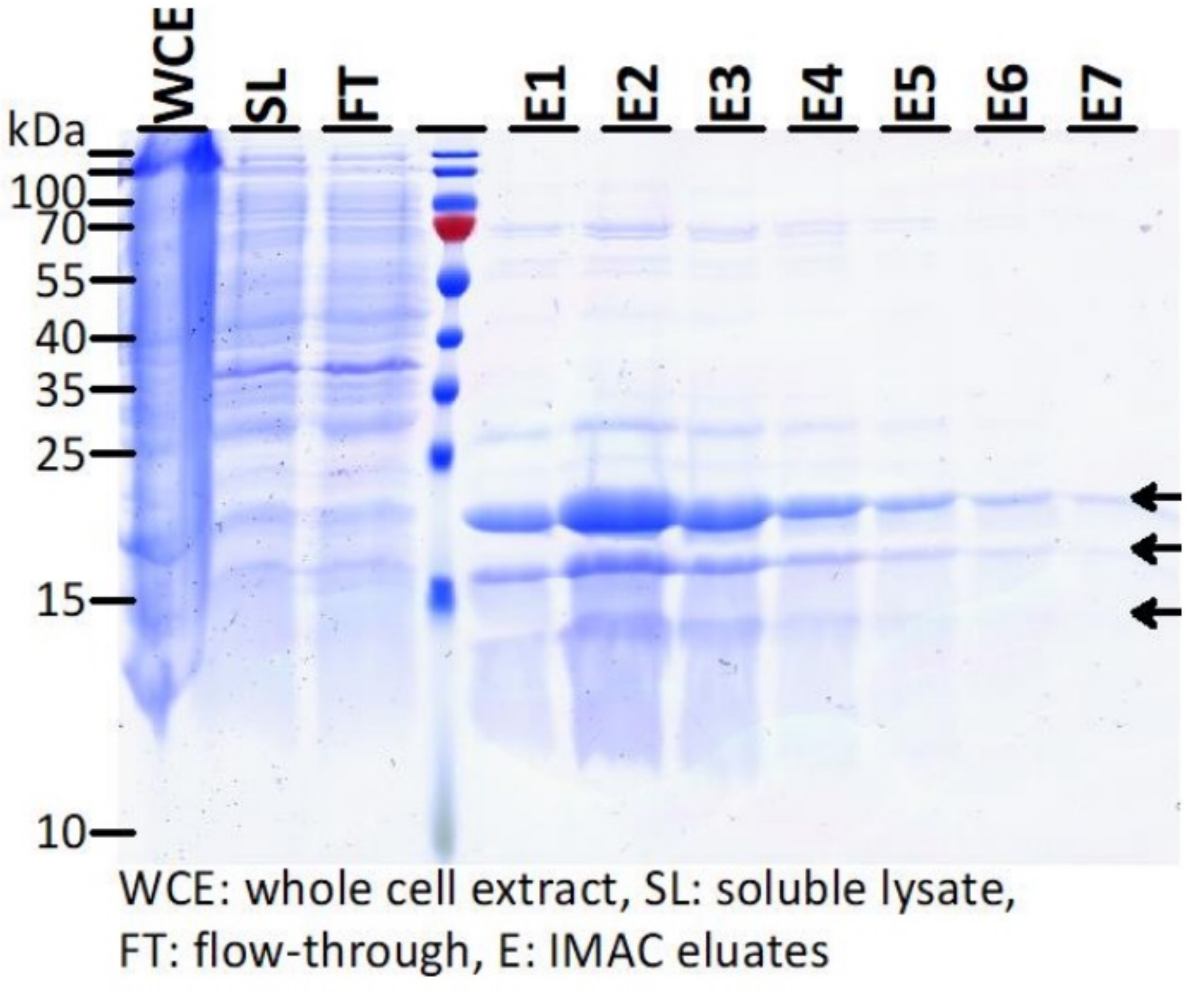

Figure SI-A1. SDS-PAGE of IMAC elution fractions E1–E7. WCE: whole cell extract. SL: soluble lysate. FT: flow-through, E: IMAC eluates. Arrows pointing on desired protein bands.

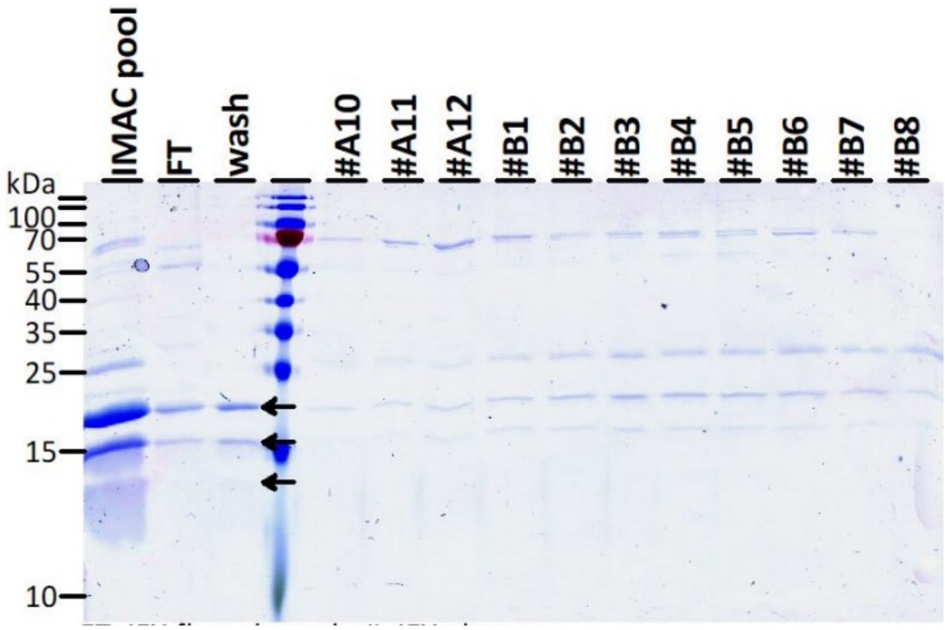

Figure SI-A2. SDS-PAGE after anion-exchange chromatography indicating no protein complex formation.

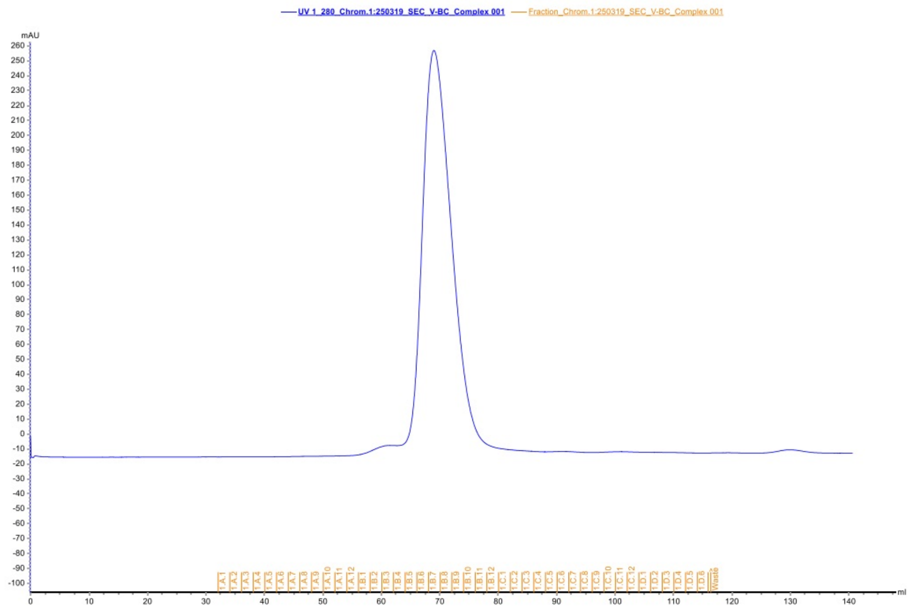

Figure SI-A3. Chromatogram from size exclusion chromatography with simultaneous co-elution of VHL, EloB and EloC.

Solid-Phase Synthesis Protocols

Solid-phase amide coupling – linker attachment. The CPG-bound oligonucleotide (10 nmol) was transferred into a filter syringe reactor. The DMT-protective group was removed by incubation with 200 µL 3% (v/v) dichloroacetic acid in for 30 seconds. The deprotection step was repeated until the yellow colour fully disappeared. The oligonucleotide was then treated with 200 µL 1% (v/v) triethylamine for 60 seconds and washed three times each with 200 µL of DMF, MeOH, ACN and DCM and finally dried in vacuo. Stock solutions of fmoc-protected amino acids, the coupling agent HATU were prepared in dry DMF and a pre-activation mixture of fmoc-protected amino acid (40 µL, 100 equiv., 1 µmol), DIPEA (0.44 µL, 250 equiv., 2.5 µmol) and HATU (40 µL, 100 equiv., 1 µmol) was shaken for 10 minutes at room temperature. The mixture was then added to the CPG-bound oligonucleotide suspended in 80 µL of dry DMF. After 1 hour of shaking at 37 °C the oligo was washed three times each with 200 µL of DMF, MeOH, ACN and DCM and finally dried in vacuo. The amide coupling step was repeated twice.

Fmoc deprotection. The fmoc-protecting group was removed from the oligonucleotide-linker conjugate by the addition of 200 µL 20% piperidine in DMF at 37 °C for 10 minutes. The oligonucleotide was then treated with 200 µL 1% (v/v) triethylamine for 60 seconds and washed three times each with 200 µL of DMF, MeOH, ACN and DCM and finally dried in vacuo. The procedure was repeated twice.

Solid-phase amide coupling – VHL ligand attachment. Stock solutions of the VHL ligand carboxylic acid, the coupling agent HATU were prepared in dry DMF and a pre-activation mixture of the VHL ligand carboxylic acid (40 µL, 100 equiv., 1 µmol), DIPEA (0.44 µL, 250 equiv., 2.5 µmol) and HATU (40 µL, 100 equiv., 1 µmol) was shaken for 10 minutes at room temperature. The mixture was then added to the CPG-bound oligonucleotide previously suspended in 80 µL of dry DMF. After 1 hour of shaking at 37 °C the oligo was washed three times each with 200 µL of DMF, MeOH, ACN and DCM and finally dried in vacuo. The amide coupling step was repeated twice.

Cleavage from CPG-solid support. The CPG-bound oligonucleotide conjugate was incubated with 250 µL of AMA solution (AMA = aqueous ammonia (30%) / aqueous methylamine (40%), 1:1, v/v) for 4 hours at room temperature. The cleaved compounds were collected in an Eppendorf tube, the filter syringe was washed with 100 µL of water and ACN twice. The mixture was dried in a vacuum concentrator and dissolved in 100 µL water. The crude was purified by semi-preparative RP-HPLC and analysed by LC-MS as previously described.

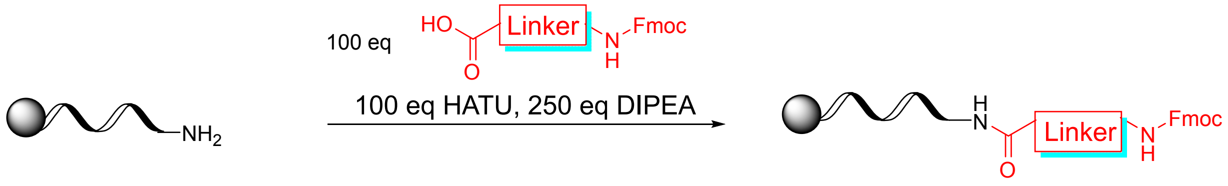

Figure SI-A4. Attachment of the fmoc-protected amino acids to the CPG-bound oligonucleotides by solid-phase amide coupling reaction.

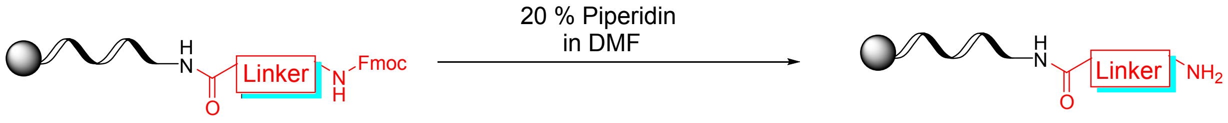

Figure SI-A5. Removal of the fmoc-protecting group from the CPG-bound oligonucleotide linker conjugate.

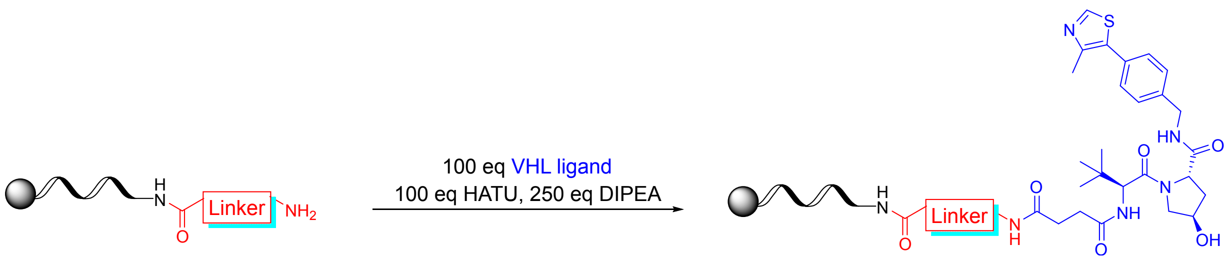

Figure SI-A6. Attachment of the VHL ligand to the CPG-bound oligonucleotide linker conjugate by solid-phase amide coupling reaction.

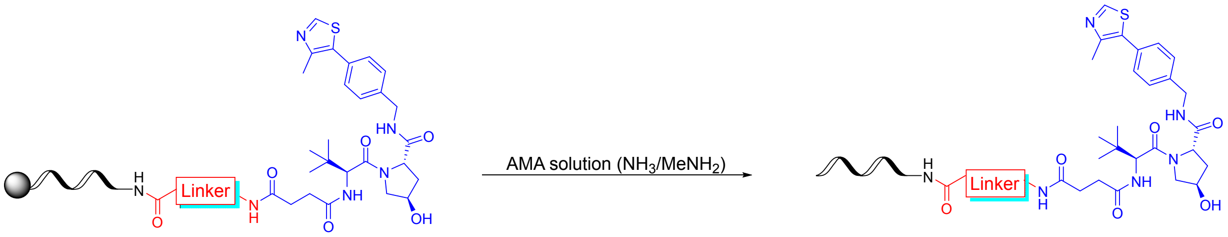

Figure SI-A7. Cleavage of the CPG-bound oligonucleotide linker VHL ligand conjugate into solution phase.

Compound Overview

Table SI-A3. Overview of Library 1 compounds and attached oligo number (oligonucleotide sequences are pseudonymized as they are intellectual property of lino Biotech AG, Adliswil, Switzerland).

Compound name	Attached oligo
Compound 1	Oligo 01
Compound 2a	Oligo 01
Compound 3a	Oligo 01
Compound 4a	Oligo 01
Compound 5a	Oligo 01
Compound 6a	Oligo 01
Compound 7a	Oligo 01
Compound 8a	Oligo 01
Compound 9a	Oligo 01
Compound 10a	Oligo 01
Compound 11a	Oligo 01
Compound 12a	Oligo 01
Compound 13a	Oligo 01
Compound 14a	Oligo 01
Compound 15a	Oligo 01
Compound 16a	Oligo 01
Compound 17a	Oligo 01
Compound 18a	Oligo 01
Compound 19a	Oligo 01
Compound 20a	Oligo 01

Table SI-A4. Overview of Library 2 compounds and attached oligo number (oligonucleotide sequences are pseudonymized as they are intellectual property of lino Biotech AG, Adliswil, Switzerland).

Compound name	Attached oligo
Compound 1	Oligo 01
Compound 2b	Oligo 02
Compound 3b	Oligo 03
Compound 4b	Oligo 04
Compound 5b	Oligo 05
Compound 6b	Oligo 06
Compound 7b	Oligo 07
Compound 8b	Oligo 08
Compound 9b	Oligo 19
Compound 10b	Oligo 16
Compound 11b	Oligo 20
Compound 12b	Oligo 09
Compound 13b	Oligo 11
Compound 14b	Oligo 10
Compound 15b	Oligo 18
Compound 16b	Oligo 17
Compound 17b	Oligo 13
Compound 18b	Oligo 14
Compound 19b	Oligo 15
Compound 20b	Oligo 12

Compound Characterization – Library 1

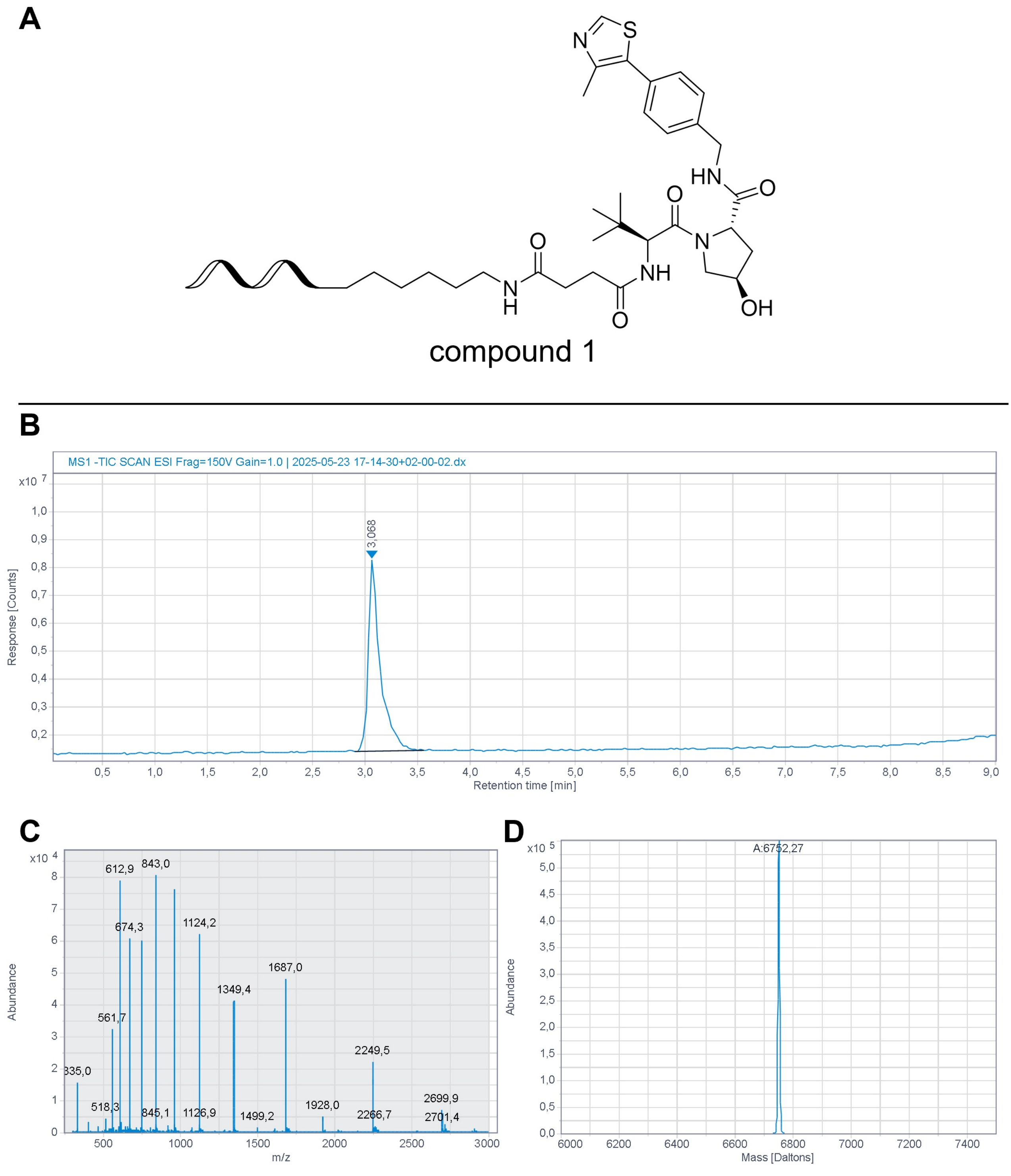

Figure SI-A8. (A) Structure of Compound 1. (B) LCMS chromatogram of purified compound 1. (C) ESI(-)-MS spectrum of purified compound 1. (D) Deconvoluted mass of purified compound 1. Mass expected: 6751.64. Mass found: 6752.27.

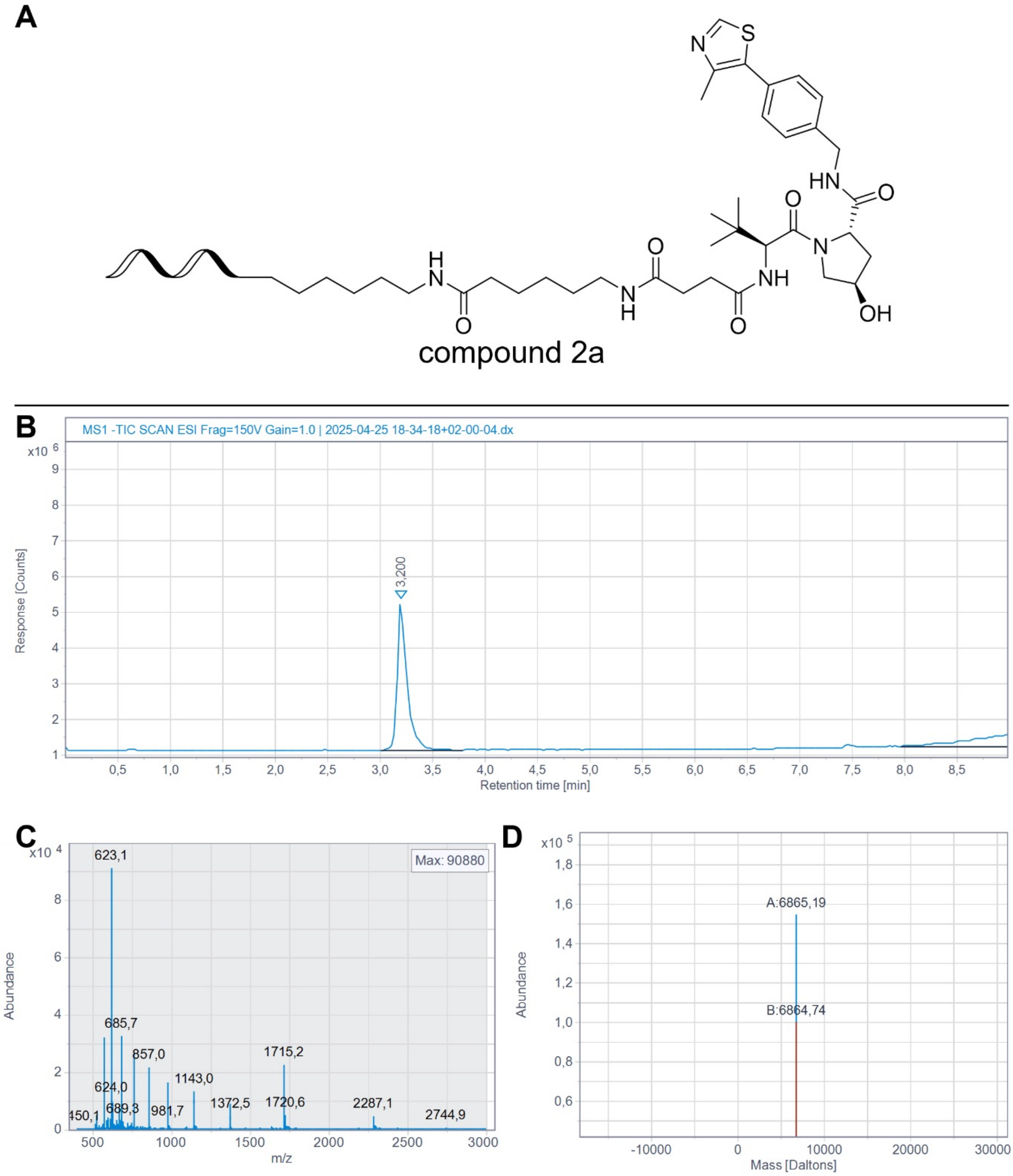

Figure SI-A9. (A) Structure of Compound 2a. (B) LCMS chromatogram of purified compound 2a. (C) ESI(-)-MS spectrum of purified compound 2a. (D) Deconvoluted mass of purified compound 2a. Mass expected: 6865.04. Mass found: 6865.19.

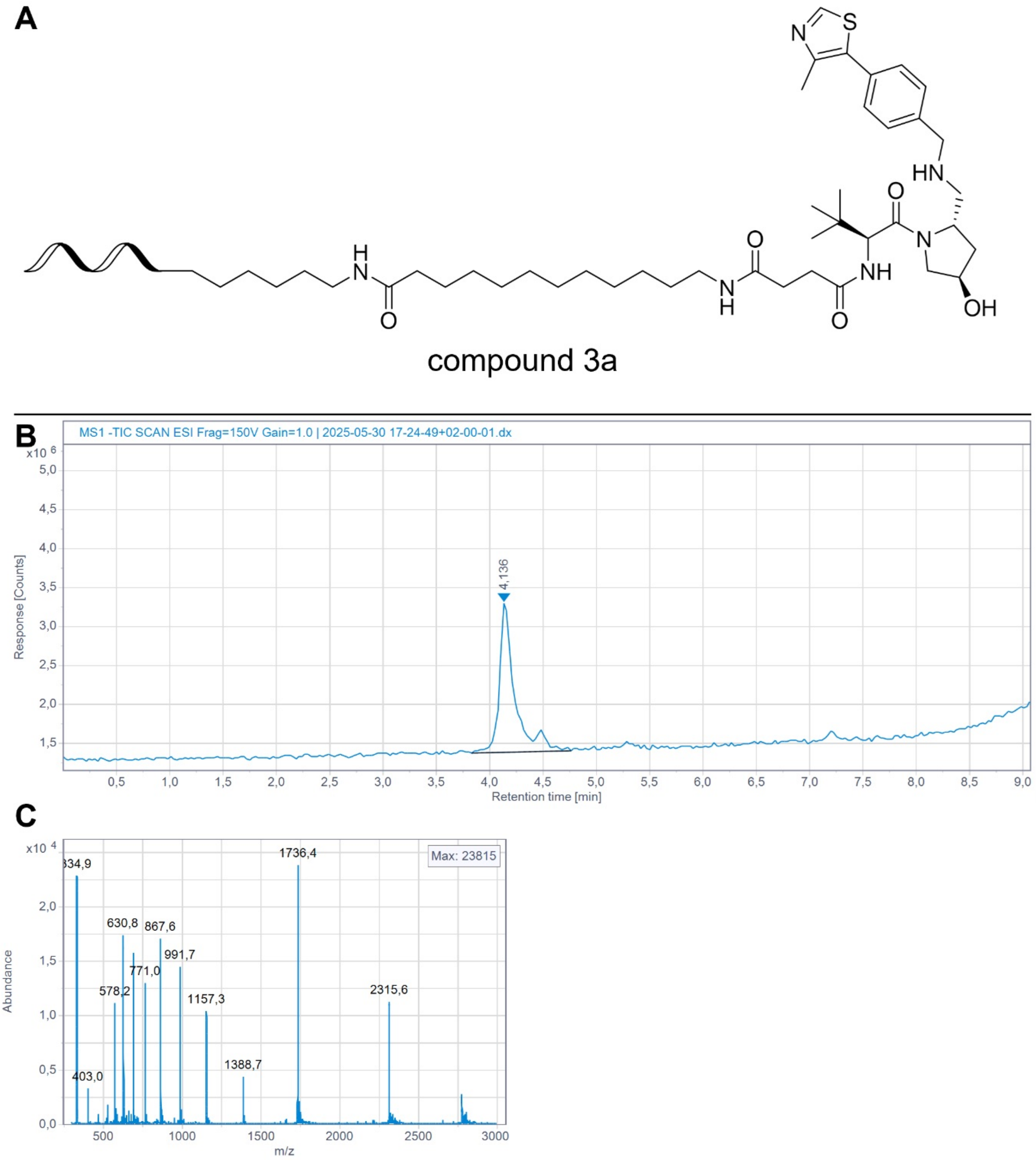

Figure SI-A10. (A) Structure of Compound 3a. (B) LCMS chromatogram of purified compound 3a. (C) ESI(-)-MS spectrum of purified compound 3a. Automated deconvolution failed. Mass expected: 6949.24. Mass found: 6949.4 (mean of manual deconvolution with ion sets: [[(630.8 x11) +11] + [(867.6 x8) +8] + [(1736.4 x4) +4]]).

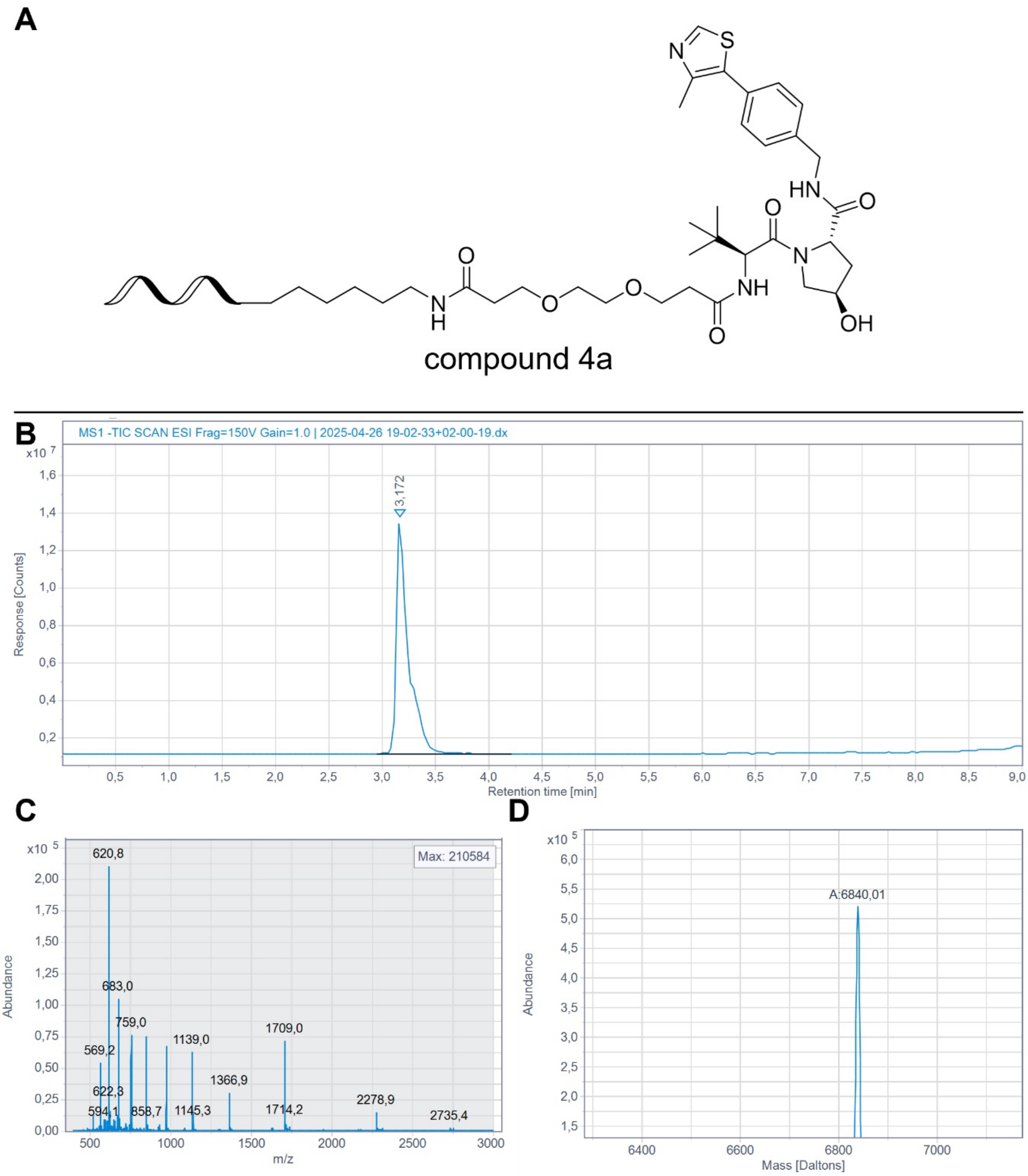

Figure SI-A11. (A) Structure of Compound 4a. (B) LCMS chromatogram of purified compound 4a. (C) ESI(-)-MS spectrum of purified compound 4a. (D) Deconvoluted mass of purified compound 4a. Mass expected: 6839.70. Mass found: 6840.01.

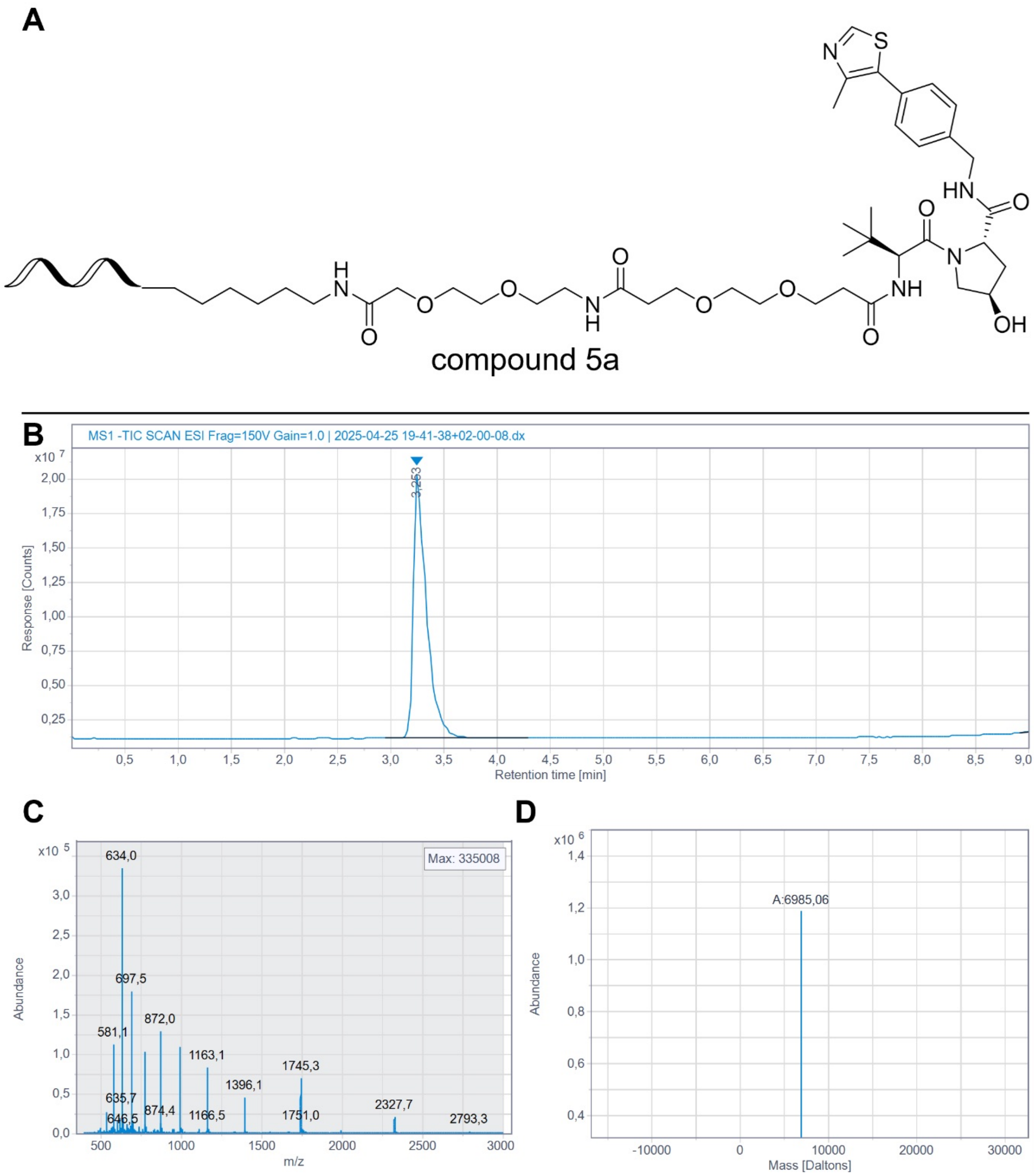

Figure SI-A12. (A) Structure of Compound 5a. (B) LCMS chromatogram of purified compound 5a. (C) ESI(-)-MS spectrum of purified compound 5a. (D) Deconvoluted mass of purified compound 5a. Mass expected: 6985.10. Mass found: 6985.06.

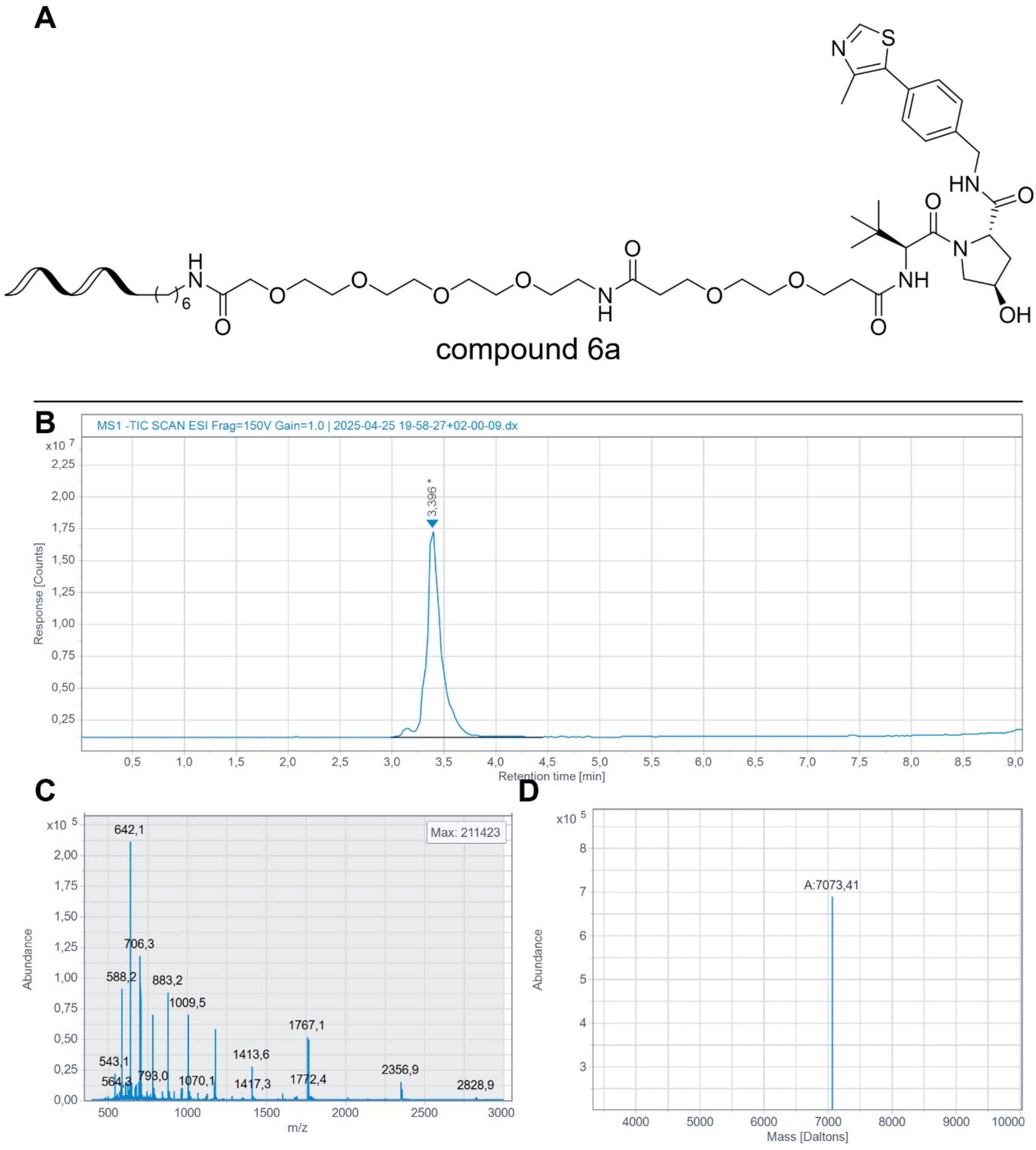

Figure SI-A13. (A) Structure of Compound 6a. (B) LCMS chromatogram of purified compound 6a. (C) ESI(-)-MS spectrum of purified compound 6a. (D) Deconvoluted mass of purified compound 6a. Mass expected: 7073.20. Mass found: 7073.41.

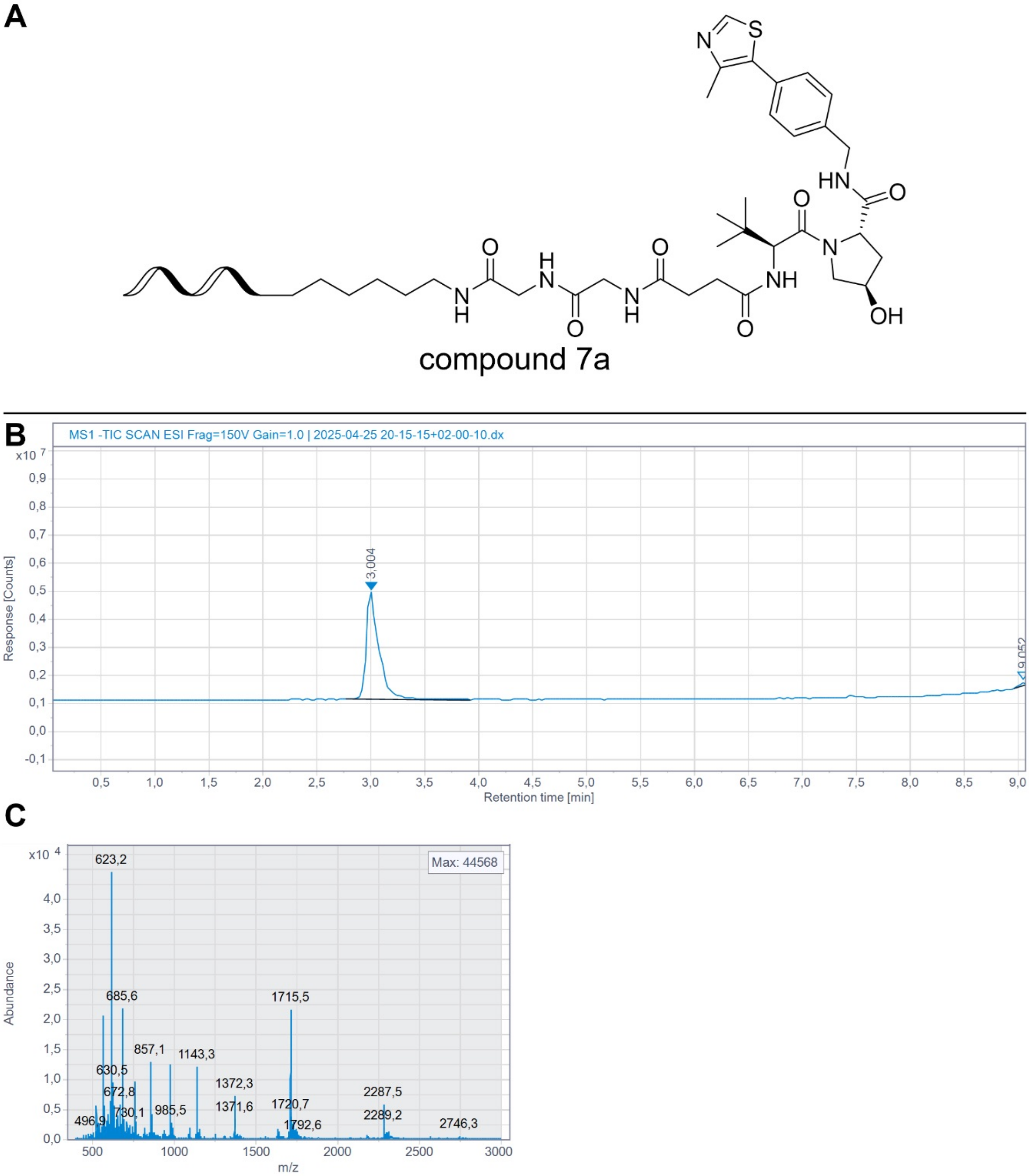

Figure SI-A14. (A) Structure of Compound 7a. (B) LCMS chromatogram of purified compound 7a. (C) ESI(-)-MS spectrum of purified compound 7a. Automated deconvolution failed. Mass expected: 6866.00. Mass found: 6866.07 (mean of manual deconvolution with ion sets: [[(623.2 x11) +11] + [(685.6 x10) +10] + [(1715.5 x4) +4]]).

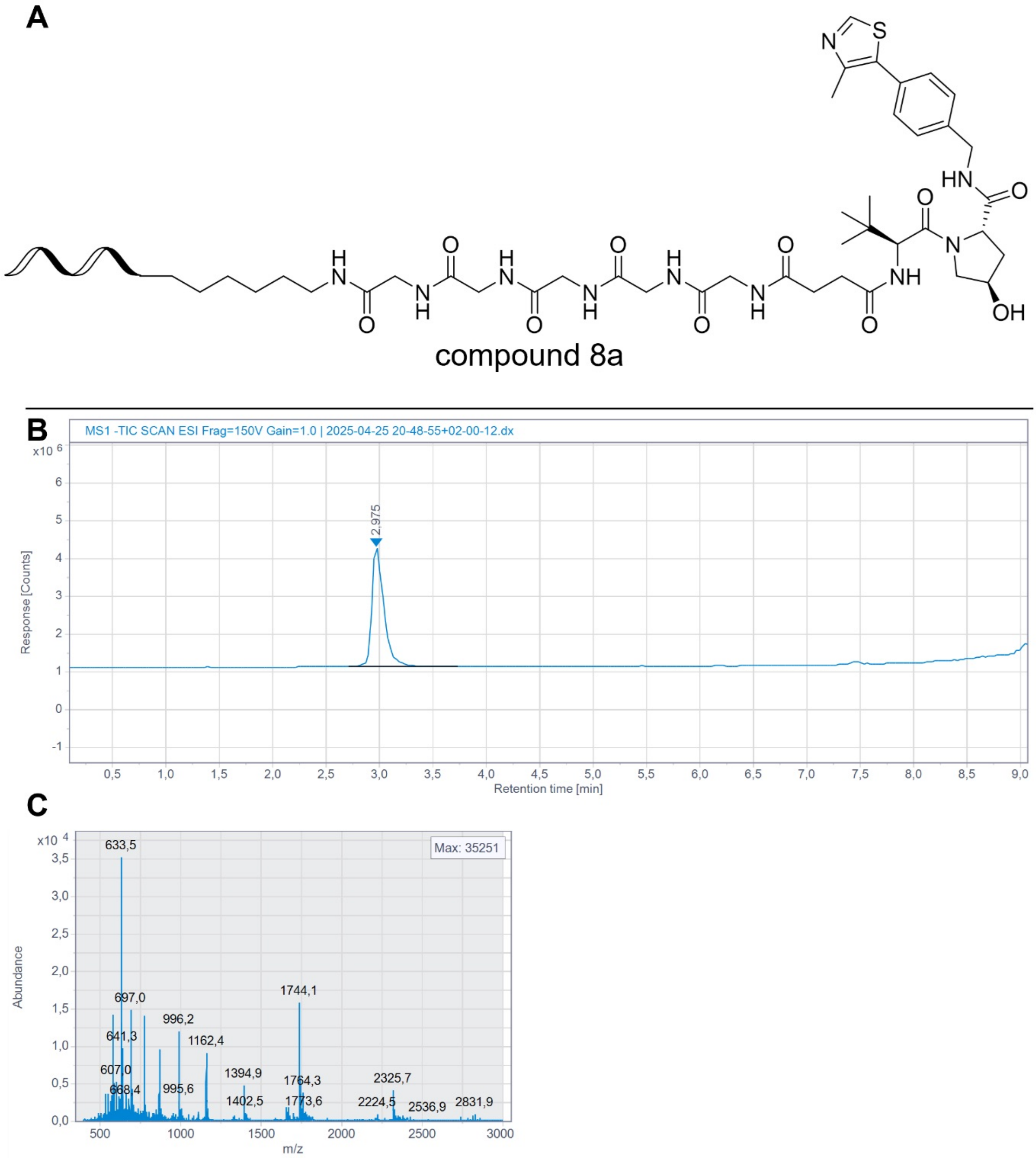

Figure SI-A15. (A) Structure of Compound 8a. (B) LCMS chromatogram of purified compound 8a. (C) ESI(-)-MS spectrum of purified compound 8a. Automated deconvolution failed. Mass expected: 6980.10. Mass found: 6980.0 (mean of manual deconvolution with ion sets: [[(633.5 x11) +11] + [(697.0 x10) +10] + [(1744.1 x4) +4]]).

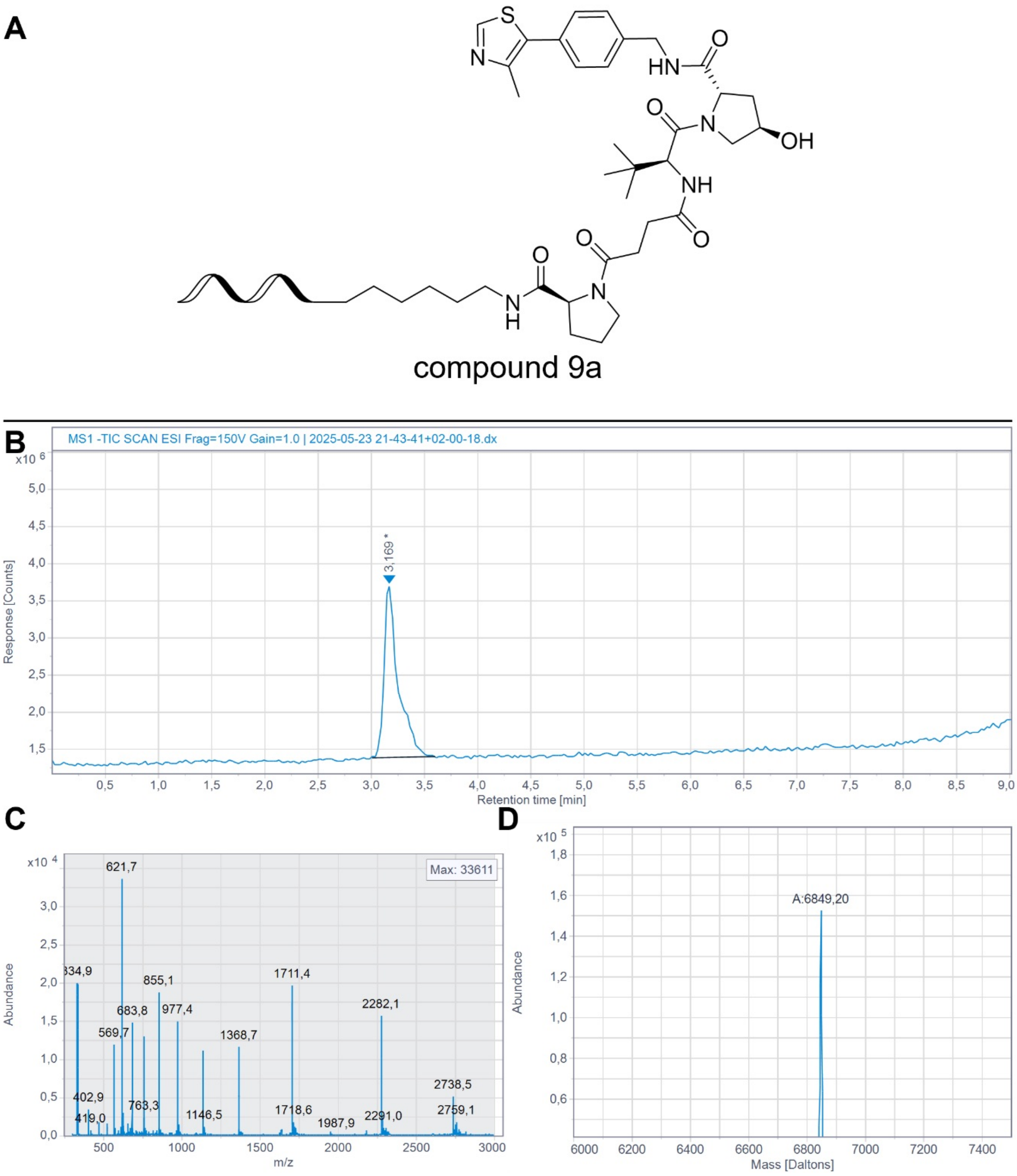

Figure SI-A16. (A) Structure of Compound 9a. (B) LCMS chromatogram of purified compound 9a. (C) ESI(-)-MS spectrum of purified compound 9a. (D) Deconvoluted mass of purified compound 9a. Mass expected: 6848.64. Mass found: 6849.20.

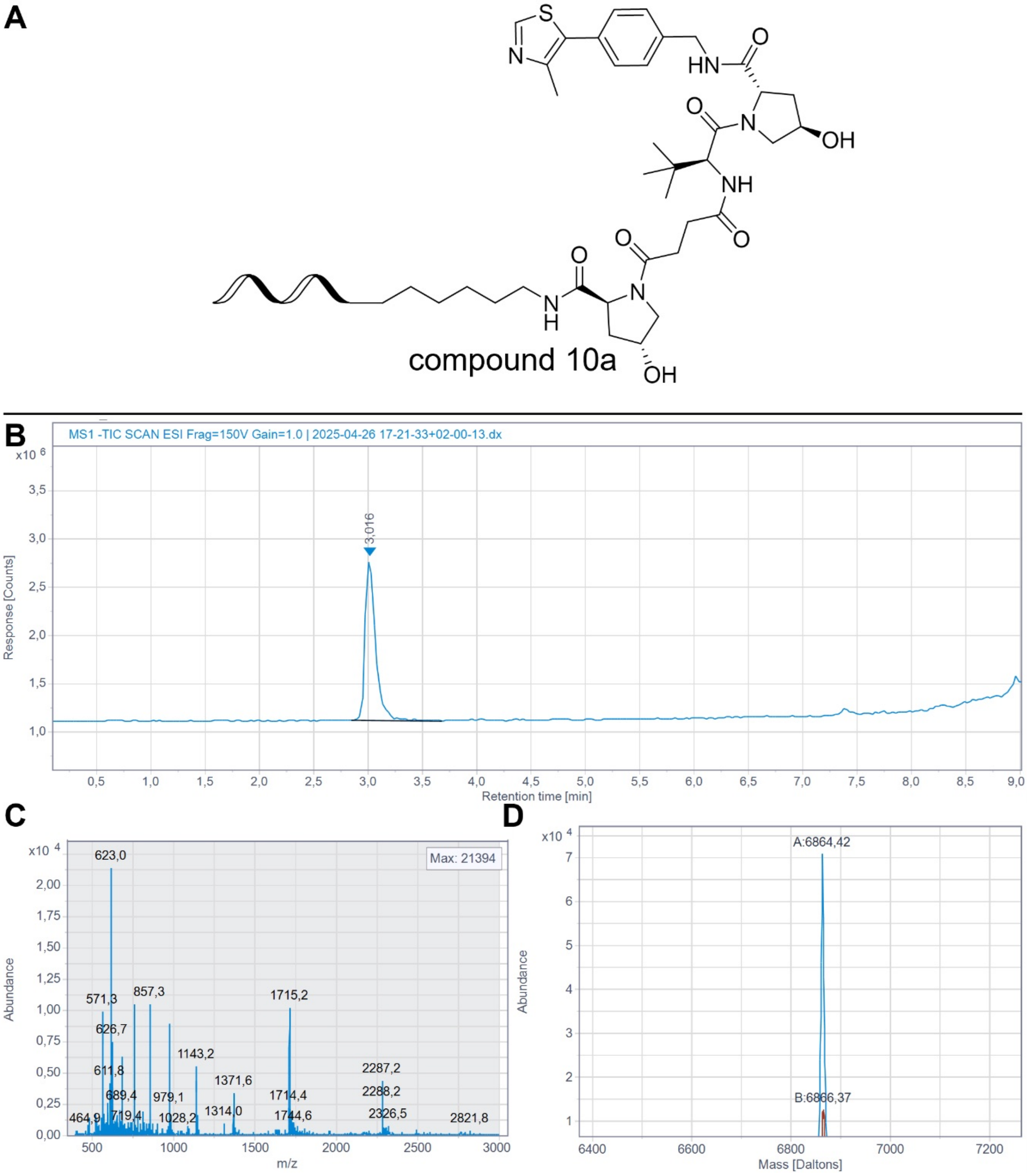

Figure SI-A17. (A) Structure of Compound 10a. (B) LCMS chromatogram of purified compound 10a. (C) ESI(-)-MS spectrum of purified compound 10a. (D) Deconvoluted mass of purified compound 10a. Mass expected: 6865.01. Mass found: 6864.42.

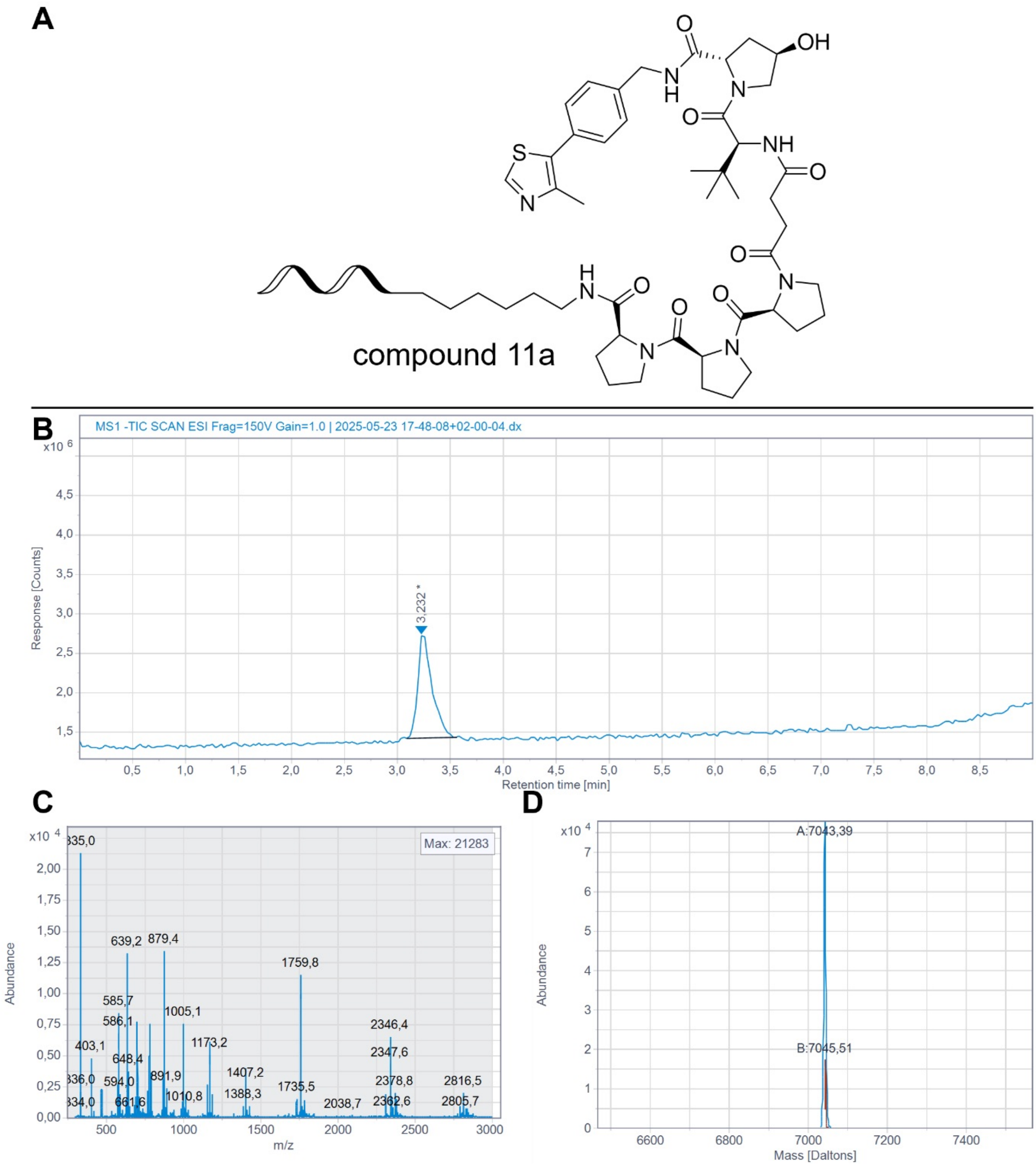

Figure SI-A18. (A) Structure of Compound 11a. (B) LCMS chromatogram of purified compound 11a. (C) ESI(-)-MS spectrum of purified compound 11a. (D) Deconvoluted mass of purified compound 11a. Mass expected: 7043.75. Mass found: 7043.39.

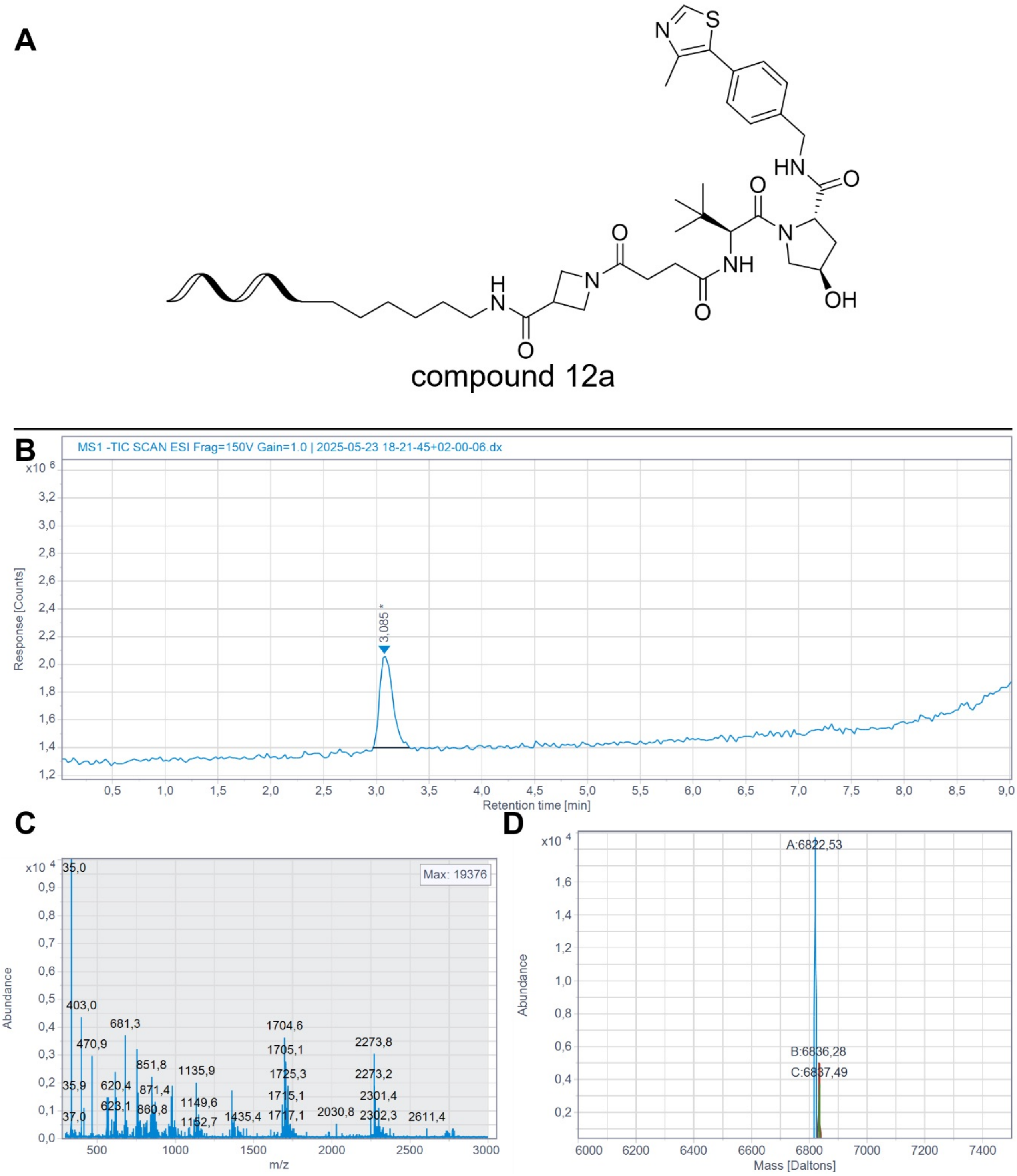

Figure SI-A19. (A) Structure of Compound 12a. (B) LCMS chromatogram of purified compound 12a. (C) ESI(-)-MS spectrum of purified compound 12a. (D) Deconvoluted mass of purified compound 12a. Mass expected: 6834.99. Mass found: 6822.53. We hypothesize in-source fragmentation with carbon-loss due to ESI as residues of unfragmented compound were also observed (M_r 6836.28).

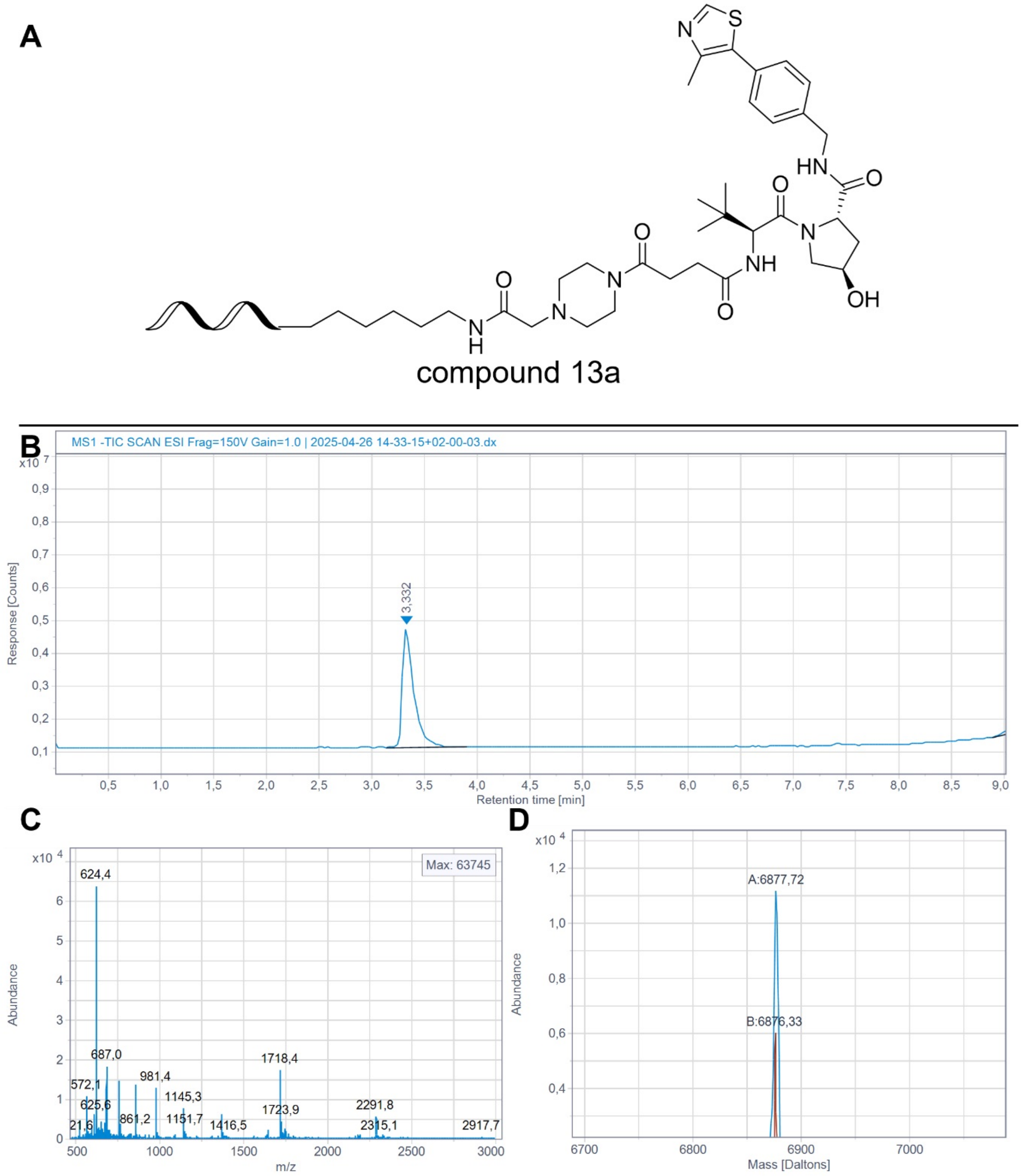

Figure SI-A20. (A) Structure of Compound 13a. (B) LCMS chromatogram of purified compound 13a. (C) ESI(-)-MS spectrum of purified compound 13a. (D) Deconvoluted mass of purified compound 13a. Mass expected: 6878.04. Mass found: 6877.72.

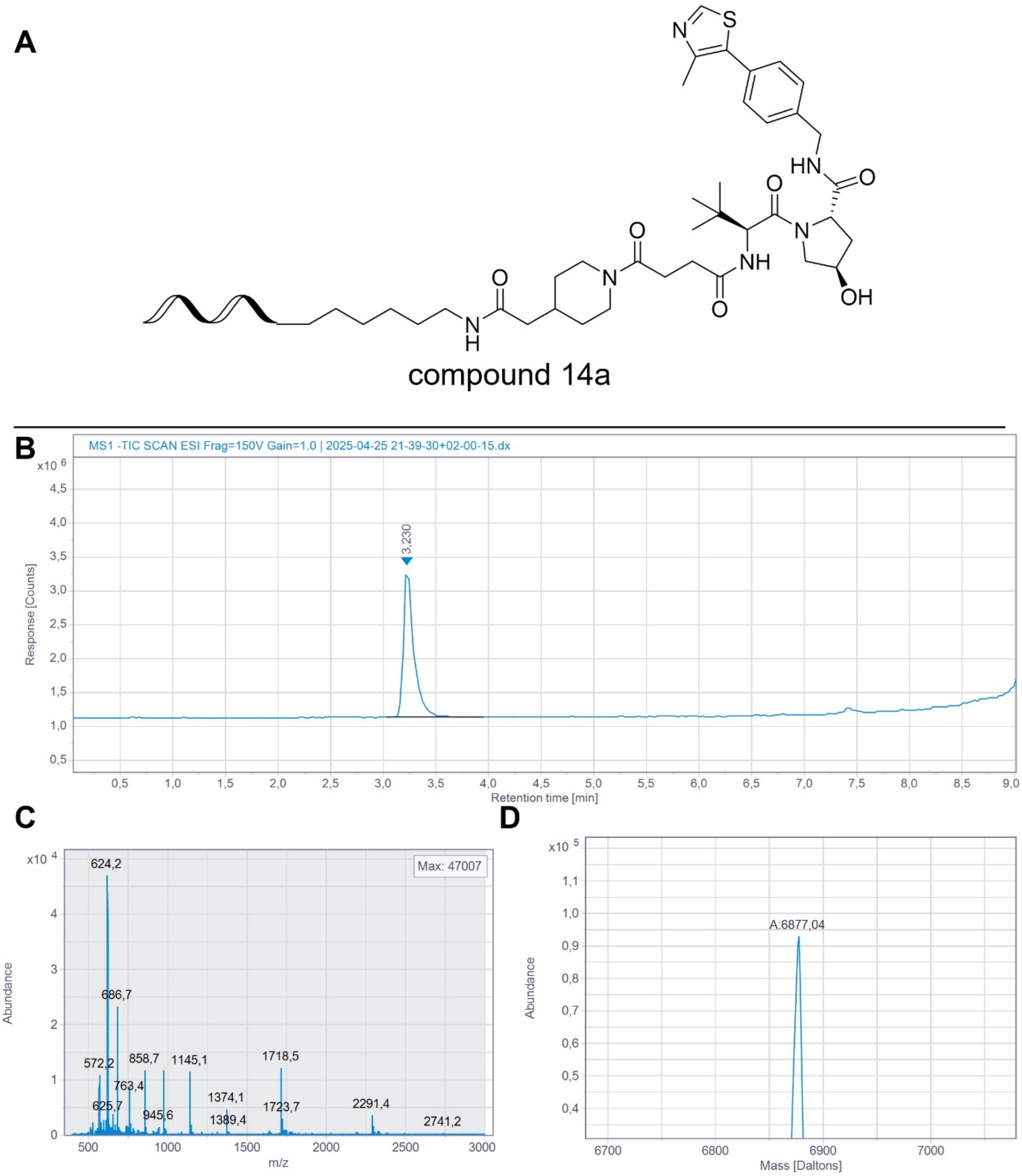

Figure SI-A21. (A) Structure of Compound 14a. (B) LCMS chromatogram of purified compound 14a. (C) ESI(-)-MS spectrum of purified compound 14a. (D) Deconvoluted mass of purified compound 14a. Mass expected: 6877.04. Mass found: 6877.04.

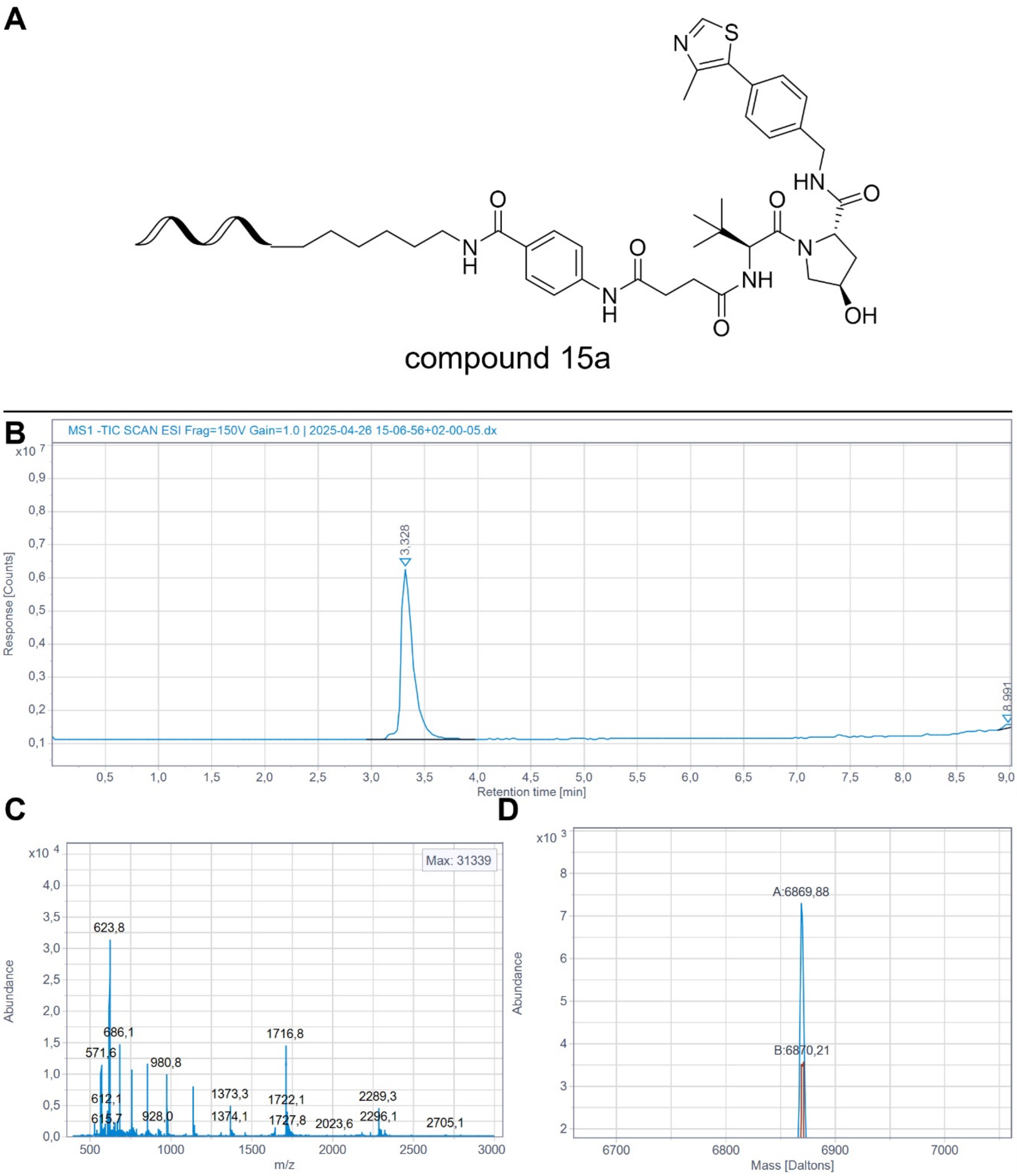

Figure SI-A22. (A) Structure of Compound 15a. (B) LCMS chromatogram of purified compound 15a. (C) ESI(-)-MS spectrum of purified compound 15a. (D) Deconvoluted mass of purified compound 15a. Mass expected: 6871.04. Mass found: 6869.88.

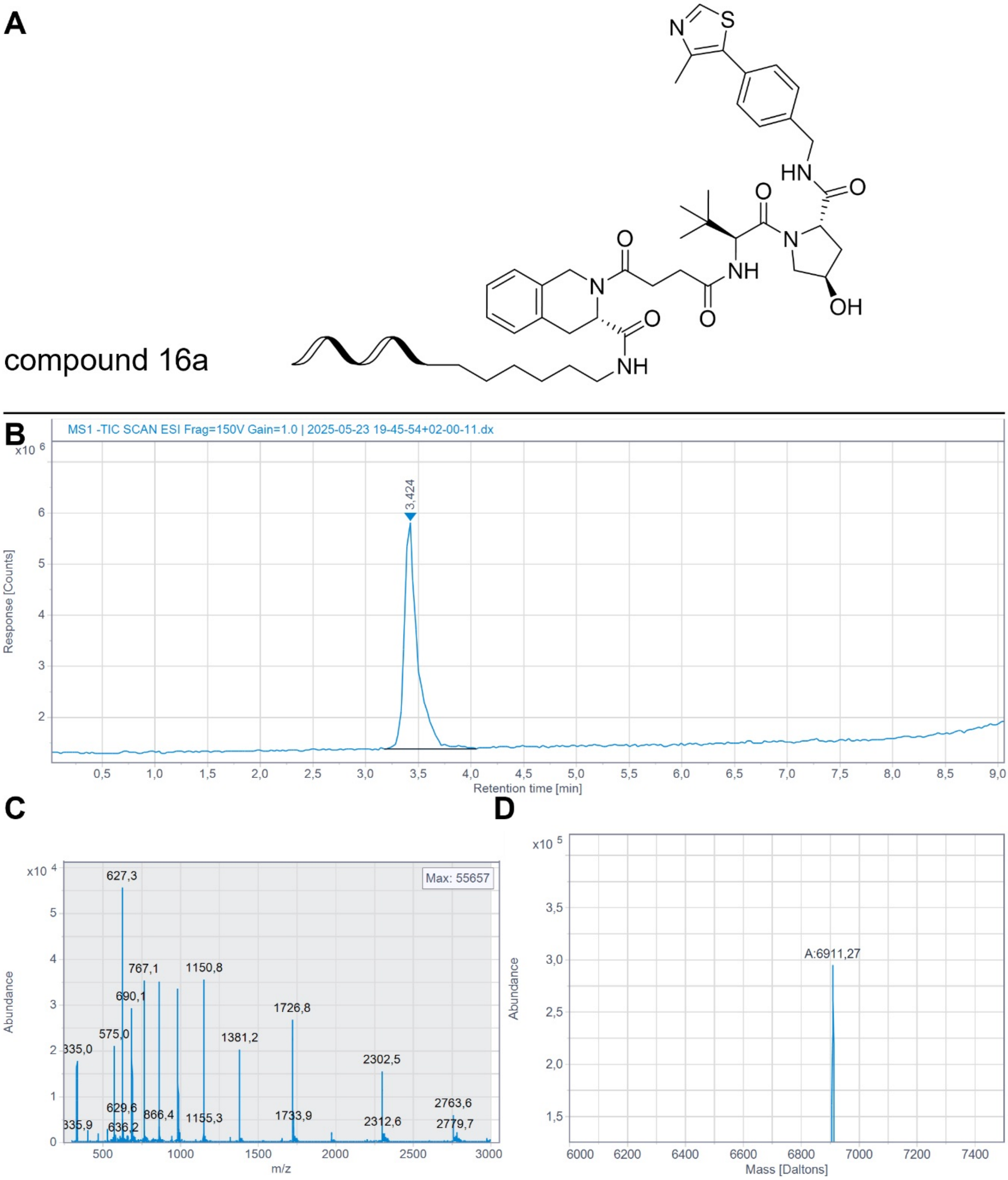

Figure SI-A23. (A) Structure of Compound 16a. (B) LCMS chromatogram of purified compound 16a. (C) ESI(-)-MS spectrum of purified compound 16a. (D) Deconvoluted mass of purified compound 16a. Mass expected: 6910.64. Mass found: 6911.27.

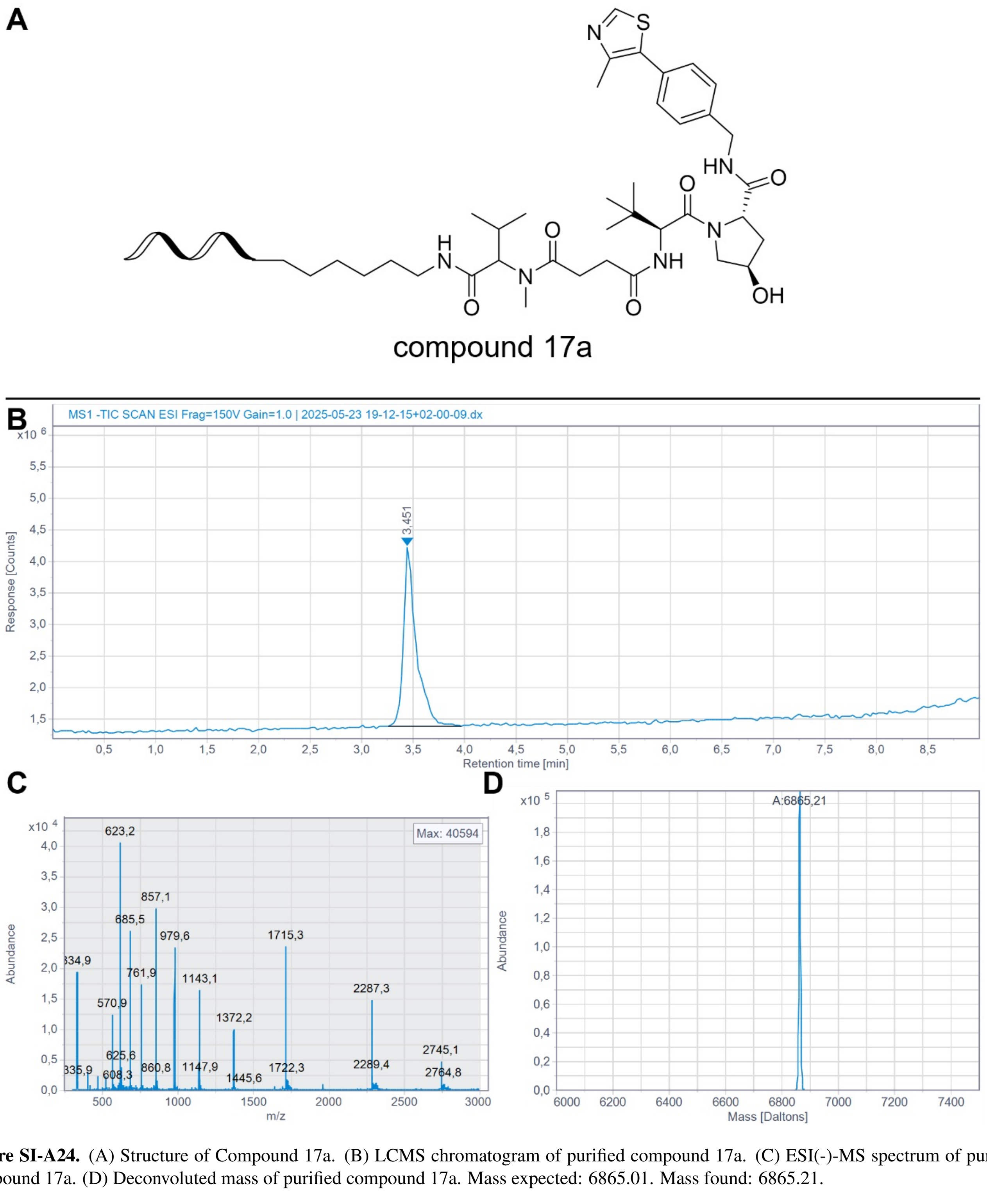

Figure SI-A24. (A) Structure of Compound 17a. (B) LCMS chromatogram of purified compound 17a. (C) ESI(-)-MS spectrum of purified compound 17a. (D) Deconvoluted mass of purified compound 17a. Mass expected: 6865.01. Mass found: 6865.21.

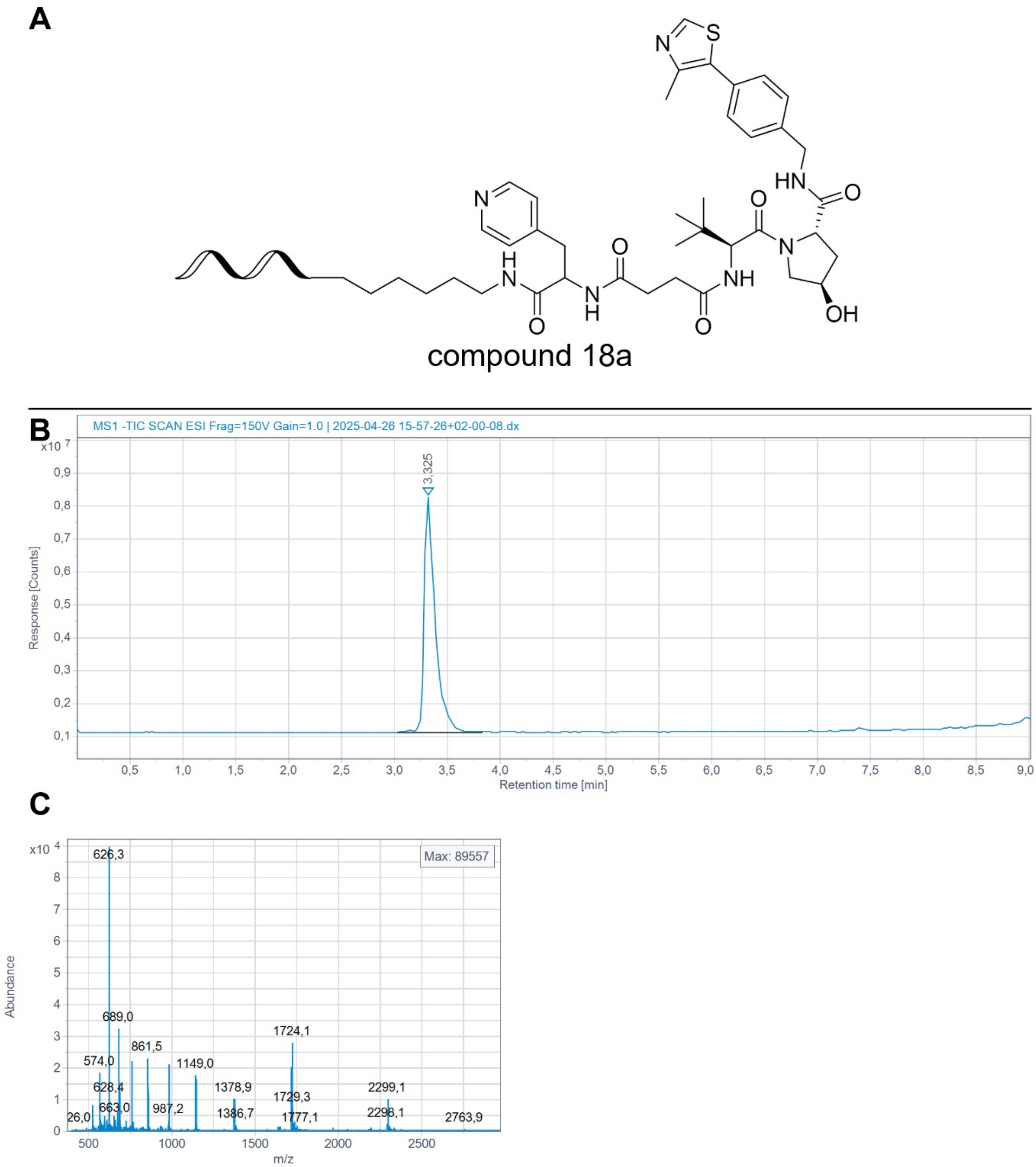

Figure SI-A25. (A) Structure of Compound 18a. (B) LCMS chromatogram of purified compound 18a. (C) ESI(-)-MS spectrum of purified compound 18a. Automated deconvolution failed. Mass expected: 6900.04. Mass found: 6900.2 (mean of manual deconvolution with ion sets: [(626.3 x11) +11] + [(689.0 x10) +10] + [(1724.1 x4) +4]]).

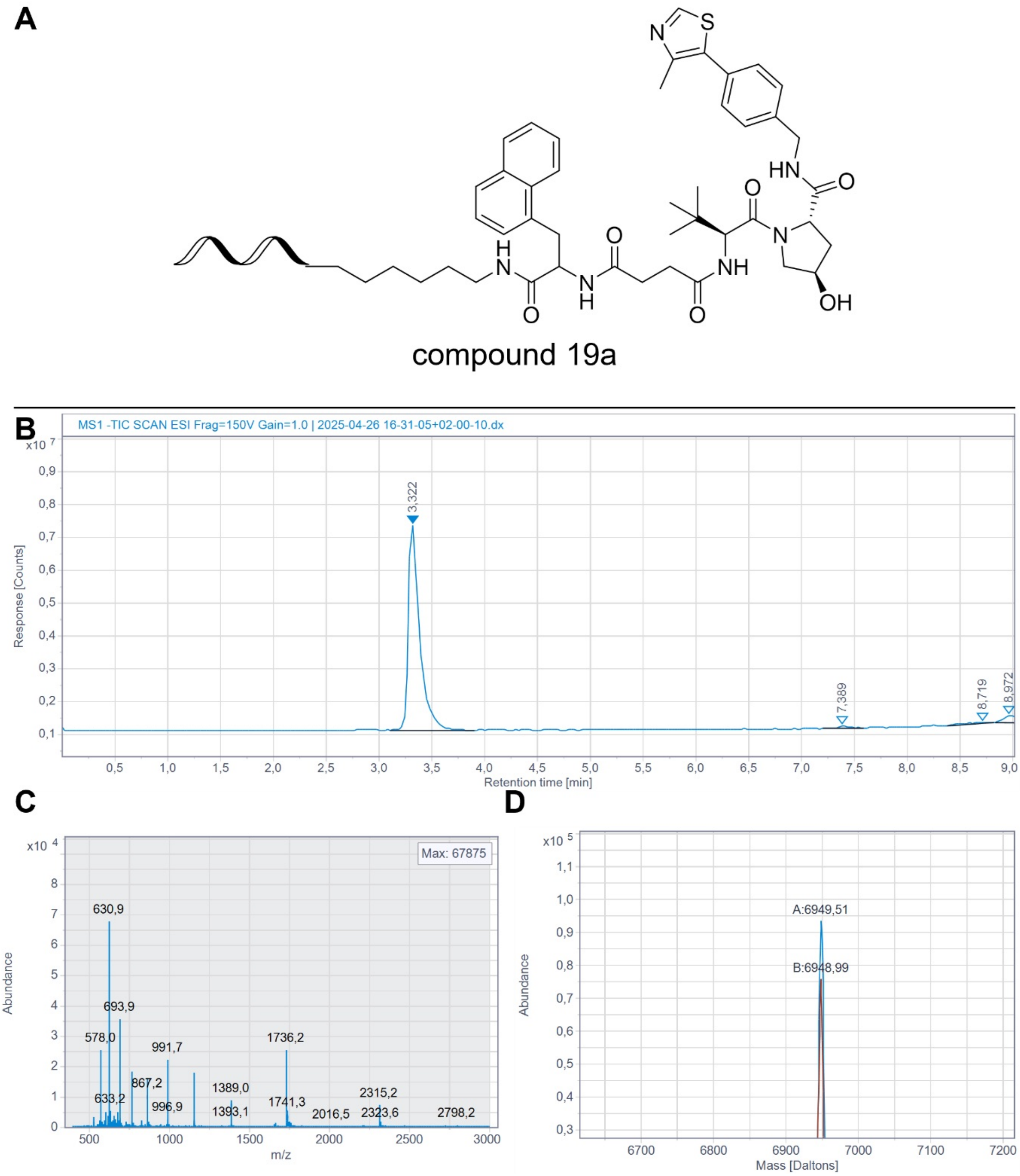

Figure SI-A26. (A) Structure of Compound 19a. (B) LCMS chromatogram of purified compound 19a. (C) ESI(-)-MS spectrum of purified compound 19a. (D) Deconvoluted mass of purified compound 19a. Mass expected: 6949.14. Mass found: 6949.51.

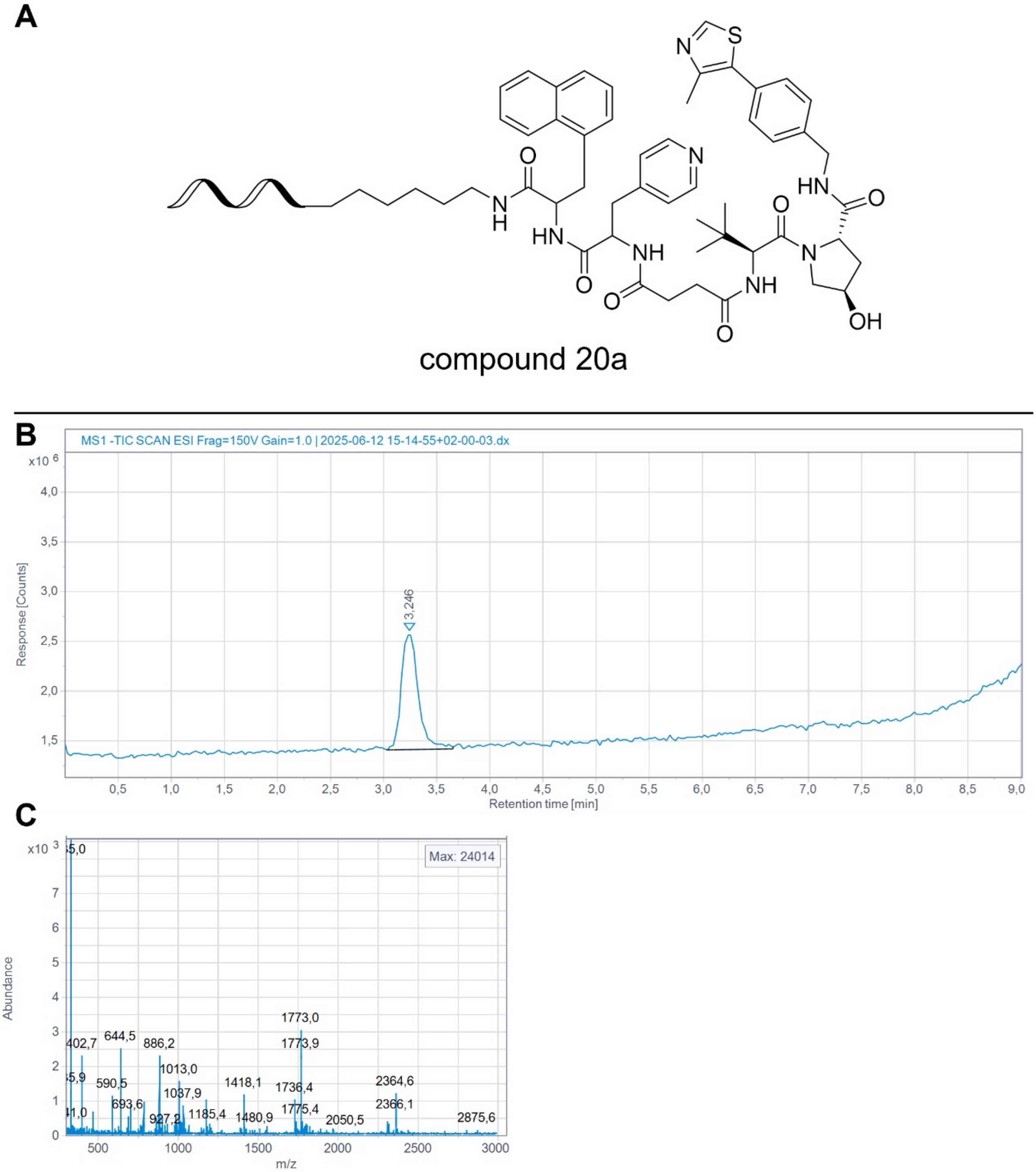

Figure SI-A27. (A) Structure of Compound 20a. (B) LCMS chromatogram of purified compound 20a. (C) ESI(-)-MS spectrum of purified compound 20a. Automated deconvolution failed. Mass expected: 7097.92. Mass found: 7097.8 (mean of manual deconvolution with ion sets: [(644.5 x11) +11] + [(1773.0 x4) +4] + [(2364.6 x3) +3]]).

Compound Characterization – Library 2

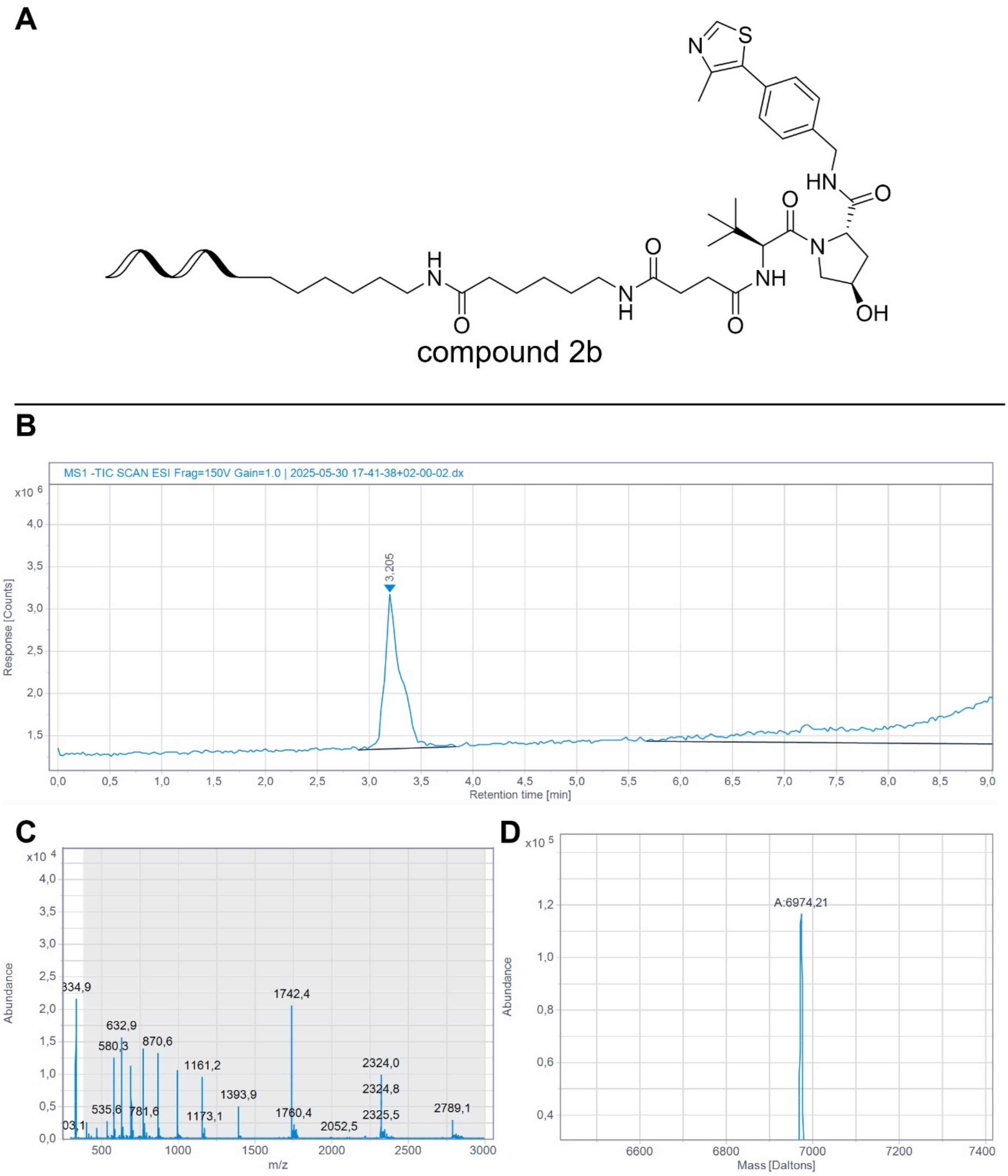

Figure SI-A28. (A) Structure of Compound 2b. (B) LCMS chromatogram of purified compound 2b. (C) ESI(-)-MS spectrum of purified compound 2b. (D) Deconvoluted mass of purified compound 2b. Mass expected: 6974.05. Mass found: 6974.21.

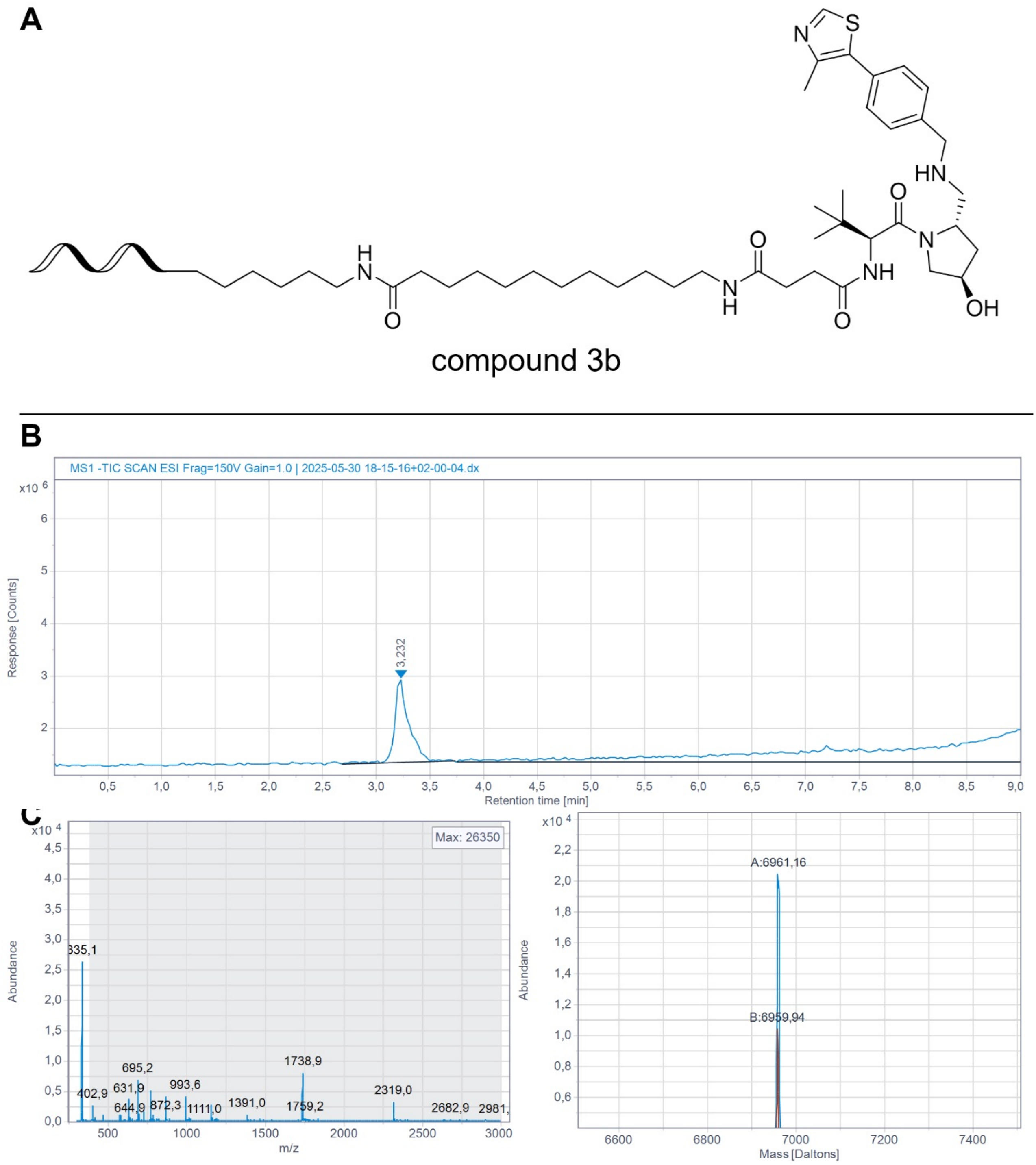

Figure SI-A29. (A) Structure of Compound 3b. (B) LCMS chromatogram of purified compound 3b. (C) ESI(-)-MS spectrum of purified compound 3b. (D) Deconvoluted mass of purified compound 3b. Mass expected: 6960.21. Mass found: 6961.16.

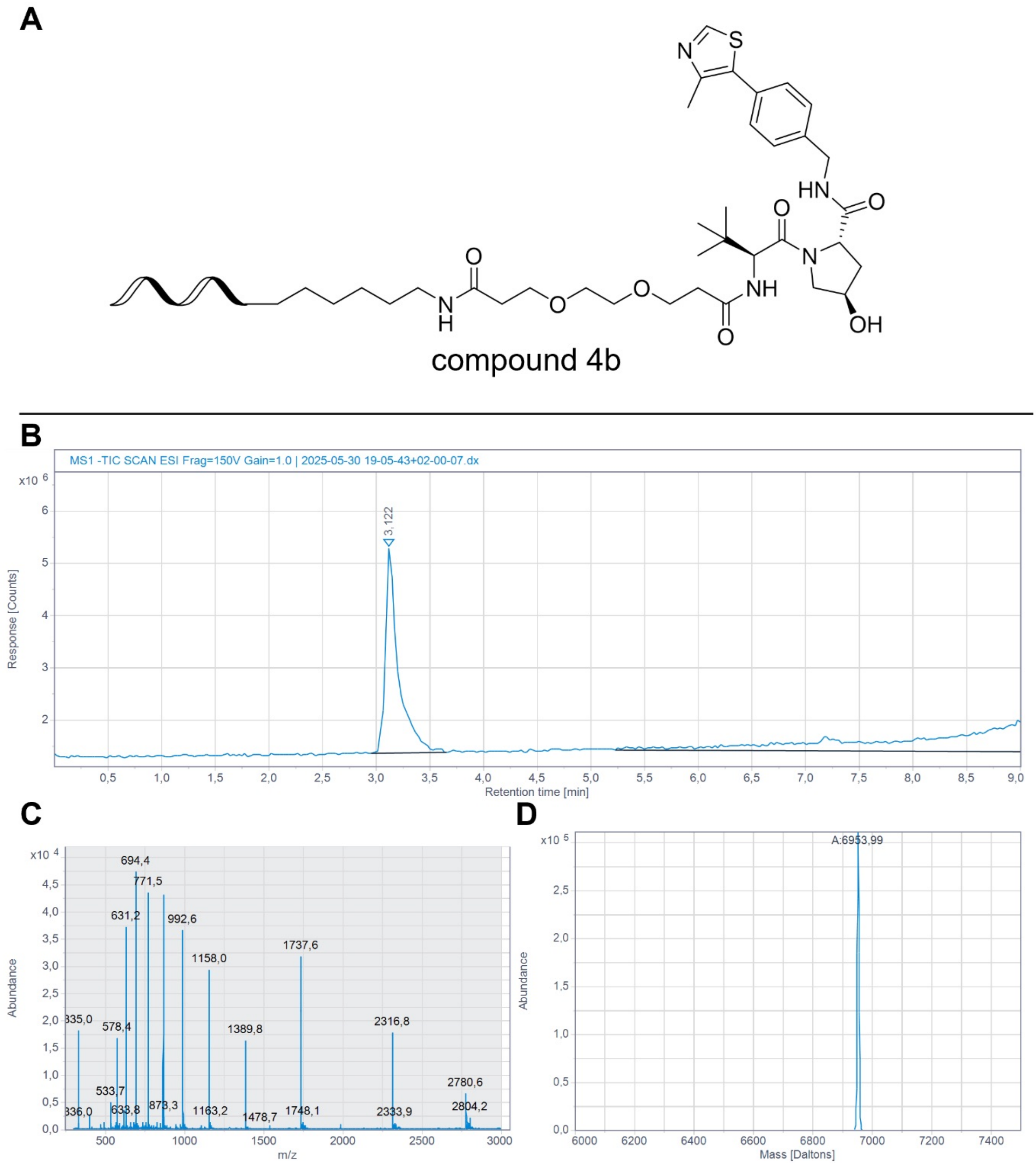

Figure SI-A30. (A) Structure of Compound 4b. (B) LCMS chromatogram of purified compound 4b. (C) ESI(-)-MS spectrum of purified compound 4b. (D) Deconvoluted mass of purified compound 4b. Mass expected: 6953.70. Mass found: 6953.99.

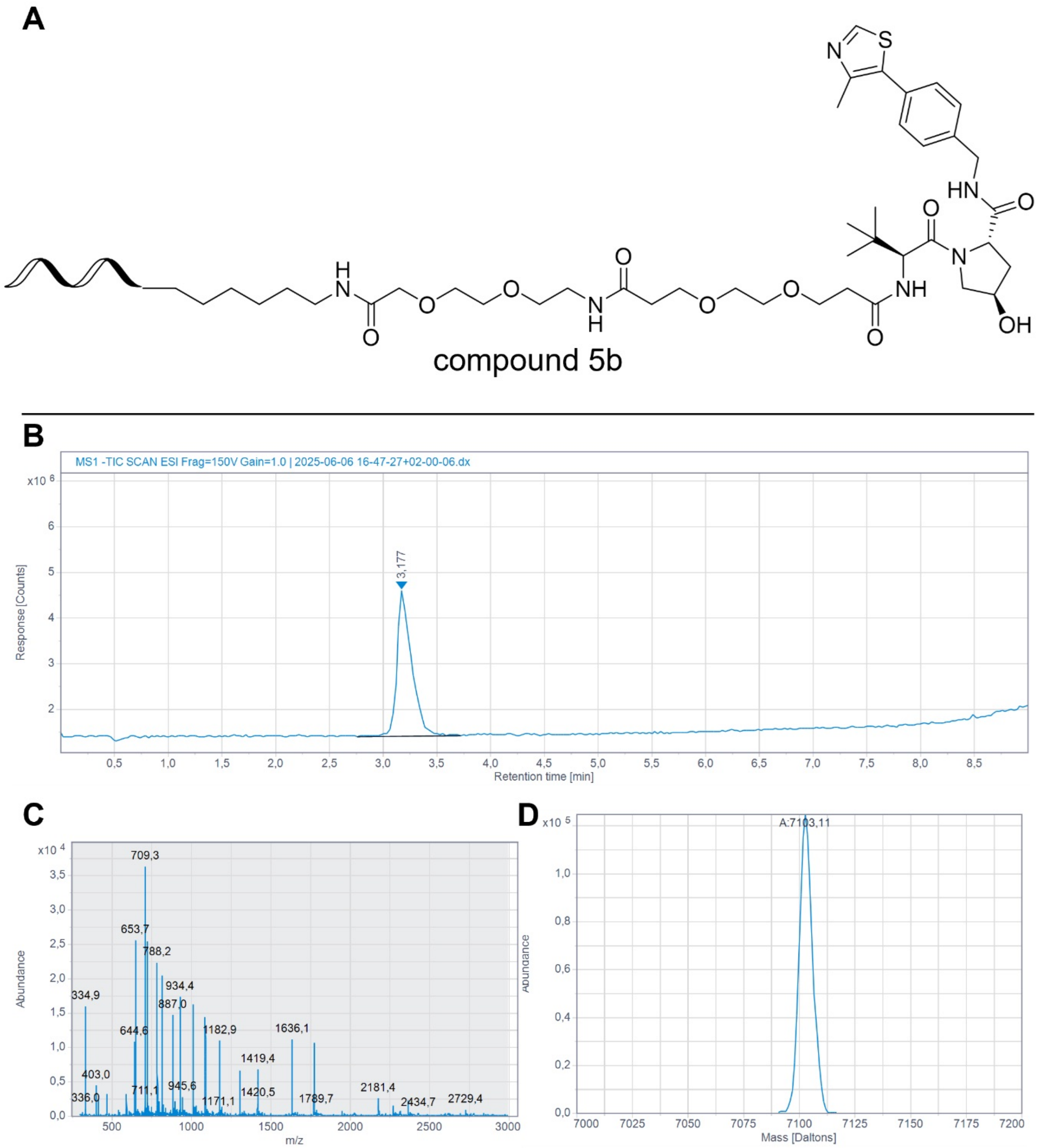

Figure SI-A31. (A) Structure of Compound 5b. (B) LCMS chromatogram of purified compound 5b. (C) ESI(-)-MS spectrum of purified compound 5b. (D) Deconvoluted mass of purified compound 5b. Mass expected: 7103.11. Mass found: 7103.11.

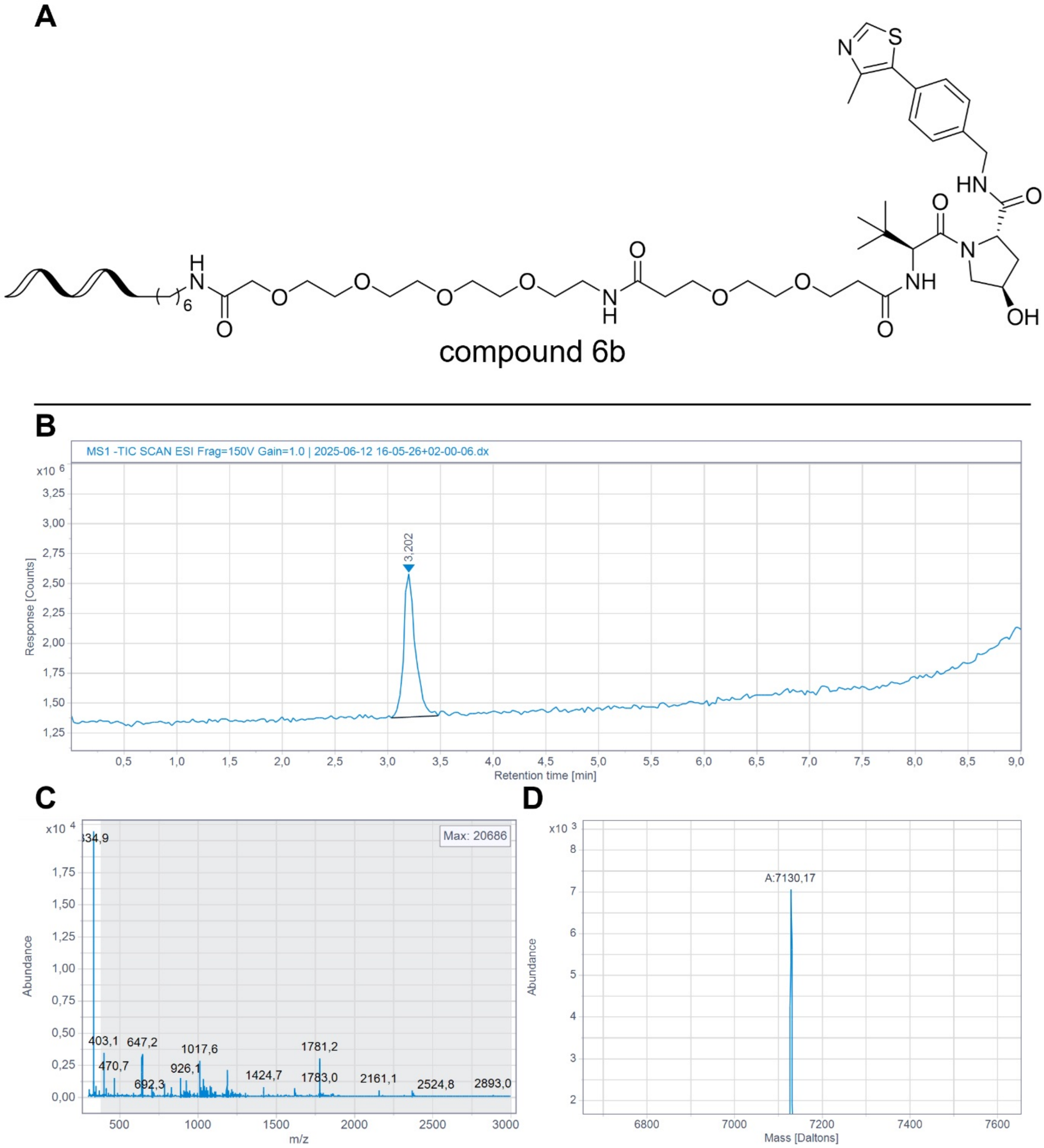

Figure SI-A32. (A) Structure of Compound 6b. (B) LCMS chromatogram of purified compound 6b. (C) ESI(-)-MS spectrum of purified compound 6b. (D) Deconvoluted mass of purified compound 6b. Mass expected: 7130.22. Mass found: 7130.17.

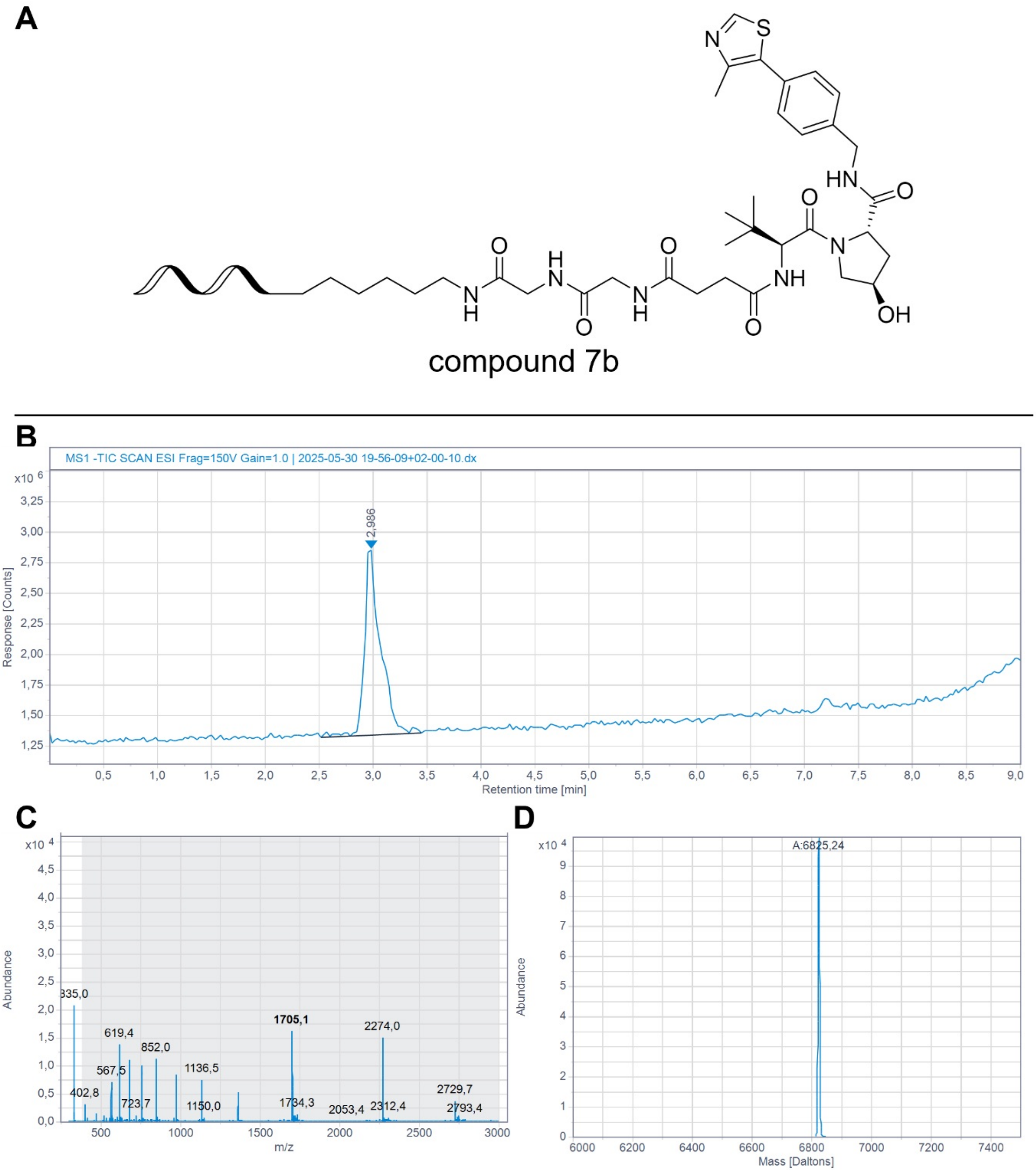

Figure SI-A33. (A) Structure of Compound 7b. (B) LCMS chromatogram of purified compound 7b. (C) ESI(-)-MS spectrum of purified compound 7b. (D) Deconvoluted mass of purified compound 7b. Mass expected: 6825.00. Mass found: 6825.24.

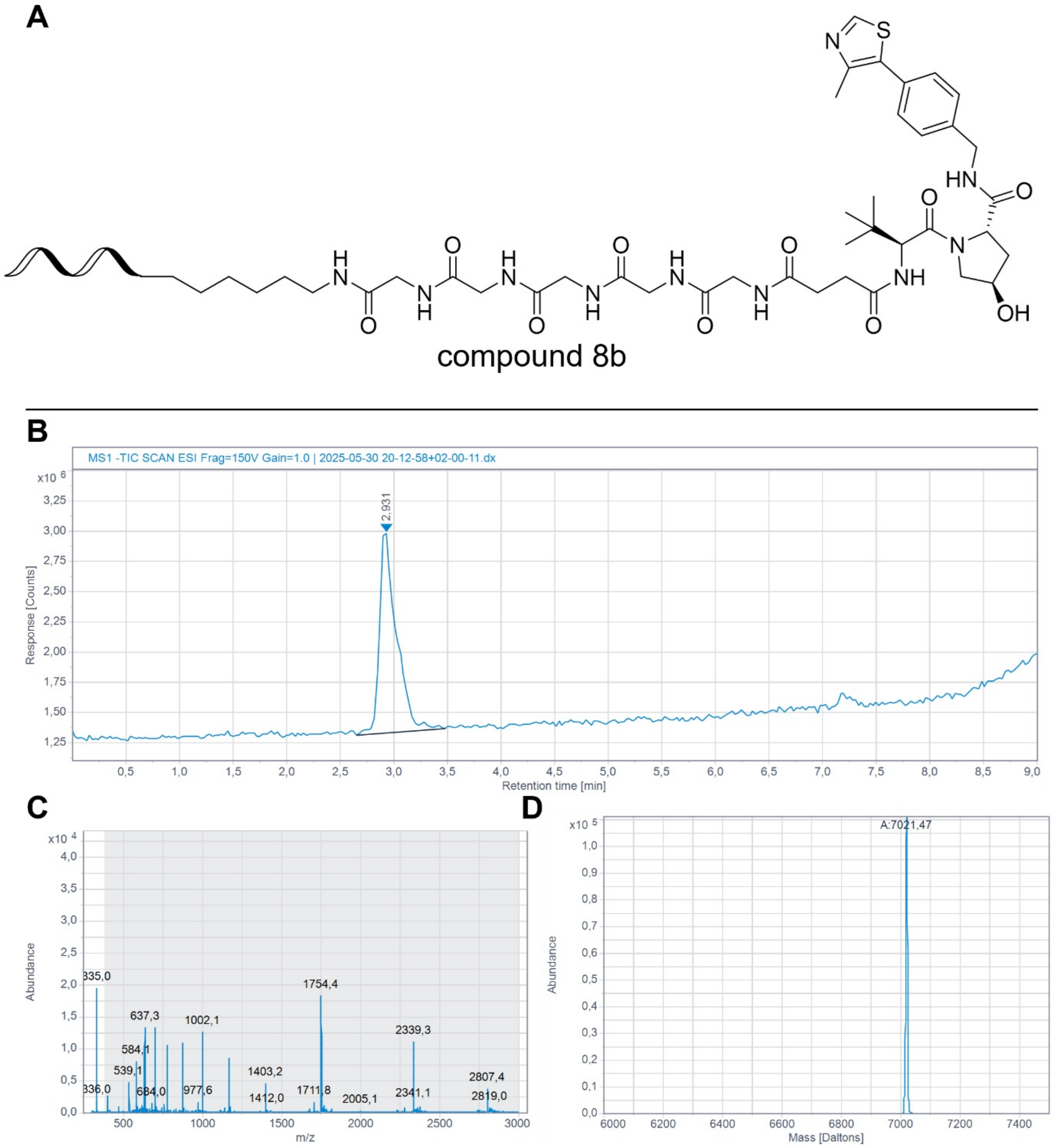

Figure SI-A34. (A) Structure of Compound 8b. (B) LCMS chromatogram of purified compound 8b. (C) ESI(-)-MS spectrum of purified compound 8b. (D) Deconvoluted mass of purified compound 8b. Mass expected: 7021.10. Mass found: 7021.47.

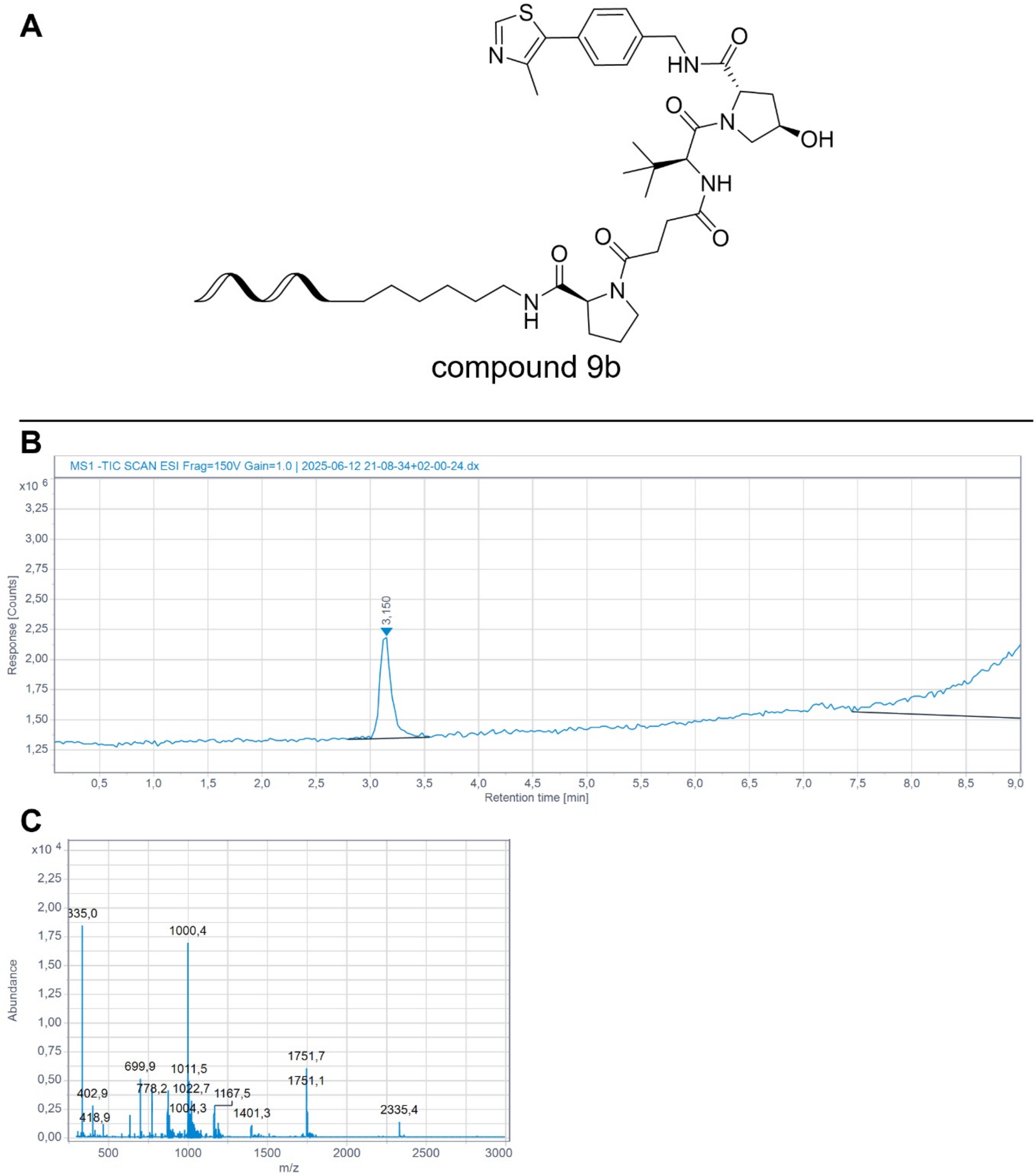

Figure SI-A35. (A) Structure of Compound 9b. (B) LCMS chromatogram of purified compound 9b. (C) ESI(-)-MS spectrum of purified compound 9b. Automated deconvolution failed. Mass expected: 7009.64. Mass found: 7009.93 (mean of manual deconvolution with ion sets: [(1000.4 x7) +7] + [(1751.7 x4) +4] + [(2335.4 x3) +3]).

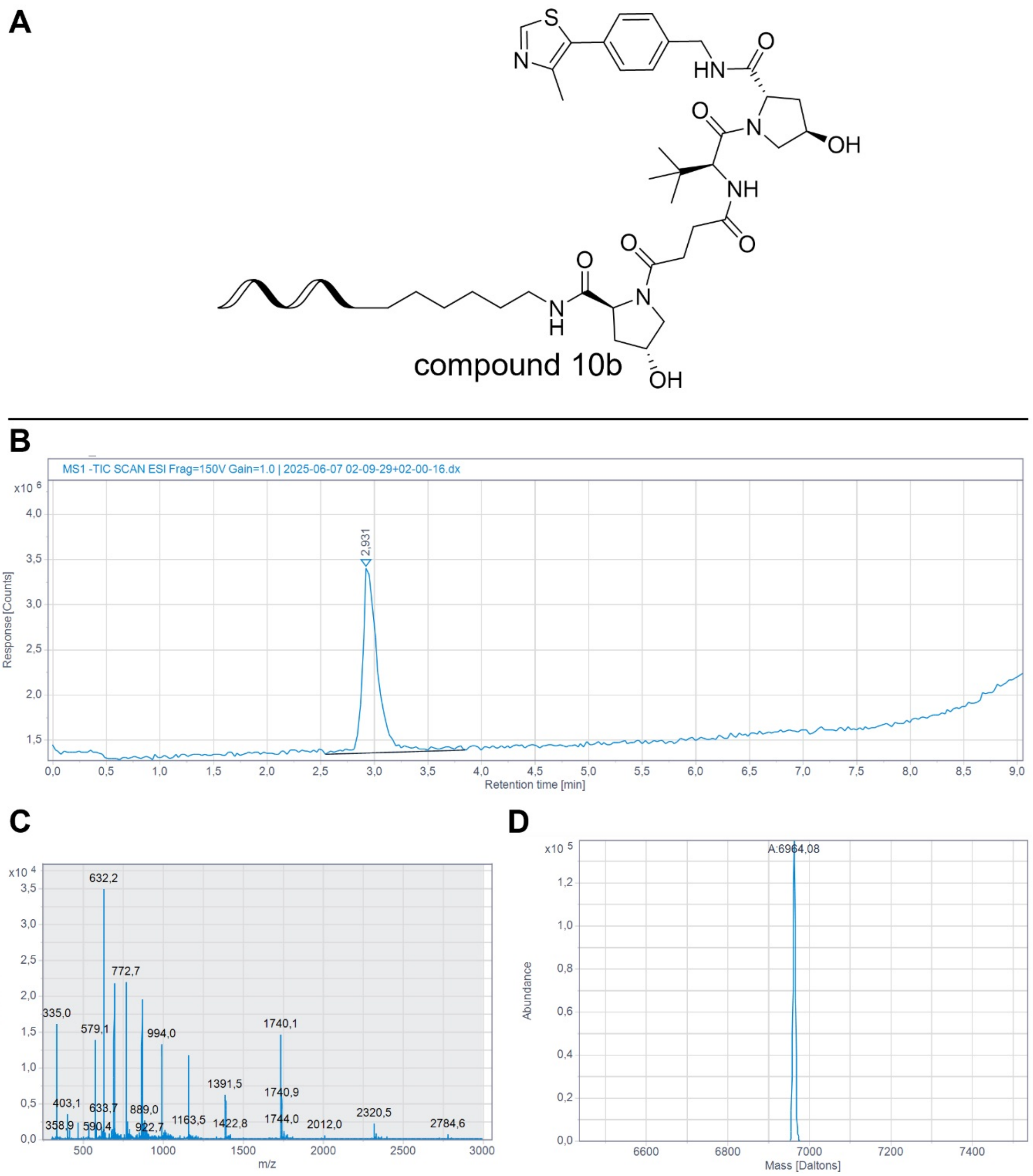

Figure SI-A36. (A) Structure of Compound 10b. (B) LCMS chromatogram of purified compound 10b. (C) ESI(-)-MS spectrum of purified compound 10b. (D) Deconvoluted mass of purified compound 10b. Mass expected: 6964.01. Mass found: 6964.08.

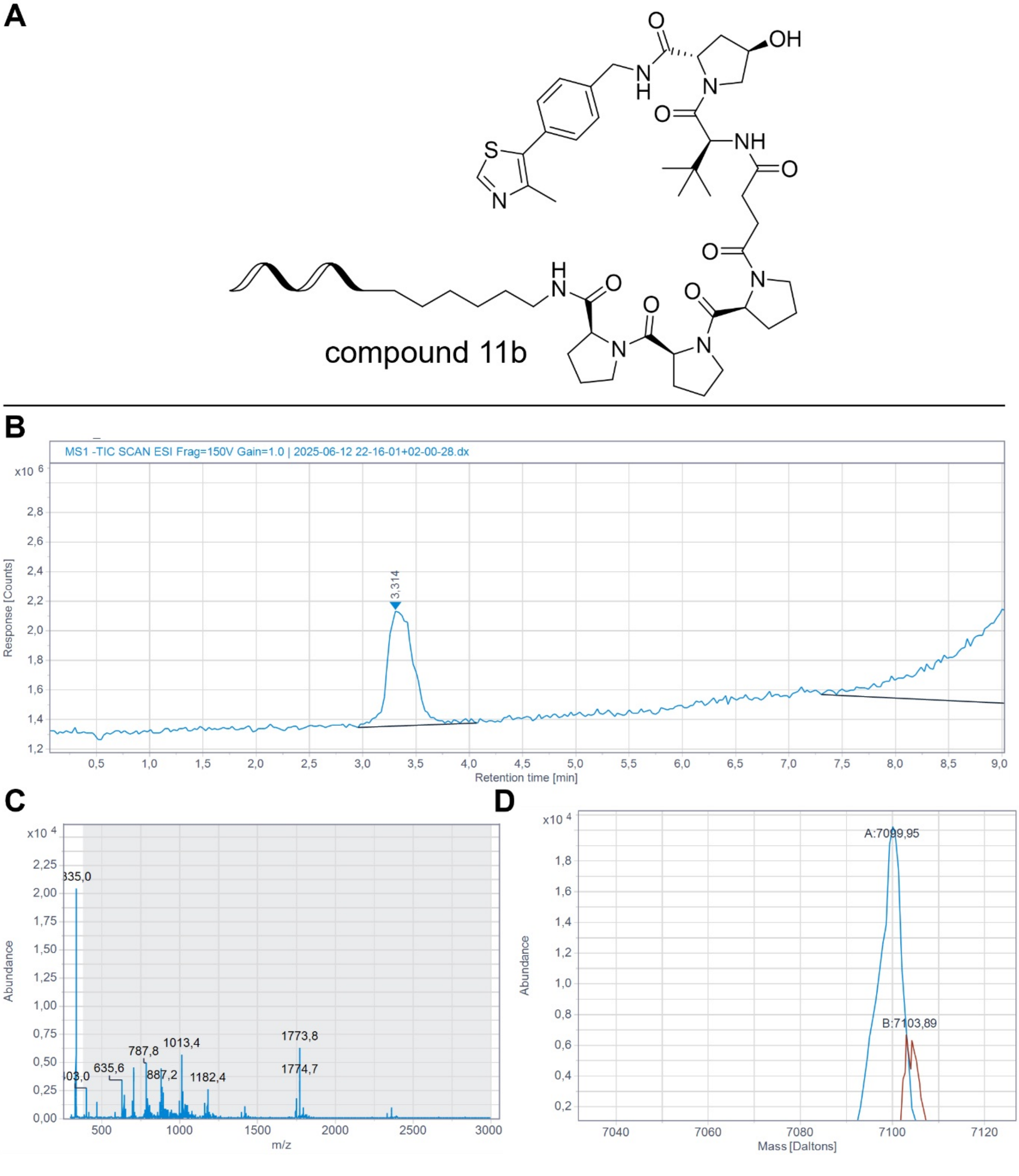

Figure SI-A37. (A) Structure of Compound 11b. (B) LCMS chromatogram of purified compound 11b. (C) ESI(-)-MS spectrum of purified compound 11b. (D) Deconvoluted mass of purified compound 11b. Mass expected: 7099.64. Mass found: 7099.95.

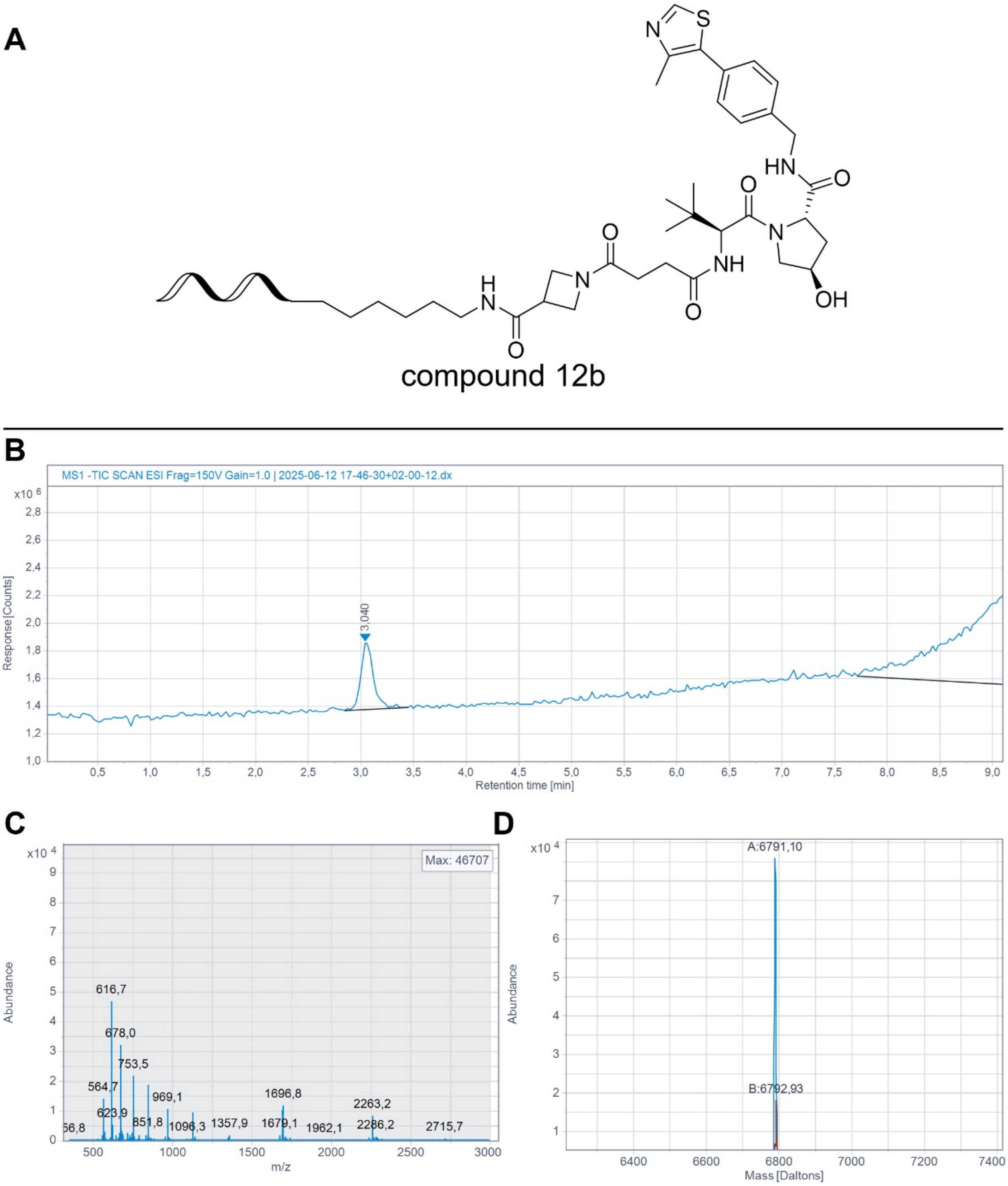

Figure SI-A38. (A) Structure of Compound 12b. (B) LCMS chromatogram of purified compound 12b. (C) ESI(-)-MS spectrum of purified compound 12b. (D) Deconvoluted mass of purified compound 12b. Mass expected: 6803.98. Mass found: 6791.10. We hypothesize in-source fragmentation with carbon-loss due to ESI.

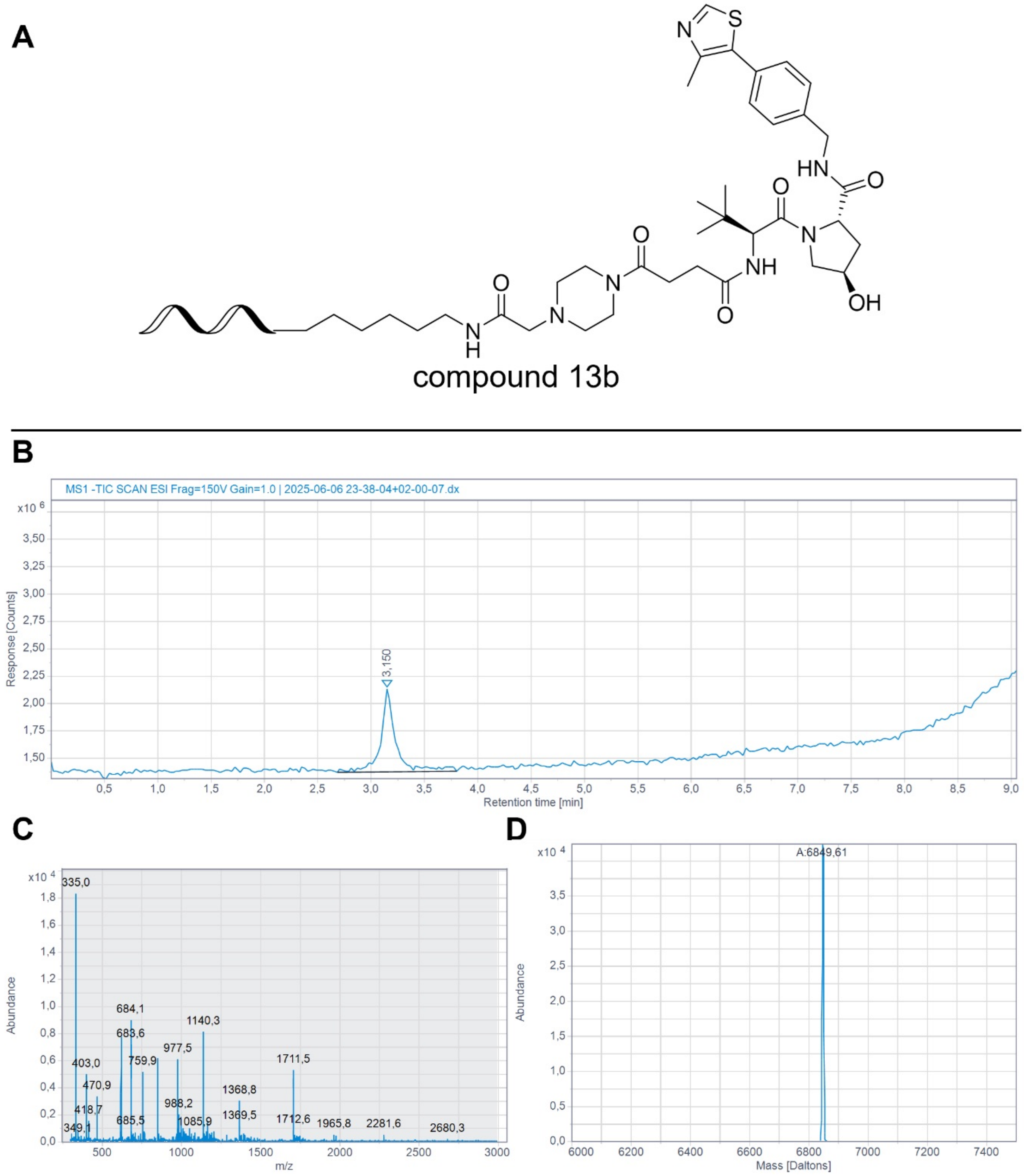

Figure SI-A39. (A) Structure of Compound 13b. (B) LCMS chromatogram of purified compound 13b. (C) ESI(-)-MS spectrum of purified compound 13b. (D) Deconvoluted mass of purified compound 13b. Mass expected: 6849.05. Mass found: 6849.61.

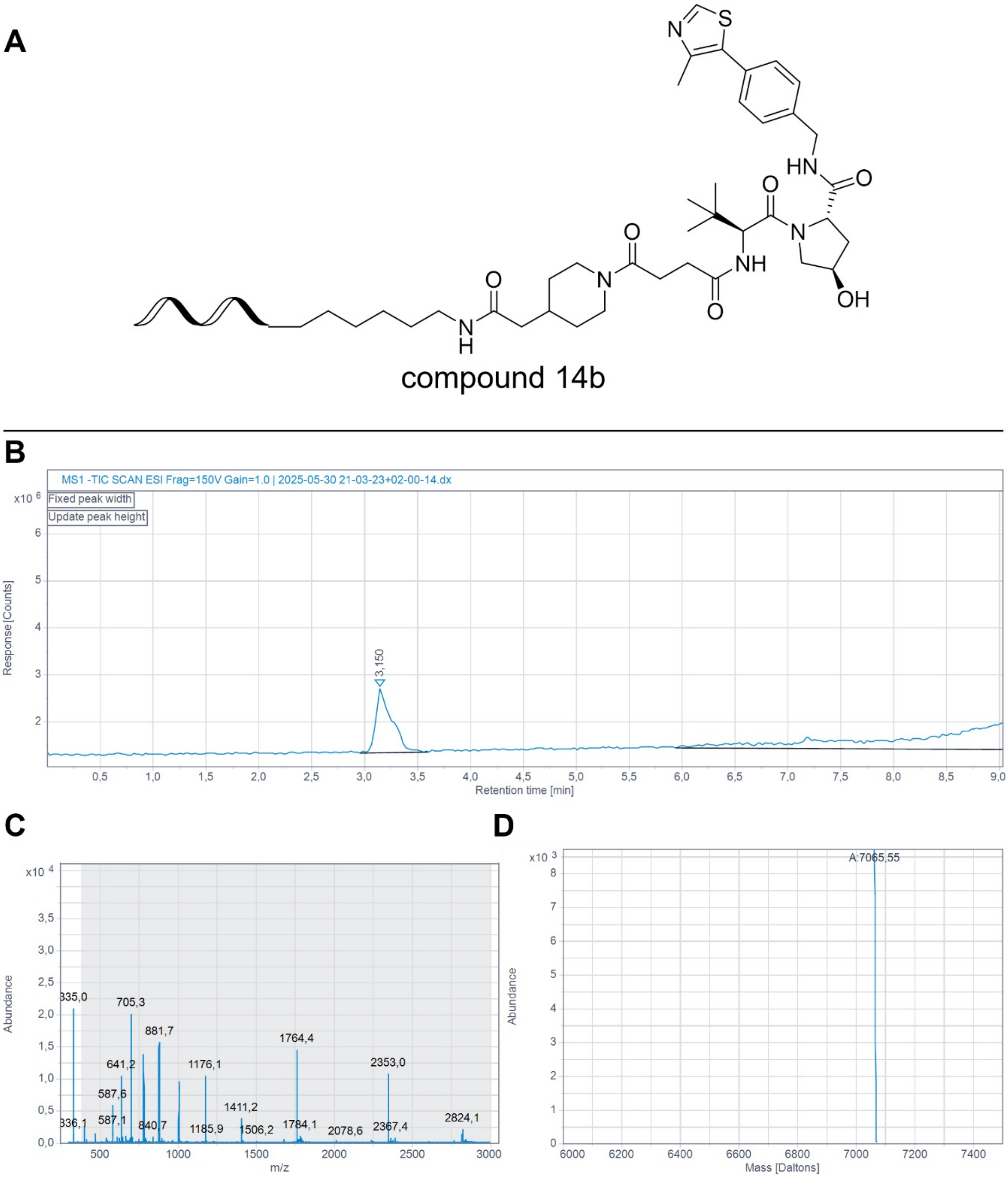

Figure SI-A40. (A) Structure of Compound 14b. (B) LCMS chromatogram of purified compound 14b. (C) ESI(-)-MS spectrum of purified compound 14b. (D) Deconvoluted mass of purified compound 14b. Mass expected: 7065.06. Mass found: 7065.55.

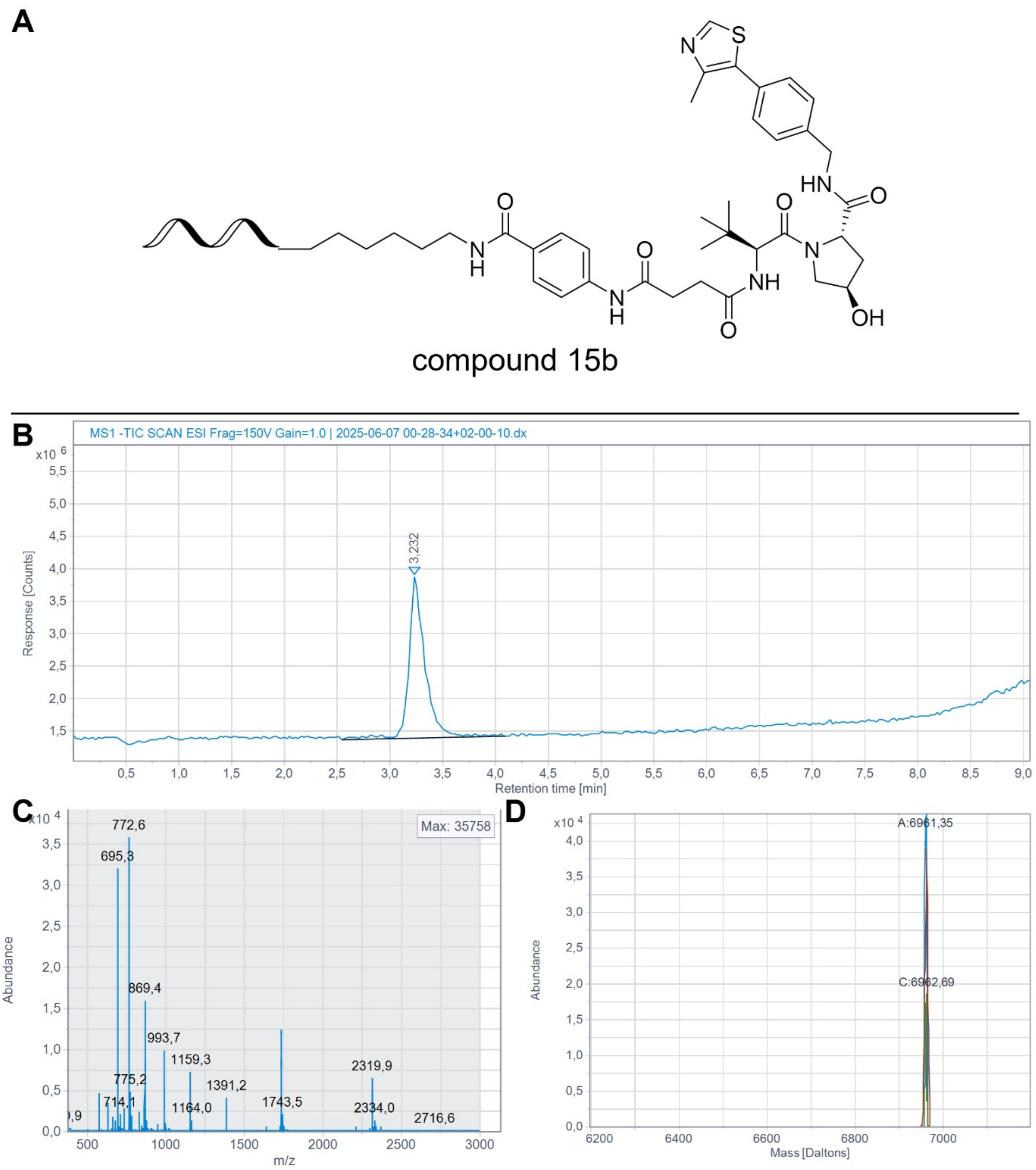

Figure SI-A41. (A) Structure of Compound 15b. (B) LCMS chromatogram of purified compound 15b. (C) ESI(-)-MS spectrum of purified compound 15b. (D) Deconvoluted mass of purified compound 15b. Mass expected: 6962.02. Mass found: 6961.35.

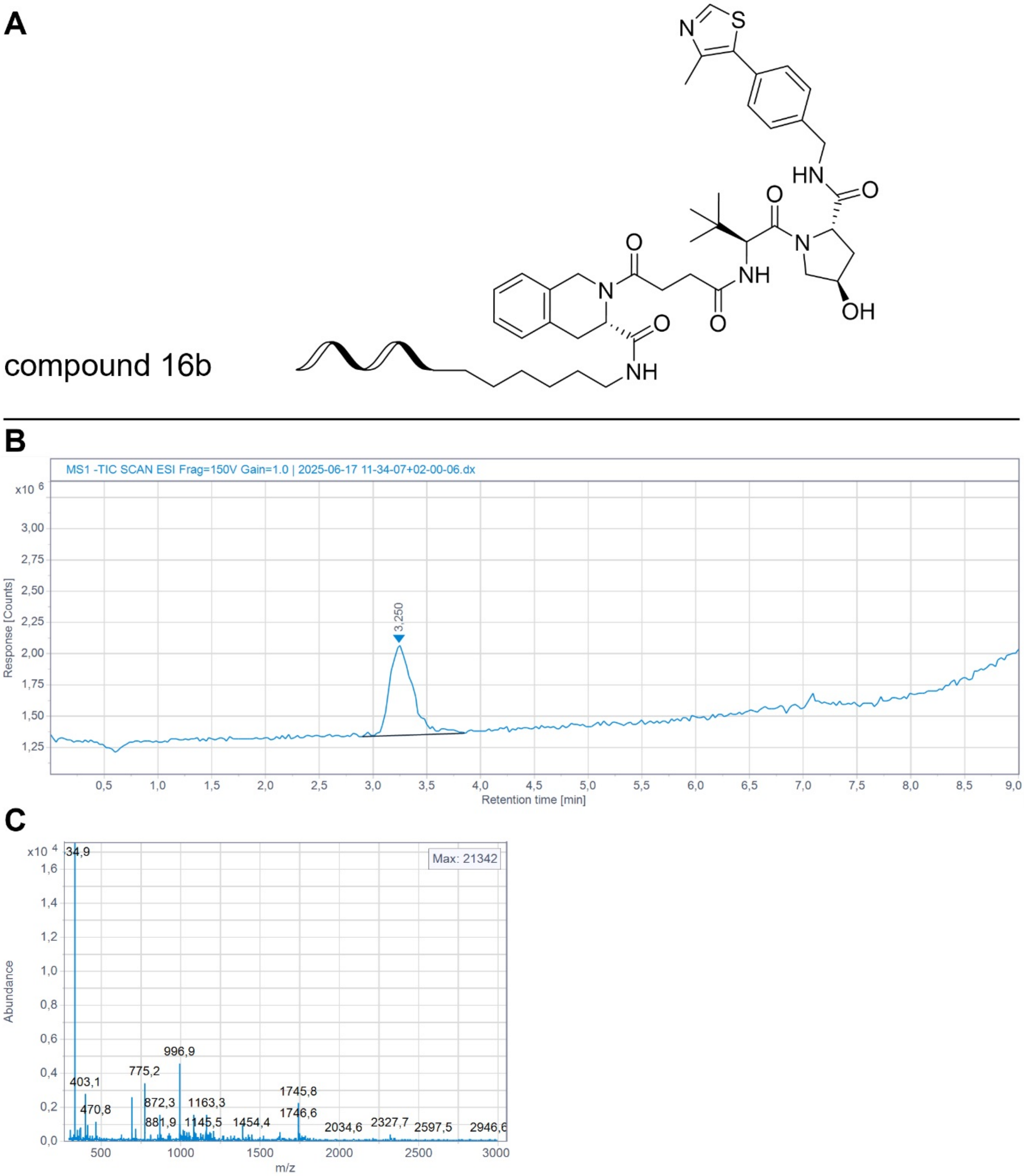

Figure SI-A42. (A) Structure of Compound 16b. (B) LCMS chromatogram of purified compound 16b. (C) ESI(-)-MS spectrum of purified compound 16b. Automated deconvolution failed. Mass expected: 6986.08. Mass found: 6986.1 (mean of manual deconvolution with ion sets: [(775.2 x9) +9] + [(996.9 x7) +7] + [(1745.8 x4) +4]]).

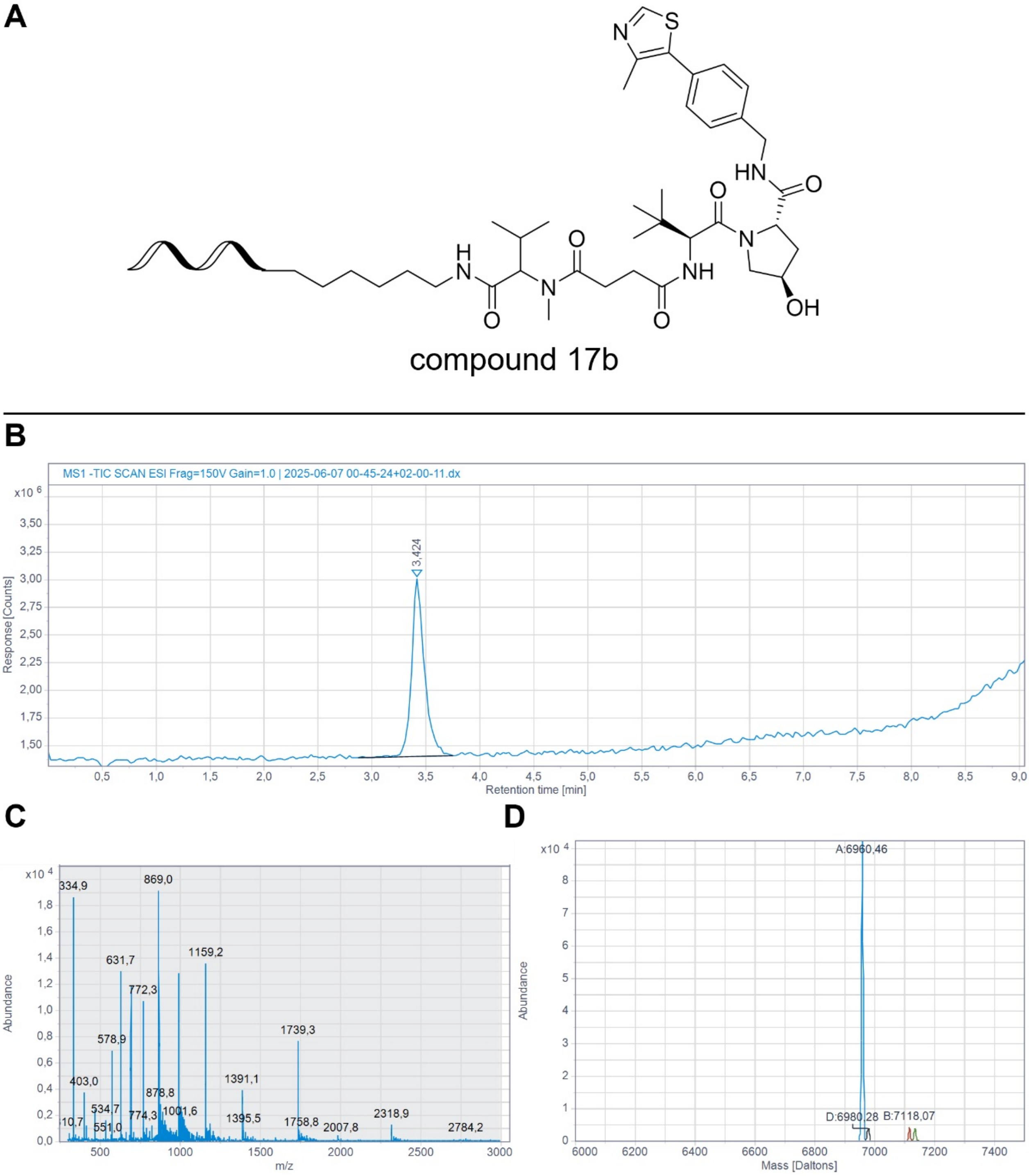

Figure SI-A43. (A) Structure of Compound 17b. (B) LCMS chromatogram of purified compound 17b. (C) ESI(-)-MS spectrum of purified compound 17b. (D) Deconvoluted mass of purified compound 17b. Mass expected: 6961.05. Mass found: 6960.46.

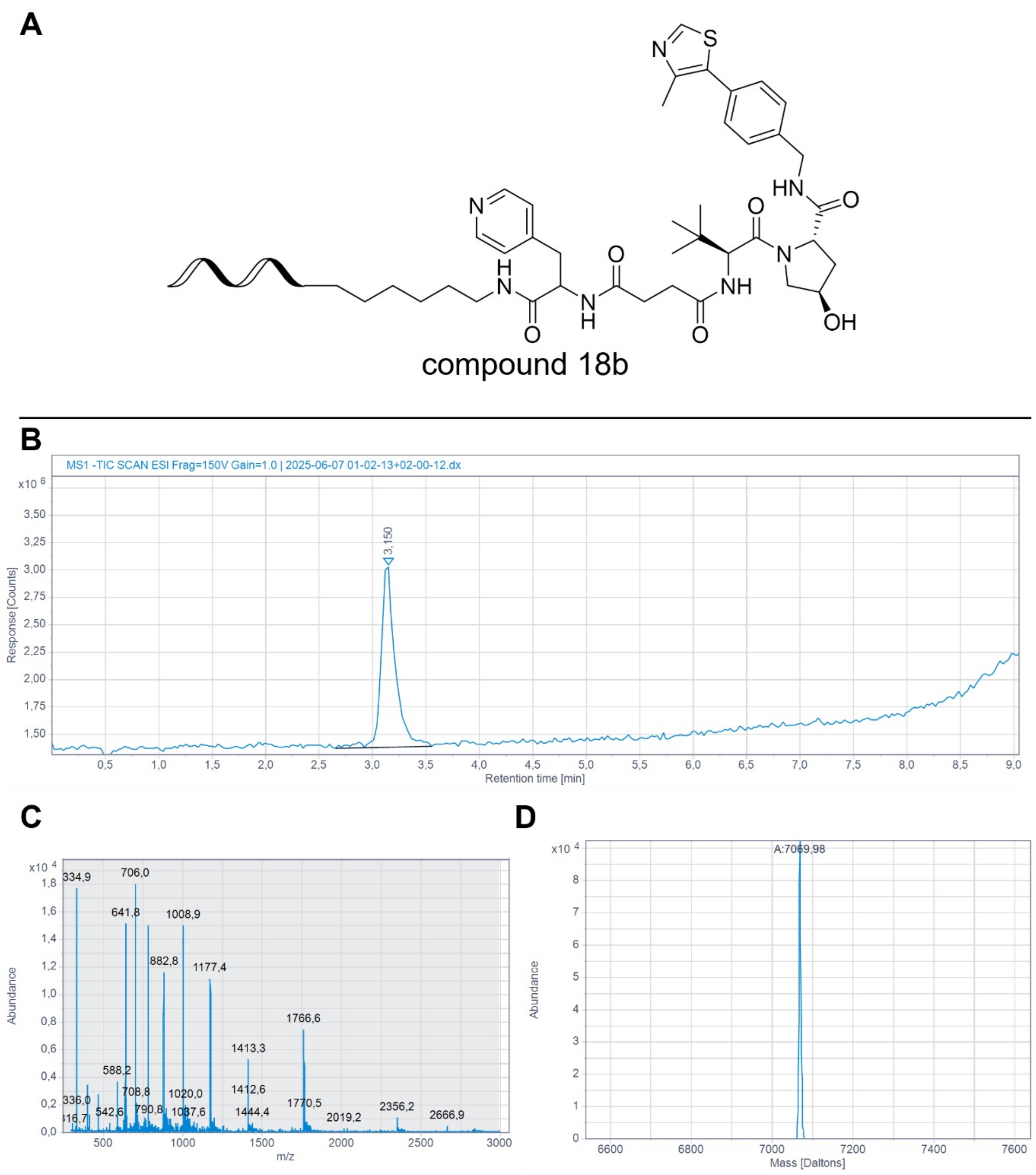

Figure SI-A44. (A) Structure of Compound 18b. (B) LCMS chromatogram of purified compound 18b. (C) ESI(-)-MS spectrum of purified compound 18b. (D) Deconvoluted mass of purified compound 18b. Mass expected: 7071.06. Mass found: 7069.88.

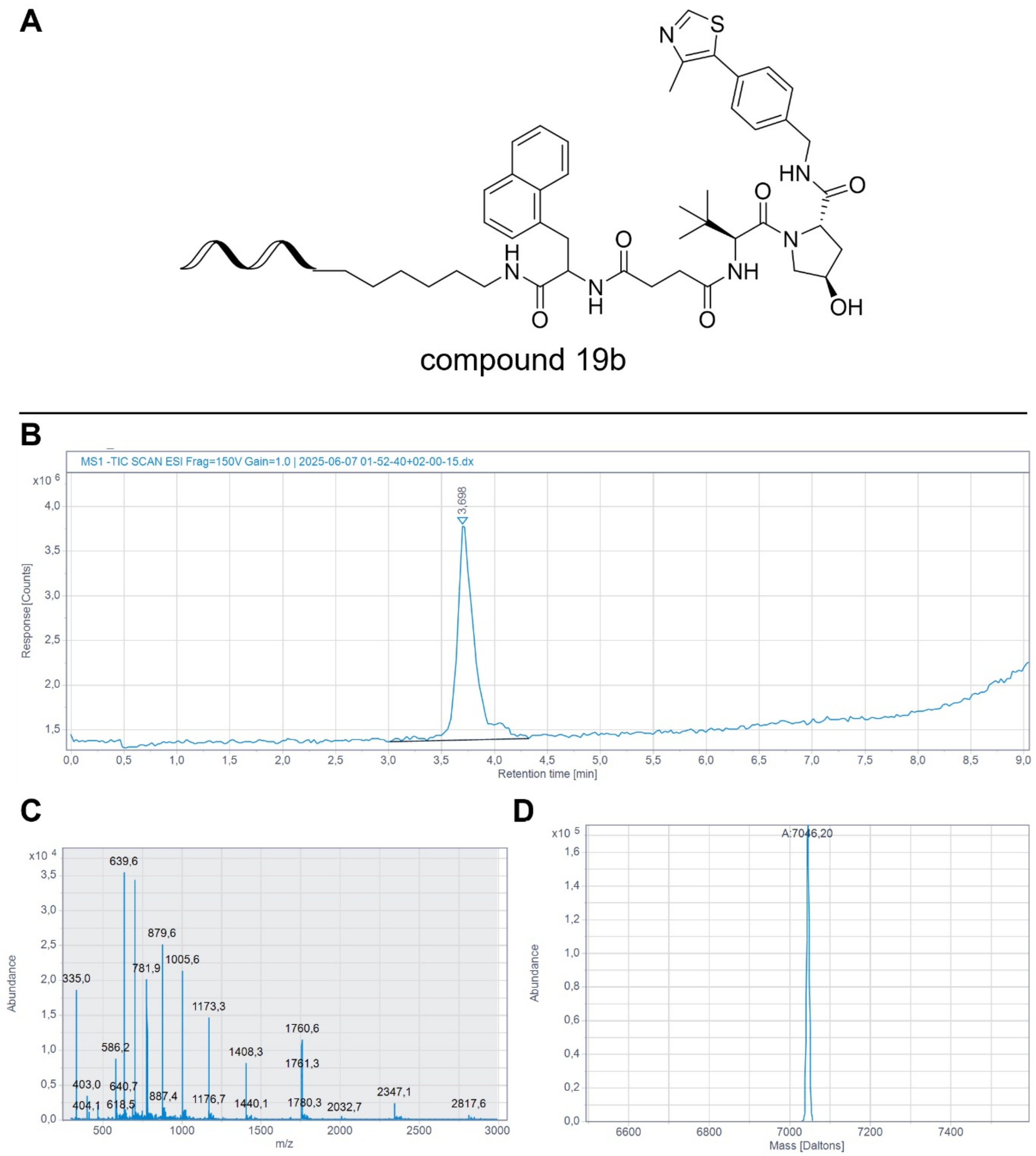

Figure SI-A45. (A) Structure of Compound 19b. (B) LCMS chromatogram of purified compound 19b. (C) ESI(-)-MS spectrum of purified compound 19b. (D) Deconvoluted mass of purified compound 19b. Mass expected: 7046.14. Mass found: 7046.20.

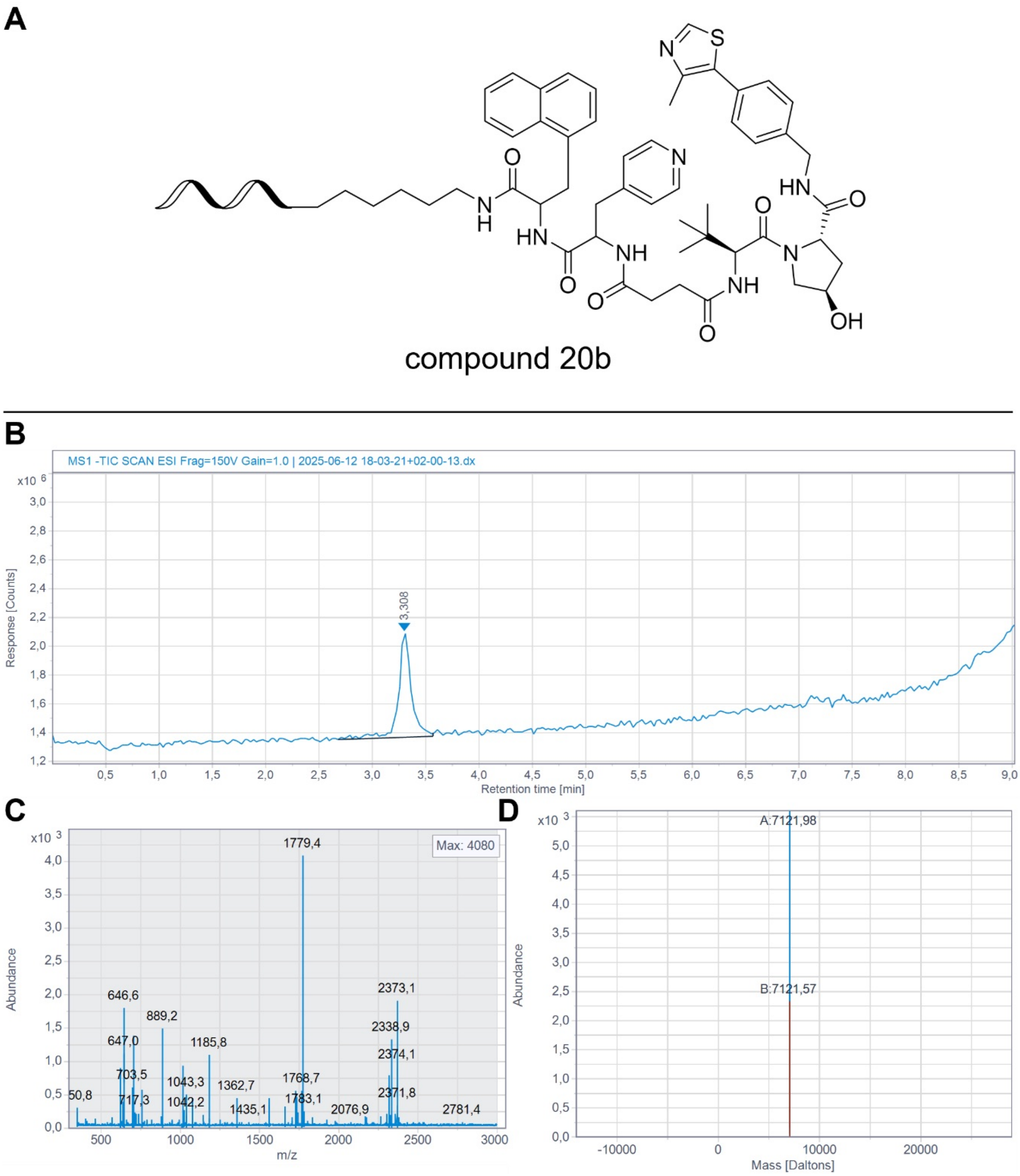

Figure SI-A46. (A) Structure of Compound 20b. (B) LCMS chromatogram of purified compound 20b. (C) ESI(-)-MS spectrum of purified compound 20b. (D) Deconvoluted mass of purified compound 20b. Mass expected: 7122.92. Mass found: 7121.98.

Table SI-A47: Physicochemical Descriptors

The following table presents the calculated physicochemical descriptors for the linker moieties of all compounds. Since the "a" and "b" variants differ only in their attached DNA sequences, the physicochemical properties of the linkers are identical. Descriptors were calculated using ChemAxon software and include cLogP (calculated partition coefficient), cLogS (calculated aqueous solubility), number of hydrogen bond acceptors and donors, total surface area, relative polar surface area (PSA), and absolute polar surface area.

Table SI-A47. Physicochemical descriptors for linker moieties. Values are identical for a/b variants as they differ only in DNA sequence.

Compound	cLogP	cLogS	H-Acc	H-Don	TSA (Å^2)	Rel. PSA	PSA (Å^2)
**1**	−0.76	−0.46	4	2	124.0	0.40	58.2
**2a/2b**	0.14	−1.31	6	3	222.0	0.33	87.3
**3a/3b**	2.87	−2.93	6	3	304.5	0.24	87.3
**4a/4b**	−0.94	−0.30	6	2	199.0	0.35	76.7
**5a/5b**	−2.05	0.09	10	3	317.0	0.36	124.2
**6a/6b**	−2.23	0.25	12	3	392.1	0.34	142.7
**7a/7b**	−2.59	0.01	8	4	209.9	0.47	116.4
**8a/8b**	−4.42	0.47	12	6	295.8	0.50	174.6
**9a/9b**	−0.70	−0.63	6	2	192.3	0.34	78.5
**10a/10b**	−1.55	−0.23	7	3	197.4	0.40	98.7
**11a/11b**	−0.58	−0.97	10	2	329.0	0.30	119.1
**12a/12b**	−1.10	−0.14	6	2	178.5	0.37	78.5
**13a/13b**	−1.33	0.57	7	2	217.6	0.32	81.8
**14a/14b**	0.04	−0.95	6	2	219.8	0.30	78.5
**15a/15b**	0.39	−1.97	6	3	212.9	0.35	87.3
**16a/16b**	0.02	−1.61	6	2	238.3	0.28	78.5
**17a/17b**	−0.38	−0.67	6	2	213.8	0.31	78.5
**18a/18b**	−0.88	−0.94	7	3	238.0	0.35	100.2
**19a/19b**	1.32	−3.34	6	3	273.8	0.27	87.3
**20a/20b**	1.20	−3.83	9	4	387.8	0.28	129.3

Table SI-A48. SMILES representations of linker moieties.

Compound	SMILES
**1**	`O=C([NH]C)CCC([NH]C)=O`
**2a/2b**	`O=C([NH]C)CCC(NCCCCCC([NH]C)=O)=O`
**3a/3b**	`O=C([NH]C)CCC(NCCCCCCCCCCCC([NH]C)=O)=O`
**4a/4b**	`O=C(CCOCCOCCC([NH]C)=O)[NH]C`
**5a/5b**	`O=C([NH]C)COCCOCCNC(CCOCCOCCC([NH]C)=O)=O`
**6a/6b**	`O=C(CCOCCOCCC(NCCOCCOCCOCCOCC([NH]C)=O)=O)[NH]C`
**7a/7b**	`O=C(CNC(CNC(CCC(NC)=O)=O)=O)NC`
**8a/8b**	`O=C([NH]C)CCC(NCC(NCC(NCC(NCC([NH]C)=O)=O)=O)=O)=O`
**9a/9b**	`O=C([NH]C)[C@H]1N(C(CCC([NH]C)=O)=O)CCC1`
**10a/10b**	`O[C@H]1CN(C(CCC(C)=O)=O)C(C(NC)=O)C1`
**11a/11b**	`C[NH]C([C@@H]1CCCN1C([C@@H]2CCCN2C([C@@H]3CCCN3C(CCC([NH]C)=O)=O)=O)=O)=O`
**12a/12b**	`O=C([NH]C)C1CN(C(CCC([NH]C)=O)=O)C1`
**13a/13b**	`O=C([NH]C)CN1CCN(C(CCC([NH]C)=O)=O)CC1`
**14a/14b**	`O=C(CC1CCN(C(CCC([NH]C)=O)=O)CC1)[NH]C`
**15a/15b**	`O=C(C(C=C1)=CC=C1NC(CCC([NH]C)=O)=O)[NH]C`
**16a/16b**	`O=C([NH]C)CCC(N1CC2=CC=CC=C2CC1C([NH]C)=O)=O`
**17a/17b**	`O=C([NH]C)CCC(N(C)C(C(C)C)C([NH]C)=O)=O`
**18a/18b**	`O=C([NH]C)CCC(NC(CC1=CC=NC=C1)C([NH]C)=O)=O`
**19a/19b**	`O=C([NH]C)CCC(NC(CC1=C(C=CC=C2)C2=CC=C1)C([NH]C)=O)=O`
**20a/20b**	`O=C(C(CC1=C(C=CC=C2)C2=CC=C1)NC(C(CC3=CC=NC=C3)NC(CCC([NH]C)=O)=O)=O)[NH]C`

Part B: Binding Data Analysis

Focal Molography Measurements

Binding measurements were performed using focal molography (FM) on a MACS Matchmaker instrument (Miltenyi Biotec, Germany). The instrument uses coherent detection of refractive index changes at the sensor surface to measure molecular binding in real-time. The signal output is the *coherent mass density* (expressed in pg/mm^2), which represents the surface mass density of molecules bound within the periodic molographic pattern. Unlike conventional SPR where all surface-bound molecules contribute to the signal, FM selectively detects only molecules that are coherently positioned within the diffractive grating structure, providing intrinsic background rejection (see Blickenstorfer *et al.*, *Phys. Rev. Appl.*, 2021, **15**, 034023).

Chip preparation. Molography chips (lino Biotech AG) were used for all measurements. Prior to each experiment, chips were regenerated by injection of 3 M guanidinium hydrochloride / 125 mM NaOH at 400 µL/min for 111 s (singleplex) or 52 s (multiplexed) to remove any previously bound material.

Ligand immobilization. DNA-directed immobilization was used to capture the DNA-VHL ligand conjugates on the chip surface. This approach eliminates the need for complex fluidic print heads or spotting procedures at the researcher's facility, as required by conventional SPR imaging platforms, enabling rapid compound loading and straightforward chip regeneration. For singleplex measurements, individual DNA-VHL ligand conjugates (200 nM) were immobilized by injection at 10 µL/min for approximately 650 s (total volume ∼100 µL). For multiplexed measurements, a mixture of 21 DNA-VHL ligand conjugates (20 compounds plus one complementary oligonucleotide without compound as negative control) was prepared with each compound at 200 nM concentration—the same concentration used in singleplex experiments—resulting in a total oligonucleotide concentration of 4.2 µM. This mixture was immobilized under identical flow conditions (10 µL/min for 600 s). The identical per-compound concentration ensures that capture strand saturation is comparable between singleplex and multiplexed formats.

Single-cycle kinetics. VHL-EloB-EloC complex (referred to as "VHL" or "Protein A") was injected at increasing concentrations without regenerations:

- Singleplex: 7 concentration cycles (0, 15.6, 31.3, 62.5, 125, 250, and 500 nM)
- Multiplexed: 7 concentration cycles (0, 15.6, 31.3, 62.5, 125, 250, and 500 nM)

Each VHL injection was performed at 30 µL/min for 120 s (injection volume: 60 µL), followed by a dissociation phase with buffer at 400 µL/min for approximately 220 s.

BSA negative control screen. To assess non-specific protein binding, bovine serum albumin (BSA) was injected across all 21 compound-loaded mologram (20 compounds in triplicate + 3 blank sensors) in a single-cycle kinetics format. Following chip regeneration (3 M guanidinium hydrochloride / 125 mM NaOH at 400 µL/min for 52 s) and immobilization of the oligo-compound mixture (10 µL/min for 600 s), BSA was injected at 12 increasing concentrations (0, 97.7 nM, 195.3 nM, 390.6 nM, 781.3 nM, 1.56, 3.13, 6.25, 12.5, 25, 50, and 100 µM) at 30 µL/min for 120 s per injection (injection volume: 60 µL), each followed by a dissociation phase with buffer at 200 µL/min for approximately 190 s.

Running buffer. All measurements were performed in PBS-T (137 mM NaCl, 2.7 mM KCl, 8 mM Na_2HPO_4, 2 mM KH_2PO_4, pH 7.4, Tween 0.05% v/v) at room temperature.

Figure SI-B1: Sensor Chip Layout for Multiplexed Measurements

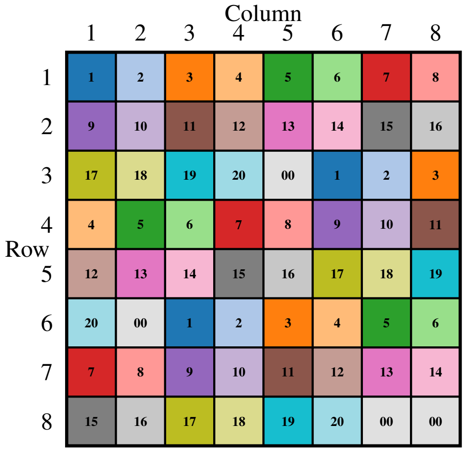

Figure SI-B1. Sensor chip layout for multiplexed 20-plex FM experiments. The 8 × 8 array (64 spots) contains 20 different DNA-PROTAC compounds (labeled **1**, **2b**–**20b**) and negative controls (**00**, containing only the complementary oligonucleotide without any compound attached). Each compound is represented by three replicate spots distributed across the chip. The chip is pre-functionalized with orthogonal 20-mer oligonucleotide capture sequences at each position. When a mixture of all 20 DNA-tagged compounds is injected, Watson–Crick base pairing automatically directs each compound to its designated sensing location within minutes. The 20-mer duplex provides quasi-covalent immobilization stability (T_m well above assay temperature), ensuring compounds remain stably bound throughout the single-cycle kinetics experiment.

Table SI-B1: Correlation Summary

Table SI-B1. Pearson correlation coefficients between normalized K_D and physicochemical properties across all four analysis methods. Significance levels: * $p < 0.05$, ** $p < 0.01$, *** $p < 0.001$, **** $p < 0.0001$.

Property	Singleplex		Multiplexed	
	Equilibrium	Kinetic	Equilibrium	Kinetic
**cLogP**	−0.59**	−0.48*	−0.84****	−0.82****
**cLogS**	+0.59**	+0.41	+0.79****	+0.78****
H-Acceptors	+0.19	+0.20	+0.58**	+0.58**
H-Donors	+0.13	+0.21	+0.33	+0.32
Total Surface Area	−0.19	−0.12	+0.10	+0.10
**Relative PSA**	+0.49*	+0.42	+0.61**	+0.58**
Polar Surface Area	+0.18	+0.23	+0.54*	+0.53*

Strong predictors of binding affinity (cLogP, cLogS, Relative PSA) are shown in bold. The multiplexed format consistently reveals stronger correlations than singleplex, supporting its utility for structure–activity relationship studies.

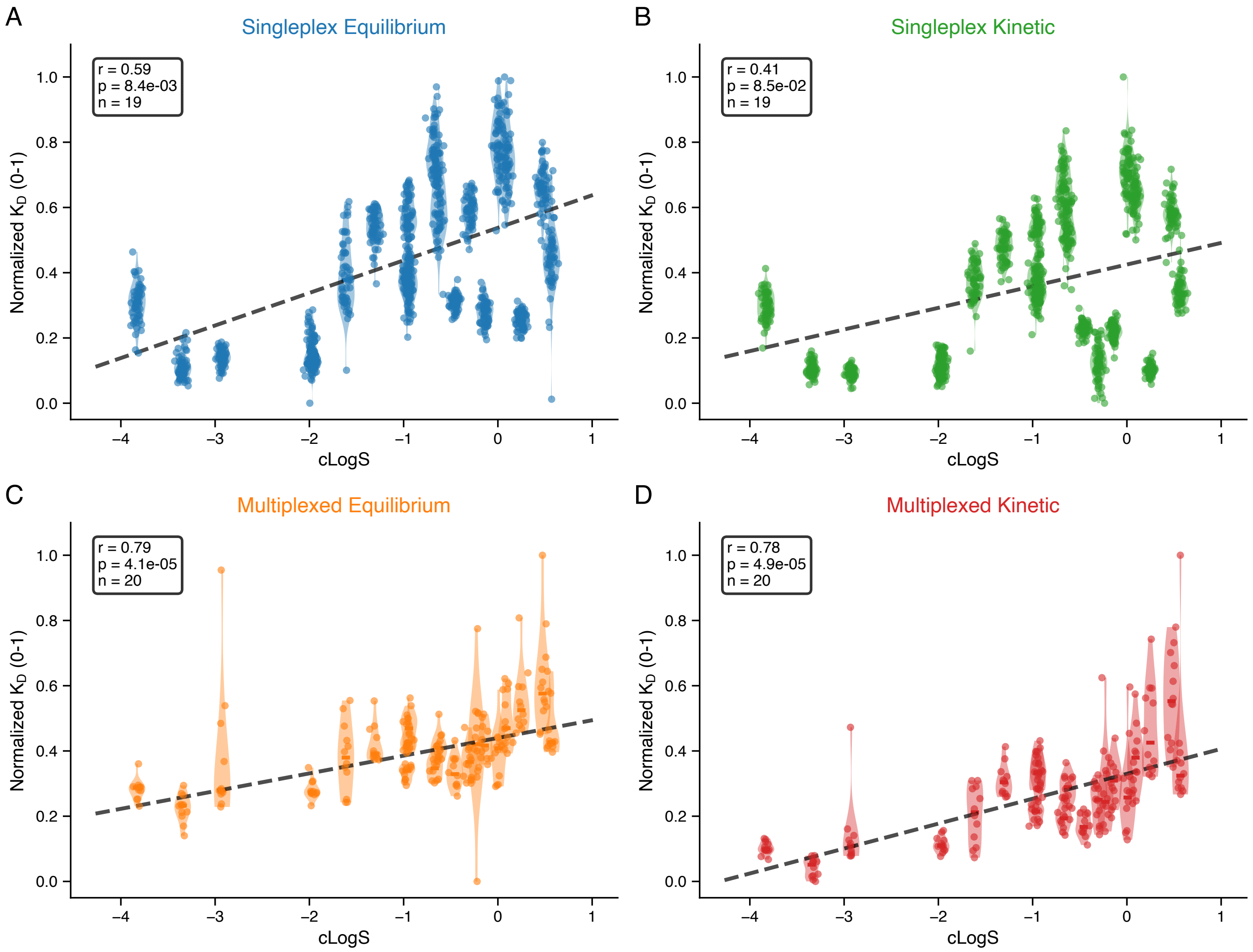

Figure SI-B2. K_D correlation with solubility (cLogS). Violin plots showing normalized K_D (0–1 scale) versus calculated aqueous solubility (cLogS) for (A) singleplex equilibrium, (B) singleplex kinetic, (C) multiplexed equilibrium, and (D) multiplexed kinetic methods. Strong positive correlations indicate that more soluble compounds (higher cLogS, less negative values) exhibit weaker binding (higher K_D). This inverse relationship with cLogP is expected, as cLogS $\approx -$cLogP for most compounds. Multiplexed methods show stronger correlations ($r = 0.78$–0.79) compared to singleplex ($r = 0.41$–0.59).

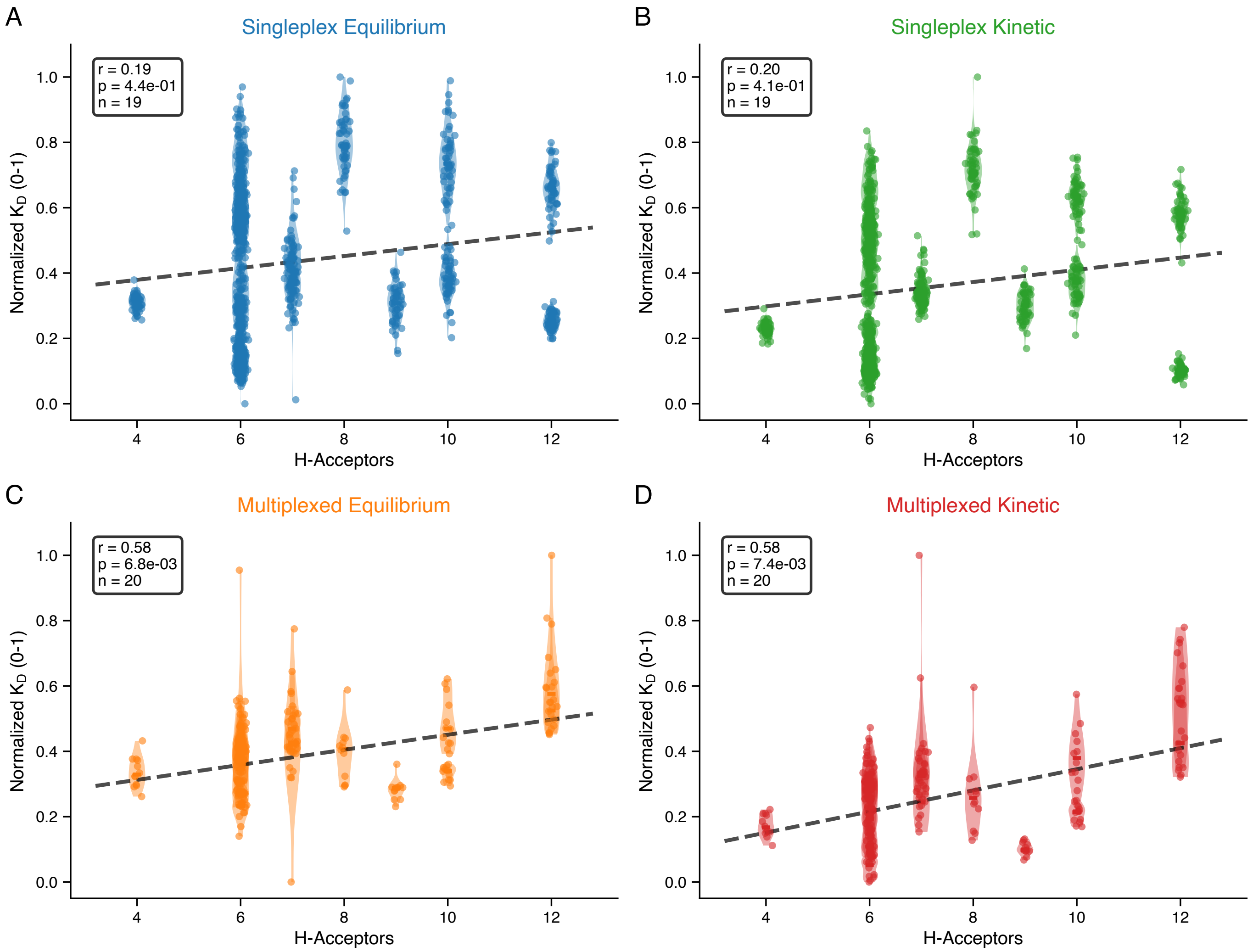

Figure SI-B3. K_D correlation with hydrogen bond acceptors. Violin plots showing normalized K_D versus the number of hydrogen bond acceptor atoms for each analysis method. Weak to moderate positive correlations are observed only in the multiplexed format ($r \approx 0.58$, $p < 0.01$), while singleplex methods show no significant correlation ($p > 0.4$). This suggests that hydrogen bond acceptor count has a modest effect on binding affinity, with compounds having more acceptors tending toward slightly weaker binding.

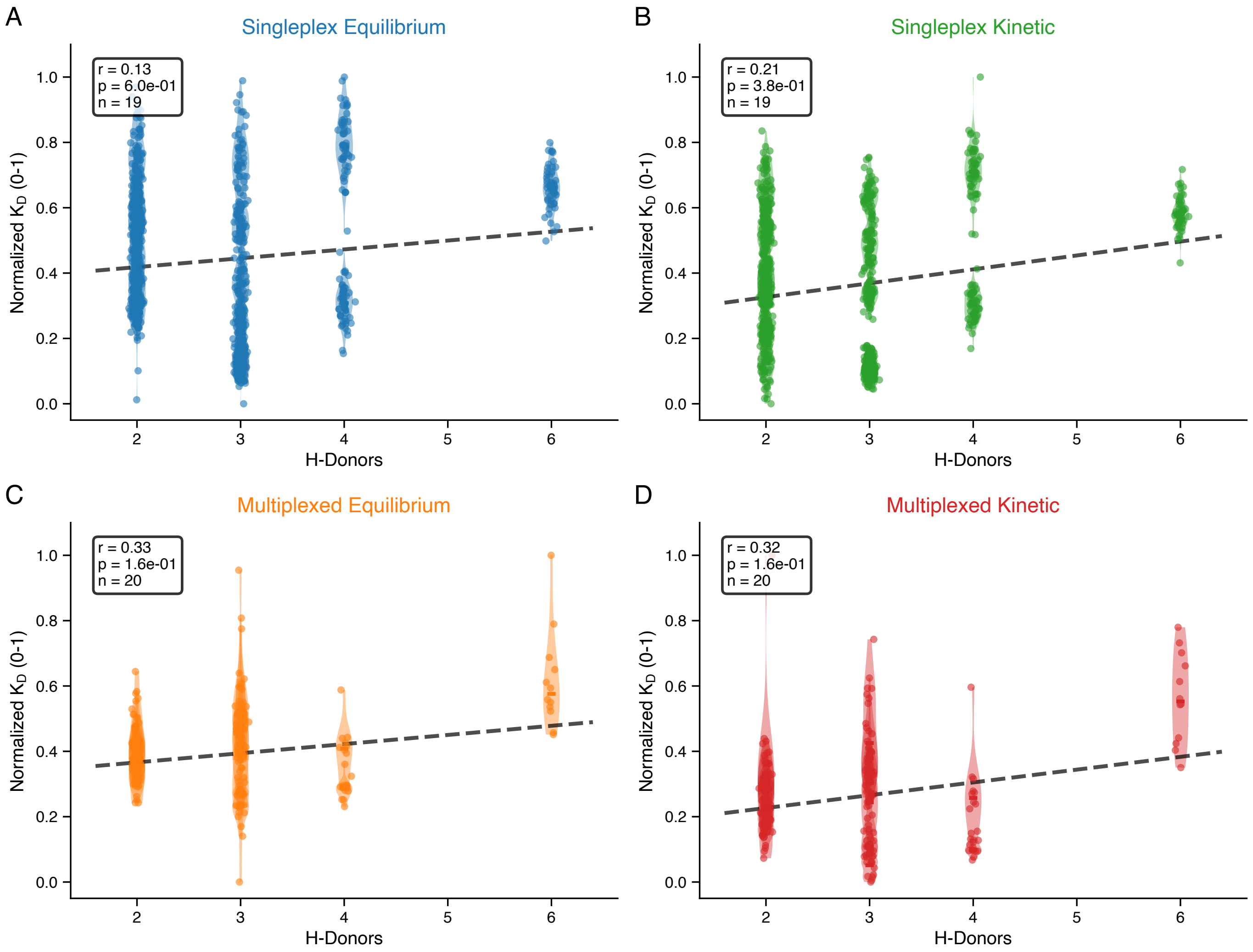

Figure SI-B4. K_D correlation with hydrogen bond donors. Violin plots showing normalized K_D versus the number of hydrogen bond donor atoms. No significant correlation is observed in any of the four analysis methods ($r = 0.13$–0.33, $p > 0.15$). The limited range of H-donors across the compound series (most compounds have 2–4 donors) may limit the ability to detect trends. These results indicate that the number of hydrogen bond donors is not a strong predictor of linker–VHL binding affinity.

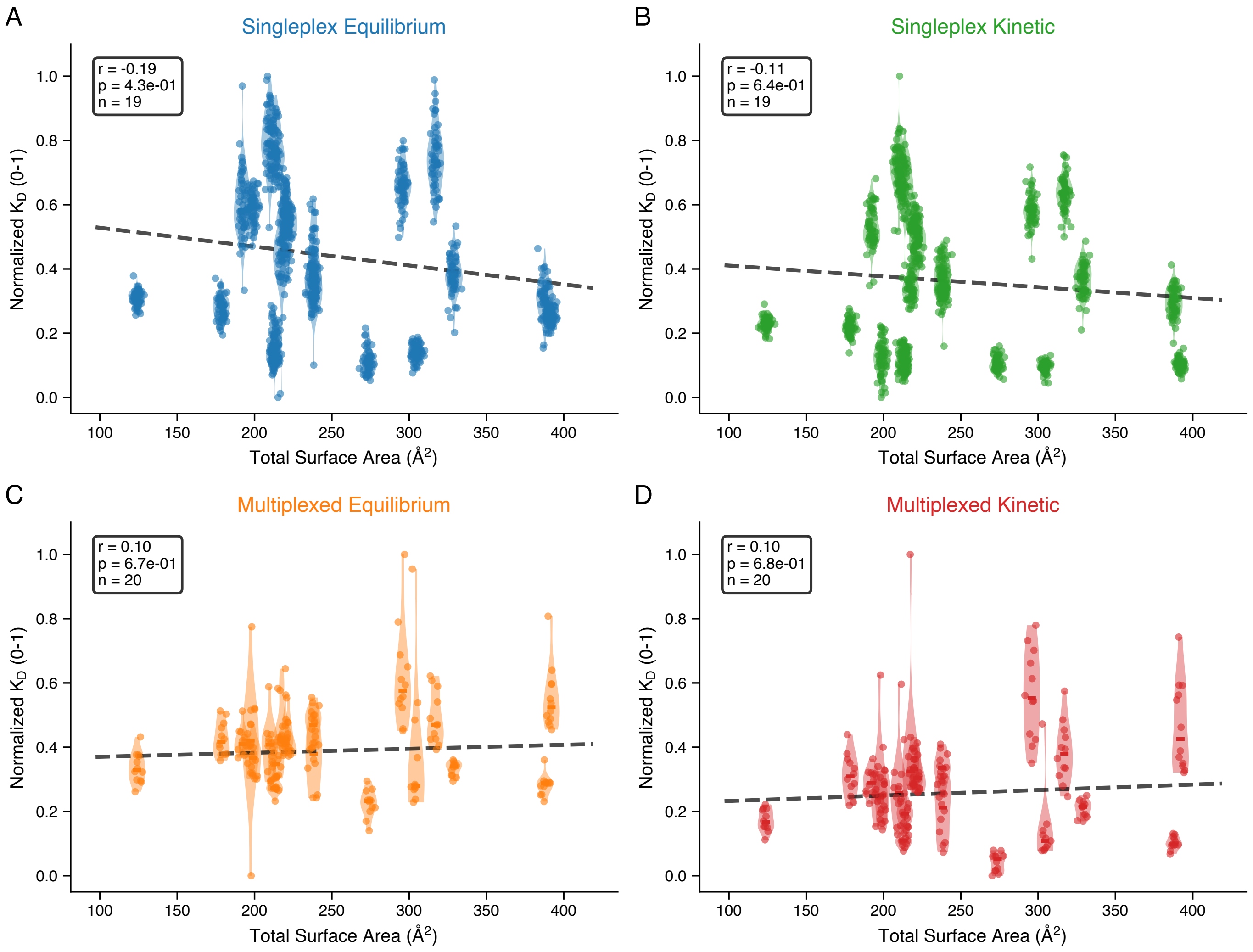

Figure SI-B5. K_{D} correlation with total surface area. Violin plots showing normalized K_{D} versus calculated total molecular surface area (Å^2). No significant correlation is observed in any analysis method ($|r| < 0.2$, $p > 0.4$). This indicates that overall molecular size does not predict binding affinity in this compound series, suggesting that binding is driven by specific interactions (hydrophobicity, shape complementarity) rather than molecular bulk.

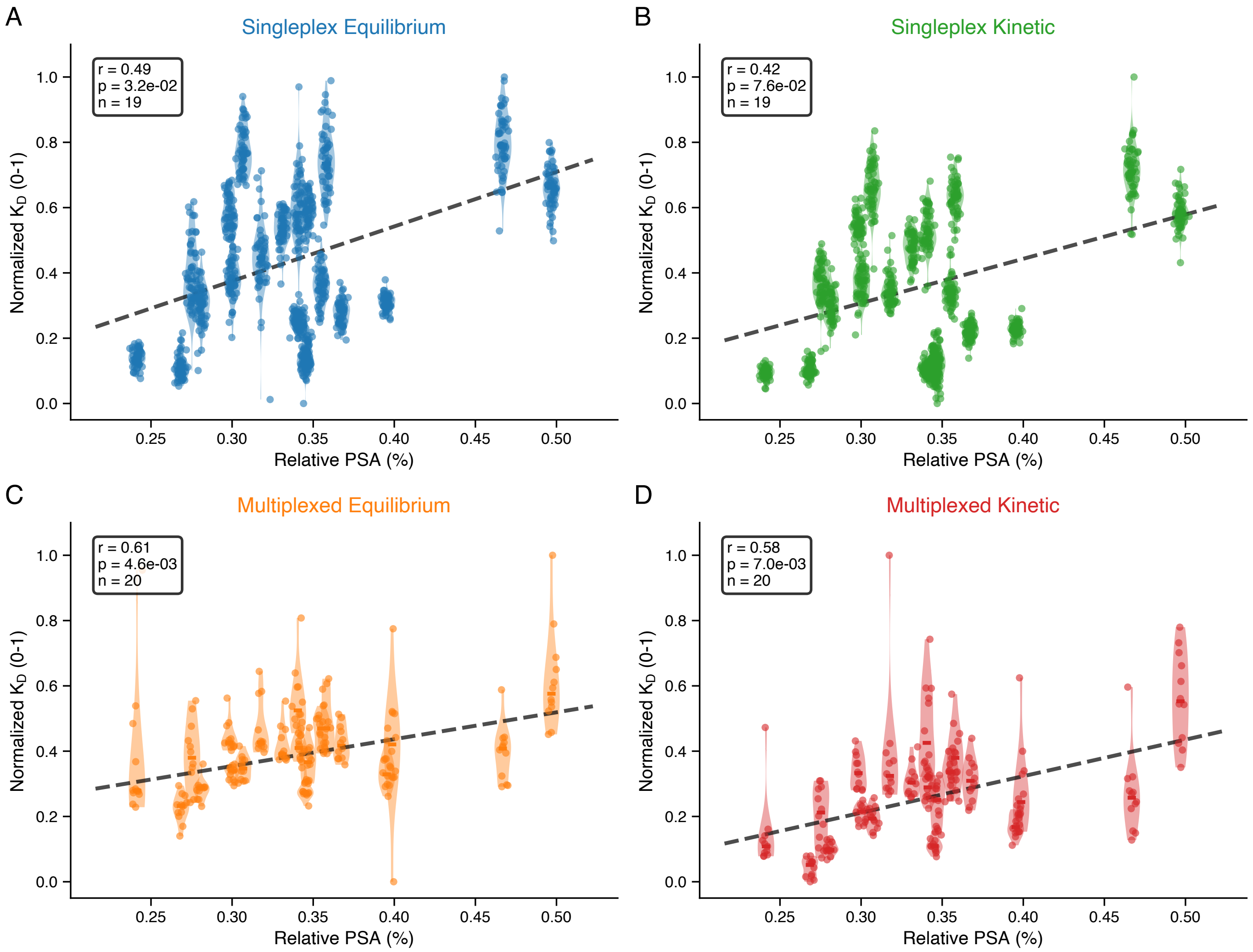

Figure SI-B6. K_D correlation with relative polar surface area. Violin plots showing normalized K_D versus the fraction of molecular surface that is polar (Relative PSA = PSA / Total Surface Area). Moderate positive correlations are observed across methods: singleplex equilibrium ($r = 0.49$, $p = 0.032$), multiplexed methods ($r = 0.58$–0.61, $p < 0.01$). Compounds with a higher polar surface fraction exhibit weaker binding, consistent with the importance of hydrophobic interactions for linker–VHL binding.

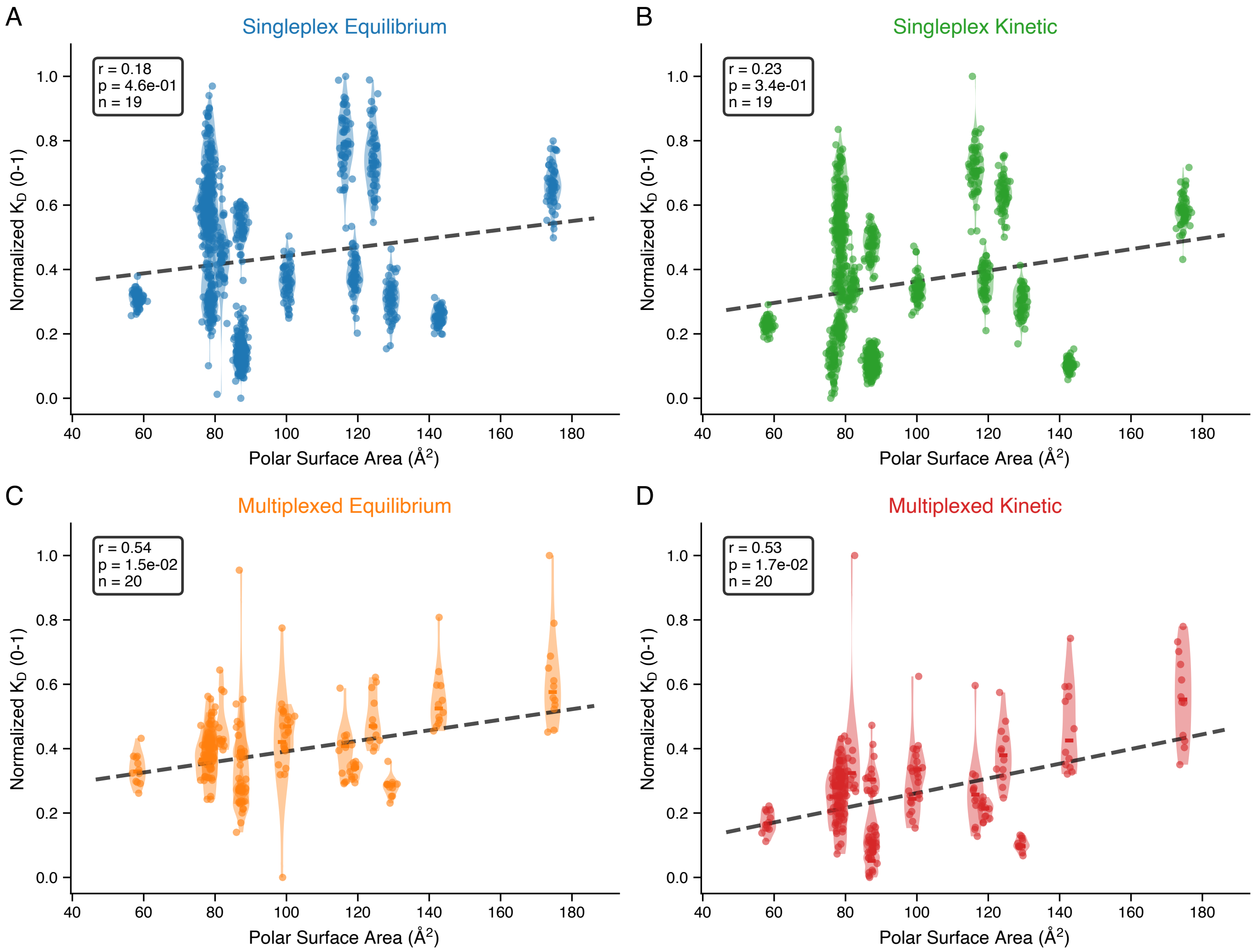

Figure SI-B7. K_D correlation with polar surface area. Violin plots showing normalized K_D versus absolute polar surface area (PSA, Å^2). Weak to moderate positive correlations are significant only in the multiplexed format ($r \approx 0.53$, $p < 0.02$), while singleplex methods show no significant correlation. Notably, absolute PSA is less predictive than relative PSA (Figure SI-B6), indicating that the *fraction* of polar surface rather than the absolute polar area determines binding strength.

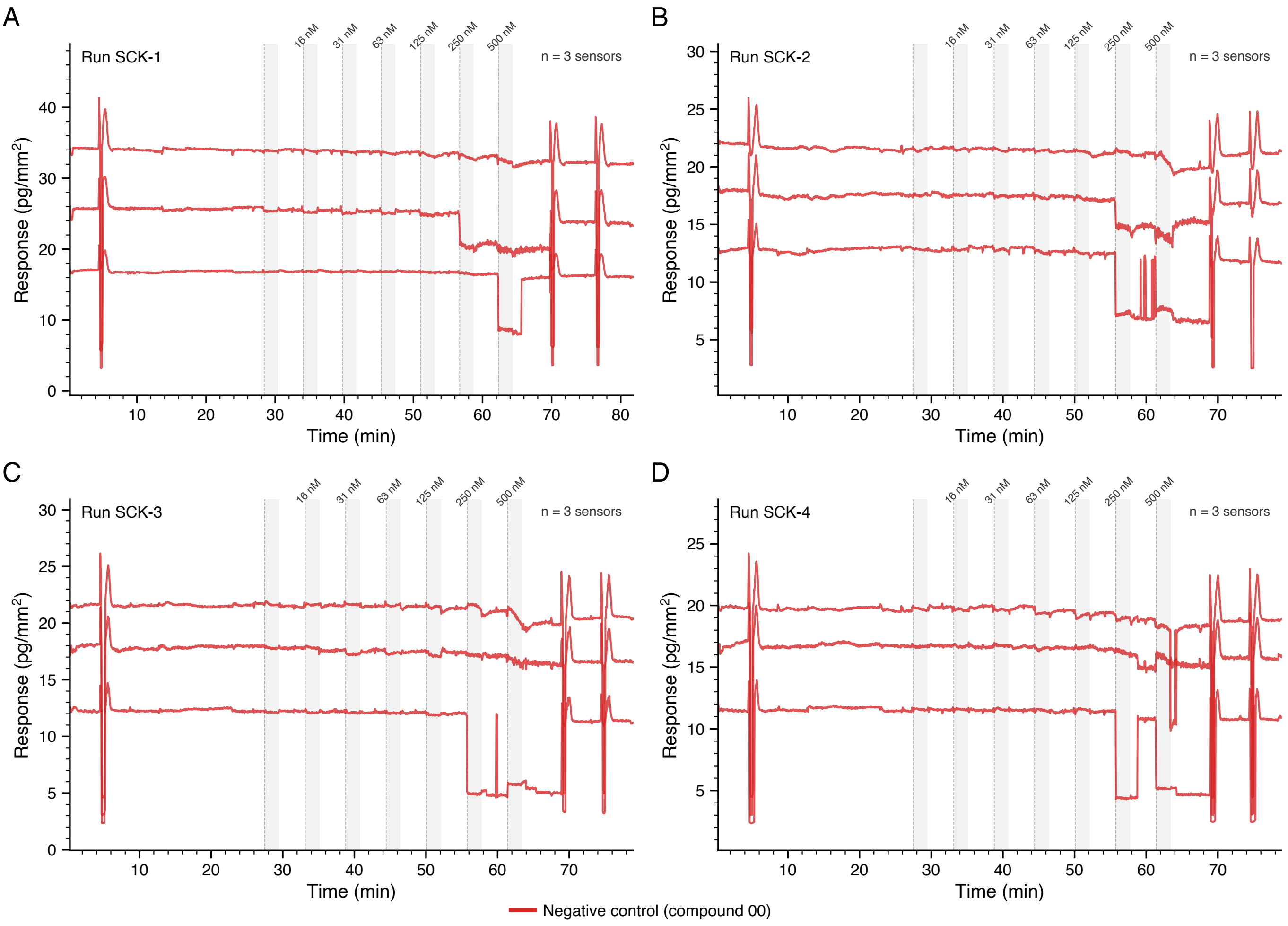

Figure SI-B8. Blank control sensorgrams from multiplexed experiments. Raw FM traces for the blank mologram position, which contains a single-stranded oligonucleotide capture sequence in the ridges but no complementary DNA-ligand conjugate in the compound pool. Data shown for four independent runs: (A) SCK-1, (B) SCK-2, (C) SCK-3, and (D) SCK-4. Each panel shows 3 sensor traces. Gray shading indicates analyte (VHL protein) injection phases with concentrations labeled. Notably, no signal increase is observed during the immobilization phase (no DNA-ligand conjugate hybridizes to the blank position), and no response is observed during subsequent VHL injections. This demonstrates a key advantage of FM: the technique selectively detects only ordered molecules on the diffractive pattern, while refractive index changes of the bulk medium and non-specific binding to the surface do not contribute to the coherent diffraction signal. The spikes observed at 250–500 nM VHL concentrations are software artifacts caused by automatic camera exposure optimization when other molograms on the chip become very bright at high analyte concentrations.

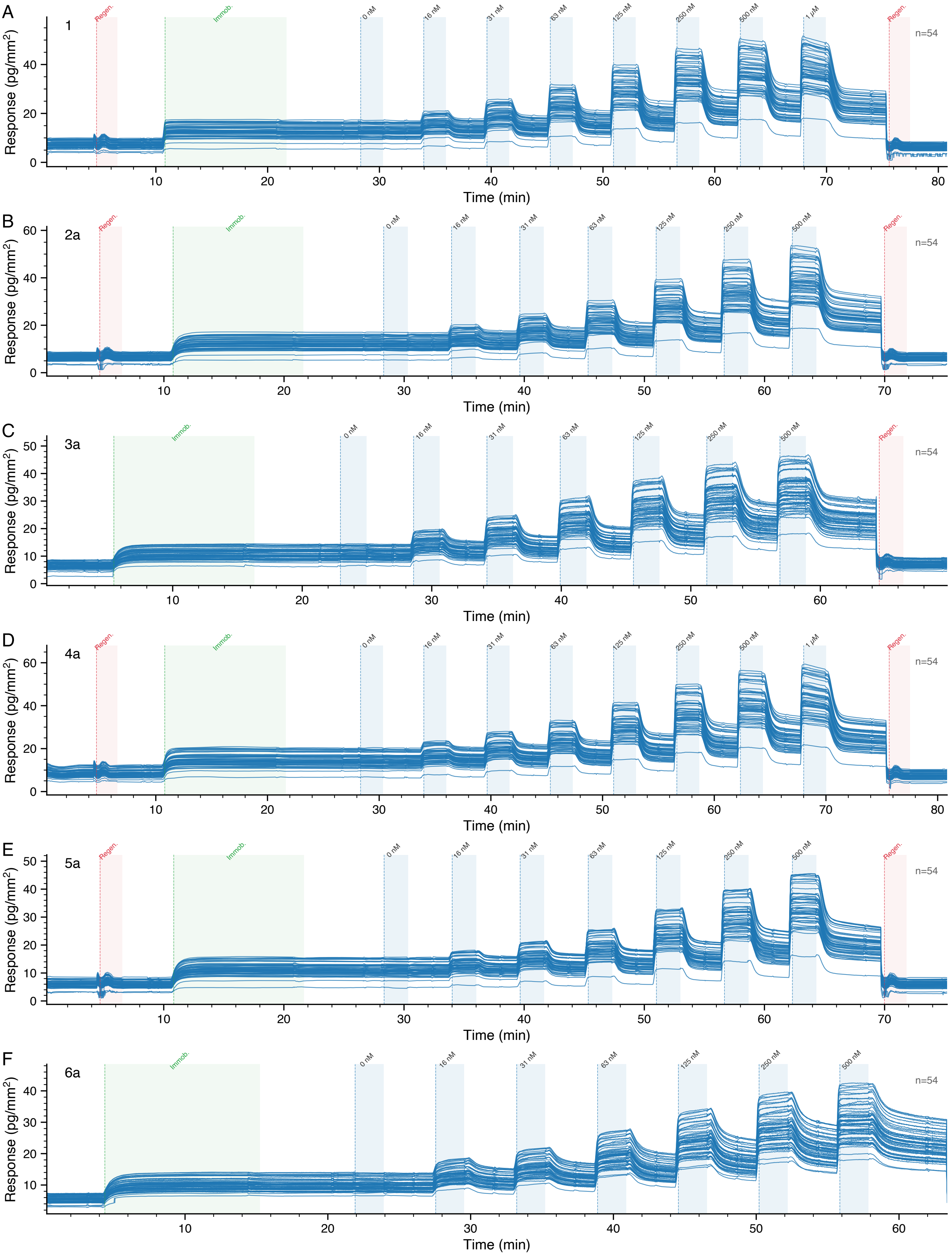

Figure SI-B9. Raw sensorgrams from singleplex experiments (compounds 1–6a). Full FM traces showing the complete experimental timeline including immobilization (green shading), analyte injections at increasing VHL concentrations (blue shading with concentrations labeled), and regeneration phases (red shading). Each panel shows all sensor replicates for one compound. The response (pg/mm^2) reflects the coherent mass density change upon molecular binding. (A) Compound 1. (B) Compound 2a. (C) Compound 3a. (D) Compound 4a. (E) Compound 5a. (F) Compound 6a.

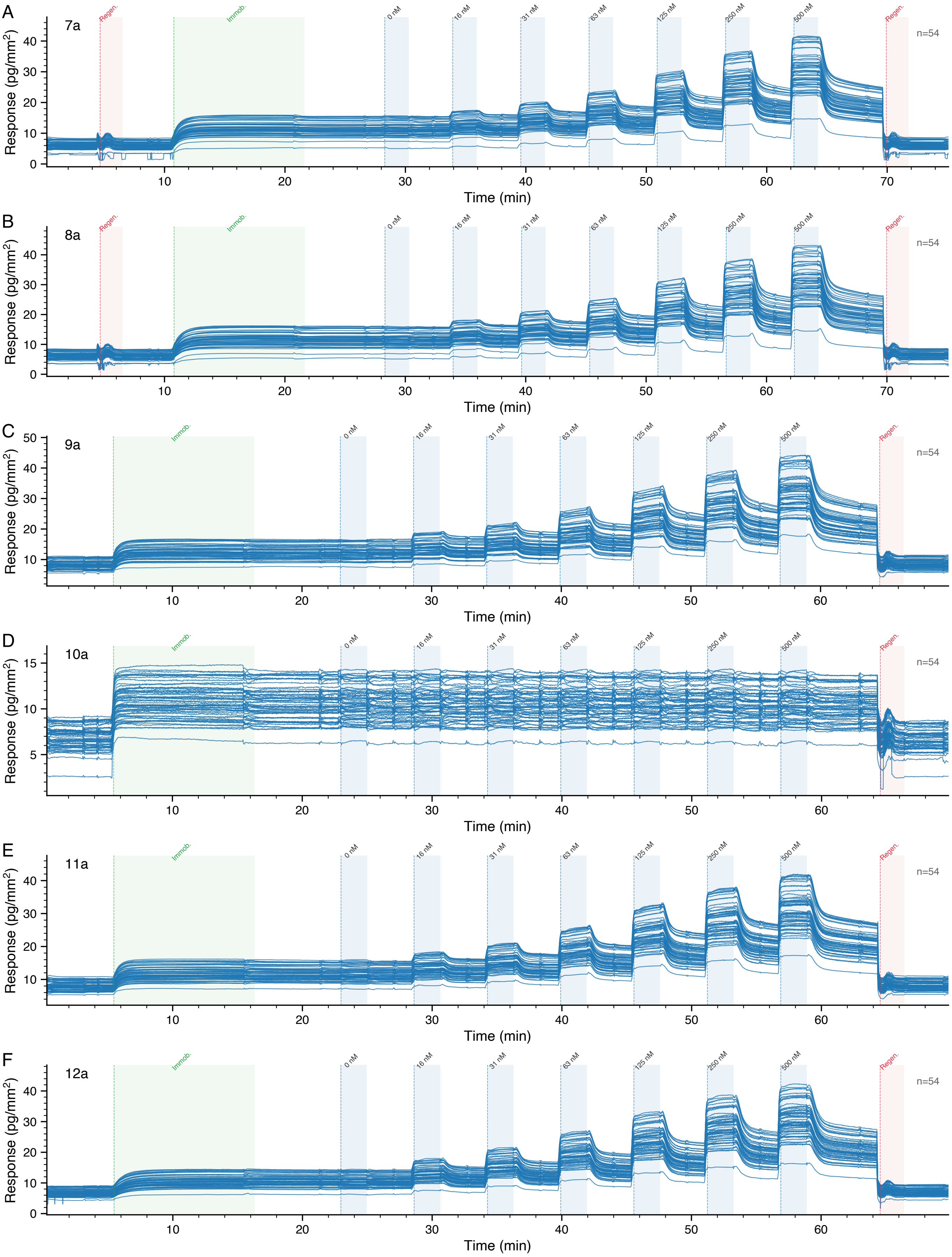

Figure SI-B10. Raw sensorgrams from singleplex experiments (compounds 7a–12a). Full FM traces showing the complete experimental timeline. (A) Compound 7a. (B) Compound 8a. (C) Compound 9a. (D) Compound 10a. (E) Compound 11a. (F) Compound 12a.

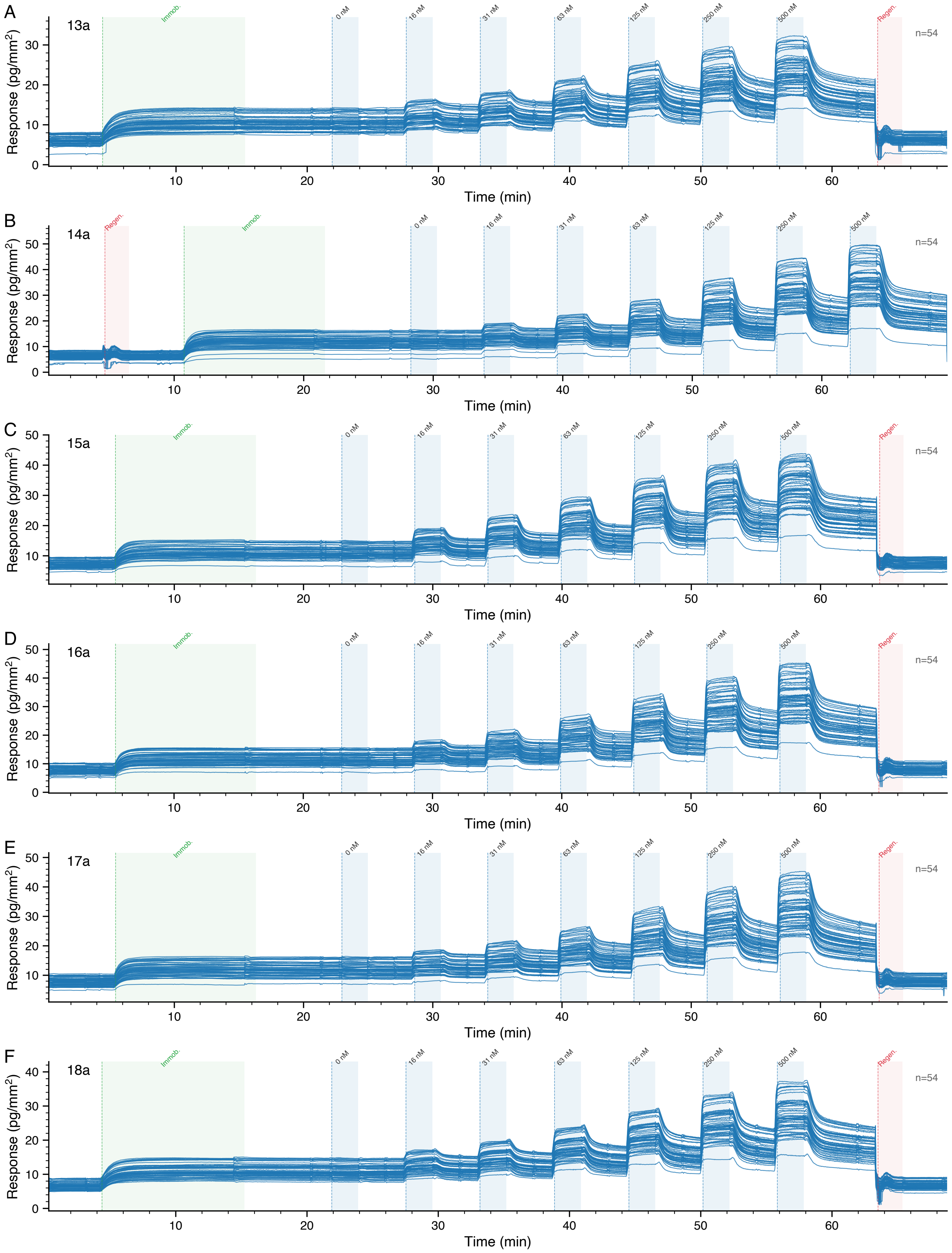

Figure SI-B11. Raw sensorgrams from singleplex experiments (compounds 13a–18a). Full FM traces showing the complete experimental timeline. (A) Compound 13a. (B) Compound 14a. (C) Compound 15a. (D) Compound 16a. (E) Compound 17a. (F) Compound 18a.

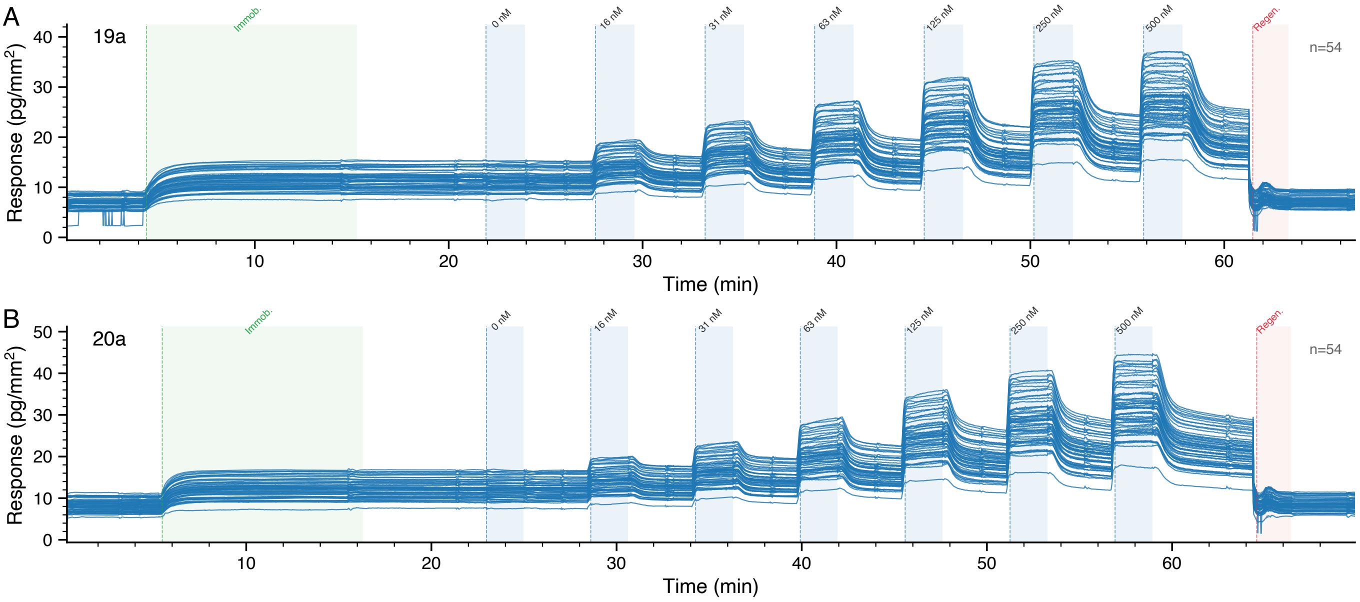

Figure SI-B12. Raw sensorgrams from singleplex experiments (compounds 19a–20a). Full FM traces showing the complete experimental timeline. (A) Compound 19a. (B) Compound 20a.

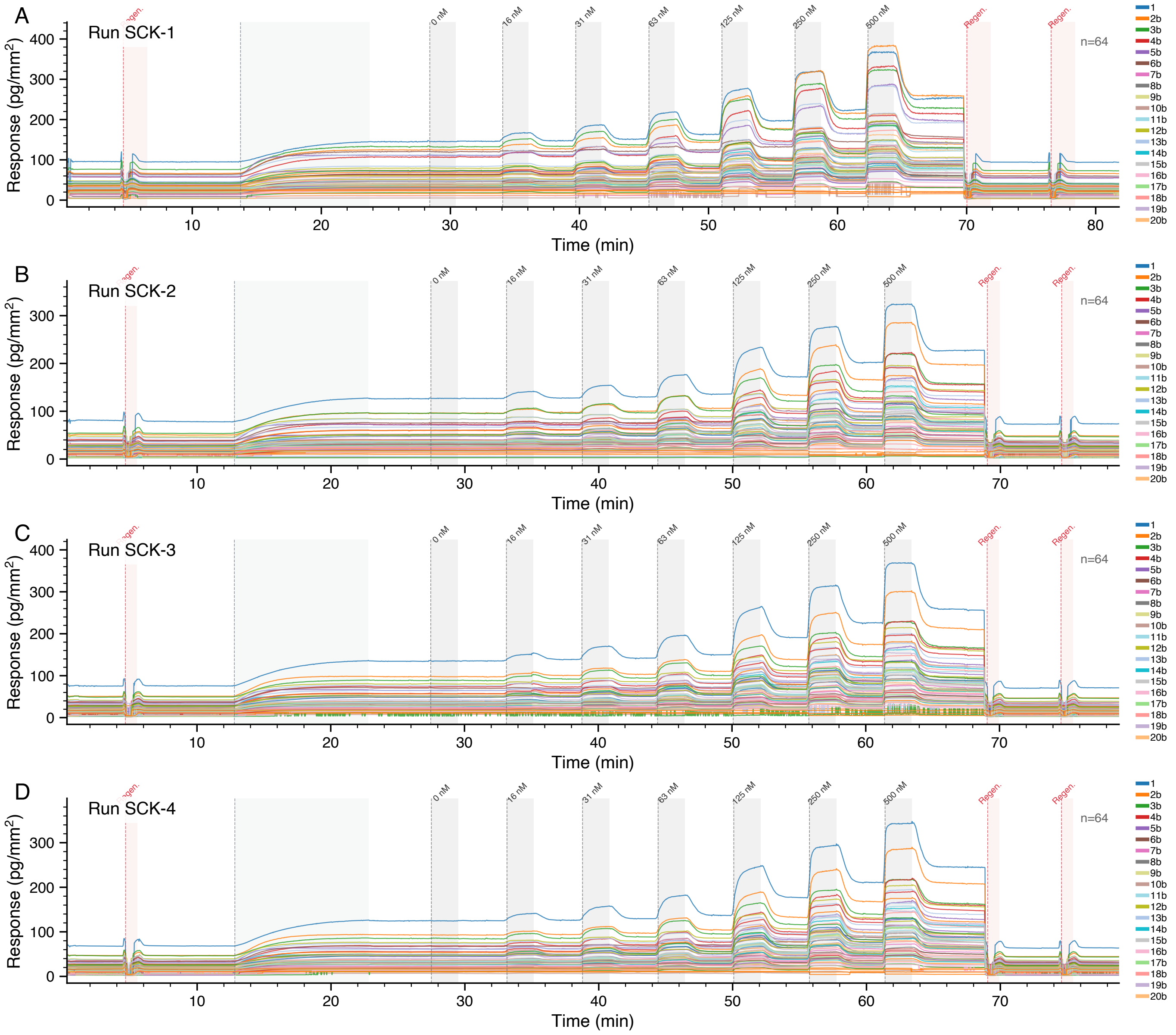

Figure SI-B13. Raw sensorgrams from multiplexed 20-plex experiments. Full FM traces for four independent runs on the same chip, each containing 20 DNA-PROTAC compounds measured simultaneously. Traces are colored by compound identity (see legend). Gray shading indicates injection phases. The multiplexed format enables direct comparison of binding kinetics across all compounds under identical experimental conditions, with four replicate runs demonstrating run-to-run reproducibility. (A) Run SCK-1. (B) Run SCK-2. (C) Run SCK-3. (D) Run SCK-4.

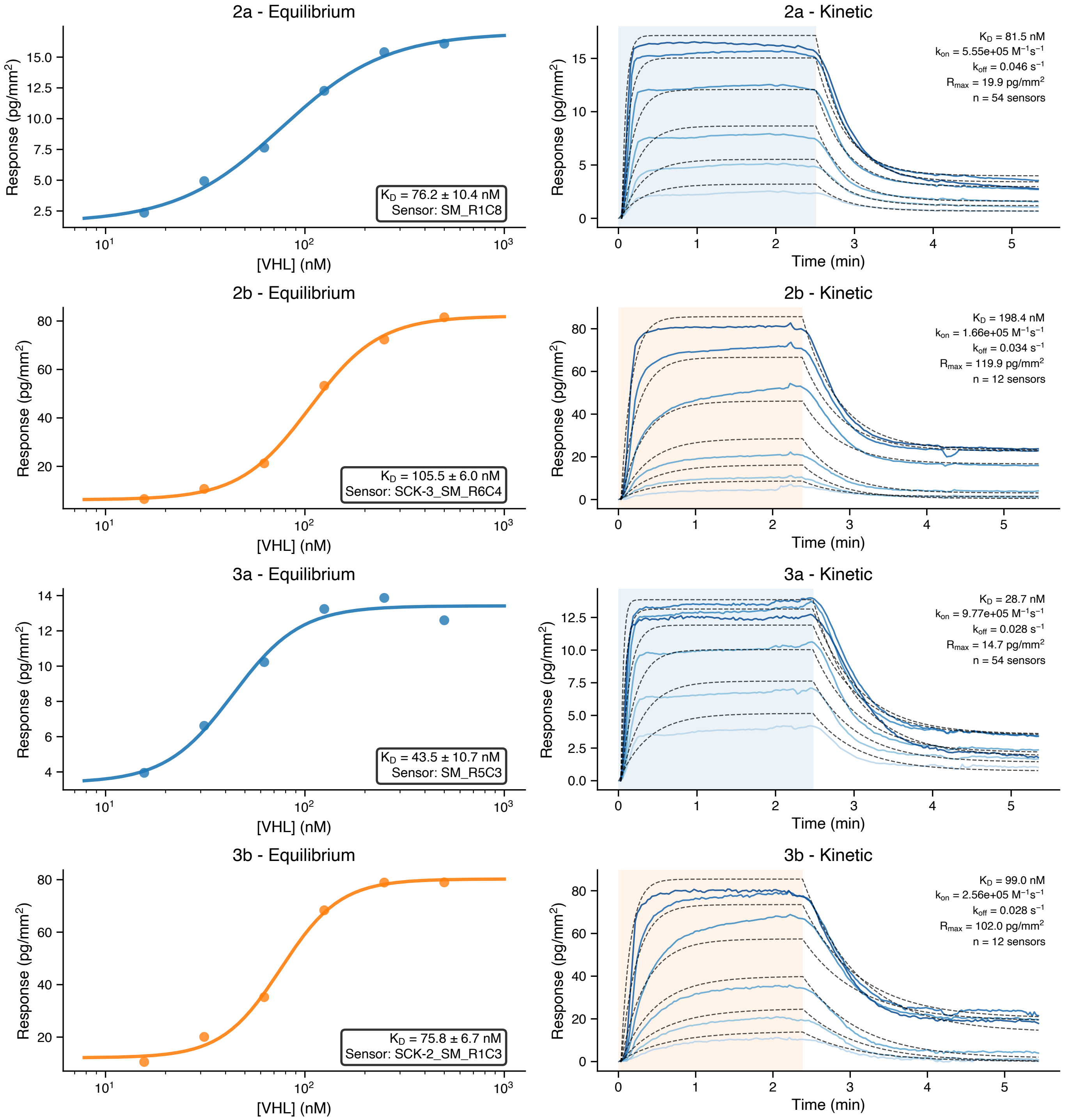

Figure SI-B14. Equilibrium and kinetic binding fits for compounds **2** and **3**. For each compound, the top row shows the singleplex variant (a) and the bottom row shows the multiplexed variant (b). Left panels: Equilibrium binding isotherms with Hill equation fits. Right panels: Multi-cycle kinetic sensorgrams with fitted curves (dashed lines). VHL concentrations used: 0, 15.6, 31.3, 62.5, 125, 250, and 500 nM. Blue shading indicates the association phase.

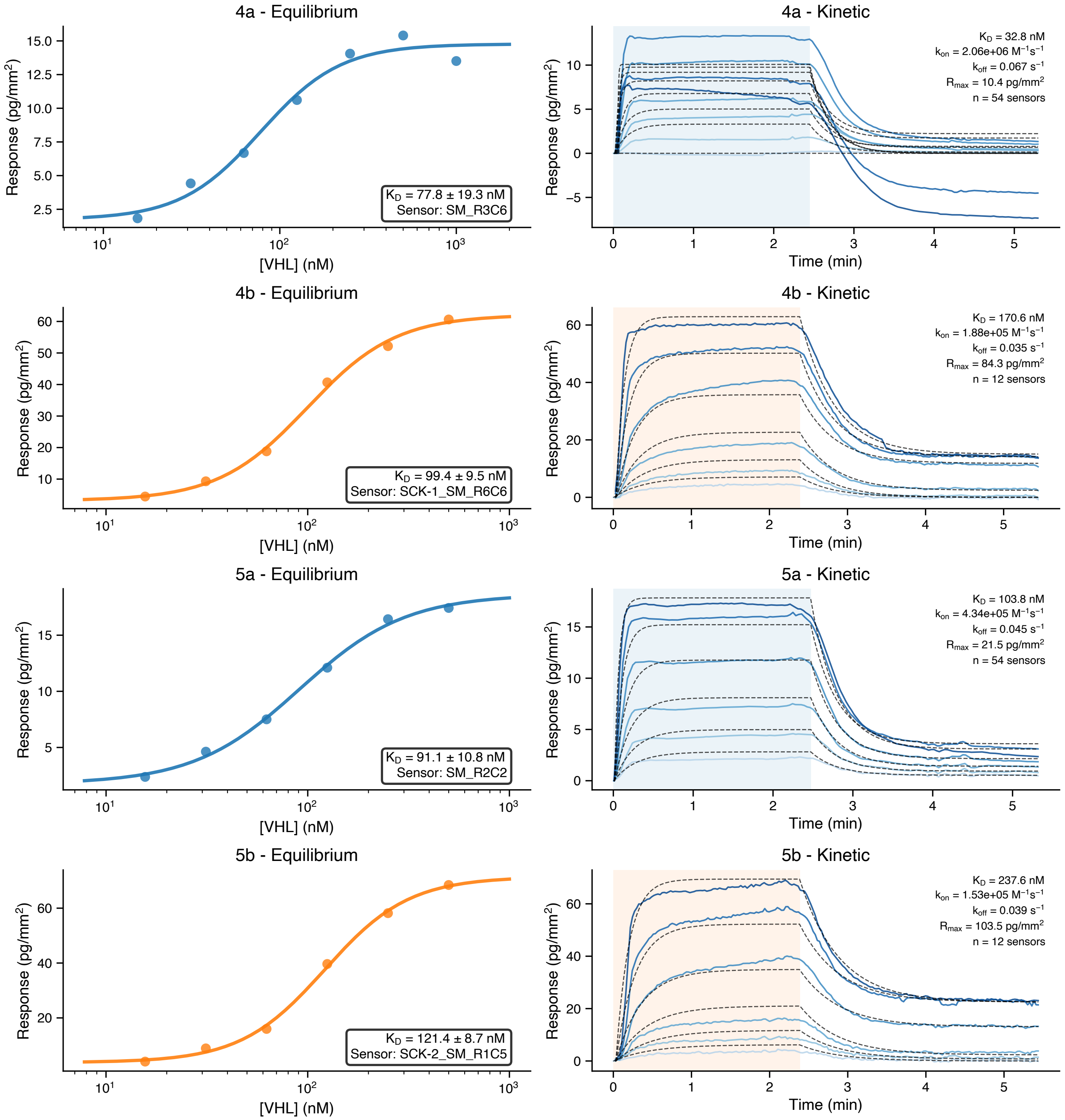

Figure SI-B15. Equilibrium and kinetic binding fits for compounds **4** and **5**. For each compound, the top row shows the singleplex variant (a) and the bottom row shows the multiplexed variant (b). Left panels: Equilibrium binding isotherms with Hill equation fits. Right panels: Multi-cycle kinetic sensorgrams with fitted curves (dashed lines). VHL concentrations used: 0, 15.6, 31.3, 62.5, 125, 250, and 500 nM. Blue shading indicates the association phase.

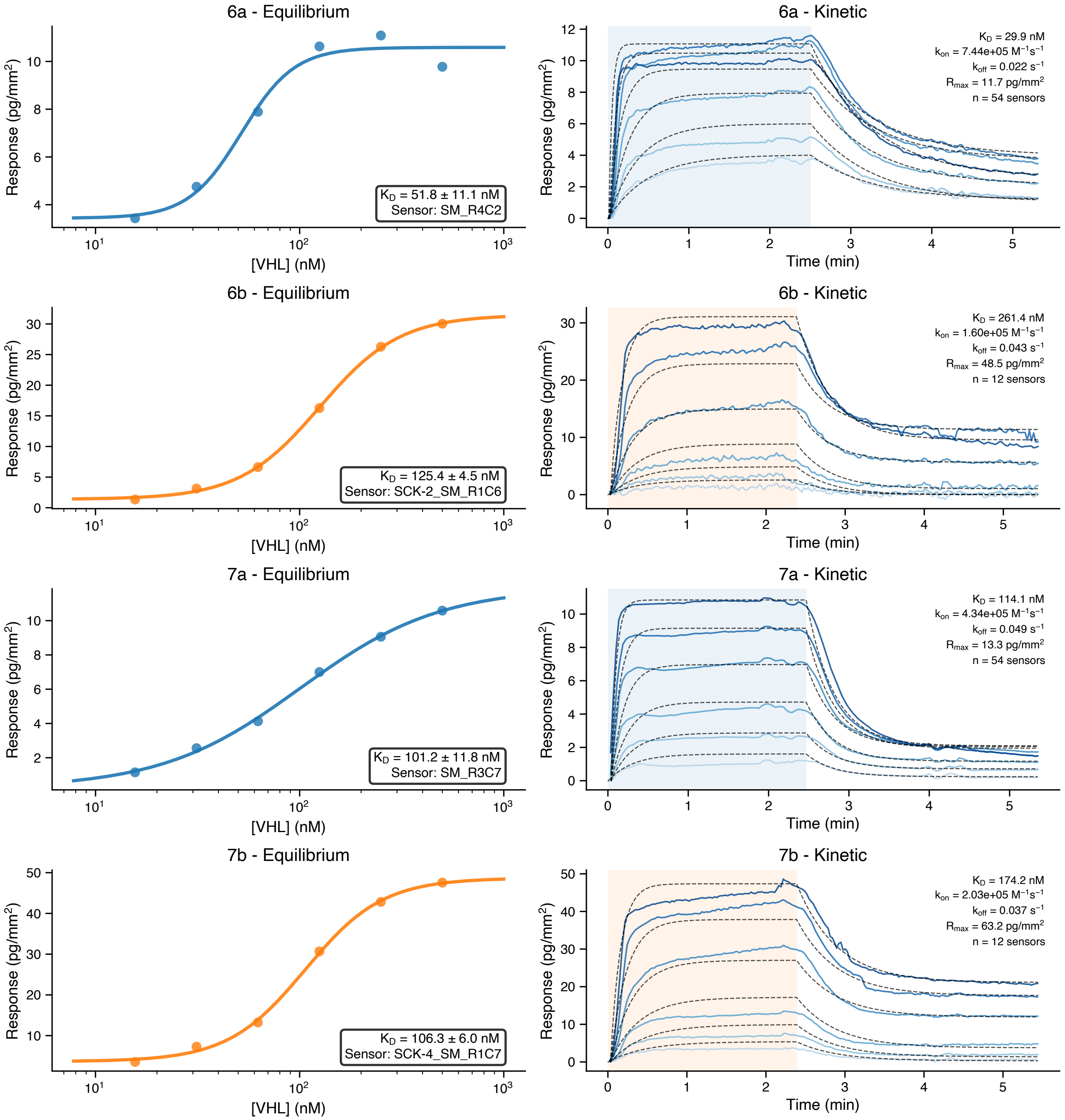

Figure SI-B16. Equilibrium and kinetic binding fits for compounds **6** and **7**. For each compound, the top row shows the singleplex variant (a) and the bottom row shows the multiplexed variant (b). Left panels: Equilibrium binding isotherms with Hill equation fits. Right panels: Multi-cycle kinetic sensorgrams with fitted curves (dashed lines). VHL concentrations used: 0, 15.6, 31.3, 62.5, 125, 250, and 500 nM. Blue shading indicates the association phase.

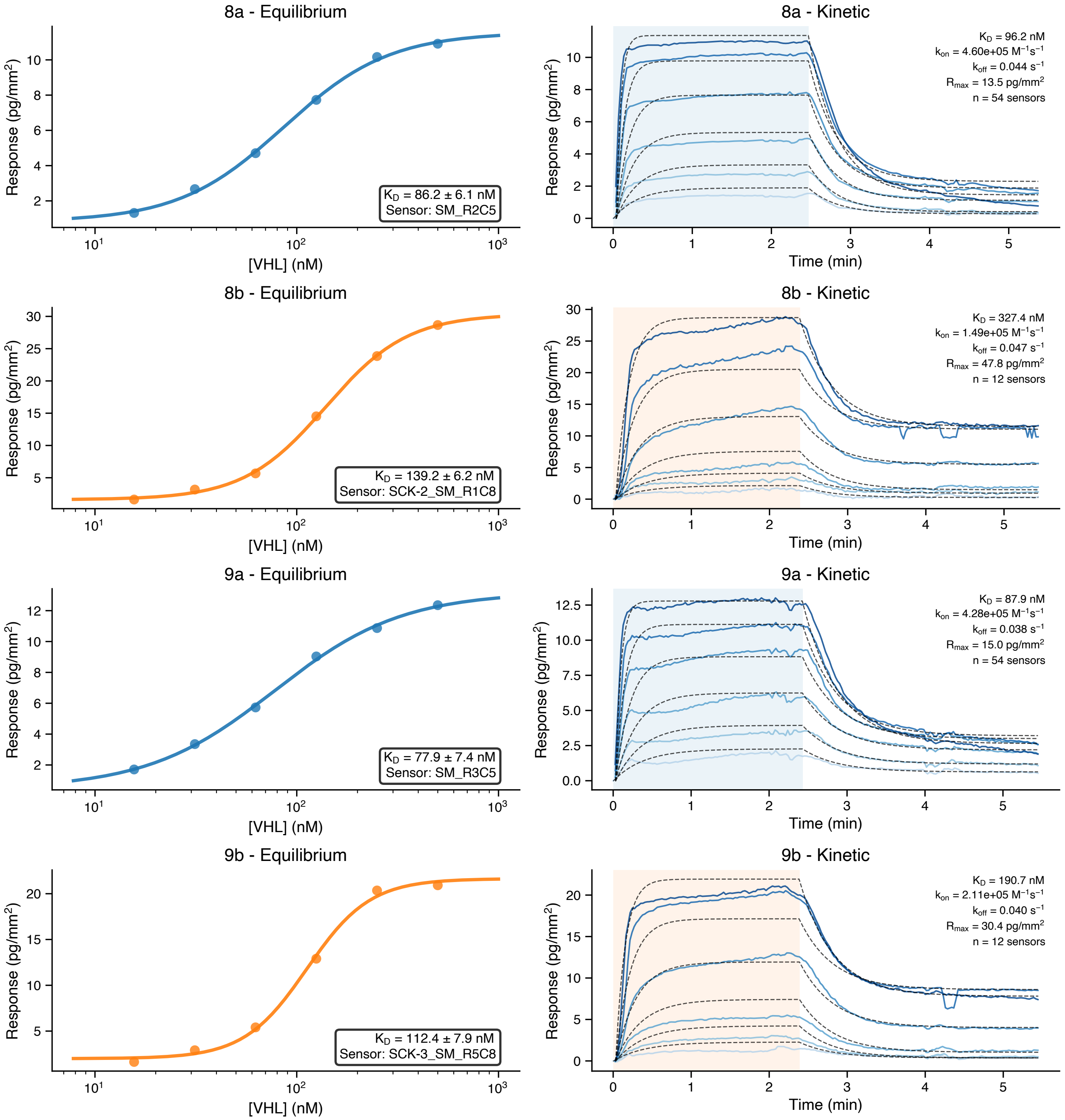

Figure SI-B17. Equilibrium and kinetic binding fits for compounds **8** and **9**. For each compound, the top row shows the singleplex variant (a) and the bottom row shows the multiplexed variant (b). Left panels: Equilibrium binding isotherms with Hill equation fits. Right panels: Multi-cycle kinetic sensorgrams with fitted curves (dashed lines). VHL concentrations used: 0, 15.6, 31.3, 62.5, 125, 250, and 500 nM. Blue shading indicates the association phase.

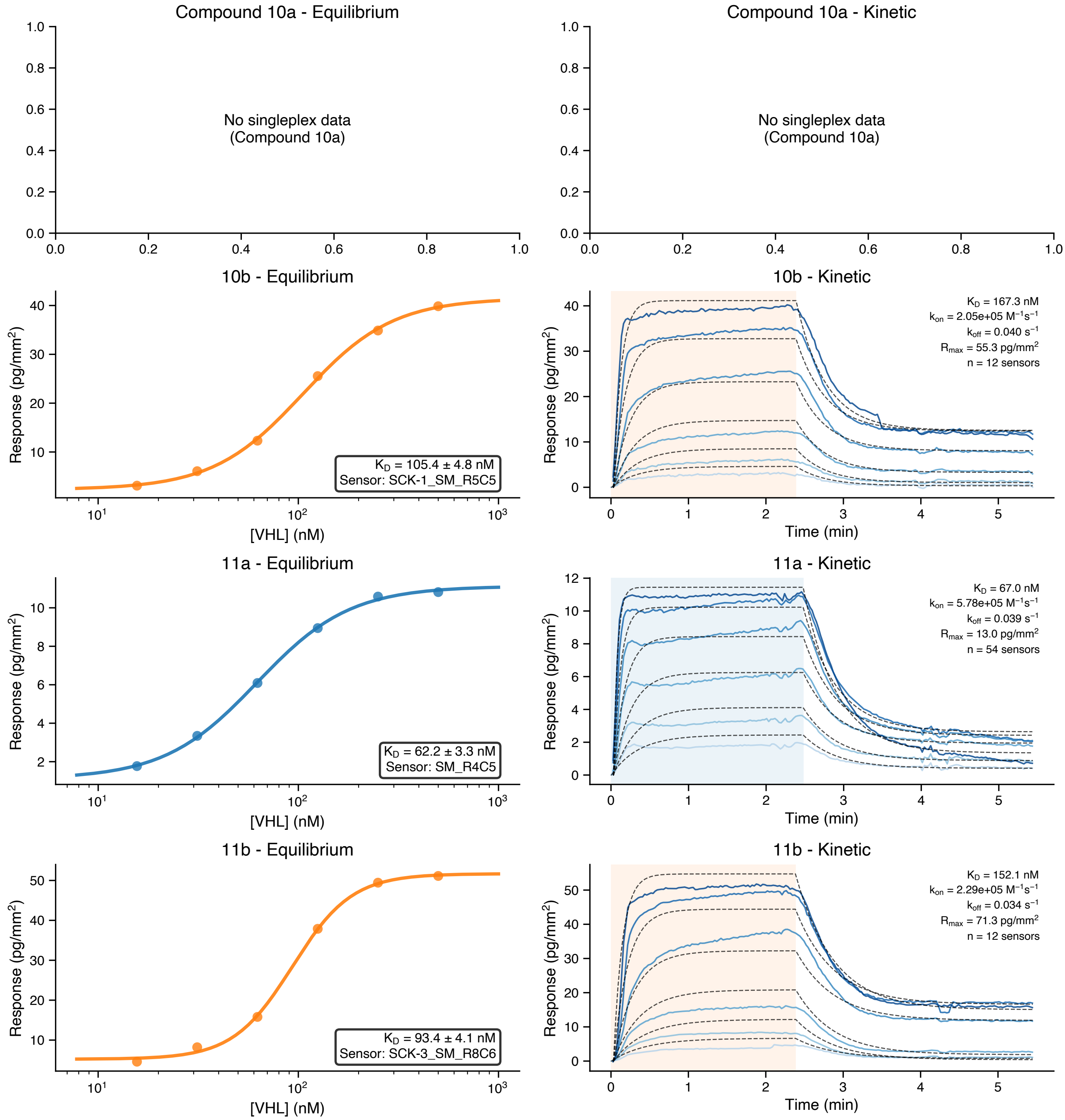

Figure SI-B18. Equilibrium and kinetic binding fits for compounds **10** and **11**. For each compound, the top row shows the singleplex variant (a) and the bottom row shows the multiplexed variant (b). Left panels: Equilibrium binding isotherms with Hill equation fits. Right panels: Multi-cycle kinetic sensorgrams with fitted curves (dashed lines). VHL concentrations used: 0, 15.6, 31.3, 62.5, 125, 250, and 500 nM. Blue shading indicates the association phase. Note: Compound 10a was not measured in singleplex format.

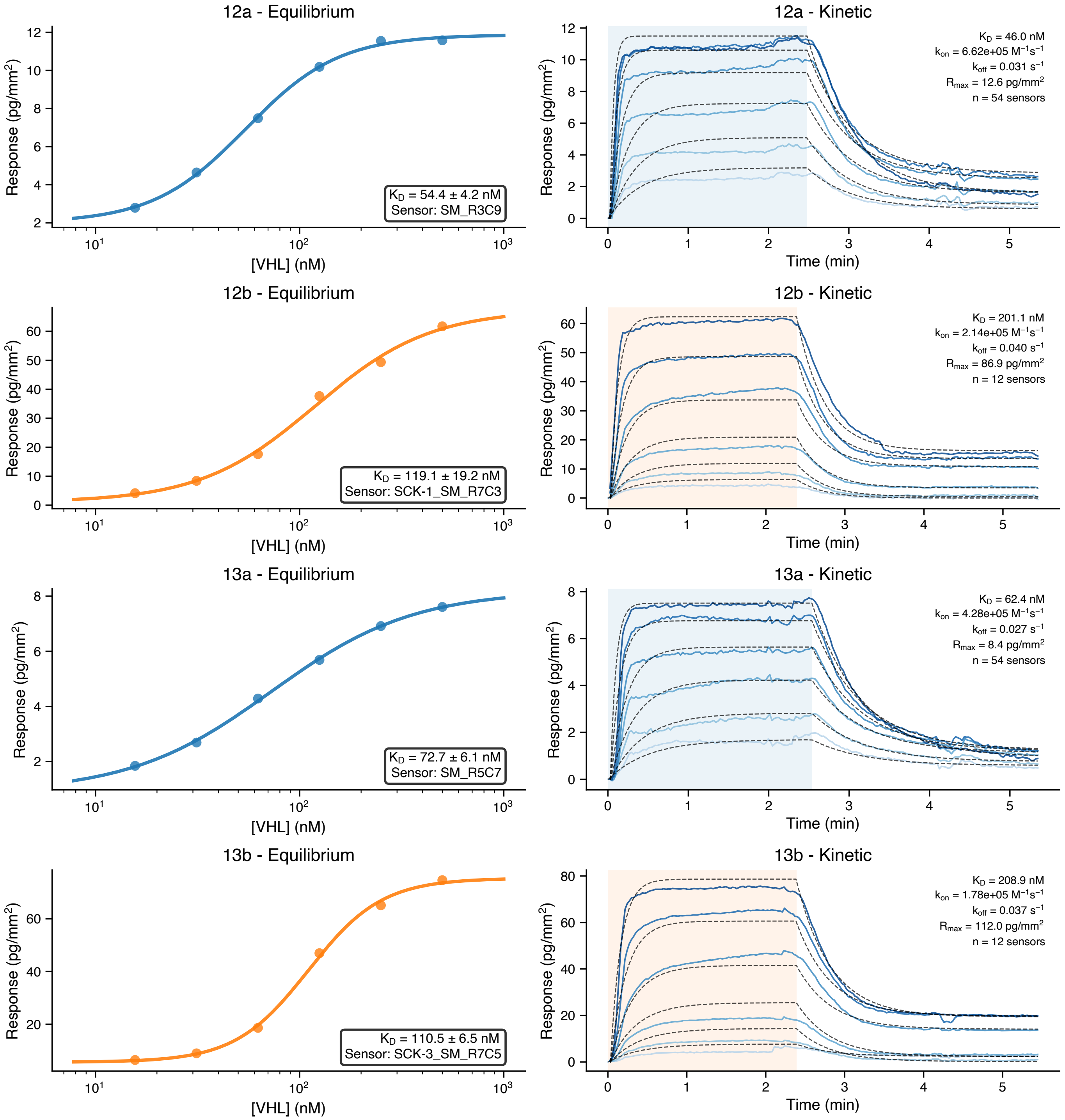

Figure SI-B19. Equilibrium and kinetic binding fits for compounds **12** and **13**. For each compound, the top row shows the singleplex variant (a) and the bottom row shows the multiplexed variant (b). Left panels: Equilibrium binding isotherms with Hill equation fits. Right panels: Multi-cycle kinetic sensorgrams with fitted curves (dashed lines). VHL concentrations used: 0, 15.6, 31.3, 62.5, 125, 250, and 500 nM. Blue shading indicates the association phase.

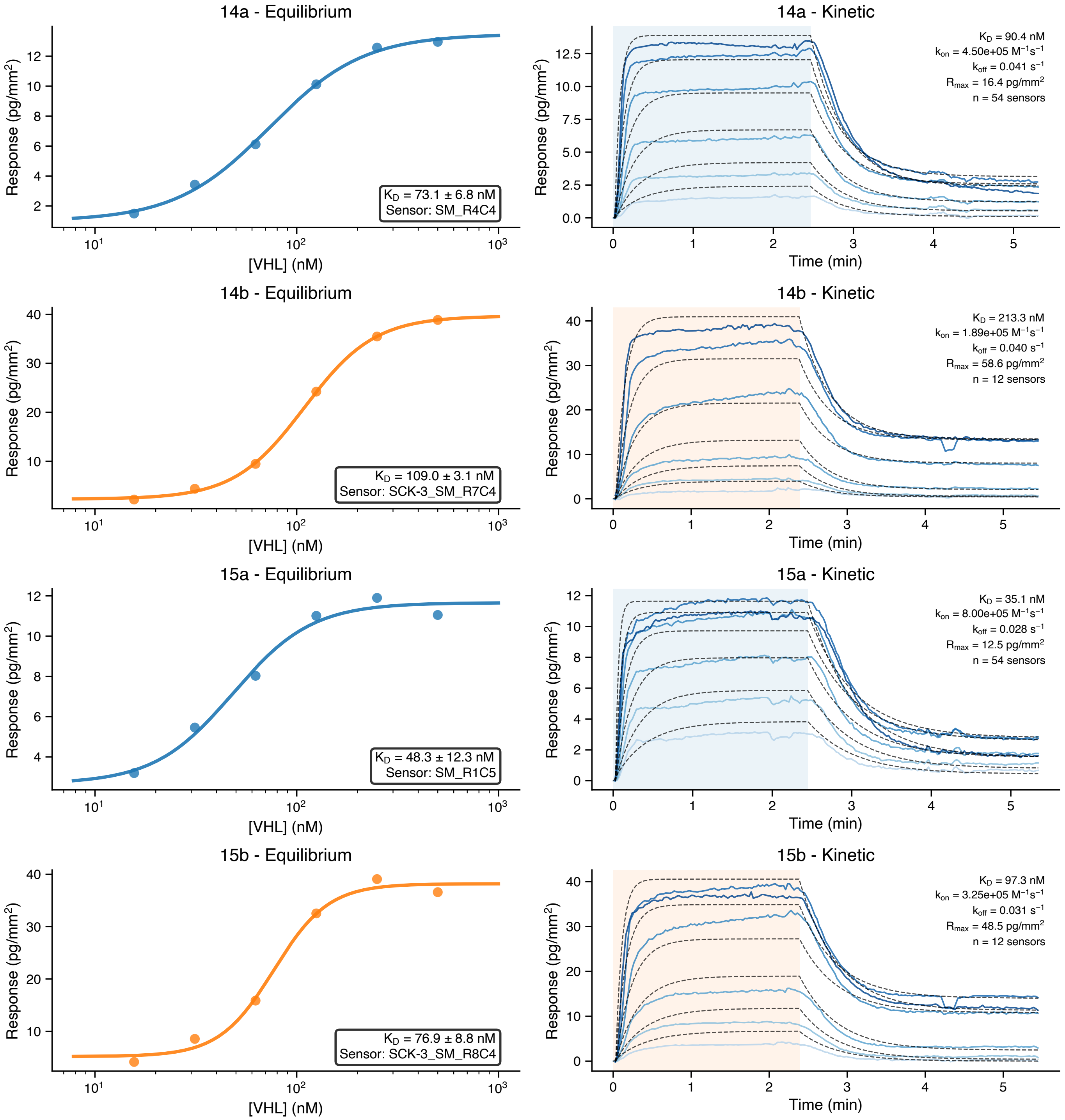

Figure SI-B20. Equilibrium and kinetic binding fits for compounds **14** and **15**. For each compound, the top row shows the singleplex variant (a) and the bottom row shows the multiplexed variant (b). Left panels: Equilibrium binding isotherms with Hill equation fits. Right panels: Multi-cycle kinetic sensorgrams with fitted curves (dashed lines). VHL concentrations used: 0, 15.6, 31.3, 62.5, 125, 250, and 500 nM. Blue shading indicates the association phase.

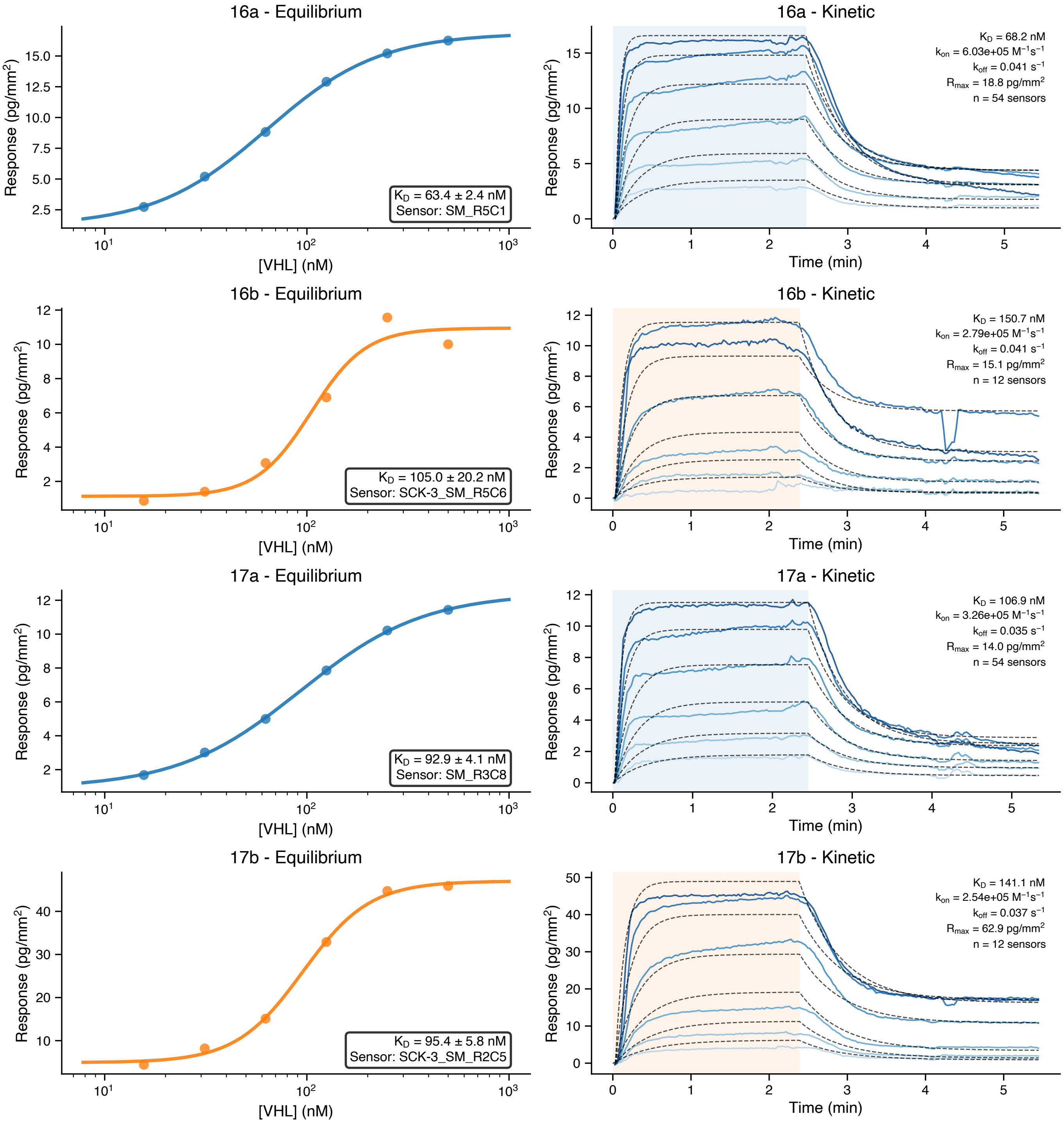

Figure SI-B21. Equilibrium and kinetic binding fits for compounds **16** and **17**. For each compound, the top row shows the singleplex variant (a) and the bottom row shows the multiplexed variant (b). Left panels: Equilibrium binding isotherms with Hill equation fits. Right panels: Multi-cycle kinetic sensorgrams with fitted curves (dashed lines). VHL concentrations used: 0, 15.6, 31.3, 62.5, 125, 250, and 500 nM. Blue shading indicates the association phase.

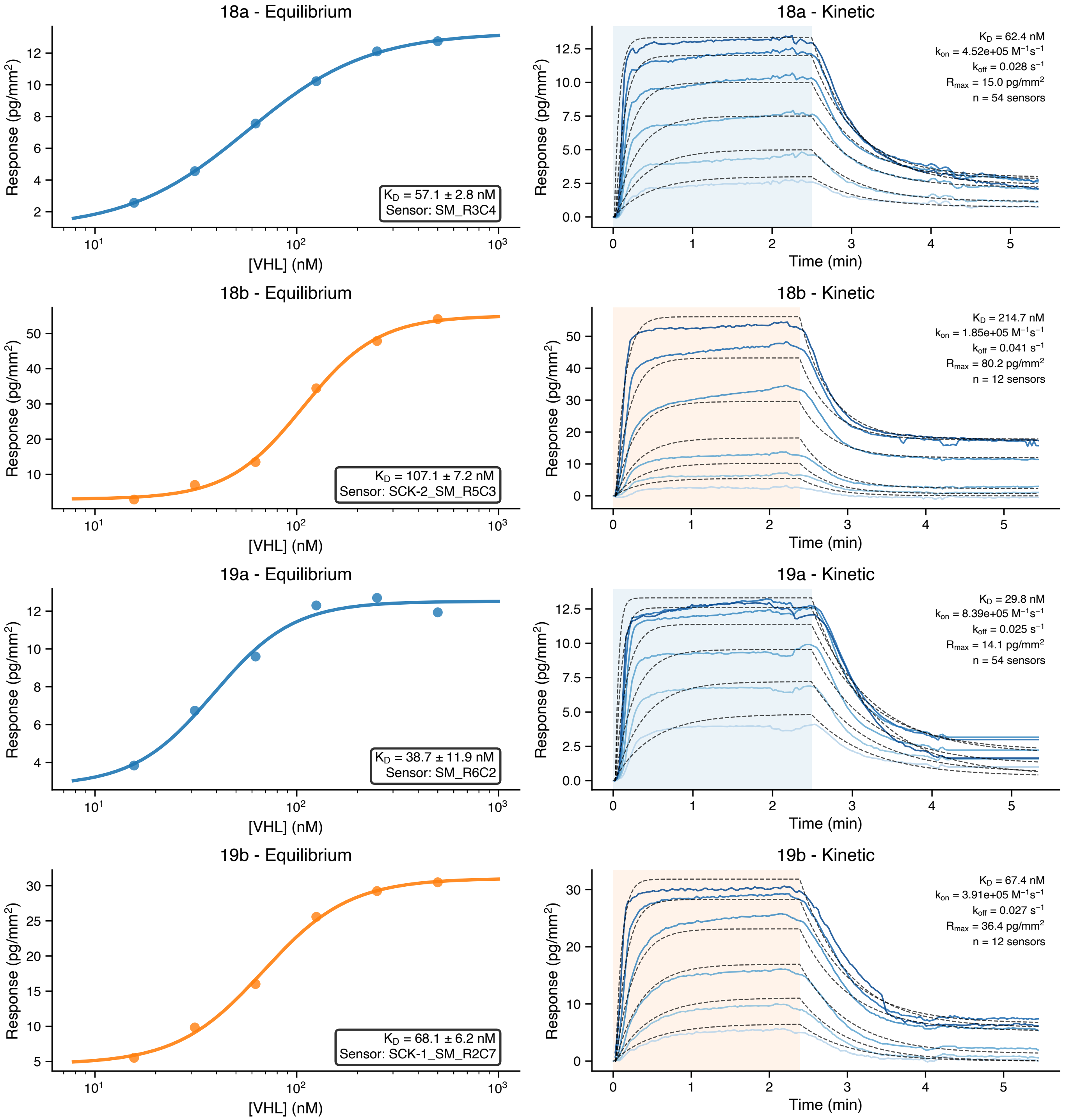

Figure SI-B22. Equilibrium and kinetic binding fits for compounds **18** and **19**. For each compound, the top row shows the singleplex variant (a) and the bottom row shows the multiplexed variant (b). Left panels: Equilibrium binding isotherms with Hill equation fits. Right panels: Multi-cycle kinetic sensorgrams with fitted curves (dashed lines). VHL concentrations used: 0, 15.6, 31.3, 62.5, 125, 250, and 500 nM. Blue shading indicates the association phase.

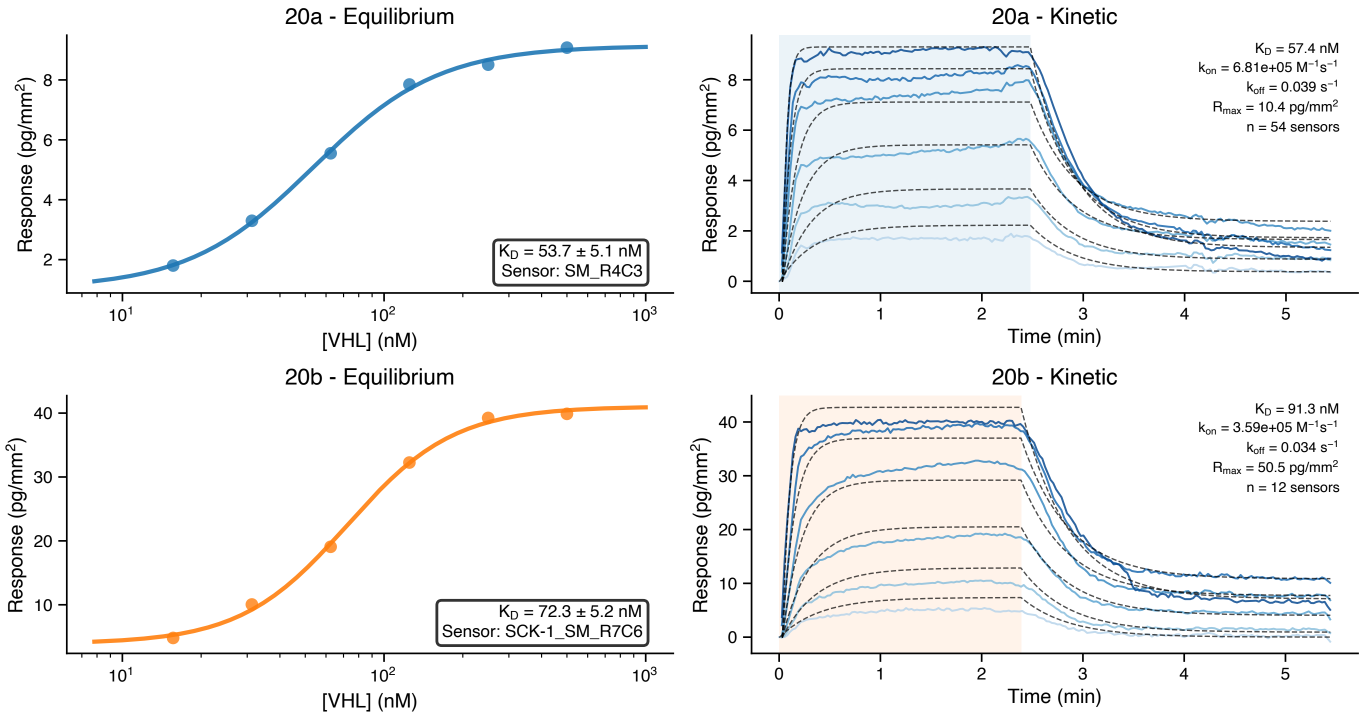

Figure SI-B23. Equilibrium and kinetic binding fits for compound **20**. The top row shows the singleplex variant (a) and the bottom row shows the multiplexed variant (b). Left panels: Equilibrium binding isotherms with Hill equation fits. Right panels: Multi-cycle kinetic sensorgrams with fitted curves (dashed lines). VHL concentrations used: 0, 15.6, 31.3, 62.5, 125, 250, and 500 nM. Blue shading indicates the association phase.

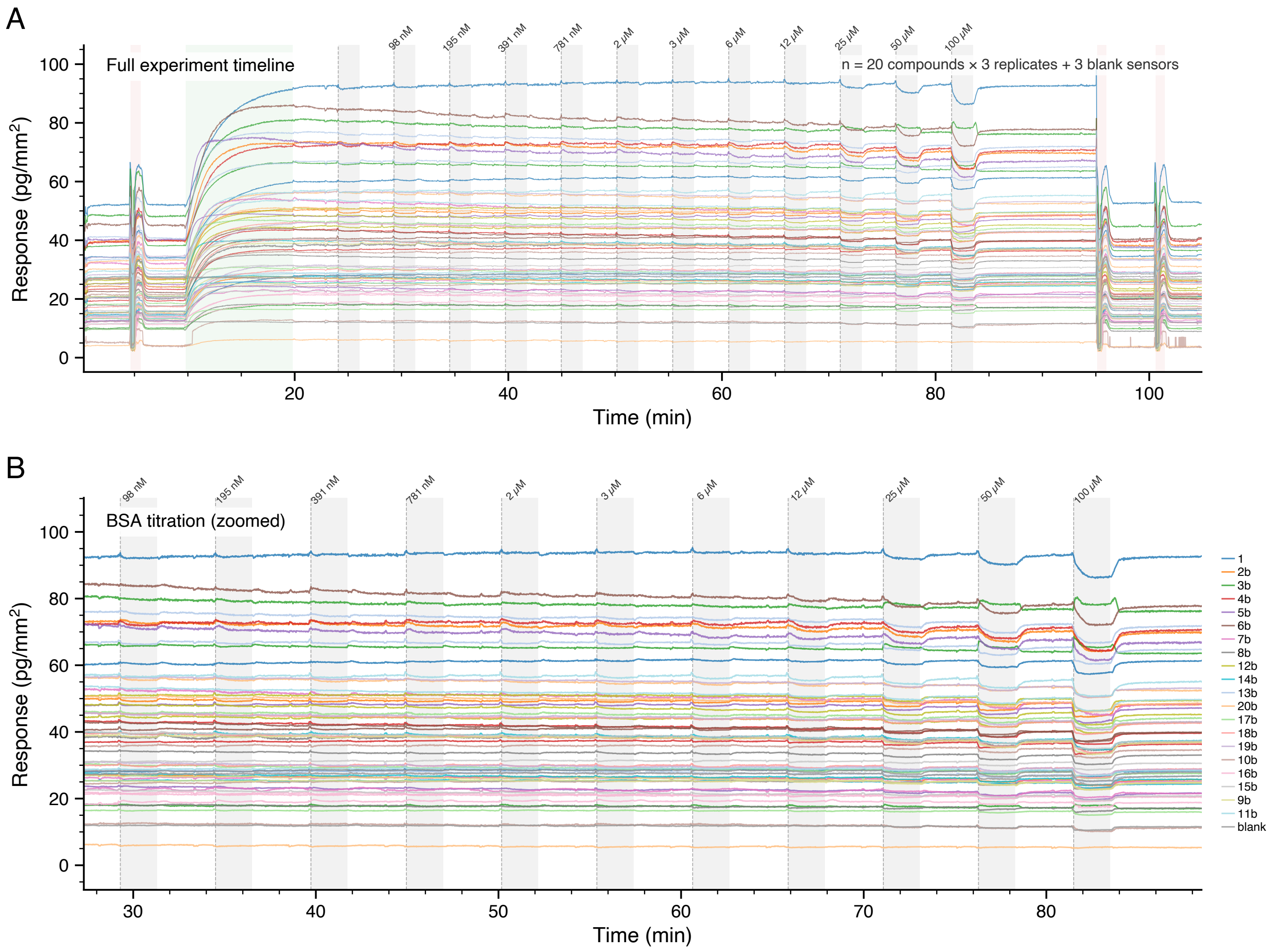

Figure SI-B24. BSA negative control screen. To assess non-specific protein binding, bovine serum albumin (BSA) was injected at increasing concentrations (97.7 nM to 100 µM) across all 21 compound-loaded molograms in a single-cycle kinetics format. (A) Full experimental timeline showing the oligo-compound immobilization phase (green shading) followed by the BSA concentration series (gray shading). Each trace corresponds to one mologram sensor (20 compounds in triplicate + 3 blank sensors, colored by compound identity). (B) Zoomed view of the BSA titration window. No significant binding response is observed for any compound up to 12.5 µM BSA. At higher concentrations (25–100 µM), a decrease in signal is observed, consistent with BSA adsorption to the PEG layer within the grating grooves. Importantly, no concentration-dependent increase in signal is seen for any compound, confirming that the VHL binding signals reported in the main text are target-specific and not caused by non-specific protein adsorption.

Tables SI-B2–SI-B20: Detailed Kinetic Parameters by Compound

The following tables present the fitted kinetic parameters for each individual sensor measurement. Parameters include the association rate constant (k_a), dissociation rate constant (k_d), dissociation constant (K_D), maximum response (R_{max}), normalized maximum response ($R_{max,norm}$), and the non-dissociating fraction (f_{stable}) for each of the six injection cycles. Multiplexed data (MP) are shown for all four runs (SCK-1 through SCK-4) with three replicate sensors per run. Singleplex data (SP) are from a separate chip.

Compound 1

Source	Sensor	k_a ($M^{-1}s^{-1}$)	k_d (s^{-1})	K_D (nM)	R_{max} (pg/mm^2)	$R_{max,n}$	$f_{s,1}$	$f_{s,2}$	$f_{s,3}$	$f_{s,4}$	$f_{s,5}$	$f_{s,6}$
MP-1	SM_R1C1	3.42×10^5	3.377×10^{-2}	98.7	175.63	1.20	0.00	0.02	0.12	0.31	0.18	0.17
MP-1	SM_R3C6	2.41×10^5	2.897×10^{-2}	120.1	80.99	1.23	0.00	0.00	0.06	0.28	0.17	0.14
MP-1	SM_R6C3	2.80×10^5	3.318×10^{-2}	118.3	48.84	1.22	0.00	0.04	0.15	0.30	0.19	0.17
MP-2	SM_R1C1	2.11×10^5	2.647×10^{-2}	125.6	158.99	1.27	0.00	0.01	0.04	0.46	0.26	0.16
MP-2	SM_R3C6	1.70×10^5	2.644×10^{-2}	155.8	53.13	1.32	0.00	0.00	0.03	0.33	0.28	0.18
MP-2	SM_R6C3	2.19×10^5	3.220×10^{-2}	146.8	62.36	1.32	0.00	0.00	0.00	0.39	0.25	0.16
MP-3	SM_R1C1	2.10×10^5	2.528×10^{-2}	120.2	183.02	1.28	0.05	0.00	0.06	0.43	0.26	0.17
MP-3	SM_R3C6	1.77×10^5	2.650×10^{-2}	149.5	58.81	1.33	0.07	0.02	0.05	0.33	0.28	0.16
MP-3	SM_R6C3	2.22×10^5	3.032×10^{-2}	136.8	73.47	1.30	0.01	0.00	0.05	0.40	0.27	0.17
MP-4	SM_R1C1	2.20×10^5	2.469×10^{-2}	112.3	170.66	1.22	0.00	0.00	0.06	0.44	0.25	0.21
MP-4	SM_R3C6	1.73×10^5	2.579×10^{-2}	149.2	60.84	1.26	0.15	0.06	0.09	0.35	0.24	0.23
MP-4	SM_R6C3	2.14×10^5	2.764×10^{-2}	129.3	71.74	1.24	0.00	0.01	0.07	0.41	0.23	0.21
SP	SM_R1C1	7.72×10^5	3.718×10^{-2}	48.1	8.25	1.05	0.03	0.14	0.13	0.24	0.26	0.13
SP	SM_R1C2	8.23×10^5	3.454×10^{-2}	42.0	13.50	1.06	0.11	0.14	0.14	0.25	0.24	0.13
SP	SM_R1C3	8.05×10^5	3.593×10^{-2}	44.7	22.19	1.05	0.12	0.13	0.13	0.21	0.25	0.18
SP	SM_R1C4	7.64×10^5	3.684×10^{-2}	48.2	17.78	1.07	0.09	0.13	0.13	0.18	0.23	0.19
SP	SM_R1C5	7.35×10^5	3.521×10^{-2}	47.9	16.23	1.07	0.08	0.12	0.11	0.19	0.23	0.17
SP	SM_R1C6	7.21×10^5	3.480×10^{-2}	48.3	18.61	1.07	0.06	0.11	0.11	0.18	0.23	0.18
SP	SM_R1C7	6.98×10^5	3.603×10^{-2}	51.6	19.02	1.07	0.00	0.05	0.08	0.16	0.19	0.15
SP	SM_R1C8	7.28×10^5	3.498×10^{-2}	48.1	16.68	1.07	0.07	0.11	0.10	0.19	0.24	0.16
SP	SM_R1C9	7.18×10^5	3.522×10^{-2}	49.1	13.96	1.08	0.08	0.14	0.11	0.20	0.22	0.15
SP	SM_R2C1	9.49×10^5	4.232×10^{-2}	44.6	16.32	1.02	0.07	0.11	0.13	0.21	0.26	0.14
SP	SM_R2C2	8.80×10^5	4.096×10^{-2}	46.5	19.76	1.05	0.10	0.13	0.14	0.20	0.24	0.16
SP	SM_R2C3	8.63×10^5	3.956×10^{-2}	45.8	22.36	1.05	0.09	0.12	0.13	0.20	0.23	0.16
SP	SM_R2C4	8.46×10^5	3.833×10^{-2}	45.3	20.28	1.05	0.09	0.12	0.13	0.18	0.23	0.17
SP	SM_R2C5	8.01×10^5	3.740×10^{-2}	46.7	16.61	1.04	0.06	0.07	0.11	0.17	0.21	0.16
SP	SM_R2C6	7.77×10^5	3.739×10^{-2}	48.1	16.51	1.05	0.02	0.07	0.10	0.16	0.20	0.16
SP	SM_R2C7	7.70×10^5	3.636×10^{-2}	47.2	18.52	1.06	0.09	0.08	0.10	0.17	0.20	0.16
SP	SM_R2C8	7.37×10^5	3.592×10^{-2}	48.7	18.96	1.06	0.06	0.11	0.09	0.16	0.21	0.17
SP	SM_R2C9	7.27×10^5	3.381×10^{-2}	46.5	16.96	1.06	0.16	0.15	0.11	0.19	0.23	0.18
SP	SM_R3C1	9.67×10^5	4.157×10^{-2}	43.0	18.77	1.02	0.14	0.15	0.16	0.22	0.26	0.16
SP	SM_R3C2	8.95×10^5	4.267×10^{-2}	47.7	16.59	1.04	0.02	0.07	0.11	0.20	0.22	0.15
SP	SM_R3C3	8.59×10^5	4.020×10^{-2}	46.8	19.18	1.04	0.06	0.08	0.10	0.18	0.22	0.16
SP	SM_R3C4	8.72×10^5	3.863×10^{-2}	44.3	20.79	1.04	0.07	0.11	0.12	0.19	0.23	0.16
SP	SM_R3C5	8.20×10^5	3.798×10^{-2}	46.3	17.81	1.04	0.02	0.10	0.11	0.15	0.20	0.17
SP	SM_R3C6	7.61×10^5	3.799×10^{-2}	49.9	14.39	1.05	0.07	0.07	0.07	0.16	0.20	0.15
SP	SM_R3C7	7.57×10^5	3.695×10^{-2}	48.8	14.85	1.06	0.05	0.08	0.09	0.16	0.20	0.16
SP	SM_R3C8	7.52×10^5	3.508×10^{-2}	46.6	15.72	1.07	0.16	0.13	0.11	0.19	0.22	0.16
SP	SM_R3C9	7.34×10^5	3.614×10^{-2}	49.2	17.22	1.07	0.09	0.10	0.10	0.16	0.20	0.15
SP	SM_R4C1	9.46×10^5	3.878×10^{-2}	41.0	22.64	1.02	0.16	0.16	0.17	0.25	0.27	0.15
SP	SM_R4C2	8.36×10^5	3.996×10^{-2}	47.8	17.48	1.05	0.05	0.10	0.10	0.18	0.23	0.15
SP	SM_R4C3	8.19×10^5	3.906×10^{-2}	47.7	14.82	1.06	0.11	0.12	0.10	0.19	0.22	0.15
SP	SM_R4C4	8.42×10^5	3.797×10^{-2}	45.1	16.04	1.04	0.09	0.10	0.11	0.19	0.23	0.15
SP	SM_R4C5	7.91×10^5	3.744×10^{-2}	47.4	19.31	1.04	0.04	0.10	0.10	0.16	0.20	0.14
SP	SM_R4C6	7.34×10^5	3.567×10^{-2}	48.6	17.60	1.06	0.15	0.12	0.07	0.17	0.22	0.16
SP	SM_R4C7	7.35×10^5	3.671×10^{-2}	50.0	14.27	1.05	0.10	0.11	0.09	0.15	0.20	0.16
SP	SM_R4C8	7.37×10^5	3.445×10^{-2}	46.7	14.72	1.06	0.15	0.13	0.10	0.18	0.21	0.17
SP	SM_R4C9	7.01×10^5	3.506×10^{-2}	50.0	16.73	1.06	0.06	0.10	0.09	0.15	0.20	0.15
SP	SM_R5C1	8.92×10^5	3.688×10^{-2}	41.4	22.31	1.02	0.14	0.16	0.16	0.24	0.28	0.16
SP	SM_R5C2	8.05×10^5	3.685×10^{-2}	45.8	21.40	1.03	0.08	0.11	0.11	0.19	0.24	0.15
SP	SM_R5C3	7.83×10^5	3.457×10^{-2}	44.1	16.96	1.04	0.12	0.15	0.13	0.19	0.24	0.17
SP	SM_R5C4	7.88×10^5	3.688×10^{-2}	46.8	12.94	1.05	0.05	0.13	0.11	0.18	0.23	0.15
SP	SM_R5C5	7.61×10^5	3.474×10^{-2}	45.7	16.73	1.05	0.11	0.13	0.12	0.19	0.23	0.16
SP	SM_R5C6	7.39×10^5	3.491×10^{-2}	47.3	22.17	1.06	0.09	0.13	0.11	0.17	0.23	0.17
SP	SM_R5C7	6.98×10^5	3.596×10^{-2}	51.5	18.22	1.06	0.01	0.10	0.09	0.14	0.19	0.16
SP	SM_R5C8	6.93×10^5	3.454×10^{-2}	49.8	14.18	1.04	0.10	0.10	0.09	0.15	0.19	0.16
SP	SM_R5C9	6.93×10^5	3.368×10^{-2}	48.6	14.05	1.05	0.08	0.12	0.11	0.16	0.20	0.16
SP	SM_R6C1	7.71×10^5	3.510×10^{-2}	45.5	16.42	1.04	0.08	0.10	0.13	0.22	0.24	0.11
SP	SM_R6C2	7.41×10^5	3.426×10^{-2}	46.2	22.40	1.04	0.02	0.09	0.11	0.17	0.22	0.13
SP	SM_R6C3	7.01×10^5	3.515×10^{-2}	50.1	22.73	1.07	0.03	0.08	0.07	0.17	0.21	0.12
SP	SM_R6C4	7.27×10^5	3.362×10^{-2}	46.2	18.37	1.05	0.07	0.10	0.11	0.16	0.21	0.15
SP	SM_R6C5	6.97×10^5	3.496×10^{-2}	50.2	14.43	1.05	0.06	0.09	0.09	0.15	0.20	0.13
SP	SM_R6C6	7.15×10^5	3.414×10^{-2}	47.7	18.71	1.06	0.09	0.11	0.11	0.18	0.21	0.15
SP	SM_R6C7	6.61×10^5	3.353×10^{-2}	50.8	23.15	1.07	0.08	0.08	0.08	0.15	0.20	0.16
SP	SM_R6C8	6.40×10^5	3.313×10^{-2}	51.8	20.98	1.06	0.08	0.10	0.08	0.14	0.18	0.14
SP	SM_R6C9	5.99×10^5	3.350×10^{-2}	55.9	15.64	1.06	0.00	0.03	0.04	0.11	0.16	0.13

Kinetic parameters for compound 1. SP = singleplex, MP-1/2/3/4 = multiplexed chip SCK-1/2/3/4. $f_{s,i}$ = non-dissociating fraction for cycle i.

Compound 2

Source	Sensor	k_a ($M^{-1}s^{-1}$)	k_d (s^{-1})	K_D (nM)	R_{max} (pg/mm^2)	$R_{max,n}$	$f_{s,1}$	$f_{s,2}$	$f_{s,3}$	$f_{s,4}$	$f_{s,5}$	$f_{s,6}$
**Variant a (54 sensors)**												
SP	SM_R1C1	6.39×10^5	4.593×10^{-2}	71.9	10.23	1.19	0.06	0.18	0.21	0.29	0.28	0.15
SP	SM_R1C2	6.34×10^5	4.259×10^{-2}	67.2	16.27	1.19	0.20	0.24	0.20	0.28	0.28	0.18
SP	SM_R1C3	5.52×10^5	4.869×10^{-2}	88.2	27.36	1.23	0.11	0.15	0.15	0.23	0.26	0.20
SP	SM_R1C4	5.39×10^5	4.559×10^{-2}	84.6	21.42	1.22	0.15	0.18	0.16	0.22	0.26	0.21
SP	SM_R1C5	5.67×10^5	4.577×10^{-2}	80.7	19.54	1.20	0.15	0.17	0.16	0.23	0.26	0.21
SP	SM_R1C6	5.50×10^5	4.569×10^{-2}	83.0	22.44	1.22	0.19	0.21	0.18	0.24	0.27	0.21
SP	SM_R1C7	5.48×10^5	4.473×10^{-2}	81.6	22.99	1.21	0.18	0.21	0.18	0.24	0.27	0.20
SP	SM_R1C8	5.49×10^5	4.468×10^{-2}	81.4	19.94	1.20	0.21	0.21	0.19	0.25	0.26	0.20
SP	SM_R1C9	5.98×10^5	4.371×10^{-2}	73.1	16.15	1.19	0.26	0.26	0.22	0.28	0.29	0.22
SP	SM_R2C1	7.02×10^5	5.244×10^{-2}	74.7	20.20	1.20	0.22	0.21	0.20	0.27	0.29	0.18
SP	SM_R2C2	6.37×10^5	4.936×10^{-2}	77.5	23.95	1.20	0.18	0.20	0.18	0.25	0.27	0.19
SP	SM_R2C3	6.08×10^5	4.935×10^{-2}	81.2	27.41	1.21	0.12	0.16	0.16	0.23	0.25	0.18
SP	SM_R2C4	5.88×10^5	4.864×10^{-2}	82.8	24.47	1.22	0.12	0.17	0.16	0.23	0.25	0.18
SP	SM_R2C5	5.83×10^5	4.581×10^{-2}	78.6	19.48	1.19	0.16	0.19	0.17	0.23	0.26	0.21
SP	SM_R2C6	5.65×10^5	4.541×10^{-2}	80.4	19.37	1.20	0.20	0.21	0.18	0.23	0.25	0.21
SP	SM_R2C7	5.15×10^5	4.564×10^{-2}	88.7	21.76	1.21	0.06	0.13	0.12	0.19	0.22	0.19
SP	SM_R2C8	5.12×10^5	4.500×10^{-2}	87.9	21.84	1.23	0.14	0.18	0.15	0.21	0.23	0.19
SP	SM_R2C9	5.05×10^5	4.609×10^{-2}	91.2	19.54	1.23	0.10	0.15	0.14	0.20	0.23	0.18
SP	SM_R3C1	6.85×10^5	5.177×10^{-2}	75.6	22.75	1.20	0.16	0.21	0.19	0.26	0.27	0.17
SP	SM_R3C2	6.55×10^5	4.946×10^{-2}	75.6	19.20	1.20	0.21	0.22	0.19	0.25	0.26	0.19
SP	SM_R3C3	5.94×10^5	4.807×10^{-2}	80.9	22.60	1.21	0.14	0.19	0.16	0.23	0.25	0.19
SP	SM_R3C4	5.78×10^5	4.925×10^{-2}	85.2	24.26	1.21	0.09	0.16	0.15	0.21	0.23	0.18
SP	SM_R3C5	5.89×10^5	4.786×10^{-2}	81.3	21.10	1.21	0.18	0.21	0.17	0.23	0.26	0.20
SP	SM_R3C6	5.58×10^5	4.819×10^{-2}	86.3	17.52	1.19	0.16	0.18	0.14	0.20	0.23	0.21
SP	SM_R3C7	5.46×10^5	4.573×10^{-2}	83.8	17.84	1.20	0.16	0.18	0.15	0.21	0.23	0.19
SP	SM_R3C8	5.11×10^5	4.520×10^{-2}	88.4	18.77	1.22	0.09	0.14	0.12	0.19	0.21	0.18
SP	SM_R3C9	5.12×10^5	4.581×10^{-2}	89.5	20.23	1.23	0.12	0.16	0.14	0.20	0.21	0.18
SP	SM_R4C1	6.41×10^5	4.844×10^{-2}	75.6	28.13	1.21	0.13	0.18	0.18	0.26	0.27	0.17
SP	SM_R4C2	6.18×10^5	4.794×10^{-2}	77.5	21.03	1.20	0.19	0.21	0.19	0.24	0.26	0.18
SP	SM_R4C3	5.90×10^5	4.796×10^{-2}	81.3	17.96	1.21	0.18	0.19	0.16	0.22	0.25	0.19
SP	SM_R4C4	5.77×10^5	4.833×10^{-2}	83.8	19.14	1.20	0.09	0.15	0.15	0.21	0.23	0.17
SP	SM_R4C5	5.82×10^5	4.723×10^{-2}	81.1	23.04	1.19	0.14	0.19	0.15	0.22	0.24	0.19
SP	SM_R4C6	5.24×10^5	4.923×10^{-2}	94.0	21.38	1.22	0.11	0.14	0.12	0.18	0.21	0.18
SP	SM_R4C7	5.21×10^5	4.617×10^{-2}	88.7	17.23	1.21	0.22	0.21	0.16	0.20	0.23	0.20
SP	SM_R4C8	5.14×10^5	4.535×10^{-2}	88.2	17.13	1.22	0.12	0.16	0.14	0.19	0.21	0.18
SP	SM_R4C9	5.18×10^5	4.553×10^{-2}	87.9	19.77	1.20	0.14	0.18	0.14	0.20	0.22	0.18
SP	SM_R5C1	6.21×10^5	4.599×10^{-2}	74.1	27.32	1.18	0.15	0.19	0.19	0.27	0.28	0.16
SP	SM_R5C2	5.89×10^5	4.484×10^{-2}	76.1	25.97	1.18	0.17	0.19	0.18	0.24	0.26	0.18
SP	SM_R5C3	5.76×10^5	4.673×10^{-2}	81.2	20.07	1.18	0.15	0.18	0.16	0.22	0.25	0.18
SP	SM_R5C4	6.01×10^5	4.523×10^{-2}	75.2	15.56	1.18	0.24	0.22	0.19	0.24	0.25	0.19
SP	SM_R5C5	5.54×10^5	4.662×10^{-2}	84.1	20.58	1.21	0.16	0.18	0.15	0.21	0.25	0.19
SP	SM_R5C6	5.54×10^5	4.523×10^{-2}	81.6	26.78	1.20	0.24	0.22	0.17	0.22	0.25	0.20
SP	SM_R5C7	5.27×10^5	4.523×10^{-2}	85.9	22.09	1.21	0.21	0.21	0.16	0.21	0.23	0.19
SP	SM_R5C8	5.09×10^5	4.451×10^{-2}	87.5	17.34	1.20	0.16	0.17	0.14	0.19	0.21	0.18
SP	SM_R5C9	4.79×10^5	4.482×10^{-2}	93.6	16.71	1.21	0.09	0.15	0.12	0.18	0.20	0.18
SP	SM_R6C1	5.92×10^5	4.033×10^{-2}	68.1	19.80	1.17	0.23	0.22	0.20	0.27	0.27	0.16
SP	SM_R6C2	5.56×10^5	4.128×10^{-2}	74.3	26.94	1.17	0.21	0.21	0.19	0.25	0.26	0.18
SP	SM_R6C3	5.27×10^5	4.075×10^{-2}	77.4	26.99	1.18	0.19	0.19	0.16	0.23	0.25	0.18
SP	SM_R6C4	5.41×10^5	3.966×10^{-2}	73.3	21.22	1.18	0.22	0.22	0.18	0.24	0.25	0.19
SP	SM_R6C5	5.22×10^5	3.945×10^{-2}	75.5	17.47	1.18	0.20	0.23	0.18	0.23	0.24	0.18
SP	SM_R6C6	4.75×10^5	4.022×10^{-2}	84.6	22.31	1.21	0.15	0.16	0.14	0.21	0.23	0.17
SP	SM_R6C7	4.76×10^5	4.161×10^{-2}	87.4	28.00	1.21	0.20	0.19	0.15	0.20	0.23	0.19
SP	SM_R6C8	4.69×10^5	4.073×10^{-2}	86.8	24.74	1.21	0.18	0.19	0.15	0.20	0.22	0.19
SP	SM_R6C9	4.73×10^5	4.040×10^{-2}	85.5	18.10	1.19	0.20	0.20	0.15	0.20	0.22	0.19
**Variant b (12 sensors)**												
MP-1	SM_R1C2	2.28×10^5	3.978×10^{-2}	174.8	234.88	1.38	0.00	0.04	0.13	0.32	0.26	0.24
MP-1	SM_R3C7	1.79×10^5	3.715×10^{-2}	207.6	67.76	1.44	0.00	0.04	0.11	0.29	0.27	0.24
MP-1	SM_R6C4	2.18×10^5	4.021×10^{-2}	184.2	140.68	1.41	0.00	0.02	0.12	0.32	0.24	0.22
MP-2	SM_R1C2	1.53×10^5	3.090×10^{-2}	202.1	180.19	1.46	0.02	0.05	0.09	0.40	0.34	0.24
MP-2	SM_R3C7	1.38×10^5	3.530×10^{-2}	255.1	30.73	1.56	0.05	0.02	0.07	0.36	0.44	0.34
MP-2	SM_R6C4	1.69×10^5	3.646×10^{-2}	215.3	115.56	1.49	0.00	0.00	0.06	0.38	0.32	0.22
MP-3	SM_R1C2	1.61×10^5	2.900×10^{-2}	180.5	183.20	1.42	0.25	0.09	0.12	0.39	0.36	0.28
MP-3	SM_R3C7	1.63×10^5	3.201×10^{-2}	196.5	27.20	1.47	0.27	0.04	0.08	0.30	0.46	0.28
MP-3	SM_R6C4	1.76×10^5	3.524×10^{-2}	200.2	119.95	1.45	0.16	0.03	0.11	0.36	0.34	0.27
MP-4	SM_R1C2	1.63×10^5	2.862×10^{-2}	176.0	173.37	1.37	0.00	0.04	0.10	0.40	0.35	0.32
MP-4	SM_R3C7	1.34×10^5	3.165×10^{-2}	235.7	31.38	1.38	0.07	0.07	0.12	0.32	0.36	0.39
MP-4	SM_R6C4	1.83×10^5	3.310×10^{-2}	181.3	112.77	1.38	0.04	0.06	0.11	0.40	0.32	0.31

Kinetic parameters for compound 2. SP = singleplex, MP-1/2/3/4 = multiplexed chip SCK-1/2/3/4. $f_{s,i}$ = non-dissociating fraction for cycle i.

Compound 3

Source	Sensor	k_a ($M^{-1}s^{-1}$)	k_d (s^{-1})	K_D (nM)	R_{max} (pg/mm^2)	$R_{max,n}$	$f_{s,1}$	$f_{s,2}$	$f_{s,3}$	$f_{s,4}$	$f_{s,5}$	$f_{s,6}$
**Variant a (54 sensors)**												
SP	SM_R1C1	1.38×10^{6}	3.050×10^{-2}	22.1	6.09	0.97	0.14	0.21	0.22	0.27	0.17	0.04
SP	SM_R1C2	1.23×10^{6}	2.693×10^{-2}	21.8	10.03	0.96	0.17	0.21	0.29	0.33	0.24	0.09
SP	SM_R1C3	1.13×10^{6}	3.185×10^{-2}	28.3	17.36	1.04	0.13	0.18	0.21	0.29	0.26	0.13
SP	SM_R1C4	1.13×10^{6}	3.185×10^{-2}	28.3	13.64	1.02	0.11	0.16	0.20	0.30	0.26	0.13
SP	SM_R1C5	1.21×10^{6}	3.301×10^{-2}	27.3	13.24	1.01	0.14	0.21	0.22	0.29	0.26	0.15
SP	SM_R1C6	1.14×10^{6}	3.120×10^{-2}	27.5	14.95	1.02	0.16	0.17	0.23	0.31	0.27	0.12
SP	SM_R1C7	1.09×10^{6}	3.075×10^{-2}	28.3	15.20	1.01	0.13	0.18	0.21	0.29	0.27	0.12
SP	SM_R1C8	1.04×10^{6}	3.021×10^{-2}	28.9	13.47	1.05	0.11	0.12	0.21	0.29	0.24	0.09
SP	SM_R1C9	1.09×10^{6}	3.069×10^{-2}	28.1	10.90	1.00	0.14	0.17	0.23	0.29	0.26	0.08
SP	SM_R2C1	1.14×10^{6}	2.939×10^{-2}	25.7	13.07	1.02	0.07	0.15	0.22	0.30	0.23	0.07
SP	SM_R2C2	1.06×10^{6}	2.938×10^{-2}	27.7	15.59	1.03	0.16	0.17	0.21	0.30	0.27	0.13
SP	SM_R2C3	1.10×10^{6}	2.949×10^{-2}	26.8	17.20	1.01	0.13	0.14	0.22	0.29	0.25	0.09
SP	SM_R2C4	1.11×10^{6}	2.963×10^{-2}	26.7	15.87	1.02	0.17	0.16	0.23	0.30	0.27	0.12
SP	SM_R2C5	1.05×10^{6}	2.981×10^{-2}	28.4	13.36	1.04	0.13	0.14	0.19	0.27	0.26	0.11
SP	SM_R2C6	1.04×10^{6}	2.895×10^{-2}	27.7	13.94	1.05	0.11	0.15	0.22	0.26	0.26	0.14
SP	SM_R2C7	9.89×10^{5}	3.012×10^{-2}	30.4	16.01	1.05	0.06	0.11	0.18	0.26	0.27	0.13
SP	SM_R2C8	9.14×10^{5}	2.942×10^{-2}	32.2	16.26	1.05	0.10	0.08	0.17	0.26	0.27	0.11
SP	SM_R2C9	9.14×10^{5}	2.992×10^{-2}	32.7	14.13	1.06	0.14	0.14	0.19	0.26	0.27	0.14
SP	SM_R3C1	1.08×10^{6}	2.712×10^{-2}	25.0	14.51	1.01	0.11	0.13	0.23	0.30	0.24	0.06
SP	SM_R3C2	1.03×10^{6}	2.667×10^{-2}	26.0	12.55	1.02	0.12	0.15	0.21	0.28	0.25	0.09
SP	SM_R3C3	9.66×10^{5}	2.812×10^{-2}	29.1	16.00	1.05	0.09	0.11	0.20	0.28	0.25	0.09
SP	SM_R3C4	1.00×10^{6}	2.802×10^{-2}	28.0	18.34	1.05	0.10	0.13	0.20	0.27	0.25	0.10
SP	SM_R3C5	9.57×10^{5}	2.921×10^{-2}	30.5	16.33	1.05	0.08	0.13	0.18	0.25	0.26	0.14
SP	SM_R3C6	9.69×10^{5}	2.915×10^{-2}	30.1	12.80	1.04	0.11	0.12	0.17	0.26	0.26	0.15
SP	SM_R3C7	9.31×10^{5}	2.832×10^{-2}	30.4	12.95	1.02	0.06	0.14	0.17	0.24	0.26	0.15
SP	SM_R3C8	9.57×10^{5}	2.899×10^{-2}	30.3	12.94	1.04	0.11	0.11	0.18	0.27	0.26	0.12
SP	SM_R3C9	8.77×10^{5}	2.694×10^{-2}	30.7	13.72	1.04	0.09	0.12	0.19	0.25	0.25	0.11
SP	SM_R4C1	1.09×10^{6}	2.660×10^{-2}	24.4	17.63	1.01	0.15	0.18	0.24	0.31	0.25	0.08
SP	SM_R4C2	1.11×10^{6}	2.914×10^{-2}	26.4	13.81	1.05	0.09	0.15	0.21	0.29	0.25	0.10
SP	SM_R4C3	1.04×10^{6}	2.805×10^{-2}	27.1	11.91	1.03	0.09	0.13	0.19	0.29	0.25	0.11
SP	SM_R4C4	1.09×10^{6}	2.986×10^{-2}	27.3	12.53	1.04	0.15	0.16	0.23	0.31	0.27	0.13
SP	SM_R4C5	9.75×10^{5}	2.784×10^{-2}	28.5	15.34	1.05	0.08	0.10	0.18	0.28	0.25	0.10
SP	SM_R4C6	9.59×10^{5}	2.845×10^{-2}	29.7	13.43	1.06	0.13	0.13	0.19	0.28	0.27	0.14
SP	SM_R4C7	8.34×10^{5}	2.517×10^{-2}	30.2	11.57	1.03	0.05	0.08	0.14	0.23	0.25	0.10
SP	SM_R4C8	8.62×10^{5}	2.581×10^{-2}	29.9	11.40	1.03	0.12	0.13	0.19	0.25	0.24	0.13
SP	SM_R4C9	9.11×10^{5}	2.737×10^{-2}	30.1	12.89	1.03	0.18	0.19	0.20	0.28	0.27	0.16
SP	SM_R5C1	9.89×10^{5}	2.738×10^{-2}	27.7	18.13	1.04	0.11	0.16	0.23	0.31	0.25	0.09
SP	SM_R5C2	9.77×10^{5}	2.799×10^{-2}	28.7	18.63	1.05	0.13	0.16	0.22	0.30	0.26	0.12
SP	SM_R5C3	1.02×10^{6}	2.936×10^{-2}	28.8	14.66	1.05	0.15	0.19	0.22	0.29	0.27	0.14
SP	SM_R5C4	8.89×10^{5}	2.613×10^{-2}	29.4	10.54	1.04	0.01	0.09	0.16	0.25	0.22	0.07
SP	SM_R5C5	9.77×10^{5}	2.999×10^{-2}	30.7	12.75	0.98	0.12	0.13	0.20	0.27	0.27	0.14
SP	SM_R5C6	9.40×10^{5}	2.832×10^{-2}	30.1	16.30	1.03	0.10	0.12	0.19	0.26	0.26	0.13
SP	SM_R5C7	8.51×10^{5}	2.702×10^{-2}	31.8	14.03	1.04	0.14	0.14	0.18	0.26	0.26	0.14
SP	SM_R5C8	9.39×10^{5}	2.897×10^{-2}	30.8	10.96	1.05	0.19	0.19	0.20	0.27	0.28	0.18
SP	SM_R5C9	8.71×10^{5}	2.750×10^{-2}	31.6	11.56	1.05	0.12	0.14	0.18	0.26	0.25	0.13
SP	SM_R6C1	1.03×10^{6}	2.626×10^{-2}	25.5	14.38	1.02	0.17	0.20	0.25	0.32	0.26	0.10
SP	SM_R6C2	9.31×10^{5}	2.643×10^{-2}	28.4	19.57	1.04	0.11	0.15	0.22	0.29	0.24	0.10
SP	SM_R6C3	9.38×10^{5}	2.599×10^{-2}	27.7	18.80	1.04	0.09	0.13	0.21	0.28	0.25	0.10
SP	SM_R6C4	8.87×10^{5}	2.646×10^{-2}	29.8	15.23	1.04	0.09	0.14	0.18	0.27	0.26	0.12
SP	SM_R6C5	8.76×10^{5}	2.588×10^{-2}	29.6	12.04	1.05	0.16	0.17	0.20	0.28	0.26	0.13
SP	SM_R6C6	8.59×10^{5}	2.609×10^{-2}	30.4	15.70	1.05	0.12	0.15	0.18	0.26	0.26	0.13
SP	SM_R6C7	8.55×10^{5}	2.686×10^{-2}	31.4	18.99	1.06	0.13	0.15	0.18	0.25	0.26	0.14
SP	SM_R6C8	8.11×10^{5}	2.520×10^{-2}	31.1	16.87	1.05	0.13	0.14	0.17	0.25	0.26	0.12
SP	SM_R6C9	8.34×10^{5}	2.821×10^{-2}	33.8	12.12	1.07	0.08	0.12	0.16	0.25	0.24	0.13
**Variant b (12 sensors)**												
MP-1	SM_R1C3	3.57×10^{5}	2.902×10^{-2}	81.2	146.11	1.19	0.00	0.00	0.09	0.24	0.19	0.17
MP-1	SM_R3C8	2.42×10^{5}	2.751×10^{-2}	113.8	26.39	1.21	0.00	0.02	0.08	0.23	0.26	0.21
MP-1	SM_R6C5	3.76×10^{5}	3.065×10^{-2}	81.4	85.71	1.20	0.00	0.00	0.13	0.27	0.21	0.17
MP-2	SM_R1C3	2.38×10^{5}	2.309×10^{-2}	97.0	102.03	1.26	0.00	0.00	0.04	0.33	0.28	0.16
MP-2	SM_R3C8	1.42×10^{5}	4.072×10^{-2}	285.9	7.61	1.46	0.00	0.00	0.12	0.41	0.93	0.59
MP-2	SM_R6C5	2.72×10^{5}	2.896×10^{-2}	106.4	64.90	1.28	0.00	0.01	0.07	0.34	0.29	0.19
MP-3	SM_R1C3	2.54×10^{5}	2.288×10^{-2}	90.2	106.25	1.24	0.26	0.05	0.10	0.34	0.32	0.23
MP-3	SM_R3C8	1.12×10^{3}	9.846×10^{-1}	875221.1	0.00	0.00	0.40	0.68	0.72	0.10	0.18	0.97
MP-3	SM_R6C5	2.75×10^{5}	2.783×10^{-2}	101.0	66.17	1.27	0.00	0.00	0.09	0.33	0.32	0.23
MP-4	SM_R1C3	2.59×10^{5}	2.162×10^{-2}	83.5	101.64	1.19	0.08	0.08	0.11	0.34	0.29	0.27
MP-4	SM_R3C8	2.01×10^{5}	2.498×10^{-2}	124.2	18.40	1.16	0.16	0.13	0.16	0.29	0.30	0.41
MP-4	SM_R6C5	2.99×10^{5}	2.592×10^{-2}	86.8	63.80	1.19	0.04	0.06	0.11	0.35	0.28	0.28

Kinetic parameters for compound 3. SP = singleplex, MP-1/2/3/4 = multiplexed chip SCK-1/2/3/4. $f_{s,i}$ = non-dissociating fraction for cycle i.

Compound 4

Source	Sensor	k_a ($M^{-1}s^{-1}$)	k_d (s^{-1})	K_D (nM)	R_{max} (pg/mm^2)	$R_{max,n}$	$f_{s,1}$	$f_{s,2}$	$f_{s,3}$	$f_{s,4}$	$f_{s,5}$	$f_{s,6}$
**Variant a (54 sensors)**												
SP	SM_R1C1	6.79×10^{6}	1.061×10^{-1}	15.6	4.70	0.60	0.40	0.09	0.20	0.26	0.45	0.39
SP	SM_R1C2	5.03×10^{6}	8.993×10^{-2}	17.9	8.38	0.66	0.40	0.16	0.25	0.20	0.35	0.31
SP	SM_R1C3	3.32×10^{6}	8.213×10^{-2}	24.7	14.09	0.69	0.40	0.10	0.16	0.16	0.29	0.32
SP	SM_R1C4	2.51×10^{6}	7.413×10^{-2}	29.6	12.03	0.74	0.40	0.06	0.17	0.13	0.26	0.28
SP	SM_R1C5	2.53×10^{6}	7.122×10^{-2}	28.1	10.74	0.74	0.40	0.07	0.16	0.13	0.26	0.28
SP	SM_R1C6	2.13×10^{6}	6.860×10^{-2}	32.2	12.70	0.77	0.40	0.13	0.19	0.16	0.28	0.30
SP	SM_R1C7	2.08×10^{6}	6.629×10^{-2}	31.9	12.91	0.79	0.40	0.13	0.18	0.17	0.29	0.31
SP	SM_R1C8	2.20×10^{6}	6.709×10^{-2}	30.6	11.34	0.76	0.40	0.12	0.19	0.16	0.27	0.29
SP	SM_R1C9	2.35×10^{6}	6.946×10^{-2}	29.6	9.52	0.76	0.40	0.14	0.22	0.18	0.31	0.31
SP	SM_R2C1	5.99×10^{6}	1.059×10^{-1}	17.7	8.87	0.60	0.40	0.04	0.12	0.17	0.34	0.32
SP	SM_R2C2	4.33×10^{6}	9.464×10^{-2}	21.8	11.62	0.66	0.40	0.07	0.17	0.15	0.29	0.28
SP	SM_R2C3	3.84×10^{6}	8.791×10^{-2}	22.9	13.47	0.68	0.40	0.08	0.15	0.15	0.28	0.28
SP	SM_R2C4	3.17×10^{6}	8.083×10^{-2}	25.5	12.72	0.71	0.40	0.08	0.15	0.12	0.26	0.27
SP	SM_R2C5	2.61×10^{6}	7.330×10^{-2}	28.1	10.92	0.73	0.40	0.05	0.17	0.12	0.25	0.28
SP	SM_R2C6	2.26×10^{6}	7.020×10^{-2}	31.0	11.44	0.75	0.40	0.11	0.13	0.14	0.24	0.27
SP	SM_R2C7	1.97×10^{6}	6.625×10^{-2}	33.7	13.49	0.79	0.40	0.08	0.14	0.11	0.21	0.24
SP	SM_R2C8	1.89×10^{6}	6.616×10^{-2}	35.0	14.02	0.81	0.40	0.11	0.16	0.11	0.23	0.24
SP	SM_R2C9	1.83×10^{6}	6.319×10^{-2}	34.5	12.61	0.81	0.40	0.07	0.15	0.09	0.21	0.23
SP	SM_R3C1	5.22×10^{6}	1.029×10^{-1}	19.7	10.71	0.64	0.40	0.06	0.17	0.16	0.34	0.31
SP	SM_R3C2	4.24×10^{6}	9.505×10^{-2}	22.4	9.93	0.67	0.40	0.06	0.20	0.13	0.29	0.27
SP	SM_R3C3	3.42×10^{6}	8.495×10^{-2}	24.9	12.47	0.69	0.40	0.08	0.16	0.13	0.27	0.26
SP	SM_R3C4	3.13×10^{6}	8.063×10^{-2}	25.8	13.80	0.71	0.40	0.11	0.17	0.12	0.26	0.26
SP	SM_R3C5	2.53×10^{6}	7.299×10^{-2}	28.9	12.53	0.74	0.40	0.09	0.14	0.12	0.24	0.25
SP	SM_R3C6	2.03×10^{6}	6.824×10^{-2}	33.5	10.42	0.78	0.40	0.04	0.16	0.10	0.21	0.24
SP	SM_R3C7	1.92×10^{6}	6.589×10^{-2}	34.2	10.90	0.80	0.40	0.07	0.15	0.11	0.21	0.22
SP	SM_R3C8	1.90×10^{6}	6.531×10^{-2}	34.4	11.52	0.81	0.40	0.09	0.16	0.13	0.23	0.23
SP	SM_R3C9	1.84×10^{6}	6.380×10^{-2}	34.7	12.89	0.82	0.40	0.09	0.13	0.12	0.23	0.24
SP	SM_R4C1	2.36×10^{6}	7.377×10^{-2}	31.2	16.66	0.80	0.40	0.05	0.15	0.12	0.25	0.24
SP	SM_R4C2	3.16×10^{6}	8.166×10^{-2}	25.9	11.45	0.72	0.40	0.10	0.15	0.16	0.27	0.26
SP	SM_R4C3	2.53×10^{6}	7.580×10^{-2}	29.9	9.92	0.74	0.40	0.06	0.15	0.11	0.23	0.26
SP	SM_R4C4	2.58×10^{6}	7.523×10^{-2}	29.2	11.00	0.75	0.40	0.10	0.17	0.13	0.26	0.27
SP	SM_R4C5	2.20×10^{6}	6.920×10^{-2}	31.5	13.80	0.77	0.40	0.09	0.14	0.12	0.25	0.26
SP	SM_R4C6	1.77×10^{6}	6.385×10^{-2}	36.1	13.13	0.81	0.40	0.09	0.15	0.11	0.20	0.24
SP	SM_R4C7	1.51×10^{6}	6.039×10^{-2}	39.9	11.29	0.86	0.40	0.07	0.14	0.08	0.19	0.21
SP	SM_R4C8	1.60×10^{6}	6.238×10^{-2}	39.0	11.33	0.84	0.40	0.07	0.16	0.11	0.20	0.22
SP	SM_R4C9	1.59×10^{6}	6.015×10^{-2}	37.8	12.27	0.85	0.40	0.10	0.16	0.13	0.22	0.22
SP	SM_R5C1	2.34×10^{6}	7.259×10^{-2}	31.0	16.64	0.78	0.40	0.10	0.16	0.13	0.27	0.28
SP	SM_R5C2	1.98×10^{6}	6.678×10^{-2}	33.7	16.38	0.81	0.40	0.09	0.15	0.13	0.24	0.26
SP	SM_R5C3	1.68×10^{6}	6.138×10^{-2}	36.5	13.47	0.84	0.40	0.10	0.13	0.11	0.20	0.24
SP	SM_R5C4	1.76×10^{6}	6.368×10^{-2}	36.1	9.74	0.81	0.40	0.06	0.11	0.13	0.20	0.25
SP	SM_R5C5	1.55×10^{6}	6.230×10^{-2}	40.1	13.14	0.87	0.40	0.13	0.15	0.12	0.21	0.24
SP	SM_R5C6	1.42×10^{6}	6.025×10^{-2}	42.4	17.49	0.88	0.40	0.12	0.14	0.10	0.20	0.22
SP	SM_R5C7	1.25×10^{6}	5.610×10^{-2}	44.9	14.83	0.91	0.40	0.08	0.11	0.13	0.19	0.21
SP	SM_R5C8	1.23×10^{6}	5.270×10^{-2}	42.7	11.49	0.92	0.40	0.07	0.16	0.11	0.21	0.21
SP	SM_R5C9	1.13×10^{6}	5.139×10^{-2}	45.7	11.97	0.93	0.40	0.06	0.14	0.12	0.19	0.22
SP	SM_R6C1	2.38×10^{6}	6.852×10^{-2}	28.8	12.24	0.78	0.40	0.09	0.17	0.20	0.29	0.31
SP	SM_R6C2	1.83×10^{6}	6.162×10^{-2}	33.7	17.35	0.82	0.40	0.09	0.15	0.15	0.28	0.28
SP	SM_R6C3	1.61×10^{6}	5.916×10^{-2}	36.8	18.20	0.85	0.40	0.06	0.17	0.13	0.25	0.27
SP	SM_R6C4	1.50×10^{6}	5.533×10^{-2}	36.9	15.08	0.86	0.40	0.12	0.13	0.12	0.22	0.26
SP	SM_R6C5	1.45×10^{6}	5.328×10^{-2}	36.8	12.24	0.89	0.40	0.12	0.14	0.10	0.22	0.22
SP	SM_R6C6	1.36×10^{6}	5.281×10^{-2}	38.9	15.81	0.89	0.40	0.10	0.15	0.10	0.19	0.21
SP	SM_R6C7	1.14×10^{6}	5.018×10^{-2}	43.9	20.10	0.94	0.40	0.11	0.16	0.12	0.20	0.21
SP	SM_R6C8	1.04×10^{6}	4.837×10^{-2}	46.5	18.77	0.95	0.40	0.07	0.13	0.09	0.18	0.20
SP	SM_R6C9	9.83×10^{5}	4.503×10^{-2}	45.8	13.59	0.95	0.40	0.08	0.14	0.08	0.19	0.21
**Variant b (12 sensors)**												
MP-1	SM_R1C4	1.91×10^{5}	3.594×10^{-2}	188.1	219.56	1.41	0.00	0.02	0.08	0.28	0.23	0.21
MP-1	SM_R4C1	3.36×10^{5}	4.329×10^{-2}	128.7	110.66	1.29	0.00	0.06	0.23	0.43	0.33	0.29
MP-1	SM_R6C6	2.27×10^{5}	3.861×10^{-2}	169.8	84.26	1.39	0.00	0.00	0.11	0.33	0.28	0.24
MP-2	SM_R1C4	1.58×10^{5}	2.989×10^{-2}	188.8	139.49	1.42	0.13	0.09	0.09	0.41	0.38	0.28
MP-2	SM_R4C1	2.68×10^{5}	3.935×10^{-2}	146.8	102.53	1.36	0.00	0.04	0.14	0.55	0.40	0.32
MP-2	SM_R6C6	1.67×10^{5}	3.510×10^{-2}	210.2	41.19	1.49	0.00	0.01	0.08	0.38	0.42	0.28
MP-3	SM_R1C4	1.66×10^{5}	2.849×10^{-2}	171.4	135.22	1.40	0.00	0.01	0.10	0.39	0.37	0.29
MP-3	SM_R4C1	2.84×10^{5}	3.549×10^{-2}	125.2	98.32	1.32	0.23	0.09	0.19	0.56	0.46	0.35
MP-3	SM_R6C6	1.76×10^{5}	3.401×10^{-2}	193.1	41.71	1.46	0.08	0.04	0.09	0.34	0.40	0.28
MP-4	SM_R1C4	1.84×10^{5}	2.823×10^{-2}	153.2	126.40	1.31	0.00	0.04	0.09	0.41	0.36	0.34
MP-4	SM_R4C1	2.82×10^{5}	3.248×10^{-2}	115.0	94.74	1.25	0.07	0.12	0.20	0.57	0.45	0.41
MP-4	SM_R6C6	1.62×10^{5}	3.239×10^{-2}	199.5	42.50	1.37	0.09	0.05	0.09	0.33	0.32	0.35

Kinetic parameters for compound 4. SP = singleplex, MP-1/2/3/4 = multiplexed chip SCK-1/2/3/4. $f_{s,i}$ = non-dissociating fraction for cycle i.

Compound 5

Source	Sensor	k_a ($M^{-1}s^{-1}$)	k_d (s^{-1})	K_D (nM)	R_{max} (pg/mm^2)	$R_{max,n}$	$f_{s,1}$	$f_{s,2}$	$f_{s,3}$	$f_{s,4}$	$f_{s,5}$	$f_{s,6}$
**Variant a (54 sensors)**												
SP	SM_R1C1	5.03×10^{5}	4.491×10^{-2}	89.2	9.17	1.18	0.00	0.09	0.12	0.20	0.19	0.11
SP	SM_R1C2	5.46×10^{5}	4.642×10^{-2}	85.0	14.97	1.20	0.19	0.23	0.18	0.22	0.26	0.15
SP	SM_R1C3	4.74×10^{5}	5.044×10^{-2}	106.3	24.75	1.24	0.20	0.18	0.15	0.20	0.24	0.19
SP	SM_R1C4	4.86×10^{5}	5.088×10^{-2}	104.6	19.69	1.23	0.19	0.21	0.17	0.21	0.24	0.21
SP	SM_R1C5	4.86×10^{5}	5.029×10^{-2}	103.5	17.98	1.23	0.09	0.13	0.14	0.20	0.22	0.18
SP	SM_R1C6	4.97×10^{5}	5.034×10^{-2}	101.3	20.04	1.23	0.16	0.19	0.17	0.20	0.24	0.18
SP	SM_R1C7	4.74×10^{5}	4.874×10^{-2}	102.9	19.73	1.23	0.14	0.18	0.16	0.20	0.24	0.19
SP	SM_R1C8	4.77×10^{5}	4.953×10^{-2}	103.9	18.05	1.23	0.16	0.16	0.15	0.19	0.24	0.20
SP	SM_R1C9	4.84×10^{5}	4.709×10^{-2}	97.2	14.92	1.22	0.24	0.22	0.19	0.22	0.26	0.20
SP	SM_R2C1	5.18×10^{5}	5.043×10^{-2}	97.3	18.15	1.23	0.13	0.20	0.18	0.21	0.26	0.16
SP	SM_R2C2	4.95×10^{5}	5.132×10^{-2}	103.7	21.53	1.24	0.19	0.19	0.17	0.18	0.24	0.17
SP	SM_R2C3	4.93×10^{5}	5.084×10^{-2}	103.2	24.13	1.25	0.11	0.15	0.14	0.19	0.23	0.17
SP	SM_R2C4	5.17×10^{5}	5.100×10^{-2}	98.6	22.07	1.23	0.22	0.20	0.16	0.20	0.25	0.19
SP	SM_R2C5	4.58×10^{5}	4.997×10^{-2}	109.0	18.20	1.25	0.09	0.13	0.12	0.16	0.22	0.17
SP	SM_R2C6	4.53×10^{5}	5.173×10^{-2}	114.2	18.50	1.26	0.10	0.14	0.12	0.17	0.23	0.18
SP	SM_R2C7	4.14×10^{5}	4.964×10^{-2}	119.8	21.44	1.26	0.08	0.13	0.12	0.15	0.21	0.19
SP	SM_R2C8	4.23×10^{5}	5.086×10^{-2}	120.2	21.75	1.27	0.07	0.14	0.12	0.15	0.20	0.18
SP	SM_R2C9	4.24×10^{5}	5.048×10^{-2}	119.1	19.11	1.25	0.09	0.15	0.14	0.15	0.20	0.17
SP	SM_R3C1	4.95×10^{5}	4.655×10^{-2}	94.1	19.91	1.22	0.12	0.19	0.15	0.21	0.26	0.16
SP	SM_R3C2	4.59×10^{5}	4.600×10^{-2}	100.2	17.50	1.24	0.13	0.14	0.14	0.19	0.23	0.17
SP	SM_R3C3	4.53×10^{5}	4.693×10^{-2}	103.5	21.75	1.24	0.05	0.12	0.12	0.19	0.22	0.17
SP	SM_R3C4	4.32×10^{5}	4.491×10^{-2}	103.9	24.54	1.24	0.09	0.15	0.13	0.19	0.22	0.17
SP	SM_R3C5	4.65×10^{5}	4.603×10^{-2}	99.0	21.15	1.22	0.13	0.16	0.14	0.19	0.20	0.17
SP	SM_R3C6	4.34×10^{5}	4.798×10^{-2}	110.5	16.93	1.25	0.02	0.09	0.10	0.17	0.21	0.19
SP	SM_R3C7	4.43×10^{5}	4.644×10^{-2}	104.9	17.27	1.22	0.26	0.23	0.17	0.17	0.22	0.21
SP	SM_R3C8	4.44×10^{5}	4.784×10^{-2}	107.8	18.07	1.23	0.12	0.17	0.14	0.18	0.20	0.16
SP	SM_R3C9	4.39×10^{5}	4.914×10^{-2}	112.0	19.92	1.24	0.12	0.18	0.13	0.15	0.22	0.17
SP	SM_R4C1	4.67×10^{5}	4.162×10^{-2}	89.1	24.40	1.22	0.14	0.18	0.17	0.23	0.26	0.17
SP	SM_R4C2	4.68×10^{5}	4.449×10^{-2}	95.1	18.95	1.23	0.15	0.18	0.17	0.20	0.24	0.17
SP	SM_R4C3	4.33×10^{5}	4.419×10^{-2}	102.0	16.09	1.21	0.08	0.17	0.15	0.18	0.23	0.16
SP	SM_R4C4	4.39×10^{5}	4.567×10^{-2}	104.0	17.08	1.24	0.00	0.09	0.09	0.18	0.25	0.17
SP	SM_R4C5	4.19×10^{5}	4.570×10^{-2}	108.9	21.49	1.24	0.11	0.14	0.13	0.16	0.23	0.18
SP	SM_R4C6	4.17×10^{5}	4.412×10^{-2}	105.9	18.30	1.23	0.04	0.13	0.11	0.17	0.22	0.16
SP	SM_R4C7	4.28×10^{5}	4.293×10^{-2}	100.2	15.56	1.22	0.24	0.23	0.17	0.18	0.21	0.21
SP	SM_R4C8	4.02×10^{5}	4.195×10^{-2}	104.3	15.55	1.24	0.12	0.15	0.11	0.18	0.19	0.17
SP	SM_R4C9	3.98×10^{5}	4.434×10^{-2}	111.5	17.51	1.26	0.07	0.13	0.10	0.16	0.20	0.17
SP	SM_R5C1	4.40×10^{5}	4.035×10^{-2}	91.6	25.38	1.22	0.16	0.19	0.17	0.23	0.25	0.18
SP	SM_R5C2	4.21×10^{5}	4.190×10^{-2}	99.5	24.79	1.24	0.14	0.17	0.16	0.20	0.24	0.19
SP	SM_R5C3	4.21×10^{5}	4.307×10^{-2}	102.4	19.18	1.23	0.11	0.17	0.15	0.19	0.24	0.18
SP	SM_R5C4	4.07×10^{5}	4.128×10^{-2}	101.3	13.97	1.20	0.09	0.15	0.13	0.21	0.21	0.17
SP	SM_R5C5	3.87×10^{5}	4.167×10^{-2}	107.7	17.27	1.24	0.06	0.14	0.14	0.17	0.22	0.15
SP	SM_R5C6	3.87×10^{5}	4.203×10^{-2}	108.5	22.86	1.24	0.10	0.17	0.13	0.19	0.20	0.16
SP	SM_R5C7	3.74×10^{5}	4.100×10^{-2}	109.7	19.56	1.25	0.10	0.15	0.12	0.17	0.18	0.17
SP	SM_R5C8	3.86×10^{5}	4.125×10^{-2}	106.9	15.65	1.24	0.16	0.18	0.14	0.17	0.22	0.19
SP	SM_R5C9	3.50×10^{5}	4.059×10^{-2}	115.8	16.45	1.25	0.09	0.14	0.12	0.15	0.19	0.16
SP	SM_R6C1	3.96×10^{5}	4.030×10^{-2}	101.7	18.44	1.22	0.00	0.12	0.12	0.20	0.23	0.11
SP	SM_R6C2	4.24×10^{5}	4.098×10^{-2}	96.6	25.50	1.22	0.10	0.14	0.14	0.20	0.25	0.15
SP	SM_R6C3	4.22×10^{5}	4.048×10^{-2}	96.0	25.40	1.22	0.09	0.16	0.15	0.20	0.24	0.16
SP	SM_R6C4	3.75×10^{5}	3.952×10^{-2}	105.4	20.95	1.24	0.05	0.12	0.11	0.19	0.22	0.15
SP	SM_R6C5	4.01×10^{5}	3.842×10^{-2}	95.9	16.47	1.22	0.11	0.18	0.14	0.19	0.22	0.17
SP	SM_R6C6	3.87×10^{5}	3.910×10^{-2}	100.9	21.69	1.21	0.14	0.18	0.15	0.19	0.21	0.17
SP	SM_R6C7	3.74×10^{5}	3.964×10^{-2}	106.1	26.43	1.24	0.09	0.15	0.14	0.18	0.21	0.17
SP	SM_R6C8	3.68×10^{5}	3.979×10^{-2}	108.3	24.30	1.25	0.16	0.18	0.13	0.18	0.20	0.18
SP	SM_R6C9	3.86×10^{5}	4.236×10^{-2}	109.6	17.20	1.21	0.07	0.15	0.12	0.16	0.22	0.17
**Variant b (12 sensors)**												
MP-1	SM_R1C5	1.43×10^{5}	4.185×10^{-2}	292.4	198.18	1.58	0.00	0.06	0.13	0.29	0.29	0.27
MP-1	SM_R4C2	2.37×10^{5}	4.792×10^{-2}	202.4	105.07	1.43	0.00	0.04	0.19	0.41	0.39	0.33
MP-1	SM_R6C7	1.65×10^{5}	4.559×10^{-2}	276.2	51.97	1.55	0.07	0.11	0.15	0.30	0.35	0.29
MP-2	SM_R1C5	1.36×10^{5}	3.342×10^{-2}	245.0	103.46	1.50	0.00	0.07	0.11	0.37	0.43	0.32
MP-2	SM_R4C2	1.91×10^{5}	4.387×10^{-2}	230.1	75.28	1.50	0.00	0.12	0.16	0.46	0.48	0.38
MP-2	SM_R6C7	1.29×10^{5}	4.383×10^{-2}	338.8	37.73	1.72	0.00	0.04	0.10	0.39	0.51	0.36
MP-3	SM_R1C5	1.46×10^{5}	3.121×10^{-2}	214.4	94.75	1.44	0.11	0.05	0.15	0.39	0.48	0.37
MP-3	SM_R4C2	2.07×10^{5}	3.851×10^{-2}	185.8	73.00	1.40	0.00	0.10	0.20	0.47	0.51	0.39
MP-3	SM_R6C7	1.60×10^{5}	3.966×10^{-2}	247.9	33.26	1.56	0.33	0.03	0.12	0.32	0.51	0.35
MP-4	SM_R1C5	1.41×10^{5}	3.033×10^{-2}	215.3	97.50	1.37	0.00	0.08	0.15	0.39	0.41	0.43
MP-4	SM_R4C2	2.10×10^{5}	3.547×10^{-2}	168.7	68.86	1.30	0.14	0.16	0.22	0.48	0.47	0.46
MP-4	SM_R6C7	1.32×10^{5}	3.489×10^{-2}	264.0	35.96	1.43	0.03	0.09	0.13	0.33	0.39	0.43

Kinetic parameters for compound 5. SP = singleplex, MP-1/2/3/4 = multiplexed chip SCK-1/2/3/4. $f_{s,i}$ = non-dissociating fraction for cycle i.

Compound 6

Source	Sensor	k_a ($M^{-1}s^{-1}$)	k_d (s^{-1})	K_D (nM)	R_{max} (pg/mm^2)	$R_{max,n}$	$f_{s,1}$	$f_{s,2}$	$f_{s,3}$	$f_{s,4}$	$f_{s,5}$	$f_{s,6}$
**Variant a (54 sensors)**												
SP	SM_R1C1	7.60×10^{5}	1.799×10^{-2}	23.7	4.95	0.92	0.11	0.06	0.19	0.32	0.27	0.08
SP	SM_R1C2	8.30×10^{5}	2.170×10^{-2}	26.2	7.39	0.96	0.30	0.14	0.29	0.40	0.35	0.18
SP	SM_R1C3	8.54×10^{5}	2.451×10^{-2}	28.7	11.44	0.98	0.26	0.22	0.28	0.41	0.39	0.20
SP	SM_R1C4	8.24×10^{5}	2.435×10^{-2}	29.5	9.49	1.00	0.28	0.18	0.28	0.41	0.39	0.21
SP	SM_R1C5	9.15×10^{5}	2.645×10^{-2}	28.9	8.10	0.96	0.26	0.26	0.28	0.42	0.40	0.19
SP	SM_R1C6	9.09×10^{5}	2.465×10^{-2}	27.1	10.43	1.01	0.32	0.23	0.32	0.45	0.41	0.24
SP	SM_R1C7	8.09×10^{5}	2.282×10^{-2}	28.2	10.24	0.98	0.28	0.21	0.27	0.40	0.38	0.21
SP	SM_R1C8	8.13×10^{5}	2.457×10^{-2}	30.2	8.65	1.01	0.19	0.14	0.25	0.39	0.36	0.20
SP	SM_R1C9	8.80×10^{5}	2.546×10^{-2}	28.9	7.50	1.00	0.28	0.24	0.29	0.43	0.39	0.23
SP	SM_R2C1	8.49×10^{5}	2.396×10^{-2}	28.2	11.01	0.98	0.26	0.20	0.30	0.44	0.41	0.23
SP	SM_R2C2	7.87×10^{5}	2.234×10^{-2}	28.4	13.08	0.98	0.32	0.20	0.28	0.42	0.40	0.24
SP	SM_R2C3	8.39×10^{5}	2.430×10^{-2}	29.0	14.55	0.97	0.28	0.19	0.28	0.42	0.40	0.21
SP	SM_R2C4	8.23×10^{5}	2.343×10^{-2}	28.5	13.47	1.00	0.33	0.21	0.31	0.40	0.41	0.23
SP	SM_R2C5	8.22×10^{5}	2.296×10^{-2}	27.9	10.42	1.00	0.24	0.20	0.29	0.39	0.38	0.22
SP	SM_R2C6	6.93×10^{5}	2.101×10^{-2}	30.3	10.21	0.97	0.29	0.12	0.26	0.39	0.37	0.22
SP	SM_R2C7	7.71×10^{5}	2.369×10^{-2}	30.7	11.90	0.98	0.26	0.17	0.28	0.39	0.41	0.24
SP	SM_R2C8	7.49×10^{5}	2.532×10^{-2}	33.8	11.87	0.99	0.19	0.17	0.26	0.37	0.40	0.25
SP	SM_R2C9	6.77×10^{5}	2.495×10^{-2}	36.8	10.50	1.00	0.17	0.14	0.24	0.38	0.39	0.26
SP	SM_R3C1	7.90×10^{5}	2.161×10^{-2}	27.4	11.40	0.99	0.31	0.16	0.29	0.42	0.39	0.22
SP	SM_R3C2	7.96×10^{5}	2.226×10^{-2}	28.0	9.92	0.98	0.26	0.20	0.28	0.40	0.38	0.23
SP	SM_R3C3	7.31×10^{5}	2.180×10^{-2}	29.8	11.89	0.98	0.26	0.18	0.28	0.39	0.39	0.20
SP	SM_R3C4	7.76×10^{5}	2.210×10^{-2}	28.5	13.77	1.00	0.31	0.17	0.29	0.40	0.39	0.23
SP	SM_R3C5	7.50×10^{5}	2.315×10^{-2}	30.9	11.83	0.99	0.29	0.22	0.27	0.40	0.39	0.22
SP	SM_R3C6	7.67×10^{5}	2.334×10^{-2}	30.4	9.89	0.98	0.27	0.15	0.26	0.38	0.39	0.23
SP	SM_R3C7	7.40×10^{5}	2.269×10^{-2}	30.7	10.02	1.00	0.44	0.21	0.29	0.41	0.42	0.27
SP	SM_R3C8	7.47×10^{5}	2.296×10^{-2}	30.7	10.02	0.99	0.33	0.22	0.29	0.39	0.40	0.24
SP	SM_R3C9	7.34×10^{5}	2.405×10^{-2}	32.8	11.03	1.01	0.32	0.18	0.28	0.39	0.39	0.24
SP	SM_R4C1	7.89×10^{5}	2.055×10^{-2}	26.1	14.57	0.98	0.30	0.21	0.31	0.42	0.39	0.22
SP	SM_R4C2	7.41×10^{5}	2.210×10^{-2}	29.8	11.73	1.01	0.29	0.18	0.27	0.39	0.38	0.23
SP	SM_R4C3	7.71×10^{5}	2.047×10^{-2}	26.5	9.52	0.99	0.38	0.22	0.29	0.40	0.40	0.23
SP	SM_R4C4	7.83×10^{5}	2.009×10^{-2}	25.7	10.34	0.97	0.29	0.16	0.27	0.38	0.35	0.19
SP	SM_R4C5	7.74×10^{5}	2.014×10^{-2}	26.0	12.57	0.98	0.26	0.16	0.28	0.38	0.35	0.21
SP	SM_R4C6	6.93×10^{5}	1.886×10^{-2}	27.2	11.00	0.96	0.25	0.14	0.24	0.35	0.35	0.20
SP	SM_R4C7	7.10×10^{5}	2.273×10^{-2}	32.0	8.17	1.02	0.30	0.14	0.25	0.38	0.38	0.21
SP	SM_R4C8	7.29×10^{5}	2.158×10^{-2}	29.6	8.16	1.02	0.35	0.18	0.27	0.38	0.39	0.25
SP	SM_R4C9	6.91×10^{5}	2.223×10^{-2}	32.2	9.71	1.01	0.35	0.19	0.26	0.38	0.38	0.23
SP	SM_R5C1	7.47×10^{5}	2.304×10^{-2}	30.8	16.00	1.03	0.28	0.19	0.28	0.41	0.38	0.21
SP	SM_R5C2	6.87×10^{5}	2.319×10^{-2}	33.8	15.25	1.03	0.25	0.15	0.25	0.38	0.38	0.22
SP	SM_R5C3	6.76×10^{5}	2.137×10^{-2}	31.6	11.25	0.99	0.26	0.17	0.26	0.38	0.39	0.21
SP	SM_R5C4	7.33×10^{5}	2.136×10^{-2}	29.2	8.68	1.04	0.35	0.20	0.29	0.39	0.35	0.22
SP	SM_R5C5	6.65×10^{5}	2.040×10^{-2}	30.7	10.76	1.01	0.20	0.20	0.24	0.36	0.36	0.20
SP	SM_R5C6	6.64×10^{5}	1.997×10^{-2}	30.1	13.36	1.02	0.21	0.13	0.23	0.34	0.33	0.20
SP	SM_R5C7	6.89×10^{5}	2.062×10^{-2}	29.9	10.99	1.02	0.35	0.19	0.27	0.36	0.36	0.21
SP	SM_R5C8	6.69×10^{5}	1.969×10^{-2}	29.4	8.79	1.01	0.32	0.19	0.23	0.35	0.35	0.21
SP	SM_R5C9	6.80×10^{5}	2.054×10^{-2}	30.2	9.46	1.01	0.28	0.19	0.25	0.36	0.36	0.20
SP	SM_R6C1	6.99×10^{5}	2.307×10^{-2}	33.0	11.70	1.00	0.25	0.17	0.29	0.40	0.39	0.22
SP	SM_R6C2	6.38×10^{5}	1.995×10^{-2}	31.3	15.52	1.00	0.25	0.14	0.25	0.38	0.37	0.22
SP	SM_R6C3	6.56×10^{5}	1.964×10^{-2}	29.9	14.34	0.98	0.21	0.12	0.24	0.36	0.36	0.20
SP	SM_R6C4	6.70×10^{5}	2.041×10^{-2}	30.5	10.97	1.01	0.27	0.14	0.24	0.36	0.37	0.22
SP	SM_R6C5	7.08×10^{5}	2.147×10^{-2}	30.3	8.89	1.01	0.43	0.15	0.28	0.38	0.37	0.24
SP	SM_R6C6	6.31×10^{5}	2.212×10^{-2}	35.1	11.47	1.04	0.29	0.12	0.24	0.35	0.37	0.22
SP	SM_R6C7	6.37×10^{5}	2.153×10^{-2}	33.8	14.01	1.03	0.22	0.16	0.23	0.34	0.35	0.19
SP	SM_R6C8	6.43×10^{5}	2.090×10^{-2}	32.5	12.71	1.02	0.28	0.15	0.22	0.32	0.34	0.21
SP	SM_R6C9	6.55×10^{5}	1.889×10^{-2}	28.8	9.03	0.98	0.35	0.21	0.28	0.36	0.37	0.22
**Variant b (12 sensors)**												
MP-1	SM_R1C6	1.18×10^{5}	5.027×10^{-2}	426.0	98.41	1.86	0.00	0.00	0.15	0.24	0.34	0.27
MP-1	SM_R4C3	1.61×10^{5}	5.228×10^{-2}	324.5	46.72	1.68	0.00	0.00	0.20	0.31	0.44	0.29
MP-1	SM_R6C8	1.53×10^{5}	5.073×10^{-2}	332.4	37.87	1.69	0.00	0.04	0.18	0.27	0.43	0.28
MP-2	SM_R1C6	1.44×10^{5}	4.027×10^{-2}	280.4	48.51	1.60	0.00	0.00	0.12	0.38	0.50	0.31
MP-2	SM_R4C3	1.61×10^{5}	5.614×10^{-2}	348.1	30.83	1.72	0.00	0.00	0.13	0.38	0.55	0.34
MP-2	SM_R6C8	1.41×10^{5}	4.899×10^{-2}	348.5	25.29	1.74	0.00	0.01	0.16	0.35	0.62	0.39
MP-3	SM_R1C6	1.59×10^{5}	3.340×10^{-2}	210.7	42.78	1.47	0.22	0.26	0.29	0.40	0.57	0.32
MP-3	SM_R4C3	2.06×10^{5}	4.572×10^{-2}	222.3	26.06	1.48	0.10	0.28	0.33	0.39	0.64	0.34
MP-3	SM_R6C8	1.70×10^{5}	3.943×10^{-2}	232.2	20.83	1.50	0.00	0.15	0.25	0.33	0.67	0.32
MP-4	SM_R1C6	1.65×10^{5}	3.584×10^{-2}	217.8	44.73	1.35	0.14	0.21	0.23	0.41	0.49	0.41
MP-4	SM_R4C3	1.76×10^{5}	3.649×10^{-2}	207.3	27.70	1.30	0.53	0.34	0.32	0.42	0.50	0.43
MP-4	SM_R6C8	1.33×10^{5}	3.223×10^{-2}	242.3	23.74	1.34	0.36	0.27	0.28	0.39	0.48	0.43

Kinetic parameters for compound 6. SP = singleplex, MP-1/2/3/4 = multiplexed chip SCK-1/2/3/4. $f_{s,i}$ = non-dissociating fraction for cycle i.

Compound 7

Source	Sensor	k_a ($M^{-1}s^{-1}$)	k_d (s^{-1})	K_D (nM)	R_{max} (pg/mm^2)	$R_{max,n}$	$f_{s,1}$	$f_{s,2}$	$f_{s,3}$	$f_{s,4}$	$f_{s,5}$	$f_{s,6}$
**Variant a (54 sensors)**												
SP	SM_R1C1	4.76×10^5	4.175×10^{-2}	87.6	7.14	1.16	0.16	0.31	0.24	0.32	0.19	0.12
SP	SM_R1C2	4.81×10^5	4.198×10^{-2}	87.3	11.85	1.15	0.35	0.29	0.26	0.33	0.21	0.17
SP	SM_R1C3	4.55×10^5	5.154×10^{-2}	113.2	19.63	1.20	0.18	0.22	0.22	0.29	0.20	0.17
SP	SM_R1C4	4.39×10^5	4.963×10^{-2}	113.0	15.32	1.20	0.15	0.27	0.25	0.31	0.20	0.18
SP	SM_R1C5	4.68×10^5	5.183×10^{-2}	110.7	13.71	1.23	0.11	0.20	0.21	0.27	0.19	0.15
SP	SM_R1C6	4.53×10^5	5.285×10^{-2}	116.7	15.55	1.23	0.23	0.19	0.22	0.30	0.21	0.16
SP	SM_R1C7	4.38×10^5	4.867×10^{-2}	111.1	15.39	1.23	0.28	0.22	0.23	0.30	0.21	0.16
SP	SM_R1C8	4.56×10^5	5.127×10^{-2}	112.4	13.07	1.21	0.00	0.21	0.21	0.28	0.13	0.13
SP	SM_R1C9	4.00×10^5	4.897×10^{-2}	122.5	11.28	1.23	0.03	0.14	0.22	0.28	0.14	0.11
SP	SM_R2C1	4.67×10^5	4.884×10^{-2}	104.5	14.18	1.21	0.23	0.22	0.27	0.33	0.21	0.15
SP	SM_R2C2	4.49×10^5	5.131×10^{-2}	114.2	17.02	1.22	0.15	0.25	0.24	0.32	0.21	0.17
SP	SM_R2C3	4.75×10^5	5.007×10^{-2}	105.5	18.47	1.21	0.22	0.27	0.24	0.31	0.22	0.17
SP	SM_R2C4	4.74×10^5	4.893×10^{-2}	103.3	16.42	1.22	0.19	0.22	0.24	0.31	0.21	0.16
SP	SM_R2C5	4.78×10^5	5.387×10^{-2}	112.8	13.43	1.24	0.09	0.20	0.21	0.28	0.20	0.17
SP	SM_R2C6	4.50×10^5	5.280×10^{-2}	117.2	13.43	1.24	0.00	0.18	0.19	0.26	0.18	0.16
SP	SM_R2C7	4.54×10^5	5.605×10^{-2}	123.4	15.47	1.25	0.08	0.21	0.21	0.27	0.19	0.18
SP	SM_R2C8	4.47×10^5	5.279×10^{-2}	118.1	15.78	1.22	0.24	0.25	0.24	0.28	0.19	0.18
SP	SM_R2C9	4.34×10^5	5.302×10^{-2}	122.0	14.30	1.23	0.04	0.22	0.19	0.26	0.16	0.16
SP	SM_R3C1	4.82×10^5	4.716×10^{-2}	97.8	15.46	1.19	0.20	0.26	0.27	0.33	0.21	0.15
SP	SM_R3C2	4.62×10^5	5.056×10^{-2}	109.4	13.59	1.21	0.15	0.26	0.25	0.31	0.22	0.18
SP	SM_R3C3	4.17×10^5	4.956×10^{-2}	118.9	16.30	1.25	0.05	0.18	0.20	0.29	0.21	0.16
SP	SM_R3C4	4.33×10^5	4.668×10^{-2}	107.8	18.50	1.22	0.15	0.24	0.24	0.29	0.23	0.17
SP	SM_R3C5	4.47×10^5	4.670×10^{-2}	104.4	16.17	1.20	0.19	0.21	0.23	0.28	0.23	0.17
SP	SM_R3C6	4.71×10^5	5.716×10^{-2}	121.4	13.00	1.26	0.12	0.23	0.20	0.30	0.26	0.21
SP	SM_R3C7	4.29×10^5	4.887×10^{-2}	113.9	13.32	1.21	0.15	0.25	0.25	0.29	0.21	0.19
SP	SM_R3C8	4.42×10^5	4.912×10^{-2}	111.1	13.94	1.21	0.13	0.25	0.22	0.28	0.21	0.19
SP	SM_R3C9	4.07×10^5	5.029×10^{-2}	123.6	15.12	1.24	0.05	0.20	0.20	0.26	0.19	0.17
SP	SM_R4C1	4.30×10^5	4.461×10^{-2}	103.7	20.13	1.22	0.20	0.27	0.27	0.33	0.23	0.16
SP	SM_R4C2	4.41×10^5	4.742×10^{-2}	107.6	15.37	1.22	0.19	0.25	0.25	0.30	0.22	0.17
SP	SM_R4C3	4.31×10^5	4.854×10^{-2}	112.6	12.55	1.21	0.10	0.24	0.23	0.30	0.23	0.19
SP	SM_R4C4	4.16×10^5	4.816×10^{-2}	115.7	13.28	1.24	0.15	0.25	0.23	0.30	0.24	0.17
SP	SM_R4C5	4.05×10^5	4.713×10^{-2}	116.4	16.22	1.25	0.11	0.20	0.22	0.27	0.22	0.18
SP	SM_R4C6	4.36×10^5	5.127×10^{-2}	117.5	14.44	1.25	0.10	0.23	0.20	0.28	0.23	0.19
SP	SM_R4C7	3.97×10^5	4.446×10^{-2}	111.9	11.96	1.23	0.29	0.22	0.21	0.28	0.23	0.19
SP	SM_R4C8	3.98×10^5	4.509×10^{-2}	113.4	11.97	1.23	0.06	0.24	0.21	0.27	0.21	0.18
SP	SM_R4C9	3.75×10^5	4.938×10^{-2}	131.6	13.39	1.28	0.00	0.14	0.19	0.26	0.22	0.17
SP	SM_R5C1	4.63×10^5	4.741×10^{-2}	102.5	21.01	1.21	0.24	0.31	0.29	0.35	0.24	0.19
SP	SM_R5C2	4.13×10^5	4.682×10^{-2}	113.5	20.18	1.22	0.22	0.28	0.25	0.31	0.24	0.19
SP	SM_R5C3	4.40×10^5	4.889×10^{-2}	111.0	15.34	1.22	0.23	0.28	0.27	0.32	0.25	0.20
SP	SM_R5C4	4.45×10^5	4.660×10^{-2}	104.6	11.23	1.20	0.21	0.30	0.27	0.32	0.23	0.19
SP	SM_R5C5	3.79×10^5	4.809×10^{-2}	126.9	14.36	1.26	0.06	0.21	0.21	0.27	0.22	0.19
SP	SM_R5C6	4.17×10^5	4.900×10^{-2}	117.4	18.23	1.25	0.21	0.25	0.23	0.29	0.25	0.20
SP	SM_R5C7	4.16×10^5	4.513×10^{-2}	108.4	15.20	1.21	0.25	0.24	0.21	0.27	0.23	0.19
SP	SM_R5C8	4.28×10^5	4.928×10^{-2}	115.2	12.12	1.21	0.23	0.35	0.26	0.28	0.25	0.22
SP	SM_R5C9	3.59×10^5	4.685×10^{-2}	130.4	13.03	1.26	0.16	0.18	0.19	0.25	0.22	0.21
SP	SM_R6C1	2.92×10^5	4.500×10^{-2}	154.1	16.66	1.31	0.05	0.10	0.18	0.25	0.23	0.16
SP	SM_R6C2	3.54×10^5	4.589×10^{-2}	129.8	21.82	1.28	0.03	0.19	0.21	0.29	0.22	0.18
SP	SM_R6C3	3.76×10^5	4.528×10^{-2}	120.5	20.96	1.25	0.16	0.25	0.26	0.30	0.25	0.19
SP	SM_R6C4	3.83×10^5	4.538×10^{-2}	118.4	16.64	1.25	0.13	0.22	0.21	0.27	0.23	0.18
SP	SM_R6C5	3.50×10^5	4.335×10^{-2}	123.9	13.76	1.24	0.21	0.23	0.23	0.29	0.24	0.20
SP	SM_R6C6	3.70×10^5	4.401×10^{-2}	119.0	17.56	1.23	0.17	0.26	0.23	0.29	0.22	0.20
SP	SM_R6C7	3.79×10^5	4.649×10^{-2}	122.6	20.97	1.25	0.20	0.26	0.24	0.30	0.24	0.21
SP	SM_R6C8	3.49×10^5	4.304×10^{-2}	123.4	18.73	1.25	0.15	0.27	0.23	0.28	0.23	0.19
SP	SM_R6C9	3.62×10^5	4.691×10^{-2}	129.5	13.18	1.27	0.00	0.21	0.22	0.27	0.23	0.18
**Variant b (12 sensors)**												
MP-1	SM_R1C7	9.70×10^4	3.394×10^{-2}	350.0	21.57	1.71	0.00	0.00	0.00	0.35	0.46	0.24
MP-1	SM_R4C4	2.32×10^5	4.239×10^{-2}	182.8	59.73	1.38	0.03	0.10	0.20	0.35	0.33	0.27
MP-1	SM_R7C1	3.66×10^5	4.447×10^{-2}	121.4	49.76	1.24	0.07	0.25	0.44	0.46	0.47	0.31
MP-2	SM_R1C7	1.74×10^5	3.565×10^{-2}	204.6	69.91	1.45	0.04	0.12	0.16	0.38	0.46	0.35
MP-2	SM_R4C4	1.82×10^5	3.779×10^{-2}	207.4	43.97	1.46	0.00	0.10	0.13	0.42	0.45	0.34
MP-2	SM_R7C1	2.94×10^5	4.616×10^{-2}	156.9	40.57	1.36	0.00	0.16	0.31	0.66	0.60	0.39
MP-3	SM_R1C7	2.01×10^5	3.309×10^{-2}	164.6	63.00	1.37	0.11	0.18	0.21	0.41	0.50	0.38
MP-3	SM_R4C4	2.05×10^5	3.800×10^{-2}	185.2	45.88	1.42	0.11	0.06	0.17	0.41	0.48	0.36
MP-3	SM_R7C1	3.54×10^5	4.185×10^{-2}	118.1	38.82	1.19	0.00	0.19	0.35	0.65	0.66	0.39
MP-4	SM_R1C7	1.98×10^5	3.317×10^{-2}	167.7	63.24	1.30	0.06	0.14	0.22	0.44	0.47	0.45
MP-4	SM_R4C4	1.94×10^5	3.501×10^{-2}	180.7	45.27	1.31	0.12	0.15	0.20	0.43	0.42	0.43
MP-4	SM_R7C1	3.10×10^5	3.312×10^{-2}	107.0	36.50	1.14	0.15	0.24	0.38	0.67	0.59	0.42

Kinetic parameters for compound 7. SP = singleplex, MP-1/2/3/4 = multiplexed chip SCK-1/2/3/4. $f_{s,i}$ = non-dissociating fraction for cycle i.

Compound 8

Source	Sensor	k_a ($M^{-1}s^{-1}$)	k_d (s^{-1})	K_D (nM)	R_{max} (pg/mm^2)	$R_{max,n}$	$f_{s,1}$	$f_{s,2}$	$f_{s,3}$	$f_{s,4}$	$f_{s,5}$	$f_{s,6}$
**Variant a (54 sensors)**												
SP	SM_R1C1	5.19×10^{5}	4.436×10^{-2}	85.4	6.56	1.15	0.13	0.03	0.22	0.22	0.21	0.08
SP	SM_R1C2	5.47×10^{5}	4.126×10^{-2}	75.4	10.72	1.15	0.35	0.09	0.29	0.28	0.27	0.12
SP	SM_R1C3	4.78×10^{5}	4.850×10^{-2}	101.5	17.80	1.21	0.18	0.12	0.23	0.27	0.26	0.13
SP	SM_R1C4	4.96×10^{5}	4.665×10^{-2}	94.0	14.46	1.18	0.29	0.15	0.26	0.28	0.27	0.14
SP	SM_R1C5	5.13×10^{5}	5.006×10^{-2}	97.5	13.89	1.21	0.27	0.09	0.25	0.28	0.29	0.16
SP	SM_R1C6	4.69×10^{5}	5.096×10^{-2}	108.7	16.00	1.25	0.15	0.00	0.23	0.26	0.28	0.14
SP	SM_R1C7	4.85×10^{5}	4.608×10^{-2}	95.0	15.80	1.20	0.25	0.10	0.26	0.28	0.28	0.16
SP	SM_R1C8	4.55×10^{5}	4.381×10^{-2}	96.3	13.11	1.19	0.20	0.04	0.25	0.26	0.25	0.10
SP	SM_R1C9	4.60×10^{5}	4.465×10^{-2}	97.1	10.66	1.19	0.23	0.07	0.26	0.27	0.25	0.12
SP	SM_R2C1	4.81×10^{5}	4.346×10^{-2}	90.3	14.03	1.20	0.17	0.03	0.27	0.29	0.27	0.10
SP	SM_R2C2	5.07×10^{5}	4.560×10^{-2}	89.9	16.84	1.18	0.28	0.12	0.27	0.27	0.27	0.13
SP	SM_R2C3	5.06×10^{5}	4.757×10^{-2}	94.0	18.99	1.22	0.22	0.08	0.24	0.25	0.25	0.12
SP	SM_R2C4	5.05×10^{5}	4.907×10^{-2}	97.2	17.17	1.23	0.16	0.04	0.23	0.25	0.26	0.13
SP	SM_R2C5	4.79×10^{5}	4.609×10^{-2}	96.2	13.55	1.23	0.15	0.12	0.21	0.25	0.23	0.13
SP	SM_R2C6	4.77×10^{5}	4.559×10^{-2}	95.5	13.24	1.22	0.20	0.13	0.17	0.25	0.27	0.13
SP	SM_R2C7	5.17×10^{5}	4.798×10^{-2}	92.7	14.06	1.22	0.27	0.10	0.25	0.28	0.29	0.16
SP	SM_R2C8	4.83×10^{5}	4.988×10^{-2}	103.2	14.91	1.22	0.26	0.13	0.24	0.27	0.26	0.16
SP	SM_R2C9	4.83×10^{5}	4.537×10^{-2}	94.0	12.94	1.22	0.39	0.20	0.27	0.27	0.26	0.16
SP	SM_R3C1	5.35×10^{5}	4.587×10^{-2}	85.8	14.93	1.22	0.28	0.11	0.28	0.29	0.28	0.12
SP	SM_R3C2	4.84×10^{5}	4.400×10^{-2}	90.9	12.93	1.21	0.25	0.09	0.25	0.27	0.27	0.13
SP	SM_R3C3	4.60×10^{5}	4.463×10^{-2}	97.0	15.36	1.24	0.11	0.09	0.22	0.24	0.24	0.12
SP	SM_R3C4	4.80×10^{5}	4.398×10^{-2}	91.6	17.29	1.22	0.18	0.14	0.23	0.26	0.26	0.13
SP	SM_R3C5	5.17×10^{5}	4.988×10^{-2}	96.5	14.77	1.23	0.12	0.08	0.21	0.26	0.27	0.15
SP	SM_R3C6	4.50×10^{5}	4.907×10^{-2}	109.1	12.50	1.24	0.00	0.00	0.17	0.21	0.25	0.14
SP	SM_R3C7	5.09×10^{5}	4.711×10^{-2}	92.6	12.03	1.21	0.23	0.13	0.23	0.27	0.28	0.18
SP	SM_R3C8	4.56×10^{5}	4.477×10^{-2}	98.2	12.83	1.22	0.14	0.17	0.21	0.25	0.24	0.13
SP	SM_R3C9	4.70×10^{5}	4.588×10^{-2}	97.7	13.93	1.22	0.24	0.20	0.23	0.26	0.26	0.16
SP	SM_R4C1	4.58×10^{5}	3.986×10^{-2}	87.0	19.52	1.21	0.22	0.12	0.26	0.28	0.26	0.12
SP	SM_R4C2	4.80×10^{5}	4.405×10^{-2}	91.8	14.89	1.23	0.21	0.13	0.24	0.27	0.26	0.13
SP	SM_R4C3	4.43×10^{5}	4.132×10^{-2}	93.3	12.35	1.20	0.19	0.10	0.23	0.25	0.26	0.13
SP	SM_R4C4	4.34×10^{5}	4.396×10^{-2}	101.3	13.02	1.21	0.08	0.02	0.20	0.24	0.24	0.11
SP	SM_R4C5	4.52×10^{5}	4.689×10^{-2}	103.7	15.81	1.21	0.12	0.06	0.21	0.25	0.26	0.15
SP	SM_R4C6	4.13×10^{5}	4.059×10^{-2}	98.3	14.27	1.18	0.16	0.12	0.20	0.22	0.24	0.15
SP	SM_R4C7	5.03×10^{5}	4.824×10^{-2}	96.0	10.71	1.22	0.28	0.11	0.22	0.27	0.29	0.18
SP	SM_R4C8	4.48×10^{5}	4.199×10^{-2}	93.6	10.77	1.21	0.12	0.22	0.19	0.24	0.24	0.14
SP	SM_R4C9	4.59×10^{5}	4.471×10^{-2}	97.4	12.99	1.22	0.23	0.13	0.23	0.25	0.25	0.17
SP	SM_R5C1	4.55×10^{5}	4.030×10^{-2}	88.7	20.54	1.21	0.26	0.17	0.27	0.31	0.29	0.16
SP	SM_R5C2	4.38×10^{5}	4.217×10^{-2}	96.2	19.24	1.23	0.19	0.15	0.24	0.29	0.27	0.17
SP	SM_R5C3	4.40×10^{5}	4.184×10^{-2}	95.0	14.68	1.19	0.25	0.08	0.25	0.27	0.29	0.18
SP	SM_R5C4	4.81×10^{5}	4.431×10^{-2}	92.1	10.71	1.22	0.22	0.14	0.24	0.28	0.28	0.17
SP	SM_R5C5	3.99×10^{5}	3.812×10^{-2}	95.4	13.94	1.18	0.20	0.14	0.22	0.26	0.26	0.16
SP	SM_R5C6	4.18×10^{5}	4.178×10^{-2}	100.1	18.00	1.19	0.17	0.10	0.21	0.22	0.25	0.16
SP	SM_R5C7	4.44×10^{5}	4.380×10^{-2}	98.6	14.85	1.21	0.20	0.20	0.20	0.25	0.26	0.18
SP	SM_R5C8	4.46×10^{5}	4.346×10^{-2}	97.5	11.95	1.21	0.31	0.15	0.22	0.24	0.25	0.17
SP	SM_R5C9	4.56×10^{5}	4.324×10^{-2}	94.8	12.14	1.20	0.25	0.20	0.22	0.27	0.28	0.19
SP	SM_R6C1	3.26×10^{5}	3.748×10^{-2}	114.9	16.39	1.20	0.03	0.02	0.21	0.28	0.26	0.15
SP	SM_R6C2	3.82×10^{5}	4.088×10^{-2}	107.1	21.30	1.21	0.17	0.08	0.23	0.27	0.27	0.17
SP	SM_R6C3	3.93×10^{5}	4.097×10^{-2}	104.3	19.64	1.22	0.08	0.07	0.21	0.28	0.26	0.17
SP	SM_R6C4	3.81×10^{5}	3.876×10^{-2}	101.8	15.14	1.22	0.10	0.07	0.20	0.26	0.25	0.16
SP	SM_R6C5	4.41×10^{5}	3.989×10^{-2}	90.4	12.31	1.19	0.33	0.18	0.25	0.29	0.28	0.20
SP	SM_R6C6	4.00×10^{5}	3.821×10^{-2}	95.5	16.29	1.20	0.27	0.18	0.23	0.25	0.26	0.18
SP	SM_R6C7	3.91×10^{5}	4.103×10^{-2}	104.8	19.99	1.24	0.16	0.15	0.19	0.24	0.25	0.18
SP	SM_R6C8	4.01×10^{5}	4.017×10^{-2}	100.1	17.43	1.21	0.23	0.18	0.21	0.25	0.25	0.18
SP	SM_R6C9	3.78×10^{5}	4.069×10^{-2}	107.6	12.50	1.22	0.21	0.04	0.22	0.22	0.25	0.18
**Variant b (12 sensors)**												
MP-1	SM_R1C8	1.11×10^{5}	4.955×10^{-2}	445.2	123.29	1.91	0.00	0.00	0.11	0.26	0.27	0.24
MP-1	SM_R4C5	1.48×10^{5}	5.307×10^{-2}	359.0	102.85	1.74	0.01	0.04	0.14	0.34	0.31	0.26
MP-1	SM_R7C2	1.74×10^{5}	7.328×10^{-2}	420.4	65.00	1.78	0.01	0.15	0.23	0.44	0.38	0.33
MP-2	SM_R1C8	1.19×10^{5}	3.959×10^{-2}	331.8	47.76	1.66	0.12	0.23	0.20	0.42	0.54	0.40
MP-2	SM_R4C5	1.18×10^{5}	4.758×10^{-2}	404.7	74.41	1.84	0.00	0.00	0.07	0.36	0.42	0.31
MP-2	SM_R7C2	1.56×10^{5}	6.008×10^{-2}	383.9	58.66	1.75	0.00	0.03	0.17	0.53	0.51	0.39
MP-3	SM_R1C8	1.64×10^{5}	4.106×10^{-2}	250.0	40.45	1.53	0.00	0.16	0.24	0.44	0.59	0.39
MP-3	SM_R4C5	1.34×10^{5}	4.320×10^{-2}	323.1	68.26	1.68	0.28	0.08	0.14	0.38	0.45	0.32
MP-3	SM_R7C2	2.03×10^{5}	5.300×10^{-2}	260.7	51.65	1.53	0.32	0.13	0.20	0.55	0.54	0.39
MP-4	SM_R1C8	1.51×10^{5}	3.350×10^{-2}	222.4	41.33	1.36	0.31	0.27	0.35	0.42	0.45	0.49
MP-4	SM_R4C5	1.28×10^{5}	4.137×10^{-2}	322.2	70.95	1.59	0.27	0.19	0.20	0.40	0.39	0.40
MP-4	SM_R7C2	1.70×10^{5}	4.597×10^{-2}	269.6	52.21	1.43	0.04	0.13	0.22	0.58	0.49	0.47

Kinetic parameters for compound 8. SP = singleplex, MP-1/2/3/4 = multiplexed chip SCK-1/2/3/4. $f_{s,i}$ = non-dissociating fraction for cycle i.

Compound 9

Source	Sensor	k_a ($M^{-1}s^{-1}$)	k_d (s^{-1})	K_D (nM)	R_{max} (pg/mm^2)	$R_{max,n}$	$f_{s,1}$	$f_{s,2}$	$f_{s,3}$	$f_{s,4}$	$f_{s,5}$	$f_{s,6}$
**Variant a (54 sensors)**												
SP	SM_R1C1	5.24×10^{5}	3.343×10^{-2}	63.8	6.30	1.07	0.45	0.46	0.47	0.31	0.23	0.08
SP	SM_R1C2	5.18×10^{5}	3.394×10^{-2}	65.5	11.11	1.10	0.55	0.40	0.44	0.36	0.28	0.19
SP	SM_R1C3	4.32×10^{5}	3.891×10^{-2}	90.0	18.53	1.17	0.16	0.20	0.33	0.32	0.24	0.18
SP	SM_R1C4	4.82×10^{5}	3.908×10^{-2}	81.0	14.58	1.12	0.33	0.26	0.40	0.35	0.29	0.22
SP	SM_R1C5	4.46×10^{5}	3.868×10^{-2}	86.8	13.91	1.14	0.39	0.32	0.38	0.31	0.29	0.22
SP	SM_R1C6	4.27×10^{5}	3.650×10^{-2}	85.5	15.44	1.13	0.27	0.26	0.36	0.32	0.28	0.21
SP	SM_R1C7	4.29×10^{5}	3.624×10^{-2}	84.5	14.83	1.13	0.40	0.35	0.40	0.33	0.31	0.21
SP	SM_R1C8	4.22×10^{5}	3.748×10^{-2}	88.8	13.21	1.16	0.21	0.31	0.36	0.33	0.27	0.19
SP	SM_R1C9	4.47×10^{5}	3.792×10^{-2}	84.9	10.56	1.15	0.33	0.30	0.37	0.29	0.26	0.18
SP	SM_R2C1	4.83×10^{5}	3.723×10^{-2}	77.1	14.00	1.12	0.39	0.31	0.38	0.37	0.28	0.22
SP	SM_R2C2	4.64×10^{5}	4.037×10^{-2}	87.1	16.30	1.14	0.28	0.22	0.35	0.30	0.26	0.19
SP	SM_R2C3	4.59×10^{5}	3.835×10^{-2}	83.5	17.83	1.14	0.25	0.26	0.34	0.34	0.26	0.18
SP	SM_R2C4	4.81×10^{5}	3.781×10^{-2}	78.7	15.88	1.12	0.36	0.28	0.36	0.36	0.28	0.21
SP	SM_R2C5	4.52×10^{5}	3.883×10^{-2}	85.9	13.14	1.12	0.38	0.24	0.39	0.33	0.29	0.23
SP	SM_R2C6	4.55×10^{5}	3.811×10^{-2}	83.7	12.17	1.14	0.22	0.26	0.35	0.34	0.30	0.22
SP	SM_R2C7	4.06×10^{5}	3.865×10^{-2}	95.1	14.72	1.17	0.37	0.26	0.37	0.34	0.30	0.23
SP	SM_R2C8	3.92×10^{5}	3.941×10^{-2}	100.6	15.32	1.18	0.11	0.25	0.27	0.32	0.27	0.20
SP	SM_R2C9	3.61×10^{5}	3.971×10^{-2}	110.0	13.85	1.21	0.10	0.17	0.26	0.29	0.25	0.21
SP	SM_R3C1	4.38×10^{5}	3.537×10^{-2}	80.8	14.54	1.15	0.19	0.27	0.36	0.32	0.26	0.17
SP	SM_R3C2	4.41×10^{5}	3.760×10^{-2}	85.3	12.62	1.16	0.16	0.27	0.36	0.30	0.26	0.19
SP	SM_R3C3	4.54×10^{5}	3.777×10^{-2}	83.2	15.79	1.15	0.32	0.25	0.38	0.34	0.26	0.21
SP	SM_R3C4	4.01×10^{5}	3.581×10^{-2}	89.4	17.48	1.17	0.15	0.23	0.32	0.32	0.26	0.18
SP	SM_R3C5	4.14×10^{5}	3.646×10^{-2}	88.1	15.04	1.16	0.28	0.30	0.35	0.34	0.29	0.21
SP	SM_R3C6	4.36×10^{5}	4.247×10^{-2}	97.5	12.31	1.17	0.14	0.16	0.27	0.31	0.25	0.19
SP	SM_R3C7	4.13×10^{5}	3.698×10^{-2}	89.6	12.29	1.18	0.21	0.26	0.33	0.30	0.30	0.21
SP	SM_R3C8	3.73×10^{5}	3.768×10^{-2}	101.0	13.34	1.19	0.05	0.16	0.30	0.29	0.26	0.19
SP	SM_R3C9	3.86×10^{5}	3.873×10^{-2}	100.3	14.23	1.16	0.15	0.17	0.26	0.31	0.23	0.19
SP	SM_R4C1	4.43×10^{5}	3.638×10^{-2}	82.2	18.62	1.16	0.23	0.27	0.39	0.29	0.26	0.17
SP	SM_R4C2	4.51×10^{5}	3.926×10^{-2}	87.0	14.11	1.15	0.21	0.25	0.32	0.30	0.24	0.18
SP	SM_R4C3	4.33×10^{5}	3.563×10^{-2}	82.3	11.32	1.15	0.20	0.23	0.35	0.32	0.26	0.19
SP	SM_R4C4	4.18×10^{5}	3.973×10^{-2}	95.1	12.74	1.12	0.31	0.20	0.35	0.29	0.26	0.21
SP	SM_R4C5	4.78×10^{5}	4.200×10^{-2}	87.8	14.57	1.11	0.29	0.30	0.33	0.34	0.27	0.20
SP	SM_R4C6	4.45×10^{5}	4.074×10^{-2}	91.4	13.27	1.13	0.18	0.27	0.28	0.31	0.27	0.20
SP	SM_R4C7	4.53×10^{5}	3.831×10^{-2}	84.5	10.25	1.15	0.13	0.29	0.26	0.34	0.27	0.19
SP	SM_R4C8	3.74×10^{5}	3.782×10^{-2}	101.1	11.25	1.18	0.25	0.20	0.29	0.32	0.26	0.21
SP	SM_R4C9	3.86×10^{5}	3.596×10^{-2}	93.1	12.25	1.15	0.18	0.21	0.32	0.30	0.28	0.20
SP	SM_R5C1	4.11×10^{5}	3.479×10^{-2}	84.6	19.89	1.16	0.24	0.26	0.33	0.32	0.24	0.17
SP	SM_R5C2	4.03×10^{5}	3.673×10^{-2}	91.2	19.00	1.17	0.19	0.23	0.27	0.31	0.25	0.18
SP	SM_R5C3	4.47×10^{5}	3.735×10^{-2}	83.6	14.94	1.15	0.31	0.28	0.34	0.34	0.28	0.21
SP	SM_R5C4	4.85×10^{5}	3.948×10^{-2}	81.4	10.58	1.13	0.44	0.38	0.40	0.34	0.29	0.20
SP	SM_R5C5	3.64×10^{5}	3.609×10^{-2}	99.2	13.00	1.16	0.03	0.06	0.27	0.26	0.24	0.16
SP	SM_R5C6	3.73×10^{5}	3.781×10^{-2}	101.3	17.10	1.18	0.00	0.12	0.24	0.28	0.24	0.17
SP	SM_R5C7	4.00×10^{5}	3.897×10^{-2}	97.5	14.43	1.17	0.16	0.21	0.31	0.29	0.25	0.20
SP	SM_R5C8	4.67×10^{5}	4.227×10^{-2}	90.5	11.45	1.15	0.37	0.30	0.31	0.32	0.24	0.21
SP	SM_R5C9	3.79×10^{5}	3.882×10^{-2}	102.6	12.20	1.16	0.15	0.20	0.30	0.30	0.25	0.21
SP	SM_R6C1	3.71×10^{5}	3.110×10^{-2}	83.7	14.65	1.15	0.32	0.31	0.37	0.35	0.25	0.18
SP	SM_R6C2	4.12×10^{5}	3.490×10^{-2}	84.6	19.37	1.13	0.25	0.28	0.32	0.36	0.26	0.19
SP	SM_R6C3	3.87×10^{5}	3.470×10^{-2}	89.7	19.12	1.15	0.25	0.21	0.31	0.32	0.26	0.20
SP	SM_R6C4	4.10×10^{5}	3.436×10^{-2}	83.8	14.60	1.15	0.24	0.22	0.34	0.33	0.27	0.18
SP	SM_R6C5	4.03×10^{5}	3.866×10^{-2}	95.9	12.25	1.19	0.27	0.26	0.29	0.30	0.26	0.17
SP	SM_R6C6	4.29×10^{5}	3.943×10^{-2}	91.9	15.96	1.16	0.34	0.31	0.31	0.35	0.28	0.21
SP	SM_R6C7	3.82×10^{5}	3.722×10^{-2}	97.4	19.44	1.17	0.17	0.21	0.31	0.30	0.26	0.20
SP	SM_R6C8	3.90×10^{5}	3.783×10^{-2}	97.0	17.25	1.17	0.26	0.29	0.32	0.33	0.28	0.20
SP	SM_R6C9	4.01×10^{5}	4.082×10^{-2}	101.7	12.76	1.16	0.49	0.34	0.37	0.29	0.29	0.25
**Variant b (12 sensors)**												
MP-1	SM_R3C3	2.36×10^{5}	4.069×10^{-2}	172.7	80.97	1.36	0.07	0.12	0.18	0.37	0.32	0.28
MP-1	SM_R5C8	2.15×10^{5}	4.388×10^{-2}	203.7	55.36	1.43	0.00	0.05	0.15	0.34	0.34	0.30
MP-1	SM_R8C5	2.29×10^{5}	4.762×10^{-2}	207.7	79.70	1.46	0.00	0.00	0.11	0.29	0.30	0.27
MP-2	SM_R3C3	1.92×10^{5}	3.937×10^{-2}	205.1	68.11	1.47	0.00	0.03	0.11	0.38	0.39	0.30
MP-2	SM_R5C8	1.80×10^{5}	4.140×10^{-2}	229.6	33.21	1.51	0.12	0.14	0.15	0.39	0.51	0.38
MP-2	SM_R8C5	2.38×10^{5}	4.458×10^{-2}	187.5	51.43	1.43	0.00	0.03	0.16	0.44	0.49	0.37
MP-3	SM_R3C3	2.07×10^{5}	3.636×10^{-2}	175.9	67.77	1.41	0.13	0.13	0.16	0.41	0.45	0.35
MP-3	SM_R5C8	2.00×10^{5}	3.867×10^{-2}	193.8	30.42	1.44	0.16	0.09	0.14	0.34	0.50	0.36
MP-3	SM_R8C5	2.55×10^{5}	4.323×10^{-2}	169.2	50.13	1.42	0.00	0.08	0.19	0.42	0.53	0.39
MP-4	SM_R3C3	1.95×10^{5}	3.456×10^{-2}	177.5	69.09	1.36	0.14	0.13	0.19	0.42	0.40	0.41
MP-4	SM_R5C8	1.68×10^{5}	3.477×10^{-2}	206.8	32.52	1.34	0.15	0.14	0.16	0.36	0.41	0.46
MP-4	SM_R8C5	2.30×10^{5}	3.618×10^{-2}	157.1	49.25	1.27	0.01	0.09	0.19	0.45	0.47	0.44

Kinetic parameters for compound 9. SP = singleplex, MP-1/2/3/4 = multiplexed chip SCK-1/2/3/4. $f_{s,i}$ = non-dissociating fraction for cycle i.

Compound 10

Source	Sensor	k_a ($M^{-1}s^{-1}$)	k_d (s^{-1})	K_D (nM)	R_{max} (pg/mm^2)	$R_{max,n}$	$f_{s,1}$	$f_{s,2}$	$f_{s,3}$	$f_{s,4}$	$f_{s,5}$	$f_{s,6}$
**Variant b (12 sensors)**												
MP-1	SM_R2C8	1.68×10^5	2.194×10^{-2}	131.0	44.48	1.10	0.00	0.00	0.46	0.10	0.11	0.57
MP-1	SM_R5C5	2.55×10^5	4.396×10^{-2}	172.4	55.33	1.38	0.07	0.12	0.22	0.35	0.38	0.30
MP-1	SM_R8C2	3.15×10^5	4.634×10^{-2}	147.0	56.22	1.34	0.04	0.17	0.33	0.35	0.41	0.29
MP-2	SM_R2C8	2.05×10^5	3.694×10^{-2}	180.0	8.30	1.26	0.66	0.32	0.39	0.44	0.86	0.60
MP-2	SM_R5C5	1.72×10^5	4.271×10^{-2}	248.1	31.30	1.56	0.00	0.08	0.14	0.35	0.49	0.34
MP-2	SM_R8C2	2.83×10^5	4.584×10^{-2}	162.2	45.64	1.38	0.00	0.12	0.31	0.54	0.61	0.38
MP-3	SM_R2C8	1.41×10^5	2.268×10^{-2}	160.4	5.23	0.92	0.00	0.08	0.35	0.17	1.00	0.61
MP-3	SM_R5C5	2.04×10^5	4.318×10^{-2}	211.7	32.80	1.48	0.00	0.05	0.15	0.30	0.48	0.34
MP-3	SM_R8C2	3.06×10^5	4.344×10^{-2}	142.0	46.11	1.26	0.00	0.19	0.35	0.55	0.63	0.38
MP-4	SM_R2C8	8.64×10^4	3.152×10^{-2}	364.7	12.97	1.31	0.59	0.49	0.33	0.30	0.51	0.68
MP-4	SM_R5C5	1.69×10^5	3.658×10^{-2}	217.0	32.69	1.36	0.03	0.11	0.17	0.35	0.40	0.44
MP-4	SM_R8C2	2.71×10^5	3.261×10^{-2}	120.2	41.96	1.20	0.10	0.20	0.36	0.58	0.60	0.41

Kinetic parameters for compound 10. SP = singleplex, MP-1/2/3/4 = multiplexed chip SCK-1/2/3/4. $f_{s,i}$ = non-dissociating fraction for cycle i.

Compound 11

Source	Sensor	k_a ($M^{-1}s^{-1}$)	k_d (s^{-1})	K_D (nM)	R_{max} (pg/mm^2)	$R_{max,n}$	$f_{s,1}$	$f_{s,2}$	$f_{s,3}$	$f_{s,4}$	$f_{s,5}$	$f_{s,6}$
**Variant a (54 sensors)**												
SP	SM_R1C1	7.22×10^{5}	3.230×10^{-2}	44.7	5.77	1.02	0.41	0.42	0.44	0.27	0.25	0.09
SP	SM_R1C2	6.06×10^{5}	3.269×10^{-2}	54.0	9.49	1.09	0.19	0.28	0.38	0.31	0.25	0.09
SP	SM_R1C3	6.54×10^{5}	3.901×10^{-2}	59.6	16.29	1.13	0.31	0.26	0.37	0.34	0.29	0.14
SP	SM_R1C4	6.57×10^{5}	4.067×10^{-2}	61.9	13.04	1.14	0.26	0.27	0.36	0.35	0.31	0.15
SP	SM_R1C5	6.23×10^{5}	3.855×10^{-2}	61.9	11.86	1.13	0.25	0.27	0.36	0.34	0.28	0.16
SP	SM_R1C6	5.45×10^{5}	3.719×10^{-2}	68.3	13.25	1.15	0.19	0.19	0.35	0.32	0.27	0.13
SP	SM_R1C7	5.43×10^{5}	3.777×10^{-2}	69.6	13.29	1.14	0.30	0.28	0.36	0.34	0.29	0.16
SP	SM_R1C8	6.23×10^{5}	3.862×10^{-2}	62.0	12.14	1.14	0.37	0.28	0.40	0.35	0.30	0.16
SP	SM_R1C9	5.39×10^{5}	3.711×10^{-2}	68.9	9.96	1.14	0.30	0.25	0.34	0.32	0.27	0.12
SP	SM_R2C1	6.39×10^{5}	3.709×10^{-2}	58.0	11.46	1.12	0.23	0.30	0.37	0.34	0.27	0.11
SP	SM_R2C2	6.48×10^{5}	3.945×10^{-2}	60.9	13.53	1.15	0.18	0.27	0.35	0.33	0.29	0.12
SP	SM_R2C3	6.53×10^{5}	3.966×10^{-2}	60.7	15.33	1.16	0.22	0.24	0.34	0.35	0.28	0.13
SP	SM_R2C4	6.51×10^{5}	3.922×10^{-2}	60.3	13.77	1.15	0.20	0.27	0.34	0.34	0.29	0.13
SP	SM_R2C5	5.89×10^{5}	3.776×10^{-2}	64.1	11.05	1.14	0.12	0.19	0.29	0.32	0.25	0.13
SP	SM_R2C6	5.69×10^{5}	4.039×10^{-2}	71.0	11.49	1.17	0.21	0.23	0.33	0.34	0.31	0.17
SP	SM_R2C7	5.94×10^{5}	4.027×10^{-2}	67.8	13.62	1.15	0.21	0.21	0.33	0.31	0.30	0.16
SP	SM_R2C8	5.90×10^{5}	4.243×10^{-2}	72.0	14.25	1.16	0.29	0.29	0.33	0.34	0.31	0.20
SP	SM_R2C9	5.57×10^{5}	4.048×10^{-2}	72.7	12.69	1.17	0.21	0.22	0.31	0.31	0.29	0.17
SP	SM_R3C1	6.66×10^{5}	3.911×10^{-2}	58.7	12.05	1.14	0.20	0.26	0.37	0.31	0.26	0.07
SP	SM_R3C2	6.38×10^{5}	3.711×10^{-2}	58.1	10.86	1.15	0.24	0.26	0.34	0.32	0.28	0.12
SP	SM_R3C3	6.31×10^{5}	3.774×10^{-2}	59.8	13.43	1.11	0.23	0.26	0.35	0.32	0.29	0.14
SP	SM_R3C4	5.71×10^{5}	3.754×10^{-2}	65.8	15.25	1.17	0.21	0.24	0.34	0.34	0.28	0.14
SP	SM_R3C5	5.86×10^{5}	3.970×10^{-2}	67.8	13.53	1.17	0.23	0.24	0.33	0.33	0.29	0.16
SP	SM_R3C6	6.52×10^{5}	4.246×10^{-2}	65.2	11.01	1.15	0.19	0.23	0.31	0.31	0.29	0.16
SP	SM_R3C7	5.76×10^{5}	3.903×10^{-2}	67.8	10.86	1.15	0.40	0.27	0.37	0.35	0.31	0.18
SP	SM_R3C8	6.31×10^{5}	4.206×10^{-2}	66.7	11.83	1.14	0.34	0.28	0.34	0.33	0.30	0.18
SP	SM_R3C9	5.40×10^{5}	4.159×10^{-2}	77.0	12.89	1.16	0.23	0.23	0.32	0.35	0.28	0.18
SP	SM_R4C1	6.74×10^{5}	3.579×10^{-2}	53.1	15.31	1.11	0.30	0.32	0.39	0.32	0.27	0.08
SP	SM_R4C2	6.29×10^{5}	3.896×10^{-2}	61.9	11.82	1.16	0.18	0.24	0.34	0.29	0.27	0.09
SP	SM_R4C3	6.45×10^{5}	3.742×10^{-2}	58.0	9.83	1.14	0.25	0.23	0.35	0.32	0.26	0.12
SP	SM_R4C4	6.29×10^{5}	3.942×10^{-2}	62.6	10.74	1.15	0.22	0.27	0.35	0.29	0.28	0.12
SP	SM_R4C5	5.80×10^{5}	3.909×10^{-2}	67.4	12.99	1.16	0.17	0.21	0.31	0.29	0.26	0.12
SP	SM_R4C6	5.64×10^{5}	4.097×10^{-2}	72.7	12.26	1.17	0.11	0.19	0.27	0.30	0.27	0.15
SP	SM_R4C7	5.47×10^{5}	4.110×10^{-2}	75.1	10.38	1.16	0.10	0.27	0.26	0.34	0.28	0.18
SP	SM_R4C8	5.57×10^{5}	3.934×10^{-2}	70.7	9.96	1.15	0.17	0.21	0.29	0.32	0.27	0.15
SP	SM_R4C9	4.98×10^{5}	3.665×10^{-2}	73.6	11.39	1.15	0.25	0.25	0.32	0.26	0.29	0.17
SP	SM_R5C1	5.67×10^{5}	3.470×10^{-2}	61.1	16.42	1.15	0.25	0.22	0.36	0.32	0.26	0.09
SP	SM_R5C2	5.87×10^{5}	3.656×10^{-2}	62.2	15.96	1.14	0.28	0.28	0.37	0.34	0.28	0.14
SP	SM_R5C3	5.51×10^{5}	3.823×10^{-2}	69.4	12.65	1.17	0.12	0.20	0.30	0.31	0.28	0.14
SP	SM_R5C4	5.82×10^{5}	3.552×10^{-2}	61.1	9.24	1.13	0.33	0.29	0.35	0.32	0.28	0.15
SP	SM_R5C5	5.60×10^{5}	3.824×10^{-2}	68.3	11.72	1.14	0.26	0.24	0.34	0.32	0.28	0.16
SP	SM_R5C6	5.48×10^{5}	3.913×10^{-2}	71.3	15.04	1.16	0.19	0.20	0.30	0.29	0.28	0.14
SP	SM_R5C7	6.07×10^{5}	4.231×10^{-2}	69.7	12.94	1.16	0.24	0.26	0.29	0.29	0.29	0.16
SP	SM_R5C8	5.60×10^{5}	4.146×10^{-2}	74.0	9.89	1.16	0.23	0.17	0.30	0.26	0.28	0.13
SP	SM_R5C9	5.30×10^{5}	3.906×10^{-2}	73.7	10.69	1.18	0.21	0.21	0.31	0.29	0.29	0.15
SP	SM_R6C1	5.55×10^{5}	3.492×10^{-2}	62.9	12.96	1.11	0.33	0.33	0.39	0.35	0.26	0.16
SP	SM_R6C2	5.30×10^{5}	3.601×10^{-2}	67.9	17.46	1.14	0.20	0.26	0.34	0.36	0.27	0.15
SP	SM_R6C3	5.29×10^{5}	3.505×10^{-2}	66.3	17.47	1.15	0.22	0.24	0.33	0.35	0.27	0.15
SP	SM_R6C4	5.50×10^{5}	3.606×10^{-2}	65.5	13.72	1.16	0.20	0.23	0.33	0.33	0.27	0.15
SP	SM_R6C5	4.76×10^{5}	3.668×10^{-2}	77.0	11.26	1.15	0.18	0.19	0.31	0.32	0.27	0.14
SP	SM_R6C6	5.38×10^{5}	3.974×10^{-2}	73.9	15.01	1.15	0.25	0.23	0.31	0.35	0.28	0.18
SP	SM_R6C7	5.14×10^{5}	3.738×10^{-2}	72.7	18.02	1.13	0.23	0.22	0.30	0.31	0.27	0.17
SP	SM_R6C8	5.11×10^{5}	3.715×10^{-2}	72.7	16.44	1.16	0.19	0.23	0.27	0.30	0.27	0.17
SP	SM_R6C9	4.52×10^{5}	3.756×10^{-2}	83.0	11.64	1.16	0.19	0.24	0.29	0.28	0.26	0.18
**Variant b (12 sensors)**												
MP-1	SM_R3C4	2.33×10^{5}	3.261×10^{-2}	139.9	111.16	1.31	0.00	0.01	0.11	0.32	0.22	0.20
MP-1	SM_R6C1	3.41×10^{5}	4.631×10^{-2}	135.7	53.07	1.24	0.00	0.09	0.24	0.44	0.30	0.29
MP-1	SM_R8C6	2.35×10^{5}	3.595×10^{-2}	152.8	91.24	1.34	0.00	0.00	0.06	0.24	0.20	0.17
MP-2	SM_R3C4	1.79×10^{5}	3.043×10^{-2}	170.3	93.52	1.40	0.02	0.00	0.03	0.37	0.31	0.21
MP-2	SM_R6C1	2.89×10^{5}	4.701×10^{-2}	162.8	50.08	1.35	0.00	0.00	0.13	0.62	0.40	0.33
MP-2	SM_R8C6	2.18×10^{5}	3.490×10^{-2}	160.2	65.03	1.39	0.00	0.02	0.06	0.39	0.37	0.25
MP-3	SM_R3C4	1.88×10^{5}	2.929×10^{-2}	155.9	98.81	1.37	0.20	0.05	0.09	0.38	0.33	0.22
MP-3	SM_R6C1	3.17×10^{5}	4.108×10^{-2}	129.6	52.11	1.30	0.12	0.05	0.17	0.62	0.44	0.31
MP-3	SM_R8C6	2.25×10^{5}	3.410×10^{-2}	151.4	71.30	1.38	0.12	0.03	0.09	0.37	0.37	0.27
MP-4	SM_R3C4	1.87×10^{5}	2.857×10^{-2}	152.9	97.81	1.32	0.07	0.07	0.09	0.39	0.30	0.28
MP-4	SM_R6C1	2.84×10^{5}	3.645×10^{-2}	128.6	51.09	1.21	0.03	0.09	0.18	0.65	0.39	0.38
MP-4	SM_R8C6	2.21×10^{5}	3.069×10^{-2}	139.0	67.72	1.27	0.04	0.06	0.12	0.39	0.34	0.33

Kinetic parameters for compound 11. SP = singleplex, MP-1/2/3/4 = multiplexed chip SCK-1/2/3/4. $f_{s,i}$ = non-dissociating fraction for cycle i.

Compound 12

Source	Sensor	k_a ($M^{-1}s^{-1}$)	k_d (s^{-1})	K_D (nM)	R_{max} (pg/mm^2)	$R_{max,n}$	$f_{s,1}$	$f_{s,2}$	$f_{s,3}$	$f_{s,4}$	$f_{s,5}$	$f_{s,6}$
**Variant a (54 sensors)**												
SP	SM_R1C1	9.11×10^{5}	3.174×10^{-2}	34.9	5.69	1.04	0.34	0.26	0.36	0.37	0.26	0.10
SP	SM_R1C2	7.28×10^{5}	2.876×10^{-2}	39.5	9.14	1.07	0.14	0.22	0.28	0.32	0.27	0.09
SP	SM_R1C3	6.88×10^{5}	3.171×10^{-2}	46.1	15.64	1.08	0.16	0.17	0.25	0.30	0.28	0.12
SP	SM_R1C4	8.01×10^{5}	3.323×10^{-2}	41.5	12.55	1.06	0.30	0.26	0.28	0.33	0.32	0.17
SP	SM_R1C5	7.27×10^{5}	3.375×10^{-2}	46.4	12.03	1.10	0.25	0.21	0.25	0.29	0.28	0.14
SP	SM_R1C6	7.09×10^{5}	3.301×10^{-2}	46.6	14.00	1.11	0.25	0.23	0.28	0.33	0.29	0.17
SP	SM_R1C7	6.82×10^{5}	3.045×10^{-2}	44.7	13.54	1.08	0.23	0.22	0.28	0.31	0.28	0.13
SP	SM_R1C8	7.04×10^{5}	3.192×10^{-2}	45.3	12.18	1.09	0.28	0.28	0.29	0.34	0.30	0.16
SP	SM_R1C9	7.01×10^{5}	3.286×10^{-2}	46.8	10.22	1.10	0.18	0.22	0.26	0.31	0.27	0.14
SP	SM_R2C1	7.11×10^{5}	2.991×10^{-2}	42.1	12.09	1.06	0.20	0.24	0.29	0.36	0.29	0.15
SP	SM_R2C2	7.17×10^{5}	3.136×10^{-2}	43.7	13.92	1.09	0.16	0.18	0.26	0.32	0.28	0.13
SP	SM_R2C3	7.23×10^{5}	2.950×10^{-2}	40.8	15.75	1.08	0.24	0.23	0.27	0.33	0.28	0.14
SP	SM_R2C4	7.20×10^{5}	3.137×10^{-2}	43.6	14.14	1.09	0.22	0.22	0.26	0.32	0.28	0.15
SP	SM_R2C5	6.62×10^{5}	3.142×10^{-2}	47.5	11.53	1.12	0.22	0.20	0.24	0.32	0.28	0.15
SP	SM_R2C6	6.33×10^{5}	3.125×10^{-2}	49.4	11.88	1.10	0.08	0.10	0.19	0.27	0.27	0.13
SP	SM_R2C7	6.33×10^{5}	3.107×10^{-2}	49.1	13.87	1.08	0.19	0.18	0.23	0.30	0.29	0.17
SP	SM_R2C8	6.17×10^{5}	3.157×10^{-2}	51.2	14.35	1.11	0.29	0.24	0.24	0.30	0.30	0.19
SP	SM_R2C9	6.29×10^{5}	3.320×10^{-2}	52.8	12.69	1.14	0.14	0.15	0.21	0.28	0.28	0.16
SP	SM_R3C1	7.28×10^{5}	3.022×10^{-2}	41.5	12.28	1.06	0.14	0.20	0.26	0.33	0.27	0.11
SP	SM_R3C2	7.07×10^{5}	3.063×10^{-2}	43.4	11.04	1.10	0.19	0.18	0.26	0.31	0.27	0.14
SP	SM_R3C3	6.18×10^{5}	3.034×10^{-2}	49.1	13.83	1.10	0.14	0.17	0.21	0.27	0.26	0.12
SP	SM_R3C4	6.87×10^{5}	3.028×10^{-2}	44.1	16.10	1.10	0.21	0.21	0.25	0.30	0.28	0.14
SP	SM_R3C5	6.30×10^{5}	3.066×10^{-2}	48.7	13.82	1.09	0.04	0.10	0.19	0.25	0.25	0.12
SP	SM_R3C6	6.85×10^{5}	3.122×10^{-2}	45.6	11.72	1.09	0.23	0.20	0.24	0.30	0.31	0.17
SP	SM_R3C7	7.04×10^{5}	3.012×10^{-2}	42.8	11.38	1.02	0.07	0.11	0.19	0.25	0.26	0.13
SP	SM_R3C8	7.07×10^{5}	3.103×10^{-2}	43.9	11.85	1.08	0.30	0.25	0.27	0.32	0.30	0.19
SP	SM_R3C9	6.58×10^{5}	3.020×10^{-2}	45.9	12.55	1.09	0.19	0.17	0.22	0.28	0.27	0.14
SP	SM_R4C1	7.40×10^{5}	3.034×10^{-2}	41.0	15.70	1.10	0.16	0.20	0.27	0.32	0.25	0.11
SP	SM_R4C2	7.31×10^{5}	3.056×10^{-2}	41.8	12.36	1.08	0.21	0.23	0.27	0.31	0.28	0.13
SP	SM_R4C3	7.20×10^{5}	3.058×10^{-2}	42.4	10.37	1.09	0.19	0.20	0.25	0.31	0.27	0.14
SP	SM_R4C4	6.71×10^{5}	3.076×10^{-2}	45.8	11.41	1.11	0.19	0.19	0.25	0.30	0.26	0.13
SP	SM_R4C5	6.47×10^{5}	2.962×10^{-2}	45.8	13.90	1.10	0.20	0.21	0.24	0.30	0.27	0.15
SP	SM_R4C6	6.17×10^{5}	2.943×10^{-2}	47.7	12.35	1.08	0.16	0.19	0.22	0.27	0.27	0.16
SP	SM_R4C7	6.58×10^{5}	3.044×10^{-2}	46.3	9.83	1.08	0.22	0.20	0.22	0.29	0.30	0.17
SP	SM_R4C8	5.96×10^{5}	3.024×10^{-2}	50.7	9.55	1.12	0.05	0.12	0.19	0.26	0.25	0.13
SP	SM_R4C9	6.03×10^{5}	3.005×10^{-2}	49.8	11.12	1.12	0.15	0.15	0.22	0.26	0.26	0.15
SP	SM_R5C1	6.86×10^{5}	3.077×10^{-2}	44.9	17.07	1.12	0.19	0.20	0.27	0.31	0.26	0.12
SP	SM_R5C2	6.79×10^{5}	3.036×10^{-2}	44.7	17.32	1.11	0.25	0.24	0.26	0.31	0.29	0.15
SP	SM_R5C3	6.76×10^{5}	3.123×10^{-2}	46.2	13.22	1.10	0.15	0.17	0.23	0.28	0.28	0.14
SP	SM_R5C4	6.30×10^{5}	3.075×10^{-2}	48.8	9.58	1.10	0.09	0.13	0.22	0.27	0.25	0.12
SP	SM_R5C5	6.38×10^{5}	3.257×10^{-2}	51.1	12.24	1.14	0.15	0.12	0.22	0.28	0.27	0.14
SP	SM_R5C6	6.62×10^{5}	3.032×10^{-2}	45.8	15.36	1.11	0.30	0.25	0.25	0.31	0.31	0.19
SP	SM_R5C7	6.24×10^{5}	3.094×10^{-2}	49.6	12.45	1.10	0.10	0.13	0.20	0.25	0.25	0.15
SP	SM_R5C8	6.38×10^{5}	3.194×10^{-2}	50.1	9.96	1.13	0.19	0.20	0.22	0.28	0.27	0.17
SP	SM_R5C9	6.35×10^{5}	3.060×10^{-2}	48.2	10.83	1.13	0.20	0.19	0.22	0.28	0.28	0.17
SP	SM_R6C1	6.60×10^{5}	2.938×10^{-2}	44.5	13.03	1.06	0.25	0.23	0.27	0.31	0.26	0.12
SP	SM_R6C2	6.48×10^{5}	3.024×10^{-2}	46.7	17.68	1.11	0.21	0.22	0.25	0.30	0.27	0.14
SP	SM_R6C3	6.40×10^{5}	2.917×10^{-2}	45.5	17.10	1.11	0.13	0.18	0.25	0.30	0.27	0.13
SP	SM_R6C4	6.07×10^{5}	2.936×10^{-2}	48.3	13.26	1.11	0.12	0.14	0.22	0.28	0.26	0.12
SP	SM_R6C5	6.16×10^{5}	2.731×10^{-2}	44.4	10.49	1.05	0.17	0.22	0.23	0.29	0.27	0.14
SP	SM_R6C6	6.19×10^{5}	2.992×10^{-2}	48.3	13.96	1.11	0.19	0.18	0.23	0.27	0.26	0.15
SP	SM_R6C7	6.11×10^{5}	3.110×10^{-2}	50.9	17.10	1.13	0.20	0.20	0.22	0.28	0.27	0.17
SP	SM_R6C8	5.93×10^{5}	2.878×10^{-2}	48.5	15.53	1.12	0.27	0.23	0.23	0.27	0.27	0.17
SP	SM_R6C9	5.68×10^{5}	3.064×10^{-2}	53.9	11.06	1.13	0.18	0.19	0.19	0.26	0.27	0.16
**Variant b (12 sensors)**												
MP-1	SM_R2C1	2.65×10^{5}	4.215×10^{-2}	159.3	101.65	1.32	0.00	0.12	0.23	0.34	0.32	0.27
MP-1	SM_R4C6	1.85×10^{5}	4.127×10^{-2}	222.9	82.52	1.47	0.00	0.00	0.11	0.26	0.25	0.21
MP-1	SM_R7C3	2.46×10^{5}	4.842×10^{-2}	196.9	86.92	1.41	0.00	0.04	0.17	0.32	0.28	0.26
MP-2	SM_R2C1	2.16×10^{5}	4.006×10^{-2}	185.2	105.16	1.42	0.05	0.10	0.16	0.41	0.38	0.29
MP-2	SM_R4C6	1.44×10^{5}	3.881×10^{-2}	268.6	59.86	1.58	0.00	0.00	0.07	0.33	0.36	0.26
MP-2	SM_R7C3	2.07×10^{5}	4.921×10^{-2}	237.6	72.15	1.52	0.00	0.00	0.08	0.39	0.37	0.28
MP-3	SM_R2C1	2.21×10^{5}	3.738×10^{-2}	168.9	111.29	1.40	0.29	0.11	0.19	0.44	0.42	0.32
MP-3	SM_R4C6	1.68×10^{5}	3.598×10^{-2}	214.1	55.95	1.48	0.06	0.06	0.12	0.35	0.39	0.28
MP-3	SM_R7C3	2.19×10^{5}	4.488×10^{-2}	205.3	74.51	1.46	0.18	0.04	0.14	0.42	0.41	0.33
MP-4	SM_R2C1	2.30×10^{5}	3.538×10^{-2}	154.1	104.34	1.32	0.08	0.13	0.20	0.46	0.40	0.37
MP-4	SM_R4C6	1.50×10^{5}	3.434×10^{-2}	228.9	60.08	1.43	0.12	0.09	0.13	0.34	0.33	0.34
MP-4	SM_R7C3	2.13×10^{5}	4.030×10^{-2}	189.6	69.56	1.35	0.00	0.07	0.12	0.44	0.38	0.38

Kinetic parameters for compound 12. SP = singleplex, MP-1/2/3/4 = multiplexed chip SCK-1/2/3/4. $f_{s,i}$ = non-dissociating fraction for cycle i.

Compound 13

Source	Sensor	k_a ($M^{-1}s^{-1}$)	k_d (s^{-1})	K_D (nM)	R_{max} (pg/mm^2)	$R_{max,n}$	$f_{s,1}$	$f_{s,2}$	$f_{s,3}$	$f_{s,4}$	$f_{s,5}$	$f_{s,6}$
**Variant a (54 sensors)**												
SP	SM_R1C1	3.65×10^{5}	2.409×10^{-2}	66.1	4.03	1.07	0.48	0.19	0.15	0.18	0.14	0.16
SP	SM_R1C2	4.27×10^{5}	2.570×10^{-2}	60.3	6.67	1.07	0.45	0.19	0.22	0.27	0.22	0.13
SP	SM_R1C3	4.56×10^{5}	2.826×10^{-2}	62.0	10.24	1.10	0.47	0.33	0.30	0.34	0.27	0.16
SP	SM_R1C4	4.49×10^{5}	2.810×10^{-2}	62.6	8.55	1.09	0.37	0.22	0.26	0.26	0.24	0.15
SP	SM_R1C5	4.92×10^{5}	2.895×10^{-2}	58.9	7.31	1.08	0.33	0.37	0.33	0.26	0.20	0.16
SP	SM_R1C6	5.22×10^{5}	2.811×10^{-2}	53.9	8.90	1.10	0.64	0.32	0.30	0.32	0.27	0.18
SP	SM_R1C7	4.46×10^{5}	2.865×10^{-2}	64.2	9.12	1.11	0.46	0.26	0.23	0.31	0.23	0.16
SP	SM_R1C8	4.61×10^{5}	2.846×10^{-2}	61.7	7.98	1.10	0.47	0.28	0.26	0.32	0.27	0.16
SP	SM_R1C9	5.33×10^{5}	3.118×10^{-2}	58.5	6.92	1.10	0.54	0.36	0.36	0.31	0.30	0.19
SP	SM_R2C1	4.29×10^{5}	2.564×10^{-2}	59.7	8.92	1.12	0.50	0.25	0.29	0.34	0.26	0.14
SP	SM_R2C2	4.62×10^{5}	2.728×10^{-2}	59.0	10.13	1.11	0.33	0.23	0.25	0.28	0.22	0.14
SP	SM_R2C3	4.42×10^{5}	2.566×10^{-2}	58.0	11.05	1.10	0.53	0.27	0.25	0.29	0.24	0.13
SP	SM_R2C4	4.47×10^{5}	2.467×10^{-2}	55.2	10.00	1.08	0.42	0.23	0.27	0.27	0.25	0.13
SP	SM_R2C5	4.97×10^{5}	2.667×10^{-2}	53.7	8.33	1.06	0.53	0.37	0.32	0.33	0.26	0.21
SP	SM_R2C6	4.35×10^{5}	3.060×10^{-2}	70.3	8.37	1.15	0.20	0.10	0.17	0.34	0.27	0.14
SP	SM_R2C7	4.20×10^{5}	2.821×10^{-2}	67.1	9.70	1.11	0.49	0.24	0.28	0.31	0.29	0.16
SP	SM_R2C8	3.81×10^{5}	3.074×10^{-2}	80.7	10.31	1.13	0.50	0.38	0.27	0.30	0.26	0.22
SP	SM_R2C9	3.71×10^{5}	3.223×10^{-2}	86.9	9.11	1.16	0.25	0.26	0.23	0.25	0.25	0.17
SP	SM_R3C1	4.21×10^{5}	2.653×10^{-2}	63.0	9.32	1.13	0.21	0.18	0.26	0.27	0.22	0.11
SP	SM_R3C2	4.54×10^{5}	2.809×10^{-2}	61.9	8.03	1.07	0.47	0.32	0.32	0.30	0.26	0.18
SP	SM_R3C3	4.10×10^{5}	2.688×10^{-2}	65.6	9.98	1.12	0.33	0.25	0.26	0.25	0.23	0.15
SP	SM_R3C4	4.57×10^{5}	2.774×10^{-2}	60.7	11.14	1.09	0.36	0.23	0.29	0.30	0.26	0.13
SP	SM_R3C5	4.39×10^{5}	2.788×10^{-2}	63.5	9.70	1.09	0.14	0.22	0.28	0.22	0.18	0.15
SP	SM_R3C6	4.73×10^{5}	2.696×10^{-2}	57.0	7.91	1.10	0.48	0.28	0.27	0.28	0.25	0.18
SP	SM_R3C7	4.50×10^{5}	2.982×10^{-2}	66.3	7.74	1.11	0.17	0.14	0.24	0.23	0.20	0.11
SP	SM_R3C8	4.86×10^{5}	2.867×10^{-2}	59.0	8.27	1.09	0.49	0.39	0.34	0.30	0.24	0.18
SP	SM_R3C9	4.66×10^{5}	2.866×10^{-2}	61.5	8.68	1.09	0.41	0.25	0.30	0.32	0.22	0.16
SP	SM_R4C1	4.53×10^{5}	2.611×10^{-2}	57.6	11.58	1.11	0.31	0.23	0.28	0.27	0.21	0.12
SP	SM_R4C2	4.16×10^{5}	2.607×10^{-2}	62.7	9.05	1.11	0.25	0.16	0.23	0.28	0.21	0.12
SP	SM_R4C3	4.23×10^{5}	2.449×10^{-2}	57.9	7.36	1.09	0.24	0.20	0.24	0.24	0.21	0.12
SP	SM_R4C4	4.06×10^{5}	2.709×10^{-2}	66.7	8.32	1.13	0.38	0.23	0.20	0.24	0.20	0.16
SP	SM_R4C5	4.03×10^{5}	2.684×10^{-2}	66.6	9.93	1.10	0.52	0.25	0.22	0.22	0.23	0.15
SP	SM_R4C6	3.76×10^{5}	2.522×10^{-2}	67.2	8.48	1.12	0.22	0.11	0.16	0.24	0.20	0.12
SP	SM_R4C7	4.02×10^{5}	2.919×10^{-2}	72.5	6.64	1.12	0.35	0.24	0.22	0.25	0.23	0.13
SP	SM_R4C8	4.31×10^{5}	2.505×10^{-2}	58.2	6.58	1.09	0.34	0.22	0.26	0.33	0.23	0.12
SP	SM_R4C9	4.44×10^{5}	2.666×10^{-2}	60.1	7.49	1.11	0.23	0.18	0.27	0.26	0.22	0.12
SP	SM_R5C1	4.17×10^{5}	2.691×10^{-2}	64.5	12.53	1.11	0.56	0.35	0.31	0.26	0.21	0.15
SP	SM_R5C2	3.92×10^{5}	2.692×10^{-2}	68.7	12.24	1.11	0.46	0.28	0.28	0.26	0.22	0.15
SP	SM_R5C3	4.12×10^{5}	2.669×10^{-2}	64.8	9.28	1.07	0.39	0.25	0.32	0.27	0.24	0.15
SP	SM_R5C4	4.46×10^{5}	2.568×10^{-2}	57.6	6.79	1.08	0.45	0.31	0.32	0.29	0.22	0.12
SP	SM_R5C5	4.39×10^{5}	2.729×10^{-2}	62.2	8.47	1.09	0.19	0.24	0.28	0.26	0.19	0.16
SP	SM_R5C6	4.36×10^{5}	2.414×10^{-2}	55.4	10.25	1.09	0.50	0.24	0.29	0.29	0.24	0.13
SP	SM_R5C7	4.29×10^{5}	2.674×10^{-2}	62.3	8.45	1.09	0.35	0.27	0.27	0.21	0.18	0.12
SP	SM_R5C8	4.17×10^{5}	2.410×10^{-2}	57.7	6.51	1.06	0.29	0.22	0.25	0.25	0.20	0.13
SP	SM_R5C9	4.19×10^{5}	2.622×10^{-2}	62.6	7.42	1.09	0.50	0.31	0.29	0.22	0.21	0.14
SP	SM_R6C1	3.84×10^{5}	2.654×10^{-2}	69.1	9.31	1.11	0.35	0.25	0.28	0.27	0.22	0.14
SP	SM_R6C2	3.54×10^{5}	2.547×10^{-2}	72.0	13.05	1.14	0.34	0.17	0.22	0.26	0.23	0.12
SP	SM_R6C3	3.67×10^{5}	2.584×10^{-2}	70.4	12.31	1.11	0.38	0.20	0.25	0.27	0.23	0.15
SP	SM_R6C4	4.16×10^{5}	2.590×10^{-2}	62.2	9.16	1.12	0.14	0.20	0.27	0.29	0.22	0.14
SP	SM_R6C5	3.76×10^{5}	2.451×10^{-2}	65.2	7.22	1.05	0.41	0.27	0.24	0.26	0.22	0.14
SP	SM_R6C6	3.69×10^{5}	2.657×10^{-2}	71.9	9.60	1.15	0.29	0.21	0.25	0.28	0.21	0.12
SP	SM_R6C7	3.54×10^{5}	2.673×10^{-2}	75.5	11.60	1.15	0.21	0.24	0.23	0.19	0.19	0.12
SP	SM_R6C8	3.85×10^{5}	2.559×10^{-2}	66.5	10.42	1.13	0.40	0.22	0.25	0.26	0.21	0.15
SP	SM_R6C9	3.86×10^{5}	2.326×10^{-2}	60.3	7.49	1.09	0.28	0.29	0.30	0.26	0.23	0.18
**Variant b (12 sensors)**												
MP-1	SM_R2C3	2.12×10^{5}	3.802×10^{-2}	179.1	166.89	1.37	0.00	0.04	0.12	0.29	0.24	0.21
MP-1	SM_R4C8	1.78×10^{5}	4.146×10^{-2}	233.0	51.47	1.46	0.00	0.02	0.09	0.27	0.25	0.21
MP-1	SM_R7C5	2.15×10^{5}	4.278×10^{-2}	199.0	135.91	1.41	0.00	0.02	0.10	0.28	0.21	0.20
MP-2	SM_R2C3	1.73×10^{5}	3.560×10^{-2}	205.7	115.33	1.46	0.00	0.00	0.06	0.34	0.30	0.21
MP-2	SM_R4C8	1.01×10^{5}	5.667×10^{-2}	559.5	12.26	2.13	0.00	0.00	0.00	0.28	0.79	0.49
MP-2	SM_R7C5	1.73×10^{5}	3.973×10^{-2}	229.0	105.27	1.51	0.00	0.00	0.05	0.33	0.30	0.22
MP-3	SM_R2C3	1.79×10^{5}	3.387×10^{-2}	189.4	116.03	1.43	0.35	0.06	0.11	0.36	0.33	0.25
MP-3	SM_R4C8	5.06×10^{4}	1.243×10^{-2}	245.6	22.31	1.09	0.52	0.00	0.01	0.20	0.45	0.01
MP-3	SM_R7C5	1.81×10^{5}	3.842×10^{-2}	212.2	112.03	1.48	0.32	0.01	0.09	0.34	0.33	0.25
MP-4	SM_R2C3	1.79×10^{5}	3.296×10^{-2}	184.6	116.30	1.37	0.12	0.10	0.12	0.38	0.31	0.31
MP-4	SM_R4C8	1.29×10^{5}	3.370×10^{-2}	260.5	29.34	1.39	0.00	0.04	0.10	0.27	0.30	0.37
MP-4	SM_R7C5	1.86×10^{5}	3.531×10^{-2}	189.5	101.57	1.38	0.00	0.05	0.10	0.35	0.30	0.30

Kinetic parameters for compound 13. SP = singleplex, MP-1/2/3/4 = multiplexed chip SCK-1/2/3/4. $f_{s,i}$ = non-dissociating fraction for cycle i.

Compound 14

Source	Sensor	k_a ($M^{-1}s^{-1}$)	k_d (s^{-1})	K_D (nM)	R_{max} (pg/mm^2)	$R_{max,n}$	$f_{s,1}$	$f_{s,2}$	$f_{s,3}$	$f_{s,4}$	$f_{s,5}$	$f_{s,6}$
**Variant a (54 sensors)**												
SP	SM_R1C1	5.03×10^{5}	4.306×10^{-2}	85.6	8.92	1.17	0.18	0.15	0.27	0.34	0.25	0.23
SP	SM_R1C2	5.37×10^{5}	4.258×10^{-2}	79.3	13.69	1.15	0.14	0.20	0.26	0.34	0.24	0.22
SP	SM_R1C3	5.04×10^{5}	4.427×10^{-2}	87.8	22.80	1.18	0.22	0.20	0.21	0.30	0.29	0.20
SP	SM_R1C4	4.88×10^{5}	4.284×10^{-2}	87.7	18.64	1.19	0.25	0.19	0.21	0.29	0.25	0.22
SP	SM_R1C5	4.73×10^{5}	4.415×10^{-2}	93.4	17.18	1.19	0.20	0.21	0.21	0.30	0.24	0.21
SP	SM_R1C6	4.72×10^{5}	4.220×10^{-2}	89.4	20.25	1.20	0.20	0.19	0.20	0.28	0.27	0.21
SP	SM_R1C7	4.48×10^{5}	4.091×10^{-2}	91.4	20.15	1.19	0.21	0.17	0.24	0.31	0.24	0.22
SP	SM_R1C8	4.46×10^{5}	4.138×10^{-2}	92.8	16.73	1.21	0.11	0.10	0.20	0.28	0.23	0.19
SP	SM_R1C9	5.21×10^{5}	4.653×10^{-2}	89.3	13.41	1.18	0.11	0.16	0.24	0.31	0.26	0.21
SP	SM_R2C1	5.24×10^{5}	4.124×10^{-2}	78.8	18.27	1.18	0.24	0.23	0.23	0.31	0.29	0.18
SP	SM_R2C2	4.89×10^{5}	4.195×10^{-2}	85.9	21.68	1.19	0.09	0.15	0.22	0.31	0.25	0.19
SP	SM_R2C3	5.12×10^{5}	4.470×10^{-2}	87.3	24.56	1.19	0.17	0.17	0.21	0.29	0.27	0.19
SP	SM_R2C4	5.11×10^{5}	4.601×10^{-2}	90.1	21.99	1.20	0.19	0.20	0.20	0.28	0.29	0.20
SP	SM_R2C5	4.84×10^{5}	4.329×10^{-2}	89.5	17.75	1.21	0.08	0.15	0.20	0.28	0.27	0.20
SP	SM_R2C6	4.89×10^{5}	4.607×10^{-2}	94.3	17.44	1.21	0.16	0.14	0.24	0.30	0.26	0.23
SP	SM_R2C7	4.43×10^{5}	4.550×10^{-2}	102.7	18.82	1.25	0.18	0.15	0.19	0.27	0.28	0.21
SP	SM_R2C8	4.32×10^{5}	4.424×10^{-2}	102.3	18.89	1.24	0.00	0.09	0.15	0.24	0.25	0.19
SP	SM_R2C9	4.76×10^{5}	4.637×10^{-2}	97.5	16.33	1.22	0.15	0.20	0.23	0.29	0.24	0.23
SP	SM_R3C1	5.32×10^{5}	4.555×10^{-2}	85.6	19.33	1.21	0.08	0.18	0.25	0.33	0.27	0.19
SP	SM_R3C2	4.71×10^{5}	4.110×10^{-2}	87.2	16.86	1.21	0.09	0.15	0.23	0.29	0.27	0.19
SP	SM_R3C3	4.55×10^{5}	3.970×10^{-2}	87.2	19.69	1.20	0.12	0.13	0.18	0.27	0.25	0.17
SP	SM_R3C4	4.93×10^{5}	4.328×10^{-2}	87.8	22.15	1.21	0.09	0.15	0.21	0.29	0.27	0.19
SP	SM_R3C5	4.49×10^{5}	4.245×10^{-2}	94.5	19.40	1.22	0.18	0.17	0.22	0.28	0.28	0.22
SP	SM_R3C6	4.56×10^{5}	4.267×10^{-2}	93.6	15.58	1.23	0.00	0.03	0.13	0.24	0.23	0.17
SP	SM_R3C7	4.52×10^{5}	4.314×10^{-2}	95.5	16.00	1.21	0.27	0.18	0.21	0.27	0.28	0.23
SP	SM_R3C8	4.29×10^{5}	4.052×10^{-2}	94.5	16.36	1.22	0.12	0.11	0.15	0.25	0.24	0.19
SP	SM_R3C9	4.66×10^{5}	4.192×10^{-2}	90.0	17.79	1.20	0.26	0.24	0.23	0.28	0.27	0.22
SP	SM_R4C1	4.83×10^{5}	3.897×10^{-2}	80.6	24.75	1.21	0.16	0.19	0.22	0.31	0.27	0.17
SP	SM_R4C2	4.64×10^{5}	4.076×10^{-2}	87.9	18.86	1.21	0.06	0.15	0.19	0.28	0.25	0.17
SP	SM_R4C3	4.42×10^{5}	3.894×10^{-2}	88.0	15.73	1.20	0.00	0.08	0.20	0.27	0.23	0.18
SP	SM_R4C4	4.46×10^{5}	4.034×10^{-2}	90.4	16.39	1.22	0.04	0.13	0.18	0.27	0.26	0.17
SP	SM_R4C5	4.56×10^{5}	4.148×10^{-2}	90.9	19.98	1.21	0.13	0.17	0.20	0.27	0.28	0.19
SP	SM_R4C6	4.64×10^{5}	4.200×10^{-2}	90.5	17.58	1.20	0.00	0.06	0.16	0.24	0.21	0.18
SP	SM_R4C7	4.23×10^{5}	4.173×10^{-2}	98.6	13.99	1.20	0.15	0.15	0.19	0.26	0.26	0.21
SP	SM_R4C8	4.40×10^{5}	4.068×10^{-2}	92.5	13.73	1.22	0.22	0.17	0.19	0.26	0.24	0.20
SP	SM_R4C9	4.50×10^{5}	4.185×10^{-2}	92.9	16.49	1.22	0.20	0.15	0.23	0.28	0.26	0.21
SP	SM_R5C1	4.54×10^{5}	3.770×10^{-2}	83.1	25.30	1.21	0.12	0.17	0.25	0.33	0.28	0.18
SP	SM_R5C2	4.30×10^{5}	3.889×10^{-2}	90.4	24.10	1.22	0.11	0.17	0.21	0.28	0.27	0.18
SP	SM_R5C3	4.26×10^{5}	3.841×10^{-2}	90.1	18.48	1.21	0.07	0.12	0.21	0.30	0.25	0.20
SP	SM_R5C4	4.41×10^{5}	4.070×10^{-2}	92.2	13.50	1.24	0.07	0.13	0.14	0.25	0.28	0.16
SP	SM_R5C5	4.42×10^{5}	3.847×10^{-2}	87.1	17.82	1.20	0.25	0.21	0.23	0.29	0.28	0.21
SP	SM_R5C6	4.40×10^{5}	3.965×10^{-2}	90.2	22.33	1.18	0.11	0.16	0.21	0.25	0.24	0.20
SP	SM_R5C7	4.28×10^{5}	3.959×10^{-2}	92.4	18.70	1.22	0.25	0.20	0.20	0.25	0.28	0.20
SP	SM_R5C8	4.16×10^{5}	3.964×10^{-2}	95.3	14.97	1.21	0.11	0.13	0.19	0.24	0.25	0.20
SP	SM_R5C9	4.29×10^{5}	3.942×10^{-2}	91.9	15.46	1.20	0.18	0.17	0.21	0.26	0.27	0.21
SP	SM_R6C1	3.91×10^{5}	3.568×10^{-2}	91.2	19.51	1.18	0.19	0.19	0.24	0.31	0.26	0.18
SP	SM_R6C2	3.82×10^{5}	3.707×10^{-2}	97.1	25.29	1.23	0.00	0.08	0.18	0.27	0.24	0.16
SP	SM_R6C3	4.13×10^{5}	3.665×10^{-2}	88.7	23.70	1.21	0.05	0.17	0.21	0.28	0.27	0.17
SP	SM_R6C4	4.30×10^{5}	3.797×10^{-2}	88.3	18.35	1.21	0.07	0.12	0.17	0.25	0.25	0.17
SP	SM_R6C5	4.22×10^{5}	3.691×10^{-2}	87.4	14.93	1.20	0.19	0.16	0.21	0.26	0.26	0.18
SP	SM_R6C6	4.00×10^{5}	3.684×10^{-2}	92.0	19.82	1.21	0.03	0.12	0.17	0.24	0.24	0.17
SP	SM_R6C7	3.95×10^{5}	3.856×10^{-2}	97.6	24.19	1.22	0.10	0.14	0.16	0.23	0.25	0.18
SP	SM_R6C8	3.89×10^{5}	3.765×10^{-2}	96.8	21.75	1.21	0.17	0.14	0.17	0.24	0.24	0.19
SP	SM_R6C9	3.81×10^{5}	3.770×10^{-2}	98.9	15.48	1.23	0.03	0.10	0.16	0.23	0.23	0.18
**Variant b (12 sensors)**												
MP-1	SM_R2C2	2.25×10^{5}	4.229×10^{-2}	188.4	116.45	1.41	0.08	0.14	0.21	0.34	0.33	0.28
MP-1	SM_R4C7	1.85×10^{5}	4.231×10^{-2}	228.8	63.21	1.49	0.00	0.05	0.13	0.28	0.29	0.25
MP-1	SM_R7C4	2.41×10^{5}	4.755×10^{-2}	197.5	75.71	1.41	0.10	0.11	0.20	0.33	0.32	0.27
MP-2	SM_R2C2	1.77×10^{5}	4.042×10^{-2}	228.9	101.99	1.52	0.00	0.10	0.15	0.38	0.40	0.30
MP-2	SM_R4C7	1.53×10^{5}	4.057×10^{-2}	264.5	35.96	1.58	0.13	0.13	0.17	0.39	0.47	0.34
MP-2	SM_R7C4	1.86×10^{5}	4.496×10^{-2}	241.7	58.84	1.54	0.00	0.01	0.11	0.37	0.42	0.30
MP-3	SM_R2C2	1.93×10^{5}	3.623×10^{-2}	187.4	99.01	1.43	0.25	0.12	0.20	0.40	0.43	0.34
MP-3	SM_R4C7	1.83×10^{5}	3.872×10^{-2}	211.6	33.43	1.50	0.04	0.15	0.19	0.35	0.50	0.33
MP-3	SM_R7C4	1.98×10^{5}	4.247×10^{-2}	214.9	58.56	1.49	0.11	0.08	0.16	0.37	0.42	0.33
MP-4	SM_R2C2	1.92×10^{5}	3.614×10^{-2}	188.1	95.52	1.39	0.11	0.12	0.18	0.40	0.40	0.40
MP-4	SM_R4C7	1.44×10^{5}	3.563×10^{-2}	247.1	35.71	1.43	0.17	0.10	0.16	0.32	0.35	0.40
MP-4	SM_R7C4	2.01×10^{5}	3.744×10^{-2}	185.9	53.81	1.34	0.10	0.14	0.18	0.41	0.40	0.38

Kinetic parameters for compound 14. SP = singleplex, MP-1/2/3/4 = multiplexed chip SCK-1/2/3/4. $f_{s,i}$ = non-dissociating fraction for cycle i.

Compound 15

Source	Sensor	k_a ($M^{-1}s^{-1}$)	k_d (s^{-1})	K_D (nM)	R_{max} (pg/mm^2)	$R_{max,n}$	$f_{s,1}$	$f_{s,2}$	$f_{s,3}$	$f_{s,4}$	$f_{s,5}$	$f_{s,6}$
**Variant a (54 sensors)**												
SP	SM_R1C1	1.08×10^{6}	2.456×10^{-2}	22.7	3.78	1.02	0.31	0.19	0.26	0.25	0.19	0.05
SP	SM_R1C2	9.54×10^{5}	2.173×10^{-2}	22.8	6.02	0.96	0.19	0.16	0.22	0.21	0.13	0.00
SP	SM_R1C3	9.53×10^{5}	2.543×10^{-2}	26.7	9.42	1.02	0.13	0.14	0.23	0.25	0.18	0.04
SP	SM_R1C4	1.03×10^{6}	2.496×10^{-2}	24.2	8.27	1.02	0.27	0.23	0.28	0.30	0.23	0.08
SP	SM_R1C5	9.81×10^{5}	2.821×10^{-2}	28.8	7.48	1.04	0.11	0.14	0.21	0.23	0.17	0.02
SP	SM_R1C6	9.36×10^{5}	2.439×10^{-2}	26.0	8.57	1.00	0.21	0.17	0.24	0.26	0.20	0.05
SP	SM_R1C7	9.19×10^{5}	2.377×10^{-2}	25.9	8.84	1.02	0.19	0.15	0.24	0.25	0.18	0.04
SP	SM_R1C8	9.73×10^{5}	2.491×10^{-2}	25.6	7.81	0.99	0.21	0.17	0.26	0.26	0.20	0.05
SP	SM_R1C9	9.95×10^{5}	2.535×10^{-2}	25.5	6.73	1.02	0.24	0.21	0.27	0.29	0.22	0.06
SP	SM_R2C1	9.52×10^{5}	2.577×10^{-2}	27.1	8.13	1.04	0.21	0.19	0.28	0.28	0.19	0.07
SP	SM_R2C2	9.37×10^{5}	2.467×10^{-2}	26.3	9.75	1.02	0.24	0.22	0.27	0.28	0.22	0.09
SP	SM_R2C3	8.87×10^{5}	2.508×10^{-2}	28.3	10.80	1.04	0.21	0.18	0.24	0.25	0.19	0.08
SP	SM_R2C4	8.83×10^{5}	2.284×10^{-2}	25.9	9.81	1.02	0.25	0.19	0.26	0.27	0.21	0.09
SP	SM_R2C5	9.92×10^{5}	2.502×10^{-2}	25.2	7.94	1.01	0.23	0.20	0.26	0.27	0.22	0.09
SP	SM_R2C6	8.28×10^{5}	2.401×10^{-2}	29.0	8.20	1.04	0.14	0.13	0.21	0.24	0.19	0.07
SP	SM_R2C7	8.17×10^{5}	2.405×10^{-2}	29.4	9.58	1.06	0.18	0.13	0.22	0.24	0.19	0.09
SP	SM_R2C8	8.53×10^{5}	2.644×10^{-2}	31.0	9.66	1.05	0.19	0.17	0.23	0.26	0.22	0.10
SP	SM_R2C9	7.52×10^{5}	2.668×10^{-2}	35.5	8.58	1.06	0.12	0.11	0.21	0.23	0.20	0.09
SP	SM_R3C1	8.55×10^{5}	2.281×10^{-2}	26.7	8.97	1.04	0.24	0.18	0.25	0.26	0.18	0.06
SP	SM_R3C2	9.50×10^{5}	2.359×10^{-2}	24.8	7.63	1.03	0.26	0.22	0.29	0.27	0.20	0.08
SP	SM_R3C3	8.38×10^{5}	2.341×10^{-2}	27.9	9.62	1.03	0.19	0.16	0.23	0.24	0.19	0.06
SP	SM_R3C4	8.85×10^{5}	2.449×10^{-2}	27.7	11.29	1.07	0.23	0.18	0.25	0.26	0.21	0.10
SP	SM_R3C5	8.40×10^{5}	2.277×10^{-2}	27.1	9.88	1.02	0.21	0.17	0.23	0.25	0.19	0.07
SP	SM_R3C6	9.41×10^{5}	2.437×10^{-2}	25.9	7.73	1.03	0.24	0.19	0.24	0.25	0.22	0.10
SP	SM_R3C7	8.37×10^{5}	2.368×10^{-2}	28.3	7.87	1.00	0.19	0.15	0.22	0.24	0.21	0.08
SP	SM_R3C8	8.78×10^{5}	2.436×10^{-2}	27.7	8.20	1.04	0.28	0.22	0.27	0.26	0.23	0.11
SP	SM_R3C9	8.07×10^{5}	2.442×10^{-2}	30.3	8.73	1.04	0.21	0.18	0.22	0.24	0.20	0.09
SP	SM_R4C1	8.51×10^{5}	2.242×10^{-2}	26.3	10.88	1.05	0.21	0.18	0.26	0.26	0.18	0.05
SP	SM_R4C2	8.39×10^{5}	2.281×10^{-2}	27.2	8.67	1.03	0.23	0.18	0.25	0.25	0.19	0.08
SP	SM_R4C3	8.36×10^{5}	2.265×10^{-2}	27.1	7.24	1.02	0.20	0.15	0.22	0.25	0.18	0.06
SP	SM_R4C4	8.37×10^{5}	2.339×10^{-2}	27.9	7.74	1.05	0.19	0.15	0.23	0.22	0.17	0.04
SP	SM_R4C5	8.85×10^{5}	2.355×10^{-2}	26.6	9.06	1.00	0.20	0.15	0.23	0.24	0.18	0.07
SP	SM_R4C6	7.76×10^{5}	2.183×10^{-2}	28.1	8.10	1.00	0.20	0.14	0.22	0.22	0.18	0.06
SP	SM_R4C7	7.54×10^{5}	2.376×10^{-2}	31.5	6.37	1.07	0.12	0.09	0.17	0.21	0.17	0.05
SP	SM_R4C8	8.38×10^{5}	2.482×10^{-2}	29.6	6.95	1.03	0.22	0.15	0.23	0.23	0.19	0.08
SP	SM_R4C9	7.33×10^{5}	2.271×10^{-2}	31.0	7.76	1.03	0.19	0.14	0.19	0.23	0.18	0.06
SP	SM_R5C1	8.28×10^{5}	2.360×10^{-2}	28.5	11.72	1.08	0.21	0.19	0.26	0.27	0.19	0.07
SP	SM_R5C2	8.13×10^{5}	2.401×10^{-2}	29.5	11.73	1.07	0.21	0.19	0.25	0.26	0.20	0.08
SP	SM_R5C3	7.53×10^{5}	2.378×10^{-2}	31.6	9.19	1.04	0.17	0.14	0.21	0.24	0.19	0.07
SP	SM_R5C4	8.13×10^{5}	2.364×10^{-2}	29.1	6.91	1.07	0.21	0.15	0.23	0.24	0.17	0.06
SP	SM_R5C5	7.44×10^{5}	2.315×10^{-2}	31.1	8.23	1.03	0.18	0.17	0.22	0.23	0.18	0.08
SP	SM_R5C6	7.34×10^{5}	2.188×10^{-2}	29.8	9.92	1.04	0.20	0.14	0.21	0.22	0.18	0.07
SP	SM_R5C7	7.41×10^{5}	2.213×10^{-2}	29.9	8.59	1.03	0.22	0.16	0.22	0.22	0.19	0.08
SP	SM_R5C8	7.78×10^{5}	2.294×10^{-2}	29.5	6.57	1.02	0.18	0.14	0.20	0.20	0.17	0.07
SP	SM_R5C9	7.53×10^{5}	2.272×10^{-2}	30.2	7.40	1.05	0.23	0.17	0.23	0.24	0.21	0.08
SP	SM_R6C1	7.44×10^{5}	2.370×10^{-2}	31.9	8.91	1.03	0.16	0.16	0.24	0.24	0.17	0.05
SP	SM_R6C2	7.28×10^{5}	2.279×10^{-2}	31.3	12.30	1.05	0.20	0.17	0.24	0.25	0.18	0.06
SP	SM_R6C3	7.35×10^{5}	2.233×10^{-2}	30.4	11.61	1.05	0.18	0.15	0.23	0.24	0.18	0.06
SP	SM_R6C4	7.39×10^{5}	2.198×10^{-2}	29.7	9.07	1.03	0.16	0.13	0.20	0.22	0.16	0.05
SP	SM_R6C5	8.13×10^{5}	2.295×10^{-2}	28.2	7.31	1.02	0.21	0.18	0.24	0.24	0.18	0.09
SP	SM_R6C6	7.21×10^{5}	2.234×10^{-2}	31.0	9.53	1.06	0.17	0.16	0.20	0.22	0.18	0.07
SP	SM_R6C7	6.98×10^{5}	2.251×10^{-2}	32.2	11.37	1.05	0.17	0.15	0.20	0.22	0.17	0.06
SP	SM_R6C8	7.36×10^{5}	2.219×10^{-2}	30.2	10.61	1.06	0.21	0.16	0.21	0.22	0.18	0.08
SP	SM_R6C9	7.03×10^{5}	2.113×10^{-2}	30.1	7.47	1.02	0.25	0.18	0.22	0.23	0.18	0.08
**Variant b (12 sensors)**												
MP-1	SM_R3C2	3.78×10^{5}	3.040×10^{-2}	80.5	46.27	1.18	0.00	0.05	0.18	0.31	0.26	0.20
MP-1	SM_R5C7	3.20×10^{5}	3.092×10^{-2}	96.7	50.59	1.23	0.00	0.00	0.10	0.28	0.24	0.19
MP-1	SM_R8C4	3.51×10^{5}	3.413×10^{-2}	97.4	55.53	1.21	0.00	0.00	0.10	0.23	0.20	0.17
MP-2	SM_R3C2	3.10×10^{5}	3.127×10^{-2}	100.9	48.64	1.29	0.00	0.05	0.13	0.40	0.36	0.25
MP-2	SM_R5C7	2.55×10^{5}	3.105×10^{-2}	121.7	34.60	1.33	0.00	0.01	0.07	0.35	0.38	0.25
MP-2	SM_R8C4	3.31×10^{5}	3.515×10^{-2}	106.2	48.71	1.29	0.00	0.00	0.09	0.39	0.37	0.25
MP-3	SM_R3C2	3.31×10^{5}	2.983×10^{-2}	90.2	49.92	1.21	0.02	0.06	0.14	0.42	0.41	0.26
MP-3	SM_R5C7	2.73×10^{5}	2.977×10^{-2}	109.1	33.71	1.23	0.10	0.05	0.10	0.31	0.39	0.25
MP-3	SM_R8C4	3.57×10^{5}	3.470×10^{-2}	97.3	48.45	1.22	0.00	0.04	0.13	0.40	0.40	0.28
MP-4	SM_R3C2	3.14×10^{5}	2.727×10^{-2}	86.9	48.12	1.20	0.02	0.06	0.16	0.43	0.37	0.33
MP-4	SM_R5C7	2.35×10^{5}	2.802×10^{-2}	119.2	34.74	1.20	0.05	0.05	0.10	0.32	0.32	0.33
MP-4	SM_R8C4	3.30×10^{5}	3.041×10^{-2}	92.2	46.80	1.18	0.00	0.03	0.13	0.41	0.36	0.34

Kinetic parameters for compound 15. SP = singleplex, MP-1/2/3/4 = multiplexed chip SCK-1/2/3/4. $f_{s,i}$ = non-dissociating fraction for cycle i.

Compound 16

Source	Sensor	k_a ($M^{-1}s^{-1}$)	k_d (s^{-1})	K_D (nM)	R_{max} (pg/mm^2)	$R_{max,n}$	$f_{s,1}$	$f_{s,2}$	$f_{s,3}$	$f_{s,4}$	$f_{s,5}$	$f_{s,6}$
**Variant a (54 sensors)**												
SP	SM_R1C1	7.70×10^5	2.908×10^{-2}	37.8	4.70	0.97	0.15	0.20	0.34	0.32	0.17	0.00
SP	SM_R1C2	6.58×10^5	3.447×10^{-2}	52.4	9.24	1.07	0.20	0.22	0.30	0.40	0.27	0.11
SP	SM_R1C3	6.65×10^5	4.172×10^{-2}	62.7	15.82	1.13	0.12	0.23	0.32	0.36	0.30	0.15
SP	SM_R1C4	6.83×10^5	4.264×10^{-2}	62.4	13.05	1.11	0.24	0.25	0.30	0.37	0.32	0.19
SP	SM_R1C5	6.13×10^5	4.000×10^{-2}	65.2	11.81	1.12	0.17	0.23	0.28	0.32	0.27	0.15
SP	SM_R1C6	6.64×10^5	4.094×10^{-2}	61.6	14.70	1.13	0.25	0.25	0.30	0.36	0.30	0.19
SP	SM_R1C7	6.27×10^5	3.936×10^{-2}	62.8	14.84	1.14	0.19	0.23	0.30	0.34	0.28	0.16
SP	SM_R1C8	6.89×10^5	4.074×10^{-2}	59.1	12.39	1.15	0.12	0.22	0.31	0.36	0.26	0.15
SP	SM_R1C9	6.83×10^5	4.129×10^{-2}	60.4	9.13	1.11	0.20	0.16	0.26	0.29	0.24	0.13
SP	SM_R2C1	6.96×10^5	4.052×10^{-2}	58.3	12.92	1.11	0.26	0.30	0.37	0.39	0.32	0.16
SP	SM_R2C2	6.55×10^5	4.084×10^{-2}	62.3	14.84	1.16	0.15	0.22	0.27	0.35	0.26	0.12
SP	SM_R2C3	6.70×10^5	4.094×10^{-2}	61.1	17.33	1.14	0.18	0.25	0.31	0.36	0.29	0.15
SP	SM_R2C4	6.77×10^5	3.920×10^{-2}	57.9	15.60	1.14	0.16	0.24	0.31	0.35	0.28	0.15
SP	SM_R2C5	7.01×10^5	4.459×10^{-2}	63.6	12.75	1.15	0.17	0.18	0.23	0.31	0.28	0.17
SP	SM_R2C6	6.20×10^5	4.118×10^{-2}	66.4	12.57	1.18	0.04	0.17	0.24	0.31	0.26	0.17
SP	SM_R2C7	6.10×10^5	3.977×10^{-2}	65.2	13.97	1.15	0.17	0.24	0.25	0.31	0.29	0.20
SP	SM_R2C8	6.62×10^5	4.111×10^{-2}	62.1	13.89	1.13	0.18	0.25	0.30	0.32	0.29	0.20
SP	SM_R2C9	5.69×10^5	4.393×10^{-2}	77.2	12.61	1.17	0.14	0.17	0.23	0.31	0.27	0.19
SP	SM_R3C1	7.18×10^5	4.101×10^{-2}	57.1	14.15	1.12	0.23	0.28	0.31	0.36	0.26	0.15
SP	SM_R3C2	6.66×10^5	3.971×10^{-2}	59.6	12.15	1.13	0.18	0.25	0.30	0.34	0.28	0.16
SP	SM_R3C3	7.06×10^5	4.200×10^{-2}	59.5	14.87	1.13	0.29	0.29	0.30	0.32	0.31	0.20
SP	SM_R3C4	6.02×10^5	3.896×10^{-2}	64.8	16.21	1.13	0.15	0.23	0.26	0.33	0.26	0.17
SP	SM_R3C5	5.78×10^5	3.975×10^{-2}	68.8	14.33	1.14	0.24	0.22	0.27	0.32	0.29	0.21
SP	SM_R3C6	6.39×10^5	4.532×10^{-2}	70.9	11.18	1.17	0.11	0.17	0.26	0.32	0.30	0.18
SP	SM_R3C7	5.83×10^5	4.043×10^{-2}	69.3	11.54	1.15	0.33	0.31	0.31	0.31	0.32	0.22
SP	SM_R3C8	6.05×10^5	4.149×10^{-2}	68.6	12.02	1.15	0.22	0.27	0.31	0.32	0.31	0.21
SP	SM_R3C9	5.72×10^5	4.182×10^{-2}	73.1	12.84	1.16	0.14	0.23	0.28	0.32	0.28	0.21
SP	SM_R4C1	6.30×10^5	3.884×10^{-2}	61.7	18.17	1.12	0.24	0.32	0.34	0.34	0.29	0.17
SP	SM_R4C2	5.89×10^5	4.014×10^{-2}	68.2	13.60	1.15	0.21	0.25	0.30	0.33	0.27	0.15
SP	SM_R4C3	6.95×10^5	4.097×10^{-2}	58.9	11.41	1.11	0.32	0.30	0.35	0.35	0.30	0.20
SP	SM_R4C4	6.53×10^5	4.487×10^{-2}	68.7	12.09	1.14	0.11	0.13	0.24	0.31	0.25	0.15
SP	SM_R4C5	6.86×10^5	4.598×10^{-2}	67.0	14.53	1.16	0.10	0.21	0.25	0.33	0.28	0.18
SP	SM_R4C6	6.83×10^5	4.451×10^{-2}	65.1	13.10	1.15	0.17	0.19	0.28	0.34	0.30	0.19
SP	SM_R4C7	5.64×10^5	4.073×10^{-2}	72.2	10.70	1.14	0.12	0.24	0.23	0.29	0.29	0.22
SP	SM_R4C8	5.50×10^5	3.872×10^{-2}	70.4	10.54	1.13	0.12	0.23	0.26	0.28	0.26	0.19
SP	SM_R4C9	5.50×10^5	3.925×10^{-2}	71.3	12.43	1.12	0.26	0.26	0.29	0.27	0.26	0.21
SP	SM_R5C1	5.38×10^5	3.667×10^{-2}	68.2	18.84	1.15	0.28	0.30	0.34	0.36	0.30	0.19
SP	SM_R5C2	5.40×10^5	3.918×10^{-2}	72.5	17.62	1.16	0.06	0.16	0.29	0.36	0.27	0.17
SP	SM_R5C3	5.82×10^5	3.850×10^{-2}	66.1	13.92	1.11	0.24	0.25	0.31	0.34	0.29	0.22
SP	SM_R5C4	5.45×10^5	4.056×10^{-2}	74.4	10.66	1.14	0.31	0.31	0.33	0.32	0.30	0.22
SP	SM_R5C5	4.68×10^5	3.905×10^{-2}	83.4	13.31	1.10	0.00	0.13	0.28	0.26	0.22	0.17
SP	SM_R5C6	5.23×10^5	4.017×10^{-2}	76.8	16.75	1.13	0.08	0.18	0.27	0.30	0.26	0.17
SP	SM_R5C7	5.44×10^5	4.306×10^{-2}	79.1	14.45	1.16	0.24	0.28	0.27	0.31	0.30	0.23
SP	SM_R5C8	5.83×10^5	4.429×10^{-2}	75.9	11.39	1.16	0.26	0.23	0.28	0.31	0.31	0.21
SP	SM_R5C9	5.31×10^5	4.062×10^{-2}	76.4	11.10	1.12	0.31	0.26	0.26	0.31	0.28	0.21
SP	SM_R6C1	4.82×10^5	3.453×10^{-2}	71.7	14.47	1.11	0.27	0.31	0.36	0.34	0.29	0.22
SP	SM_R6C2	4.61×10^5	3.469×10^{-2}	75.2	18.72	1.13	0.06	0.21	0.34	0.32	0.25	0.17
SP	SM_R6C3	5.27×10^5	3.629×10^{-2}	68.9	17.40	1.11	0.12	0.24	0.30	0.36	0.26	0.19
SP	SM_R6C4	5.43×10^5	3.805×10^{-2}	70.1	13.99	1.12	0.23	0.22	0.34	0.32	0.27	0.21
SP	SM_R6C5	5.66×10^5	4.266×10^{-2}	75.4	11.49	1.14	0.10	0.23	0.27	0.32	0.30	0.21
SP	SM_R6C6	5.08×10^5	3.913×10^{-2}	77.0	14.93	1.13	0.26	0.30	0.29	0.30	0.28	0.23
SP	SM_R6C7	4.89×10^5	3.928×10^{-2}	80.3	18.67	1.14	0.31	0.28	0.31	0.31	0.30	0.23
SP	SM_R6C8	4.96×10^5	3.933×10^{-2}	79.3	16.40	1.14	0.25	0.27	0.28	0.32	0.29	0.24
SP	SM_R6C9	5.57×10^5	4.196×10^{-2}	75.4	11.88	1.13	0.22	0.26	0.31	0.32	0.29	0.23
**Variant b (12 sensors)**												
MP-1	SM_R3C1	3.41×10^5	3.062×10^{-2}	89.7	14.96	1.18	0.14	0.31	0.55	0.40	0.44	0.07
MP-1	SM_R5C6	2.23×10^5	4.197×10^{-2}	188.1	19.88	1.40	0.10	0.16	0.28	0.26	0.41	0.22
MP-1	SM_R8C3	2.09×10^5	3.607×10^{-2}	172.6	14.87	1.31	0.00	0.12	0.35	0.23	0.38	0.10
MP-2	SM_R3C1	3.34×10^5	4.408×10^{-2}	132.0	18.16	1.26	0.19	0.27	0.43	0.49	0.66	0.31
MP-2	SM_R5C6	2.25×10^5	4.526×10^{-2}	201.2	15.69	1.48	0.01	0.00	0.13	0.29	0.55	0.31
MP-2	SM_R8C3	1.30×10^6	1.019×10^{-1}	78.5	6.21	0.75	0.02	0.41	0.50	0.68	1.00	0.55
MP-3	SM_R3C1	4.16×10^5	3.929×10^{-2}	94.5	17.49	1.07	0.00	0.26	0.43	0.55	0.68	0.20
MP-3	SM_R5C6	2.62×10^5	4.093×10^{-2}	156.1	15.13	1.28	0.28	0.13	0.25	0.36	0.61	0.26
MP-3	SM_R8C3	6.98×10^5	1.014×10^{-1}	145.3	9.79	1.07	0.21	0.26	0.41	0.50	0.88	0.22
MP-4	SM_R3C1	2.84×10^5	3.169×10^{-2}	111.5	19.39	1.21	0.18	0.26	0.39	0.52	0.51	0.32
MP-4	SM_R5C6	1.75×10^5	3.349×10^{-2}	191.6	16.83	1.25	0.12	0.19	0.26	0.31	0.42	0.40
MP-4	SM_R8C3	2.74×10^5	5.510×10^{-2}	201.0	13.07	1.19	0.00	0.26	0.42	0.46	0.51	0.39

Kinetic parameters for compound 16. SP = singleplex, MP-1/2/3/4 = multiplexed chip SCK-1/2/3/4. $f_{s,i}$ = non-dissociating fraction for cycle i.

Compound 17

Source	Sensor	k_a ($M^{-1}s^{-1}$)	k_d (s^{-1})	K_D (nM)	R_{max} (pg/mm^2)	$R_{max,n}$	$f_{s,1}$	$f_{s,2}$	$f_{s,3}$	$f_{s,4}$	$f_{s,5}$	$f_{s,6}$
**Variant a (54 sensors)**												
SP	SM_R1C1	3.90×10^{5}	2.836×10^{-2}	72.7	7.24	1.11	0.31	0.31	0.29	0.30	0.23	0.10
SP	SM_R1C2	2.94×10^{5}	2.945×10^{-2}	100.2	11.75	1.16	0.20	0.25	0.26	0.29	0.23	0.16
SP	SM_R1C3	3.48×10^{5}	3.522×10^{-2}	101.3	19.19	1.20	0.21	0.26	0.26	0.33	0.27	0.18
SP	SM_R1C4	3.39×10^{5}	3.584×10^{-2}	105.8	15.66	1.19	0.40	0.34	0.29	0.33	0.30	0.21
SP	SM_R1C5	3.27×10^{5}	3.500×10^{-2}	107.2	14.88	1.21	0.25	0.25	0.30	0.33	0.29	0.20
SP	SM_R1C6	3.07×10^{5}	3.361×10^{-2}	109.6	17.17	1.17	0.30	0.31	0.33	0.35	0.31	0.21
SP	SM_R1C7	3.19×10^{5}	3.391×10^{-2}	106.4	17.15	1.18	0.36	0.28	0.34	0.34	0.31	0.22
SP	SM_R1C8	3.27×10^{5}	3.388×10^{-2}	103.7	14.37	1.19	0.23	0.32	0.32	0.36	0.30	0.22
SP	SM_R1C9	3.53×10^{5}	3.467×10^{-2}	98.2	11.16	1.19	0.36	0.33	0.33	0.34	0.30	0.19
SP	SM_R2C1	3.60×10^{5}	3.450×10^{-2}	95.7	15.26	1.17	0.22	0.30	0.31	0.34	0.29	0.17
SP	SM_R2C2	3.51×10^{5}	3.575×10^{-2}	102.0	18.02	1.21	0.21	0.28	0.29	0.34	0.29	0.19
SP	SM_R2C3	3.36×10^{5}	3.322×10^{-2}	99.0	20.60	1.18	0.37	0.33	0.33	0.36	0.31	0.20
SP	SM_R2C4	3.58×10^{5}	3.588×10^{-2}	100.2	18.34	1.17	0.42	0.34	0.33	0.37	0.32	0.22
SP	SM_R2C5	3.21×10^{5}	3.672×10^{-2}	114.5	15.02	1.20	0.29	0.28	0.31	0.34	0.32	0.23
SP	SM_R2C6	3.21×10^{5}	3.610×10^{-2}	112.3	14.08	1.21	0.21	0.25	0.29	0.34	0.32	0.23
SP	SM_R2C7	3.56×10^{5}	3.485×10^{-2}	97.9	15.56	1.18	0.50	0.34	0.35	0.34	0.34	0.25
SP	SM_R2C8	3.17×10^{5}	3.710×10^{-2}	117.0	16.07	1.20	0.06	0.28	0.25	0.33	0.27	0.21
SP	SM_R2C9	3.17×10^{5}	3.648×10^{-2}	115.1	14.20	1.21	0.38	0.32	0.31	0.33	0.30	0.24
SP	SM_R3C1	3.95×10^{5}	3.637×10^{-2}	92.2	16.03	1.18	0.37	0.30	0.35	0.36	0.31	0.20
SP	SM_R3C2	3.53×10^{5}	3.517×10^{-2}	99.5	13.62	1.18	0.30	0.30	0.32	0.35	0.28	0.19
SP	SM_R3C3	3.56×10^{5}	3.634×10^{-2}	102.0	16.91	1.20	0.30	0.31	0.30	0.34	0.30	0.20
SP	SM_R3C4	3.46×10^{5}	3.545×10^{-2}	102.3	18.65	1.19	0.26	0.32	0.29	0.35	0.28	0.20
SP	SM_R3C5	3.29×10^{5}	3.459×10^{-2}	105.2	15.99	1.20	0.26	0.24	0.31	0.31	0.29	0.21
SP	SM_R3C6	3.55×10^{5}	3.784×10^{-2}	106.5	13.25	1.22	0.19	0.28	0.25	0.31	0.30	0.20
SP	SM_R3C7	3.21×10^{5}	3.468×10^{-2}	108.0	13.12	1.19	0.26	0.25	0.29	0.30	0.31	0.22
SP	SM_R3C8	3.49×10^{5}	3.717×10^{-2}	106.6	13.96	1.19	0.26	0.30	0.28	0.31	0.29	0.22
SP	SM_R3C9	3.18×10^{5}	3.888×10^{-2}	122.2	14.78	1.22	0.16	0.20	0.24	0.29	0.28	0.20
SP	SM_R4C1	3.56×10^{5}	3.304×10^{-2}	92.8	21.21	1.16	0.41	0.33	0.34	0.36	0.31	0.21
SP	SM_R4C2	3.56×10^{5}	3.597×10^{-2}	101.2	15.67	1.19	0.40	0.34	0.34	0.33	0.30	0.20
SP	SM_R4C3	3.44×10^{5}	3.661×10^{-2}	106.5	13.08	1.21	0.16	0.26	0.26	0.32	0.28	0.19
SP	SM_R4C4	3.35×10^{5}	3.791×10^{-2}	113.2	14.25	1.22	0.26	0.23	0.29	0.33	0.30	0.21
SP	SM_R4C5	3.30×10^{5}	3.546×10^{-2}	107.4	16.91	1.20	0.21	0.33	0.29	0.32	0.29	0.21
SP	SM_R4C6	3.13×10^{5}	3.669×10^{-2}	117.3	14.67	1.23	0.23	0.32	0.27	0.35	0.29	0.23
SP	SM_R4C7	3.24×10^{5}	3.497×10^{-2}	107.8	11.62	1.19	0.15	0.26	0.26	0.33	0.27	0.23
SP	SM_R4C8	3.10×10^{5}	3.813×10^{-2}	122.8	11.64	1.22	0.37	0.25	0.30	0.32	0.29	0.22
SP	SM_R4C9	3.10×10^{5}	3.698×10^{-2}	119.4	13.46	1.21	0.21	0.26	0.28	0.34	0.29	0.22
SP	SM_R5C1	3.40×10^{5}	3.161×10^{-2}	92.9	21.72	1.18	0.34	0.32	0.31	0.33	0.29	0.18
SP	SM_R5C2	3.18×10^{5}	3.465×10^{-2}	109.1	20.73	1.22	0.12	0.20	0.24	0.30	0.26	0.18
SP	SM_R5C3	3.07×10^{5}	3.571×10^{-2}	116.4	15.88	1.23	0.09	0.15	0.24	0.29	0.26	0.18
SP	SM_R5C4	3.50×10^{5}	3.326×10^{-2}	95.1	12.03	1.19	0.29	0.27	0.35	0.31	0.29	0.22
SP	SM_R5C5	3.04×10^{5}	3.995×10^{-2}	131.3	15.14	1.24	0.23	0.15	0.26	0.30	0.30	0.21
SP	SM_R5C6	3.26×10^{5}	3.887×10^{-2}	119.2	19.03	1.24	0.15	0.19	0.25	0.32	0.27	0.21
SP	SM_R5C7	3.11×10^{5}	3.689×10^{-2}	118.4	15.78	1.22	0.09	0.19	0.23	0.28	0.27	0.20
SP	SM_R5C8	3.26×10^{5}	3.776×10^{-2}	115.8	12.39	1.21	0.15	0.22	0.20	0.29	0.27	0.18
SP	SM_R5C9	3.03×10^{5}	3.773×10^{-2}	124.7	12.93	1.24	0.02	0.16	0.23	0.27	0.27	0.20
SP	SM_R6C1	2.97×10^{5}	3.128×10^{-2}	105.4	16.93	1.18	0.23	0.27	0.29	0.32	0.27	0.19
SP	SM_R6C2	3.19×10^{5}	3.489×10^{-2}	109.2	21.85	1.23	0.18	0.22	0.24	0.31	0.27	0.18
SP	SM_R6C3	3.18×10^{5}	3.307×10^{-2}	103.9	20.30	1.21	0.28	0.28	0.27	0.33	0.28	0.19
SP	SM_R6C4	3.21×10^{5}	3.407×10^{-2}	106.1	15.48	1.22	0.25	0.23	0.29	0.33	0.28	0.20
SP	SM_R6C5	2.93×10^{5}	3.459×10^{-2}	117.9	12.80	1.23	0.05	0.28	0.23	0.30	0.25	0.18
SP	SM_R6C6	3.18×10^{5}	3.519×10^{-2}	110.5	16.82	1.23	0.30	0.30	0.26	0.31	0.28	0.20
SP	SM_R6C7	3.05×10^{5}	3.653×10^{-2}	119.7	20.88	1.24	0.18	0.23	0.24	0.28	0.28	0.19
SP	SM_R6C8	2.93×10^{5}	3.369×10^{-2}	114.8	17.96	1.22	0.21	0.23	0.26	0.30	0.26	0.19
SP	SM_R6C9	3.27×10^{5}	3.778×10^{-2}	115.6	12.70	1.22	0.41	0.29	0.30	0.30	0.31	0.23
**Variant b (12 sensors)**												
MP-1	SM_R2C5	2.44×10^{5}	3.400×10^{-2}	139.1	111.32	1.32	0.02	0.06	0.16	0.31	0.29	0.26
MP-1	SM_R5C2	3.53×10^{5}	4.204×10^{-2}	119.0	56.10	1.26	0.03	0.10	0.23	0.38	0.35	0.30
MP-1	SM_R7C7	2.90×10^{5}	3.961×10^{-2}	136.7	70.44	1.31	0.03	0.08	0.18	0.29	0.31	0.26
MP-2	SM_R2C5	2.03×10^{5}	3.256×10^{-2}	160.2	66.74	1.38	0.06	0.06	0.13	0.36	0.39	0.30
MP-2	SM_R5C2	3.03×10^{5}	4.054×10^{-2}	133.6	55.19	1.36	0.00	0.06	0.16	0.46	0.44	0.31
MP-2	SM_R7C7	2.27×10^{5}	3.957×10^{-2}	174.2	33.39	1.43	0.00	0.01	0.10	0.33	0.44	0.31
MP-3	SM_R2C5	2.18×10^{5}	3.117×10^{-2}	143.1	62.94	1.36	0.15	0.12	0.18	0.37	0.43	0.33
MP-3	SM_R5C2	3.20×10^{5}	4.034×10^{-2}	126.0	57.21	1.31	0.14	0.06	0.20	0.47	0.49	0.37
MP-3	SM_R7C7	2.64×10^{5}	3.977×10^{-2}	150.3	31.93	1.35	0.25	0.11	0.18	0.34	0.50	0.34
MP-4	SM_R2C5	2.11×10^{5}	3.088×10^{-2}	146.4	63.68	1.29	0.08	0.11	0.17	0.37	0.40	0.39
MP-4	SM_R5C2	3.07×10^{5}	3.506×10^{-2}	114.2	53.39	1.24	0.07	0.11	0.23	0.50	0.46	0.41
MP-4	SM_R7C7	2.22×10^{5}	3.495×10^{-2}	157.2	32.54	1.26	0.00	0.10	0.17	0.34	0.38	0.42

Kinetic parameters for compound 17. SP = singleplex, MP-1/2/3/4 = multiplexed chip SCK-1/2/3/4. $f_{s,i}$ = non-dissociating fraction for cycle i.

Compound 18

Source	Sensor	k_a ($M^{-1}s^{-1}$)	k_d (s^{-1})	K_D (nM)	R_{max} (pg/mm^2)	$R_{max,n}$	$f_{s,1}$	$f_{s,2}$	$f_{s,3}$	$f_{s,4}$	$f_{s,5}$	$f_{s,6}$
**Variant a (54 sensors)**												
SP	SM_R1C1	5.50×10^{5}	2.898×10^{-2}	52.7	5.18	1.06	0.52	0.41	0.31	0.28	0.24	0.20
SP	SM_R1C2	5.47×10^{5}	2.814×10^{-2}	51.4	8.22	1.08	0.42	0.27	0.30	0.28	0.23	0.17
SP	SM_R1C3	4.75×10^{5}	3.329×10^{-2}	70.1	13.51	1.12	0.20	0.25	0.29	0.27	0.24	0.19
SP	SM_R1C4	5.63×10^{5}	3.125×10^{-2}	55.5	10.76	1.12	0.39	0.33	0.29	0.32	0.28	0.19
SP	SM_R1C5	4.93×10^{5}	3.020×10^{-2}	61.2	9.77	1.12	0.39	0.31	0.27	0.27	0.24	0.16
SP	SM_R1C6	4.80×10^{5}	3.028×10^{-2}	63.1	11.62	1.15	0.19	0.21	0.25	0.27	0.25	0.16
SP	SM_R1C7	4.86×10^{5}	3.059×10^{-2}	62.9	12.02	1.14	0.34	0.28	0.28	0.27	0.25	0.16
SP	SM_R1C8	5.22×10^{5}	3.039×10^{-2}	58.2	10.81	1.11	0.28	0.25	0.28	0.27	0.24	0.16
SP	SM_R1C9	5.49×10^{5}	3.257×10^{-2}	59.3	9.24	1.10	0.41	0.29	0.30	0.31	0.25	0.19
SP	SM_R2C1	4.49×10^{5}	2.875×10^{-2}	64.1	11.11	1.10	0.14	0.22	0.30	0.29	0.22	0.17
SP	SM_R2C2	4.97×10^{5}	2.973×10^{-2}	59.8	12.76	1.09	0.22	0.26	0.28	0.28	0.24	0.17
SP	SM_R2C3	4.79×10^{5}	3.031×10^{-2}	63.3	14.40	1.10	0.20	0.25	0.28	0.27	0.22	0.18
SP	SM_R2C4	4.96×10^{5}	2.912×10^{-2}	58.7	12.96	1.12	0.23	0.22	0.29	0.29	0.24	0.16
SP	SM_R2C5	4.53×10^{5}	2.995×10^{-2}	66.1	10.86	1.10	0.14	0.24	0.26	0.26	0.23	0.17
SP	SM_R2C6	4.54×10^{5}	3.198×10^{-2}	70.5	11.63	1.14	0.29	0.15	0.26	0.29	0.26	0.18
SP	SM_R2C7	4.60×10^{5}	3.133×10^{-2}	68.1	13.05	1.12	0.13	0.19	0.27	0.27	0.22	0.18
SP	SM_R2C8	4.05×10^{5}	3.280×10^{-2}	81.1	13.65	1.12	0.00	0.22	0.28	0.25	0.23	0.22
SP	SM_R2C9	4.02×10^{5}	3.160×10^{-2}	78.7	11.67	1.15	0.11	0.17	0.25	0.27	0.24	0.17
SP	SM_R3C1	4.69×10^{5}	2.811×10^{-2}	60.0	12.18	1.13	0.13	0.18	0.29	0.27	0.22	0.11
SP	SM_R3C2	4.78×10^{5}	2.898×10^{-2}	60.6	10.55	1.10	0.24	0.21	0.27	0.26	0.22	0.15
SP	SM_R3C3	4.46×10^{5}	2.843×10^{-2}	63.7	13.27	1.11	0.22	0.23	0.25	0.24	0.22	0.15
SP	SM_R3C4	4.70×10^{5}	2.935×10^{-2}	62.4	14.99	1.11	0.25	0.23	0.29	0.28	0.25	0.18
SP	SM_R3C5	4.73×10^{5}	2.761×10^{-2}	58.4	12.75	1.13	0.48	0.26	0.27	0.28	0.27	0.14
SP	SM_R3C6	5.02×10^{5}	3.007×10^{-2}	59.9	10.05	1.13	0.15	0.25	0.25	0.26	0.23	0.16
SP	SM_R3C7	5.08×10^{5}	3.042×10^{-2}	59.9	9.99	1.10	0.53	0.28	0.30	0.32	0.29	0.21
SP	SM_R3C8	4.59×10^{5}	2.768×10^{-2}	60.4	10.57	1.12	0.36	0.29	0.30	0.29	0.25	0.19
SP	SM_R3C9	4.64×10^{5}	2.963×10^{-2}	63.8	11.40	1.12	0.38	0.26	0.30	0.28	0.26	0.19
SP	SM_R4C1	4.61×10^{5}	2.586×10^{-2}	56.1	14.74	1.11	0.32	0.25	0.29	0.29	0.23	0.15
SP	SM_R4C2	4.77×10^{5}	2.771×10^{-2}	58.2	11.39	1.12	0.32	0.25	0.27	0.29	0.24	0.15
SP	SM_R4C3	4.50×10^{5}	2.592×10^{-2}	57.6	9.65	1.12	0.29	0.19	0.27	0.28	0.24	0.14
SP	SM_R4C4	4.37×10^{5}	2.710×10^{-2}	62.0	10.39	1.10	0.23	0.24	0.24	0.26	0.21	0.12
SP	SM_R4C5	4.52×10^{5}	2.912×10^{-2}	64.4	12.51	1.10	0.14	0.20	0.28	0.26	0.22	0.15
SP	SM_R4C6	4.20×10^{5}	2.655×10^{-2}	63.2	10.97	1.10	0.03	0.13	0.23	0.23	0.20	0.13
SP	SM_R4C7	3.84×10^{5}	3.015×10^{-2}	78.6	9.03	1.15	0.06	0.10	0.24	0.24	0.22	0.19
SP	SM_R4C8	4.92×10^{5}	2.778×10^{-2}	56.4	8.96	1.11	0.27	0.27	0.27	0.26	0.24	0.15
SP	SM_R4C9	4.31×10^{5}	2.691×10^{-2}	62.4	10.50	1.12	0.40	0.26	0.26	0.26	0.23	0.15
SP	SM_R5C1	4.31×10^{5}	2.716×10^{-2}	63.0	15.25	1.13	0.28	0.24	0.26	0.28	0.24	0.14
SP	SM_R5C2	4.26×10^{5}	2.742×10^{-2}	64.4	15.23	1.14	0.25	0.21	0.26	0.27	0.24	0.13
SP	SM_R5C3	4.21×10^{5}	2.731×10^{-2}	64.9	11.80	1.15	0.18	0.17	0.23	0.25	0.23	0.10
SP	SM_R5C4	4.26×10^{5}	2.653×10^{-2}	62.3	8.71	1.10	0.38	0.23	0.26	0.29	0.24	0.15
SP	SM_R5C5	4.33×10^{5}	2.558×10^{-2}	59.1	10.51	1.12	0.28	0.30	0.22	0.26	0.23	0.12
SP	SM_R5C6	4.14×10^{5}	2.524×10^{-2}	60.9	13.15	1.13	0.15	0.19	0.22	0.24	0.21	0.10
SP	SM_R5C7	4.37×10^{5}	2.630×10^{-2}	60.2	11.22	1.11	0.42	0.29	0.27	0.26	0.25	0.17
SP	SM_R5C8	4.18×10^{5}	2.595×10^{-2}	62.0	8.77	1.11	0.29	0.28	0.23	0.25	0.20	0.15
SP	SM_R5C9	4.14×10^{5}	2.688×10^{-2}	64.9	9.32	1.12	0.30	0.23	0.26	0.24	0.22	0.15
SP	SM_R6C1	4.10×10^{5}	2.608×10^{-2}	63.5	11.58	1.12	0.31	0.28	0.32	0.30	0.24	0.15
SP	SM_R6C2	4.16×10^{5}	2.609×10^{-2}	62.7	15.76	1.12	0.20	0.21	0.28	0.27	0.23	0.12
SP	SM_R6C3	4.25×10^{5}	2.538×10^{-2}	59.7	15.17	1.11	0.24	0.20	0.25	0.28	0.22	0.11
SP	SM_R6C4	4.51×10^{5}	2.462×10^{-2}	54.6	11.78	1.08	0.39	0.24	0.28	0.28	0.24	0.13
SP	SM_R6C5	4.13×10^{5}	2.731×10^{-2}	66.2	9.32	1.11	0.12	0.21	0.23	0.23	0.20	0.15
SP	SM_R6C6	3.91×10^{5}	2.795×10^{-2}	71.5	12.32	1.14	0.27	0.24	0.23	0.24	0.23	0.16
SP	SM_R6C7	4.08×10^{5}	2.711×10^{-2}	66.4	15.00	1.13	0.30	0.21	0.26	0.25	0.24	0.14
SP	SM_R6C8	4.16×10^{5}	2.804×10^{-2}	67.4	13.55	1.14	0.41	0.24	0.24	0.25	0.23	0.17
SP	SM_R6C9	3.82×10^{5}	2.451×10^{-2}	64.2	10.00	1.12	0.20	0.23	0.25	0.23	0.22	0.14
**Variant b (12 sensors)**												
MP-1	SM_R2C6	1.86×10^{5}	4.041×10^{-2}	217.6	85.46	1.46	0.00	0.06	0.14	0.29	0.33	0.30
MP-1	SM_R5C3	2.82×10^{5}	4.726×10^{-2}	167.8	91.55	1.36	0.01	0.11	0.24	0.36	0.37	0.32
MP-1	SM_R7C8	2.25×10^{5}	4.549×10^{-2}	202.2	49.29	1.39	0.13	0.16	0.21	0.29	0.35	0.31
MP-2	SM_R2C6	1.64×10^{5}	4.057×10^{-2}	246.8	50.55	1.51	0.00	0.11	0.17	0.31	0.44	0.37
MP-2	SM_R5C3	2.00×10^{5}	4.278×10^{-2}	213.7	80.16	1.47	0.00	0.07	0.13	0.40	0.40	0.32
MP-2	SM_R7C8	1.70×10^{5}	4.310×10^{-2}	253.1	40.97	1.54	0.00	0.03	0.13	0.33	0.46	0.35
MP-3	SM_R2C6	1.67×10^{5}	3.606×10^{-2}	215.7	47.70	1.46	0.31	0.14	0.20	0.38	0.50	0.37
MP-3	SM_R5C3	2.20×10^{5}	4.268×10^{-2}	194.0	83.11	1.41	0.33	0.12	0.22	0.43	0.47	0.40
MP-3	SM_R7C8	1.83×10^{5}	4.168×10^{-2}	227.2	41.88	1.52	0.23	0.01	0.14	0.32	0.49	0.35
MP-4	SM_R2C6	1.63×10^{5}	3.394×10^{-2}	208.0	50.02	1.37	0.23	0.19	0.23	0.37	0.43	0.44
MP-4	SM_R5C3	2.19×10^{5}	3.810×10^{-2}	174.3	77.99	1.34	0.08	0.15	0.21	0.44	0.44	0.43
MP-4	SM_R7C8	1.53×10^{5}	3.631×10^{-2}	237.2	42.15	1.41	0.04	0.09	0.15	0.33	0.39	0.43

Kinetic parameters for compound 18. SP = singleplex, MP-1/2/3/4 = multiplexed chip SCK-1/2/3/4. $f_{s,i}$ = non-dissociating fraction for cycle i.

Compound 19

Source	Sensor	k_a ($M^{-1}s^{-1}$)	k_d (s^{-1})	K_D (nM)	R_{max} (pg/mm^2)	$R_{max,n}$	$f_{s,1}$	$f_{s,2}$	$f_{s,3}$	$f_{s,4}$	$f_{s,5}$	$f_{s,6}$
**Variant a (54 sensors)**												
SP	SM_R1C1	1.10×10^{6}	2.694×10^{-2}	24.5	4.68	1.03	0.21	0.17	0.25	0.26	0.17	0.08
SP	SM_R1C2	9.82×10^{5}	2.306×10^{-2}	23.5	7.82	1.01	0.31	0.23	0.21	0.27	0.27	0.10
SP	SM_R1C3	8.97×10^{5}	2.609×10^{-2}	29.1	12.43	1.06	0.17	0.18	0.15	0.23	0.21	0.08
SP	SM_R1C4	9.06×10^{5}	2.530×10^{-2}	27.9	10.11	1.07	0.19	0.16	0.14	0.25	0.21	0.11
SP	SM_R1C5	8.90×10^{5}	2.513×10^{-2}	28.2	8.78	1.02	0.04	0.09	0.20	0.18	0.15	0.00
SP	SM_R1C6	8.43×10^{5}	2.489×10^{-2}	29.5	10.83	1.04	0.01	0.09	0.11	0.14	0.11	0.01
SP	SM_R1C7	8.50×10^{5}	2.403×10^{-2}	28.3	10.74	1.03	0.10	0.10	0.13	0.17	0.15	0.00
SP	SM_R1C8	8.49×10^{5}	2.800×10^{-2}	33.0	9.14	1.04	0.02	0.09	0.09	0.18	0.17	0.05
SP	SM_R1C9	8.39×10^{5}	3.040×10^{-2}	36.2	7.61	1.09	0.10	0.12	0.16	0.22	0.18	0.06
SP	SM_R2C1	9.60×10^{5}	2.839×10^{-2}	29.6	10.21	1.07	0.12	0.13	0.18	0.23	0.17	0.05
SP	SM_R2C2	9.19×10^{5}	2.486×10^{-2}	27.0	11.77	1.06	0.08	0.13	0.15	0.23	0.18	0.05
SP	SM_R2C3	9.05×10^{5}	2.627×10^{-2}	29.0	13.41	1.02	0.18	0.15	0.12	0.20	0.18	0.09
SP	SM_R2C4	9.13×10^{5}	2.623×10^{-2}	28.7	12.16	1.05	0.17	0.16	0.11	0.19	0.19	0.09
SP	SM_R2C5	9.28×10^{5}	2.560×10^{-2}	27.6	9.59	1.00	0.13	0.14	0.17	0.17	0.17	0.06
SP	SM_R2C6	9.27×10^{5}	2.623×10^{-2}	28.3	9.85	1.06	0.15	0.22	0.13	0.22	0.22	0.07
SP	SM_R2C7	7.88×10^{5}	2.662×10^{-2}	33.8	11.32	1.07	0.12	0.09	0.08	0.14	0.18	0.09
SP	SM_R2C8	7.82×10^{5}	2.957×10^{-2}	37.8	11.35	1.08	0.05	0.07	0.10	0.16	0.18	0.13
SP	SM_R2C9	7.80×10^{5}	2.778×10^{-2}	35.6	10.33	1.08	0.17	0.13	0.11	0.18	0.24	0.13
SP	SM_R3C1	9.95×10^{5}	2.593×10^{-2}	26.1	10.76	1.06	0.17	0.19	0.16	0.24	0.21	0.04
SP	SM_R3C2	8.77×10^{5}	2.489×10^{-2}	28.4	8.89	1.06	0.09	0.14	0.13	0.17	0.14	0.01
SP	SM_R3C3	9.40×10^{5}	2.746×10^{-2}	29.2	11.15	1.08	0.13	0.20	0.18	0.22	0.21	0.09
SP	SM_R3C4	8.69×10^{5}	2.463×10^{-2}	28.3	12.51	1.07	0.16	0.18	0.14	0.20	0.17	0.06
SP	SM_R3C5	8.88×10^{5}	2.502×10^{-2}	28.2	10.85	1.05	0.15	0.17	0.14	0.22	0.19	0.05
SP	SM_R3C6	7.96×10^{5}	2.461×10^{-2}	30.9	8.89	1.02	0.00	0.06	0.11	0.14	0.16	0.07
SP	SM_R3C7	7.65×10^{5}	2.428×10^{-2}	31.7	8.66	1.05	0.16	0.17	0.08	0.21	0.19	0.01
SP	SM_R3C8	7.99×10^{5}	2.636×10^{-2}	33.0	9.14	1.08	0.05	0.12	0.10	0.15	0.15	0.05
SP	SM_R3C9	7.83×10^{5}	2.655×10^{-2}	33.9	9.95	1.08	0.13	0.15	0.09	0.19	0.16	0.06
SP	SM_R4C1	9.36×10^{5}	2.397×10^{-2}	25.6	13.28	1.07	0.12	0.14	0.18	0.20	0.15	0.01
SP	SM_R4C2	8.79×10^{5}	2.417×10^{-2}	27.5	10.34	1.06	0.14	0.13	0.14	0.18	0.17	0.03
SP	SM_R4C3	9.03×10^{5}	2.442×10^{-2}	27.0	8.43	1.07	0.10	0.16	0.13	0.19	0.15	0.03
SP	SM_R4C4	8.55×10^{5}	2.403×10^{-2}	28.1	9.57	1.06	0.06	0.09	0.13	0.17	0.14	0.03
SP	SM_R4C5	8.49×10^{5}	2.473×10^{-2}	29.1	11.39	1.06	0.07	0.09	0.08	0.14	0.14	0.05
SP	SM_R4C6	8.93×10^{5}	2.493×10^{-2}	27.9	10.35	1.06	0.14	0.15	0.14	0.20	0.22	0.09
SP	SM_R4C7	8.35×10^{5}	2.502×10^{-2}	30.0	7.74	1.07	0.12	0.14	0.12	0.12	0.13	0.08
SP	SM_R4C8	8.39×10^{5}	2.330×10^{-2}	27.8	7.27	1.06	0.15	0.14	0.13	0.19	0.17	0.01
SP	SM_R4C9	8.42×10^{5}	2.518×10^{-2}	29.9	8.63	1.05	0.16	0.16	0.13	0.20	0.17	0.03
SP	SM_R5C1	8.23×10^{5}	2.553×10^{-2}	31.0	14.27	1.06	0.08	0.13	0.13	0.17	0.14	0.02
SP	SM_R5C2	7.65×10^{5}	2.611×10^{-2}	34.1	14.00	1.08	0.09	0.08	0.11	0.16	0.16	0.06
SP	SM_R5C3	7.42×10^{5}	2.428×10^{-2}	32.7	10.77	1.07	0.00	0.03	0.11	0.17	0.15	0.05
SP	SM_R5C4	7.15×10^{5}	2.470×10^{-2}	34.5	7.59	1.09	0.01	0.10	0.11	0.12	0.10	0.00
SP	SM_R5C5	7.26×10^{5}	2.463×10^{-2}	33.9	10.11	1.06	0.00	0.00	0.14	0.17	0.14	0.00
SP	SM_R5C6	7.58×10^{5}	2.432×10^{-2}	32.1	12.65	1.06	0.05	0.06	0.14	0.16	0.16	0.04
SP	SM_R5C7	7.01×10^{5}	2.408×10^{-2}	34.4	10.22	1.09	0.11	0.12	0.05	0.12	0.11	0.03
SP	SM_R5C8	7.69×10^{5}	2.397×10^{-2}	31.2	8.41	1.07	0.12	0.09	0.11	0.18	0.15	0.04
SP	SM_R5C9	6.99×10^{5}	2.532×10^{-2}	36.2	8.97	1.09	0.10	0.06	0.08	0.15	0.14	0.06
SP	SM_R6C1	7.83×10^{5}	2.506×10^{-2}	32.0	10.26	1.06	0.02	0.09	0.15	0.18	0.11	0.00
SP	SM_R6C2	7.66×10^{5}	2.285×10^{-2}	29.8	14.10	1.06	0.07	0.08	0.13	0.19	0.16	0.03
SP	SM_R6C3	7.26×10^{5}	2.186×10^{-2}	30.1	13.32	1.06	0.06	0.04	0.07	0.14	0.15	0.03
SP	SM_R6C4	7.92×10^{5}	2.158×10^{-2}	27.3	9.91	1.07	0.05	0.08	0.13	0.19	0.15	0.02
SP	SM_R6C5	7.59×10^{5}	2.263×10^{-2}	29.8	7.97	1.08	0.17	0.15	0.07	0.17	0.16	0.07
SP	SM_R6C6	7.23×10^{5}	2.238×10^{-2}	30.9	10.54	1.07	0.12	0.09	0.11	0.15	0.10	0.02
SP	SM_R6C7	7.38×10^{5}	2.287×10^{-2}	31.0	13.20	1.06	0.13	0.12	0.12	0.13	0.14	0.05
SP	SM_R6C8	6.86×10^{5}	2.268×10^{-2}	33.1	11.58	1.06	0.09	0.05	0.06	0.12	0.15	0.03
SP	SM_R6C9	7.10×10^{5}	2.200×10^{-2}	31.0	8.35	1.06	0.05	0.07	0.12	0.13	0.13	0.03
**Variant b (12 sensors)**												
MP-1	SM_R2C7	3.73×10^{5}	2.674×10^{-2}	71.6	36.40	1.19	0.00	0.00	0.08	0.23	0.23	0.18
MP-1	SM_R5C4	6.20×10^{5}	3.016×10^{-2}	48.7	56.26	1.15	0.00	0.02	0.16	0.31	0.25	0.18
MP-1	SM_R8C1	8.40×10^{5}	3.422×10^{-2}	40.7	41.32	1.09	0.00	0.06	0.24	0.32	0.30	0.16
MP-2	SM_R2C7	3.30×10^{5}	2.700×10^{-2}	81.8	21.43	1.19	0.00	0.00	0.08	0.35	0.43	0.31
MP-2	SM_R5C4	3.86×10^{5}	2.789×10^{-2}	72.2	46.67	1.18	0.00	0.00	0.08	0.40	0.31	0.19
MP-2	SM_R8C1	6.49×10^{5}	3.396×10^{-2}	52.3	46.68	1.09	0.00	0.05	0.18	0.50	0.41	0.22
MP-3	SM_R2C7	3.18×10^{5}	2.306×10^{-2}	72.5	22.55	1.10	0.14	0.06	0.09	0.34	0.45	0.24
MP-3	SM_R5C4	3.76×10^{5}	2.709×10^{-2}	72.0	57.43	1.16	0.00	0.00	0.08	0.37	0.31	0.22
MP-3	SM_R8C1	6.17×10^{5}	3.063×10^{-2}	49.7	58.88	1.05	0.07	0.05	0.20	0.54	0.42	0.25
MP-4	SM_R2C7	2.83×10^{5}	2.302×10^{-2}	81.4	24.63	1.10	0.04	0.02	0.10	0.34	0.29	0.35
MP-4	SM_R5C4	3.95×10^{5}	2.495×10^{-2}	63.2	55.15	1.15	0.01	0.04	0.09	0.39	0.28	0.26
MP-4	SM_R8C1	6.47×10^{5}	2.802×10^{-2}	43.3	55.64	1.01	0.02	0.09	0.21	0.57	0.40	0.28

Kinetic parameters for compound 19. SP = singleplex, MP-1/2/3/4 = multiplexed chip SCK-1/2/3/4. $f_{s,i}$ = non-dissociating fraction for cycle i.

Compound 20

Source	Sensor	k_a ($M^{-1}s^{-1}$)	k_d (s^{-1})	K_D (nM)	R_{max} (pg/mm^2)	$R_{max,n}$	$f_{s,1}$	$f_{s,2}$	$f_{s,3}$	$f_{s,4}$	$f_{s,5}$	$f_{s,6}$
**Variant a (54 sensors)**												
SP	SM_R1C1	9.32×10^{5}	3.638×10^{-2}	39.0	5.48	0.99	0.62	0.37	0.41	0.41	0.18	0.06
SP	SM_R1C2	7.15×10^{5}	3.480×10^{-2}	48.7	9.25	1.09	0.26	0.31	0.39	0.36	0.22	0.14
SP	SM_R1C3	7.40×10^{5}	3.932×10^{-2}	53.2	15.27	1.11	0.17	0.25	0.35	0.36	0.23	0.16
SP	SM_R1C4	7.65×10^{5}	4.073×10^{-2}	53.3	11.91	1.11	0.19	0.26	0.31	0.37	0.26	0.16
SP	SM_R1C5	6.79×10^{5}	3.923×10^{-2}	57.8	11.55	1.11	0.21	0.24	0.29	0.36	0.24	0.13
SP	SM_R1C6	7.00×10^{5}	3.854×10^{-2}	55.0	13.45	1.11	0.30	0.31	0.34	0.39	0.26	0.15
SP	SM_R1C7	7.19×10^{5}	3.627×10^{-2}	50.5	13.30	1.08	0.29	0.27	0.34	0.38	0.27	0.16
SP	SM_R1C8	6.91×10^{5}	3.728×10^{-2}	54.0	11.32	1.10	0.34	0.26	0.38	0.38	0.25	0.18
SP	SM_R1C9	7.11×10^{5}	4.182×10^{-2}	58.8	8.93	1.12	0.33	0.22	0.32	0.38	0.22	0.13
SP	SM_R2C1	7.99×10^{5}	3.627×10^{-2}	45.4	12.07	1.08	0.24	0.34	0.37	0.38	0.24	0.13
SP	SM_R2C2	7.72×10^{5}	4.062×10^{-2}	52.6	13.92	1.12	0.16	0.25	0.33	0.35	0.22	0.12
SP	SM_R2C3	7.50×10^{5}	3.840×10^{-2}	51.2	15.80	1.10	0.24	0.28	0.34	0.36	0.23	0.13
SP	SM_R2C4	7.57×10^{5}	3.954×10^{-2}	52.2	14.11	1.11	0.22	0.28	0.34	0.37	0.24	0.15
SP	SM_R2C5	7.84×10^{5}	3.958×10^{-2}	50.5	11.47	1.10	0.18	0.26	0.33	0.35	0.25	0.15
SP	SM_R2C6	7.11×10^{5}	3.988×10^{-2}	56.1	10.76	1.12	0.20	0.17	0.30	0.40	0.25	0.14
SP	SM_R2C7	6.68×10^{5}	3.860×10^{-2}	57.8	12.38	1.13	0.21	0.22	0.30	0.36	0.26	0.17
SP	SM_R2C8	6.13×10^{5}	3.850×10^{-2}	62.8	12.78	1.11	0.22	0.22	0.31	0.36	0.25	0.18
SP	SM_R2C9	6.35×10^{5}	4.198×10^{-2}	66.1	11.13	1.12	0.15	0.16	0.27	0.34	0.23	0.17
SP	SM_R3C1	7.58×10^{5}	3.805×10^{-2}	50.2	12.47	1.09	0.20	0.27	0.32	0.36	0.21	0.11
SP	SM_R3C2	7.45×10^{5}	3.983×10^{-2}	53.4	10.71	1.11	0.19	0.23	0.33	0.35	0.21	0.13
SP	SM_R3C3	7.76×10^{5}	3.906×10^{-2}	50.4	13.34	1.11	0.28	0.29	0.34	0.37	0.27	0.18
SP	SM_R3C4	6.34×10^{5}	3.650×10^{-2}	57.6	14.76	1.12	0.23	0.22	0.30	0.36	0.23	0.16
SP	SM_R3C5	5.97×10^{5}	3.554×10^{-2}	59.6	12.77	1.11	0.19	0.18	0.30	0.38	0.23	0.15
SP	SM_R3C6	6.86×10^{5}	4.059×10^{-2}	59.2	10.16	1.10	0.16	0.23	0.29	0.35	0.26	0.17
SP	SM_R3C7	6.04×10^{5}	3.653×10^{-2}	60.5	10.64	1.10	0.22	0.17	0.28	0.38	0.24	0.15
SP	SM_R3C8	6.16×10^{5}	3.875×10^{-2}	62.9	11.25	1.11	0.13	0.17	0.25	0.30	0.22	0.15
SP	SM_R3C9	6.17×10^{5}	4.058×10^{-2}	65.8	11.84	1.10	0.00	0.14	0.25	0.29	0.21	0.16
SP	SM_R4C1	7.55×10^{5}	3.605×10^{-2}	47.7	15.88	1.09	0.26	0.26	0.33	0.36	0.22	0.11
SP	SM_R4C2	7.18×10^{5}	3.809×10^{-2}	53.0	12.04	1.12	0.28	0.23	0.32	0.36	0.21	0.12
SP	SM_R4C3	6.83×10^{5}	3.902×10^{-2}	57.1	10.37	1.11	0.17	0.24	0.31	0.33	0.20	0.14
SP	SM_R4C4	7.62×10^{5}	4.179×10^{-2}	54.9	10.62	1.10	0.11	0.23	0.27	0.35	0.22	0.15
SP	SM_R4C5	8.22×10^{5}	4.034×10^{-2}	49.1	12.52	1.07	0.30	0.35	0.37	0.39	0.25	0.19
SP	SM_R4C6	8.33×10^{5}	4.291×10^{-2}	51.5	11.22	1.08	0.31	0.28	0.35	0.36	0.25	0.19
SP	SM_R4C7	6.06×10^{5}	3.774×10^{-2}	62.2	9.29	1.10	0.09	0.17	0.31	0.32	0.23	0.19
SP	SM_R4C8	5.80×10^{5}	3.815×10^{-2}	65.8	9.16	1.11	0.15	0.18	0.25	0.30	0.23	0.15
SP	SM_R4C9	5.88×10^{5}	3.707×10^{-2}	63.0	10.71	1.10	0.26	0.22	0.27	0.32	0.25	0.16
SP	SM_R5C1	6.40×10^{5}	3.421×10^{-2}	53.4	17.01	1.12	0.27	0.26	0.34	0.36	0.20	0.10
SP	SM_R5C2	6.57×10^{5}	3.853×10^{-2}	58.7	16.15	1.12	0.17	0.21	0.32	0.34	0.21	0.12
SP	SM_R5C3	7.17×10^{5}	3.940×10^{-2}	55.0	12.99	1.10	0.30	0.27	0.32	0.35	0.25	0.18
SP	SM_R5C4	6.74×10^{5}	3.670×10^{-2}	54.4	9.48	1.09	0.33	0.22	0.34	0.39	0.23	0.17
SP	SM_R5C5	5.42×10^{5}	3.945×10^{-2}	72.8	11.79	1.09	0.00	0.07	0.19	0.28	0.17	0.11
SP	SM_R5C6	5.92×10^{5}	3.811×10^{-2}	64.4	14.85	1.10	0.17	0.15	0.25	0.31	0.19	0.12
SP	SM_R5C7	6.36×10^{5}	3.928×10^{-2}	61.7	12.64	1.13	0.06	0.18	0.26	0.30	0.23	0.15
SP	SM_R5C8	7.23×10^{5}	4.211×10^{-2}	58.3	10.21	1.09	0.27	0.28	0.33	0.34	0.24	0.19
SP	SM_R5C9	5.93×10^{5}	3.807×10^{-2}	64.2	10.12	1.09	0.20	0.13	0.26	0.34	0.22	0.16
SP	SM_R6C1	5.94×10^{5}	3.360×10^{-2}	56.6	12.90	1.09	0.19	0.28	0.33	0.35	0.22	0.14
SP	SM_R6C2	5.90×10^{5}	3.519×10^{-2}	59.6	16.96	1.09	0.21	0.23	0.32	0.34	0.21	0.15
SP	SM_R6C3	6.30×10^{5}	3.792×10^{-2}	60.2	16.28	1.12	0.23	0.25	0.32	0.34	0.22	0.16
SP	SM_R6C4	6.74×10^{5}	3.768×10^{-2}	55.9	12.69	1.09	0.32	0.26	0.33	0.35	0.23	0.19
SP	SM_R6C5	6.49×10^{5}	4.019×10^{-2}	61.9	10.36	1.09	0.36	0.32	0.35	0.36	0.25	0.20
SP	SM_R6C6	6.24×10^{5}	3.912×10^{-2}	62.7	13.45	1.11	0.28	0.29	0.32	0.35	0.24	0.19
SP	SM_R6C7	6.26×10^{5}	3.911×10^{-2}	62.5	16.50	1.12	0.24	0.24	0.30	0.33	0.25	0.16
SP	SM_R6C8	6.07×10^{5}	3.811×10^{-2}	62.8	14.64	1.10	0.18	0.23	0.29	0.32	0.25	0.20
SP	SM_R6C9	6.96×10^{5}	4.317×10^{-2}	62.1	10.51	1.11	0.49	0.29	0.33	0.38	0.29	0.20
**Variant b (12 sensors)**												
MP-1	SM_R2C4	3.63×10^{5}	3.283×10^{-2}	90.5	63.81	1.24	0.00	0.08	0.22	0.30	0.32	0.19
MP-1	SM_R5C1	3.93×10^{5}	3.521×10^{-2}	89.7	10.23	1.09	0.27	0.38	0.48	0.37	0.52	0.24
MP-1	SM_R7C6	3.93×10^{5}	3.595×10^{-2}	91.5	50.54	1.25	0.00	0.07	0.20	0.26	0.29	0.17
MP-2	SM_R2C4	2.89×10^{5}	3.102×10^{-2}	107.4	42.85	1.25	0.09	0.15	0.24	0.40	0.43	0.25
MP-2	SM_R5C1	4.30×10^{5}	4.463×10^{-2}	103.8	12.46	1.12	0.00	0.22	0.33	0.55	0.71	0.35
MP-2	SM_R7C6	3.36×10^{5}	3.666×10^{-2}	109.0	32.41	1.22	0.01	0.14	0.24	0.37	0.48	0.26
MP-3	SM_R2C4	3.11×10^{5}	2.978×10^{-2}	95.7	45.05	1.18	0.12	0.16	0.26	0.37	0.48	0.25
MP-3	SM_R5C1	5.67×10^{5}	4.292×10^{-2}	75.7	12.56	0.97	0.00	0.25	0.40	0.54	0.77	0.24
MP-3	SM_R7C6	3.55×10^{5}	3.550×10^{-2}	100.0	32.64	1.15	0.00	0.12	0.25	0.36	0.51	0.25
MP-4	SM_R2C4	3.14×10^{5}	2.825×10^{-2}	90.0	43.87	1.20	0.17	0.19	0.24	0.39	0.42	0.31
MP-4	SM_R5C1	3.71×10^{5}	2.978×10^{-2}	80.3	13.14	1.05	0.16	0.26	0.41	0.55	0.55	0.40
MP-4	SM_R7C6	3.28×10^{5}	2.991×10^{-2}	91.1	32.02	1.16	0.06	0.13	0.25	0.39	0.43	0.32

Kinetic parameters for compound 20. SP = singleplex, MP-1/2/3/4 = multiplexed chip SCK-1/2/3/4. $f_{s,i}$ = non-dissociating fraction for cycle i.
